# Supplementary material for: Multi-ancestry genome-wide association meta-analysis of Parkinson’s disease
Source: Nat Genet. 2023 Dec 28;56(1):27–36. doi: 10.1038/s41588-023-01584-8 (PMC10786718; doi:10.1038/s41588-023-01584-8)
Supplement: Supplementary file 1 — Supplementary Figs. 1–12. [file 41588_2023_1584_MOESM1_ESM.pdf]

nature genetics

Article https://doi.org/10.1038/s41588-023-01584-8

Multi-ancestry genome-wide association meta-analysis of Parkinson's disease

In the format provided by the authors and unedited

Supplementary Figures

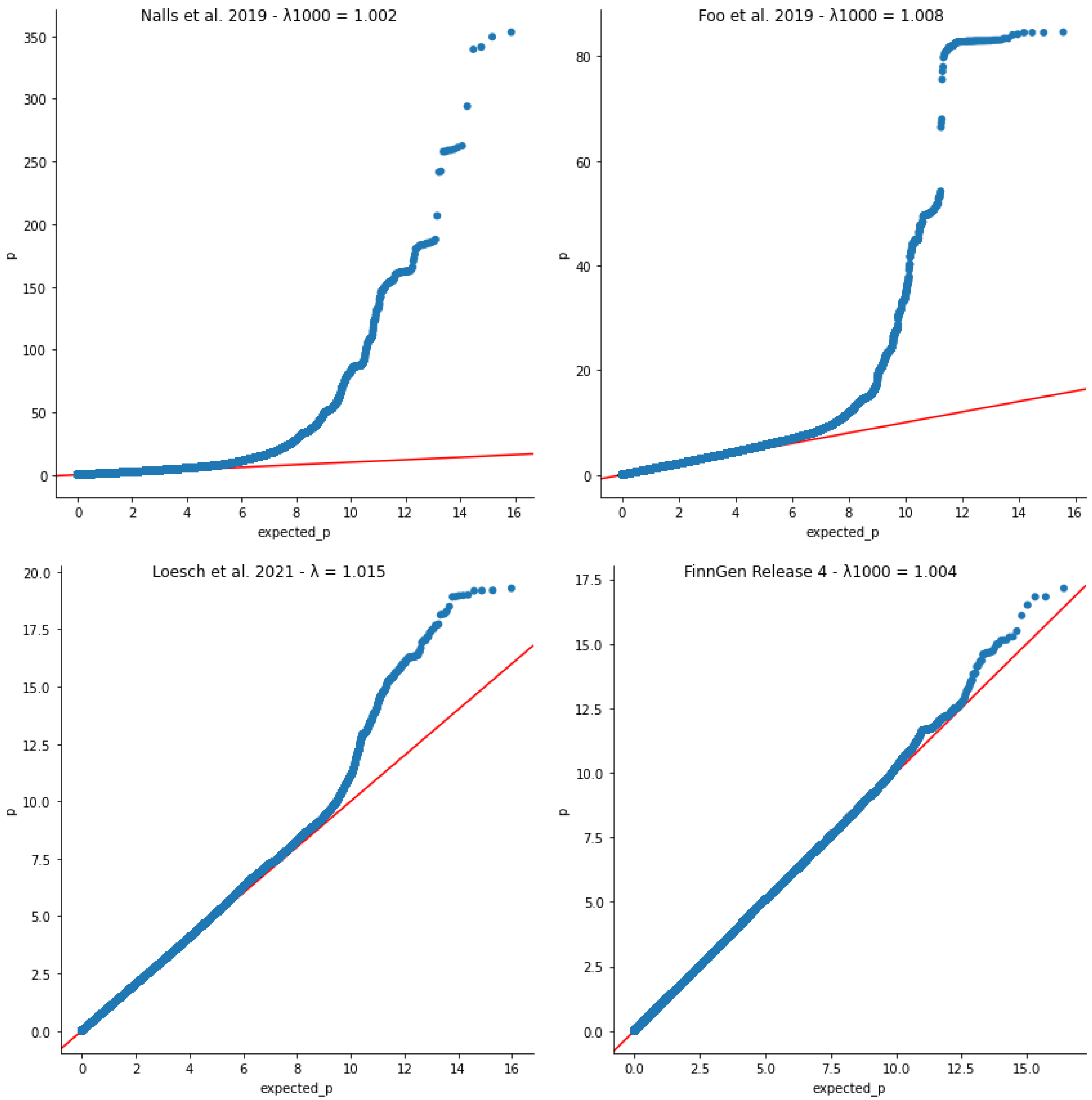

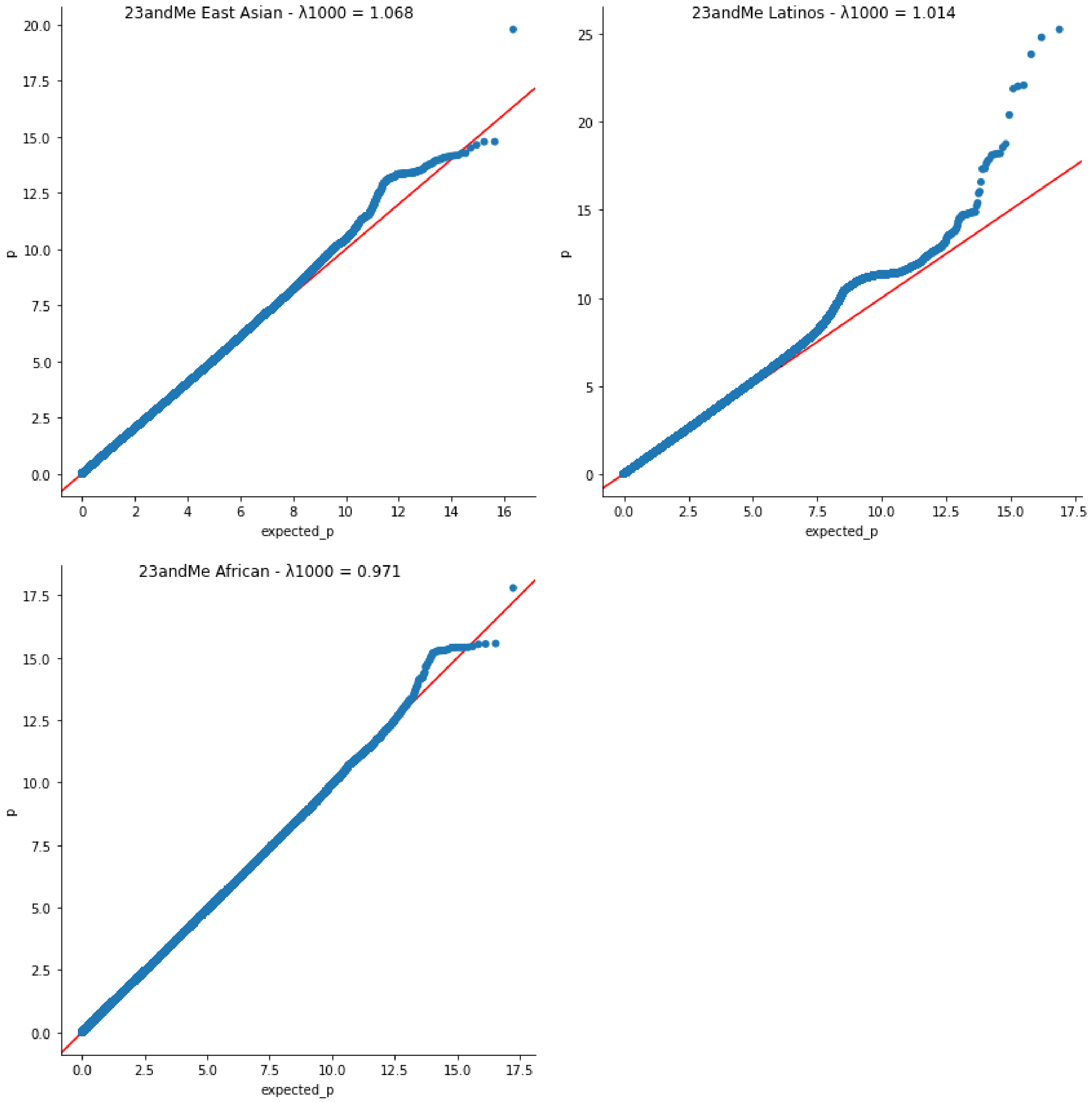

Supplementary Figure 1 – QQ Plots and Genomic Inflation (λ) of each ancestry cohort. Genomic inflation was normalized to 1000 cases and 1000 controls for all datasets outside of Loesch et al 2021 due to large discrepancy between number of cases and controls.

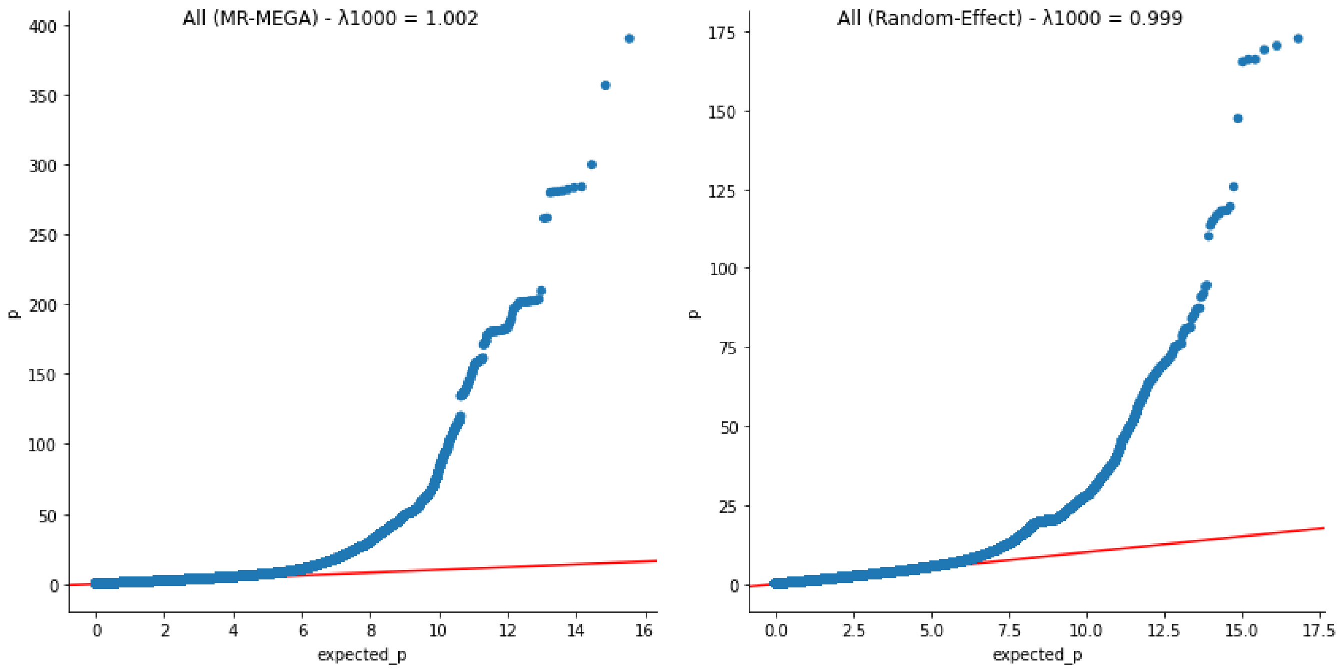

Supplementary Figure 2 – QQ Plots and normalized Genomic Inflation (λ) of the two meta-analyses. Genomic inflation was normalized to 1000 cases and 1000 controls for all datasets due to large discrepancy between number of cases and controls.

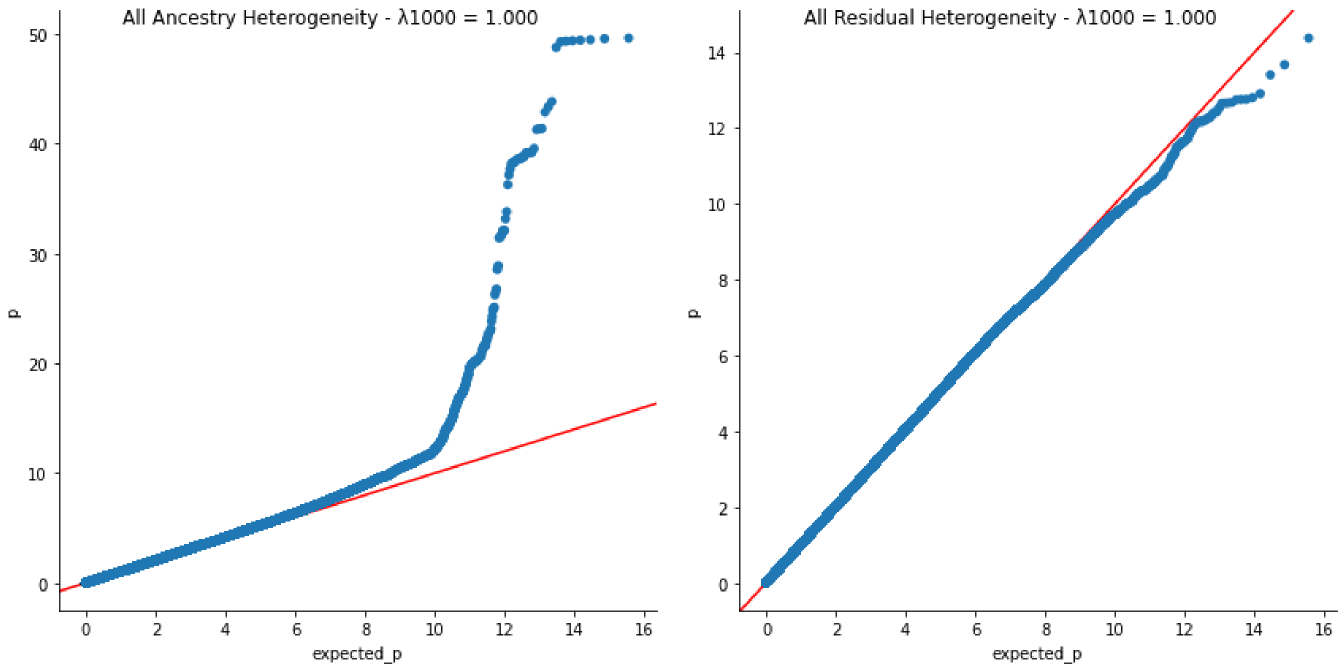

Supplementary Figure 3 - QQ Plot and Genomic Inflation of MR-MEGA ancestry heterogeneity and residual heterogeneity. Genomic inflation was normalized to 1000 cases and 1000 controls.

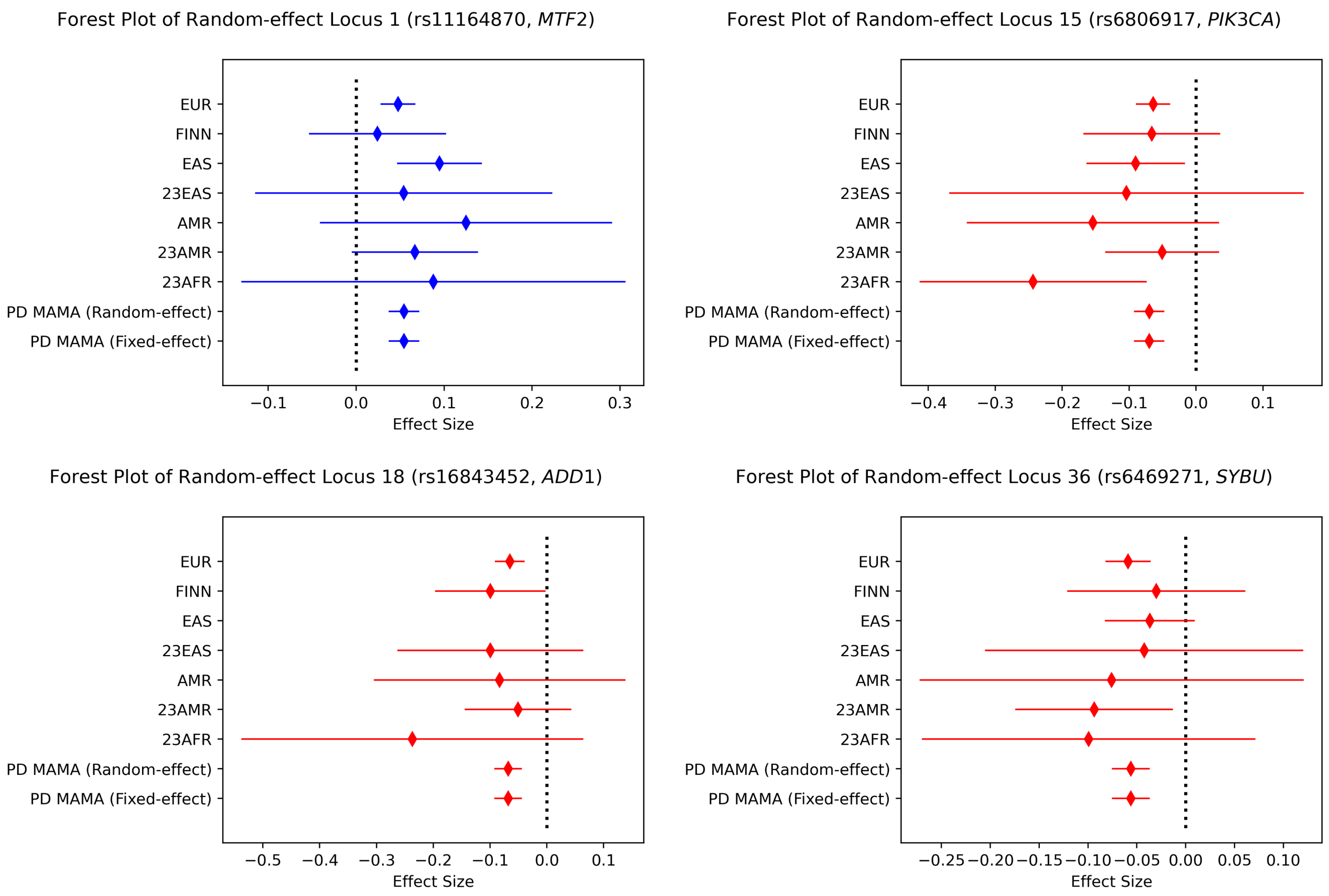
Forest Plot of Random-effect Locus 1 (rs11164870, *MTF2*)
EUR
FINN
EAS
23EAS
AMR
23AMR
23AFR
PD MAMA (Random-effect)
PD MAMA (Fixed-effect)
−0.1 0.0 0.1 0.2 0.3
Effect Size
Forest Plot of Random-effect Locus 15 (rs6806917, *PIK3CA*)
EUR
FINN
EAS
23EAS
AMR
23AMR
23AFR
PD MAMA (Random-effect)
PD MAMA (Fixed-effect)
−0.4 −0.3 −0.2 −0.1 0.0 0.1
Effect Size
Forest Plot of Random-effect Locus 18 (rs16843452, *ADD1*)
EUR
FINN
EAS
23EAS
AMR
23AMR
23AFR
PD MAMA (Random-effect)
PD MAMA (Fixed-effect)
−0.5 −0.4 −0.3 −0.2 −0.1 0.0 0.1
Effect Size
Forest Plot of Random-effect Locus 36 (rs6469271, *SYBU*)
EUR
FINN
EAS
23EAS
AMR
23AMR
23AFR
PD MAMA (Random-effect)
PD MAMA (Fixed-effect)
−0.25 −0.20 −0.15 −0.10 −0.05 0.00 0.05 0.10
Effect Size

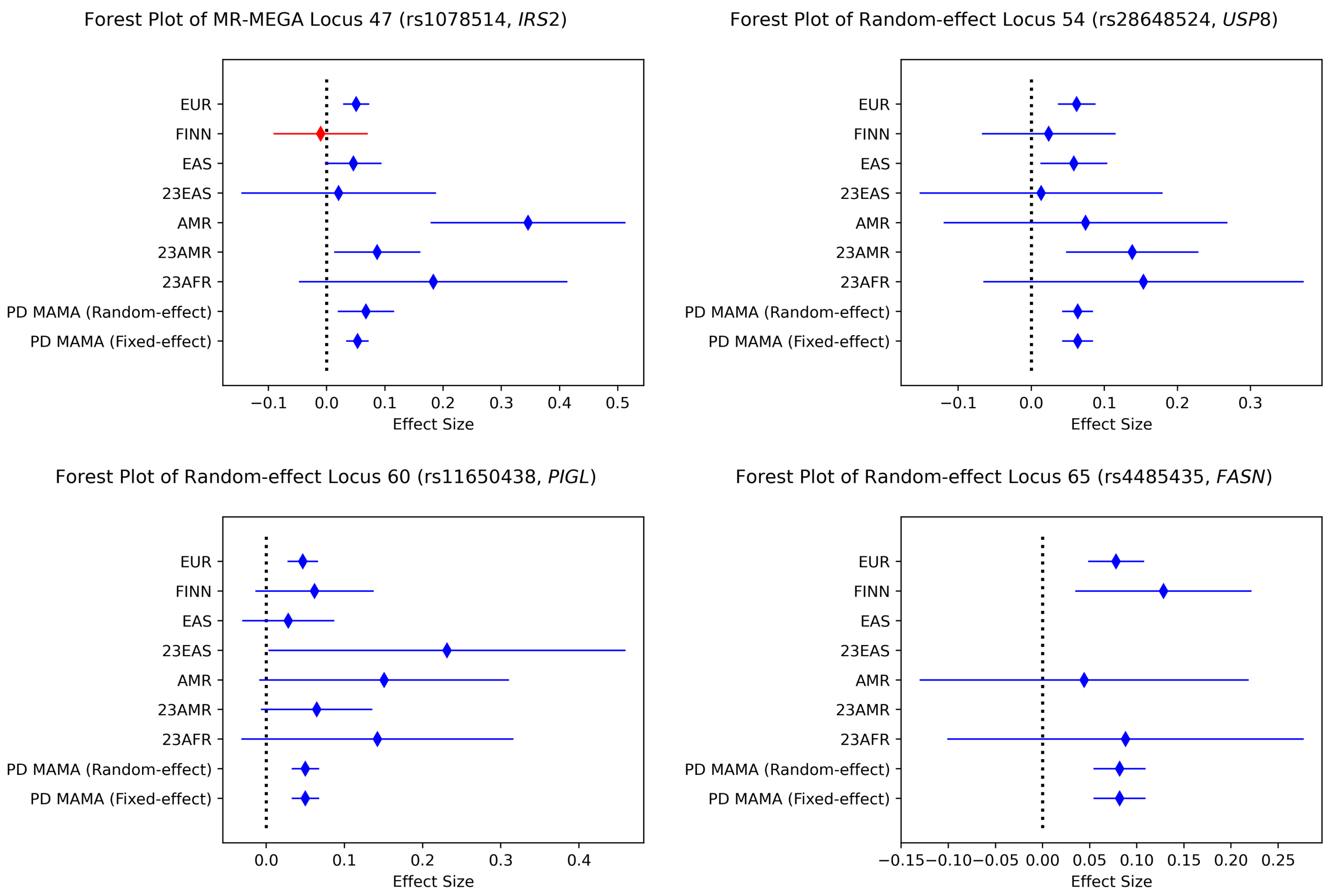

Forest Plot of MR-MEGA Locus 47 (rs1078514, *IRS2*)
EUR
FINN
EAS
23EAS
AMR
23AMR
23AFR
PD MAMA (Random-effect)
PD MAMA (Fixed-effect)
−0.1 0.0 0.1 0.2 0.3 0.4 0.5
Effect Size
Forest Plot of Random-effect Locus 54 (rs28648524, *USP8*)
EUR
FINN
EAS
23EAS
AMR
23AMR
23AFR
PD MAMA (Random-effect)
PD MAMA (Fixed-effect)
−0.1 0.0 0.1 0.2 0.3
Effect Size
Forest Plot of Random-effect Locus 60 (rs11650438, *PIGL*)
EUR
FINN
EAS
23EAS
AMR
23AMR
23AFR
PD MAMA (Random-effect)
PD MAMA (Fixed-effect)
0.0 0.1 0.2 0.3 0.4
Effect Size
Forest Plot of Random-effect Locus 65 (rs4485435, *FASN*)
EUR
FINN
EAS
23EAS
AMR
23AMR
23AFR
PD MAMA (Random-effect)
PD MAMA (Fixed-effect)
−0.15 −0.10 −0.05 0.00 0.05 0.10 0.15 0.20 0.25
Effect Size

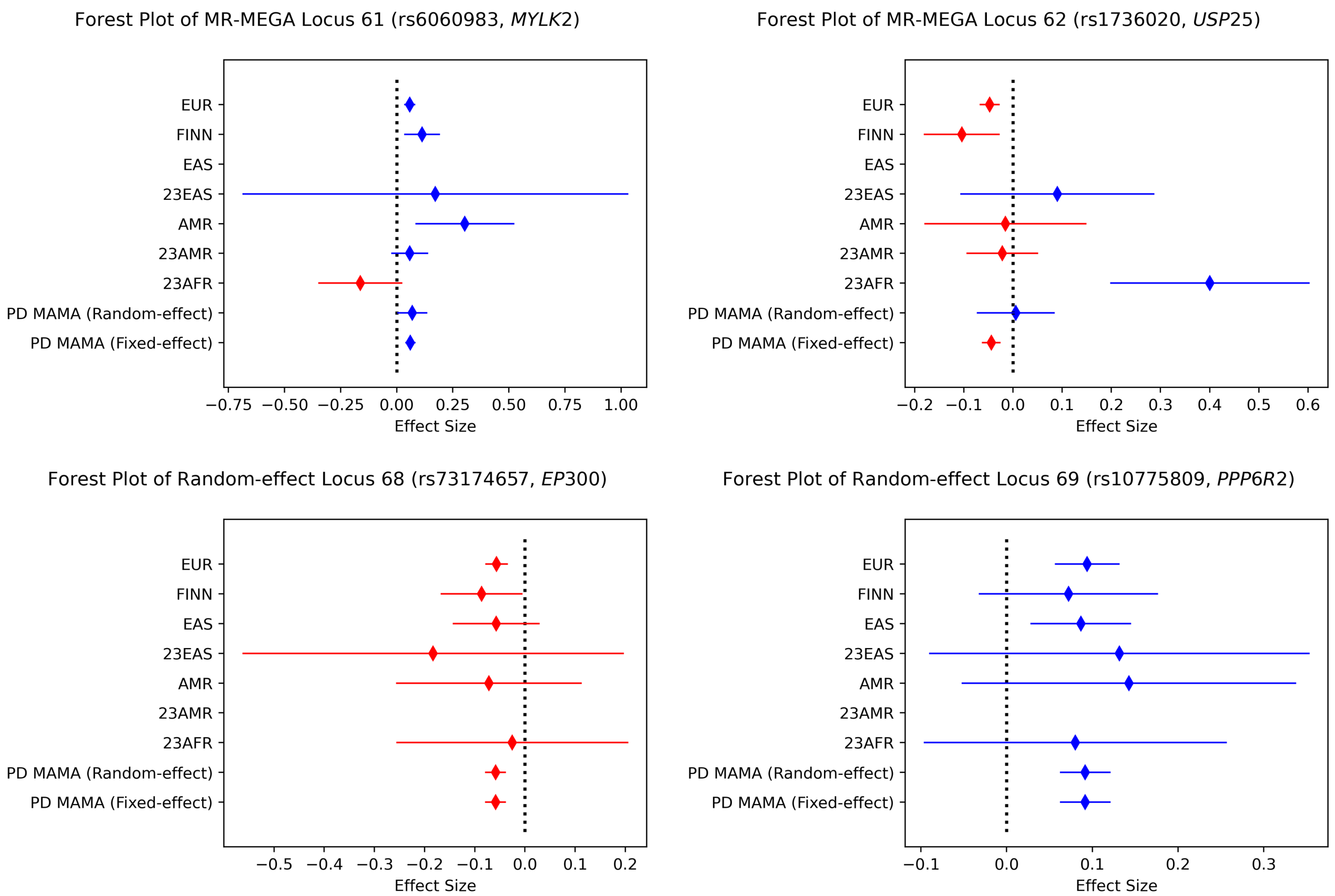

Supplementary Figure 4 - Forest plots of the lead SNPs of novel loci in the ancestrally distinct cohorts and the random effect meta-analysis results. The error bar indicates the 95% confidence interval for the SNP effect size in log odds ratio. MR-MEGA results were not represented as they do not provide effect direction or size. Red represents negative effect direction and blue represents positive effect direction. EUR: Nalls et al. 2019 (n = 1,467,312), FINN: FinnGen R4 (n = 95,683), EAS: Foo et al. 2020 (n= 31,575), 23EAS: 23andMe East Asians (n = 152,227), AMR: Loesch et al. 2021 (n = 1,497), 23AMR: 23andMe Latino (n = 583,163), 23AFR: 23andMe African (n = 194,273), PD MAMA: Multi-ancestry Meta-Analysis (n = 2,525,730)

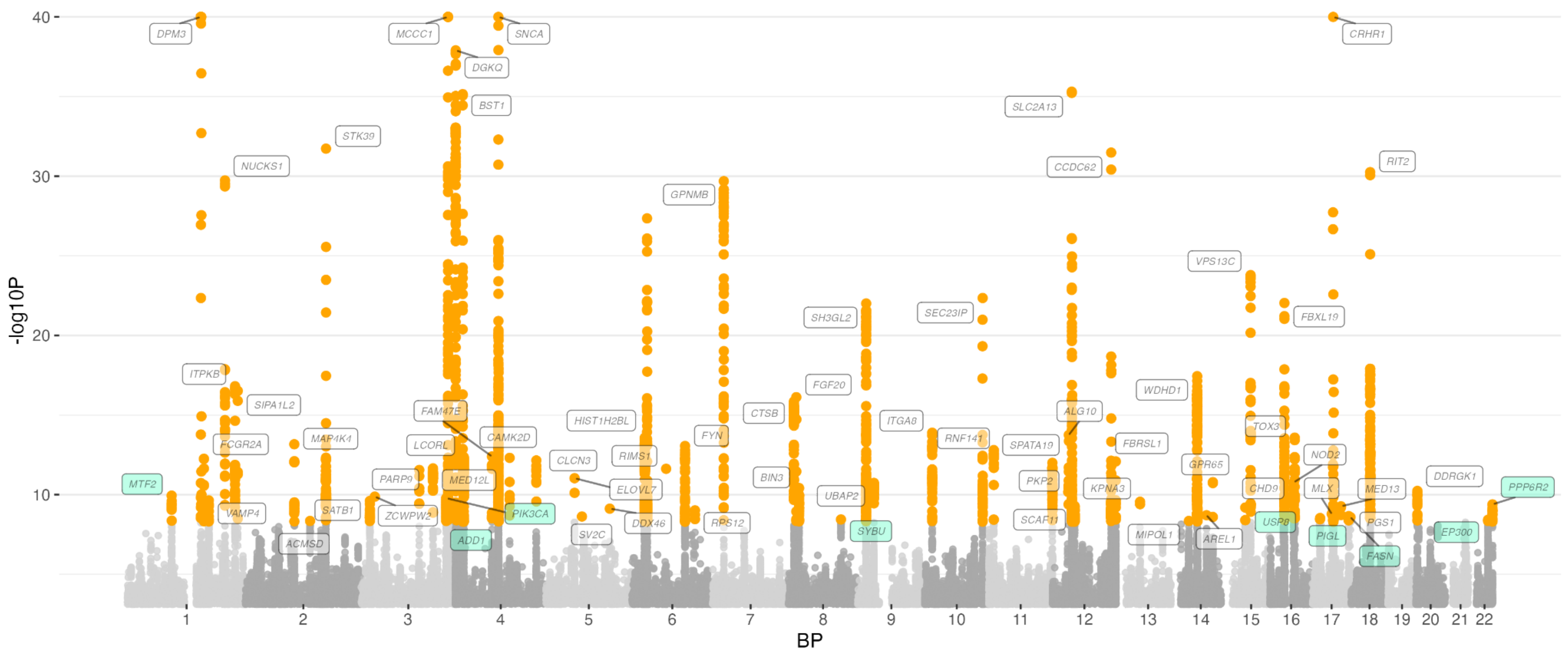

Supplementary Figure 5 - Manhattan plot of the random effect meta-analysis result. The x-axis shows chromosome and base pair positions of each variant tested in the meta-analyses.The y-axis shows the two-sided P value in the -$\log_{10}$ scale. Orange points indicate a significant variant at $P < 5 \times 10^{-9}$. The genomic risk loci are annotated with the nearest protein coding gene, with novel loci highlighted in green.

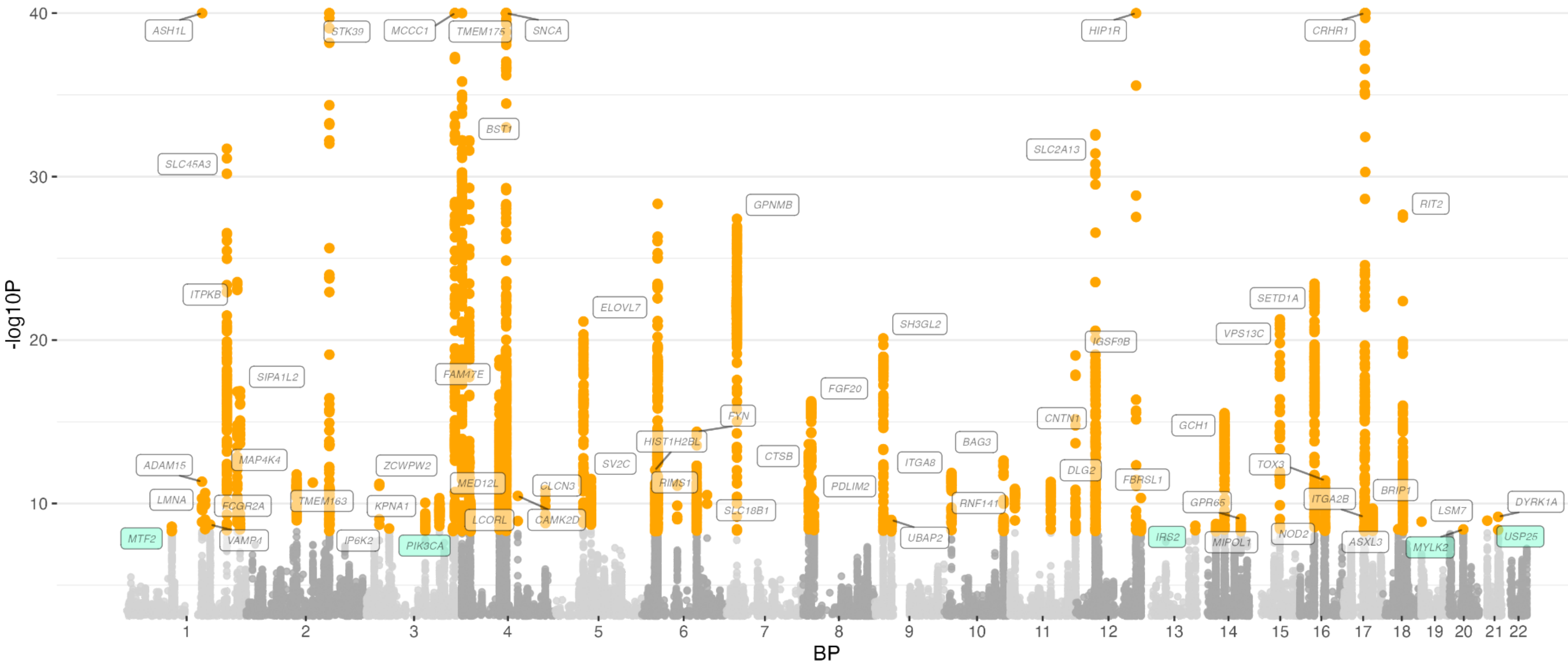

Supplementary Figure 6 - Manhattan plot of the MR-MEGA meta-analysis result. The x-axis shows chromosome and base pair positions of each variant tested in the meta-analyses.The y-axis shows the two-sided P value in the $-\log_{10}$ scale. Orange points indicate a significant variant at $P < 5 \times 10^{-9}$. The genomic risk loci are annotated with the nearest protein coding gene, with novel loci highlighted in green.

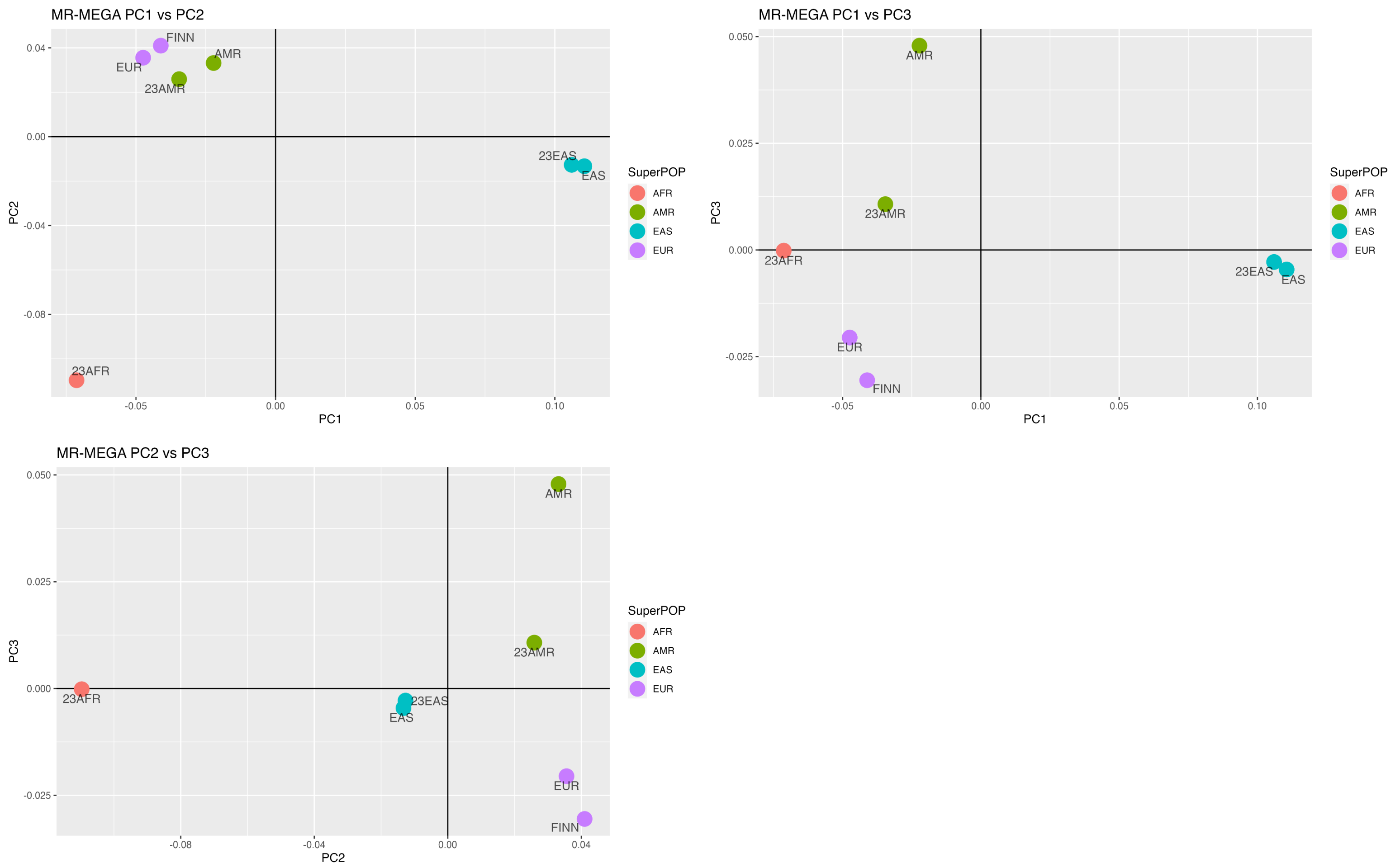

Supplementary Figure 7 - Principal Component of Ancestry Heterogeneity from MR-MEGA.

ADORA2B

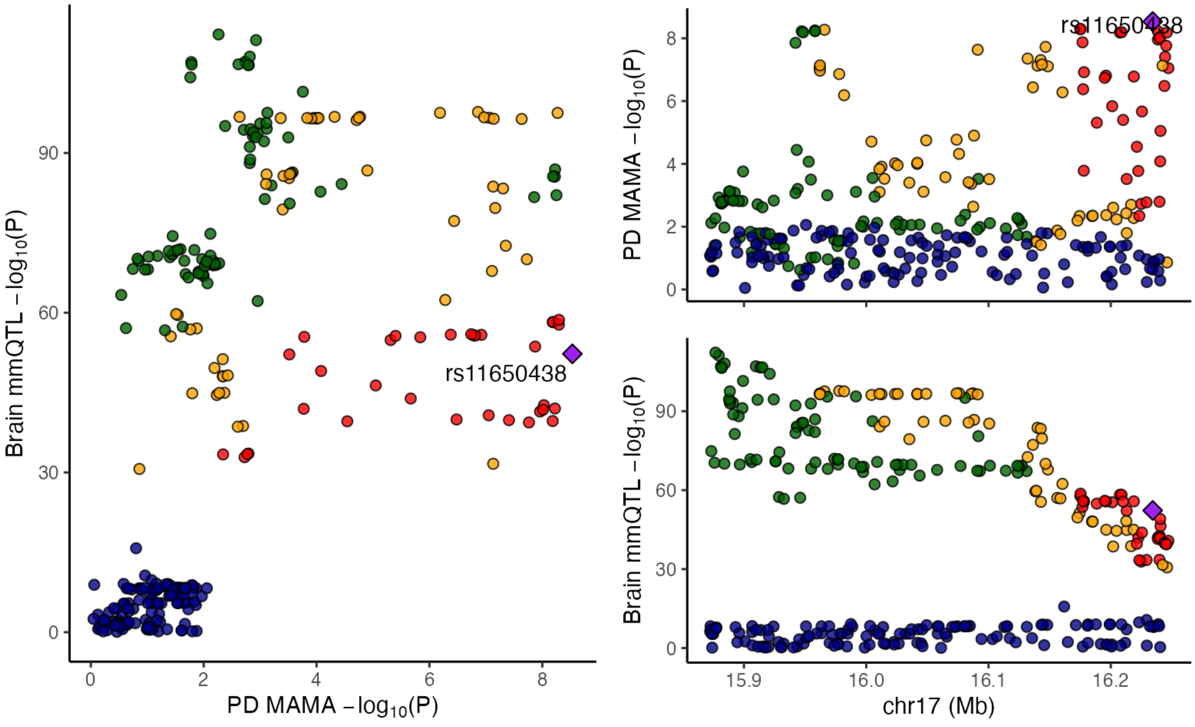

CCDC18-AS1

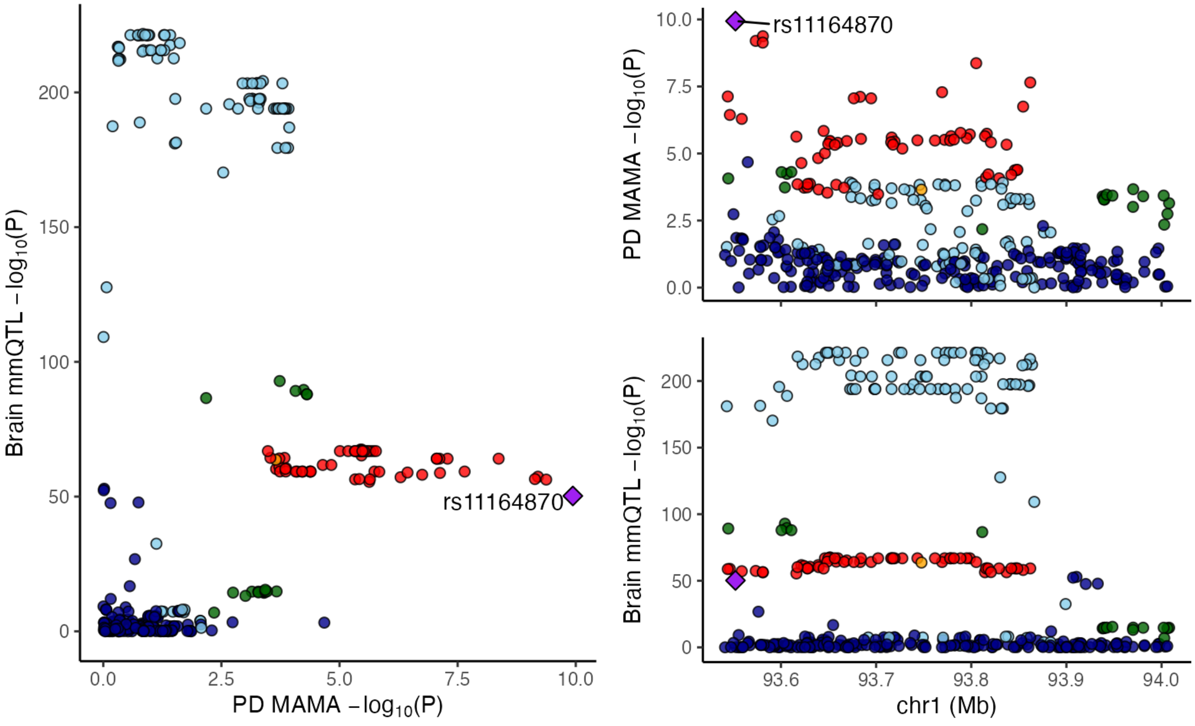

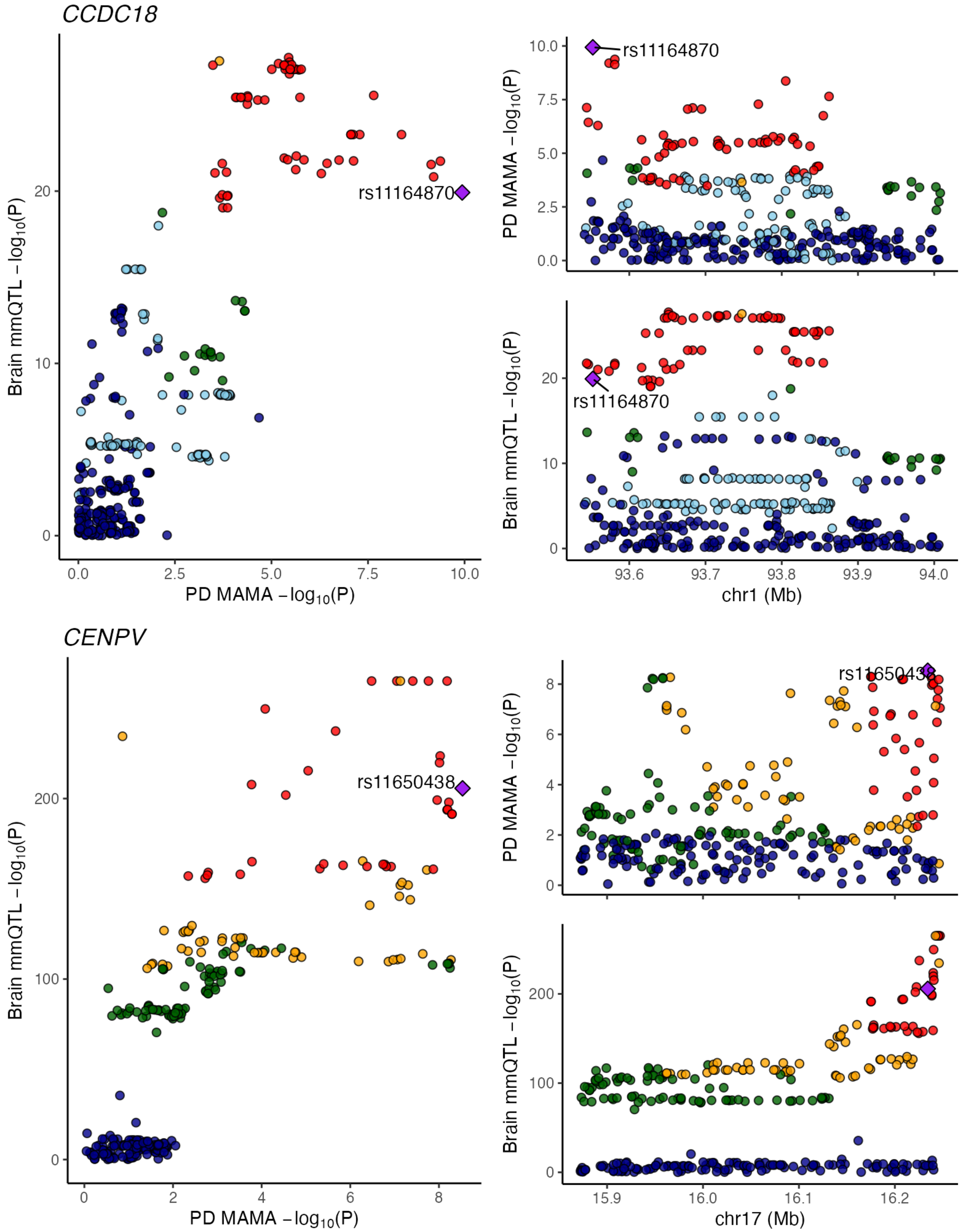
CCDC18
Brain mmQTL −log10(P)
PD MAMA −log10(P)
rs11164870
PD MAMA −log10(P)
rs11164870
Brain mmQTL −log10(P)
rs11164870
chr1 (Mb)
CENPV
Brain mmQTL −log10(P)
PD MAMA −log10(P)
rs11650438
PD MAMA −log10(P)
rs11650438
Brain mmQTL −log10(P)
chr17 (Mb)

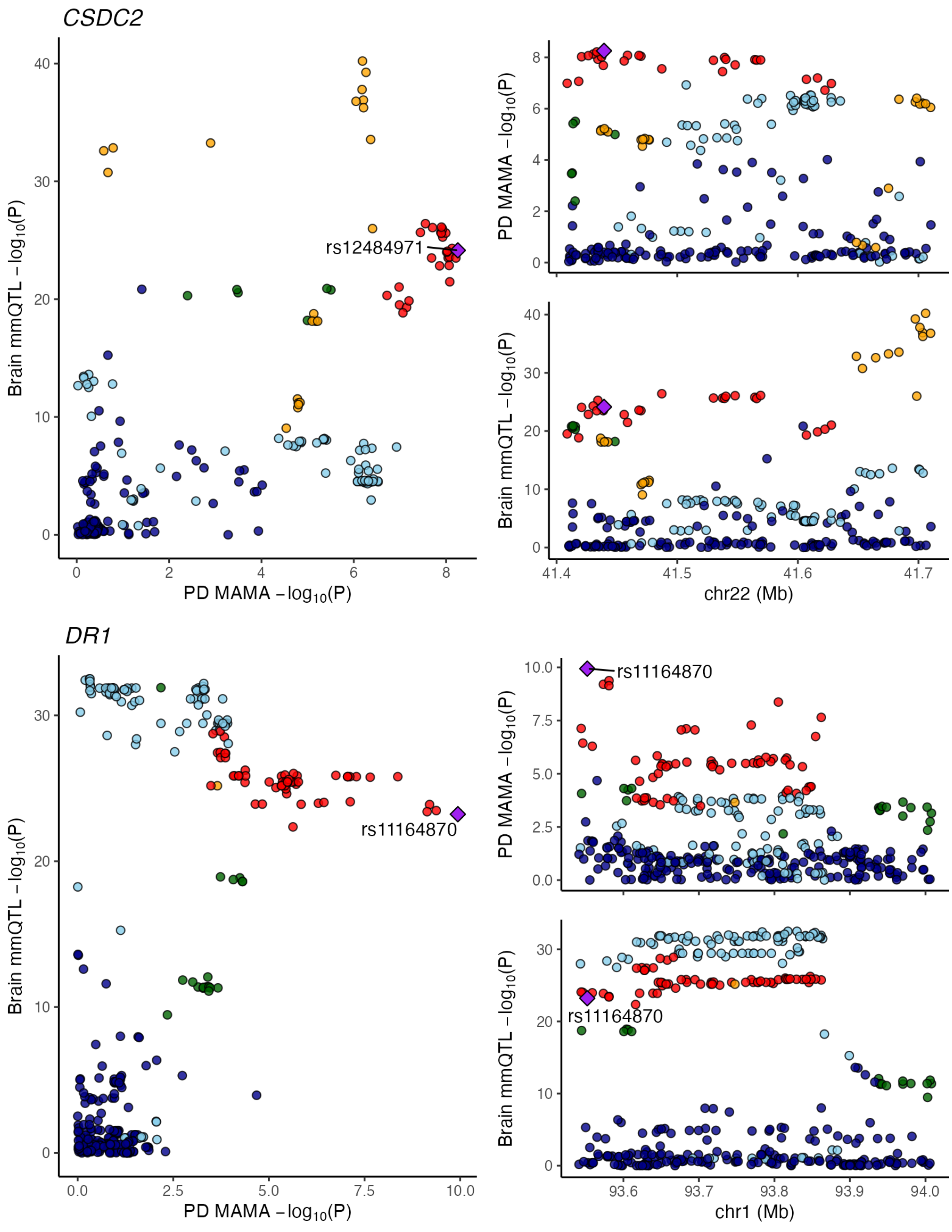
CSDC2
Brain mmQTL −log10(P)
PD MAMA −log10(P)
rs12484971
chr22 (Mb)
DR1
rs11164870
chr1 (Mb)

EP300

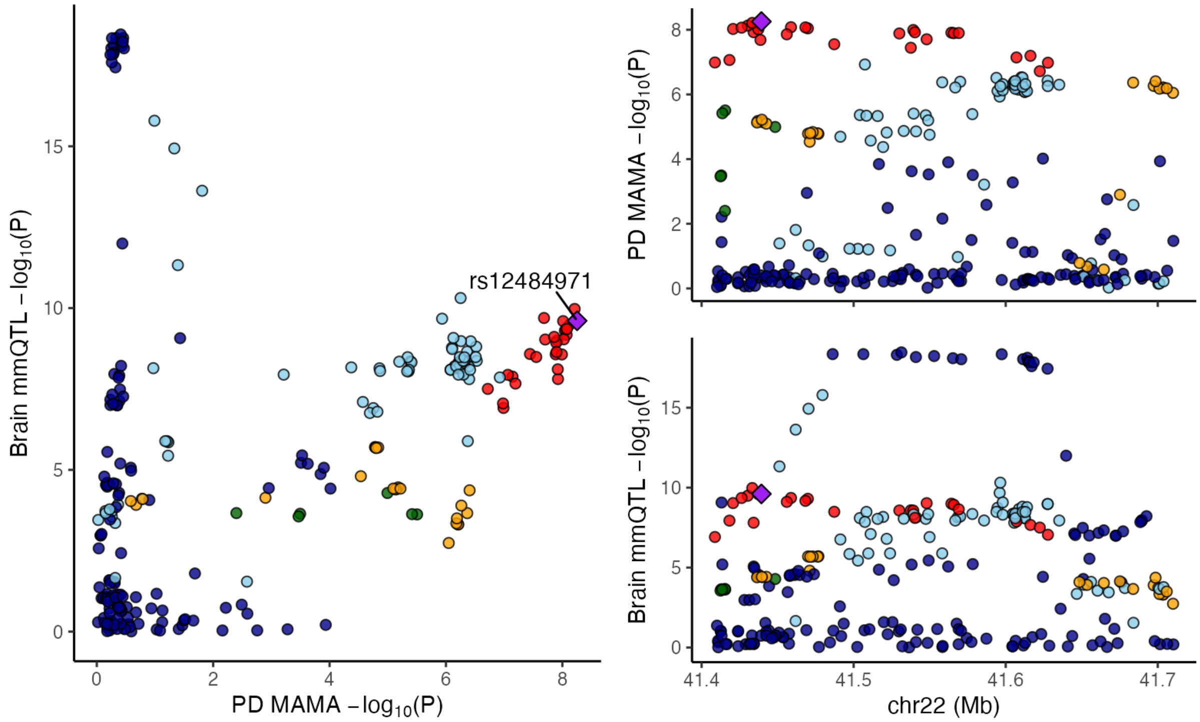

KCNMB3

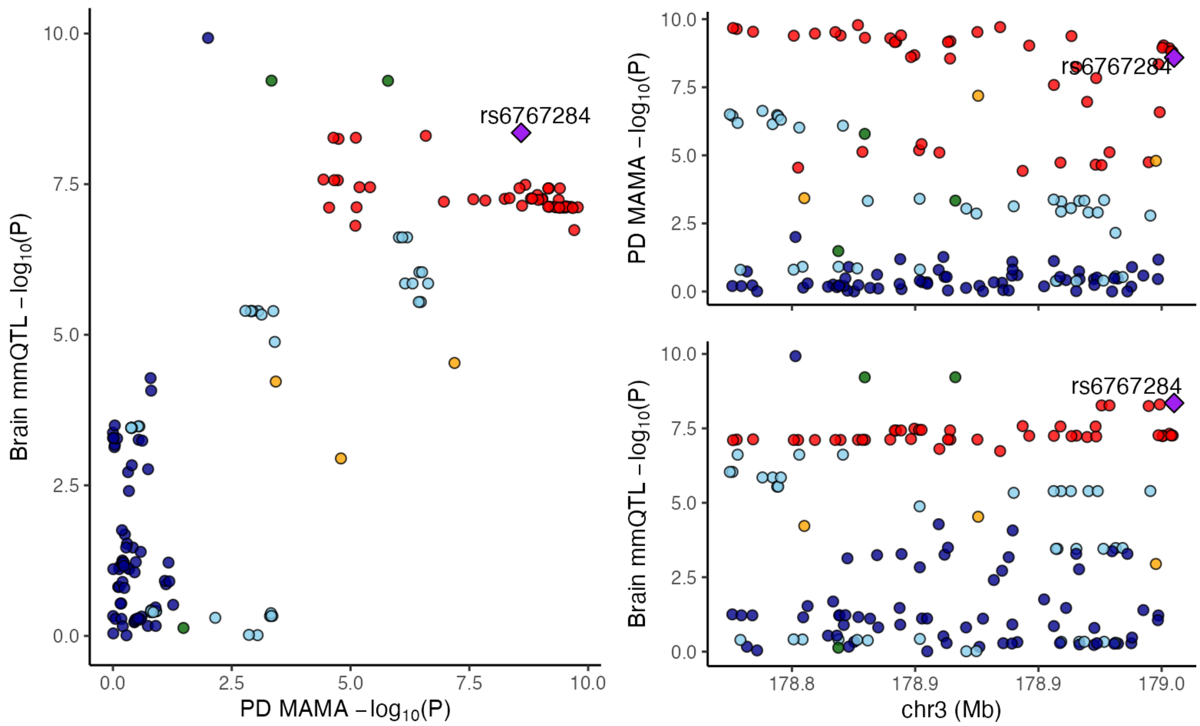

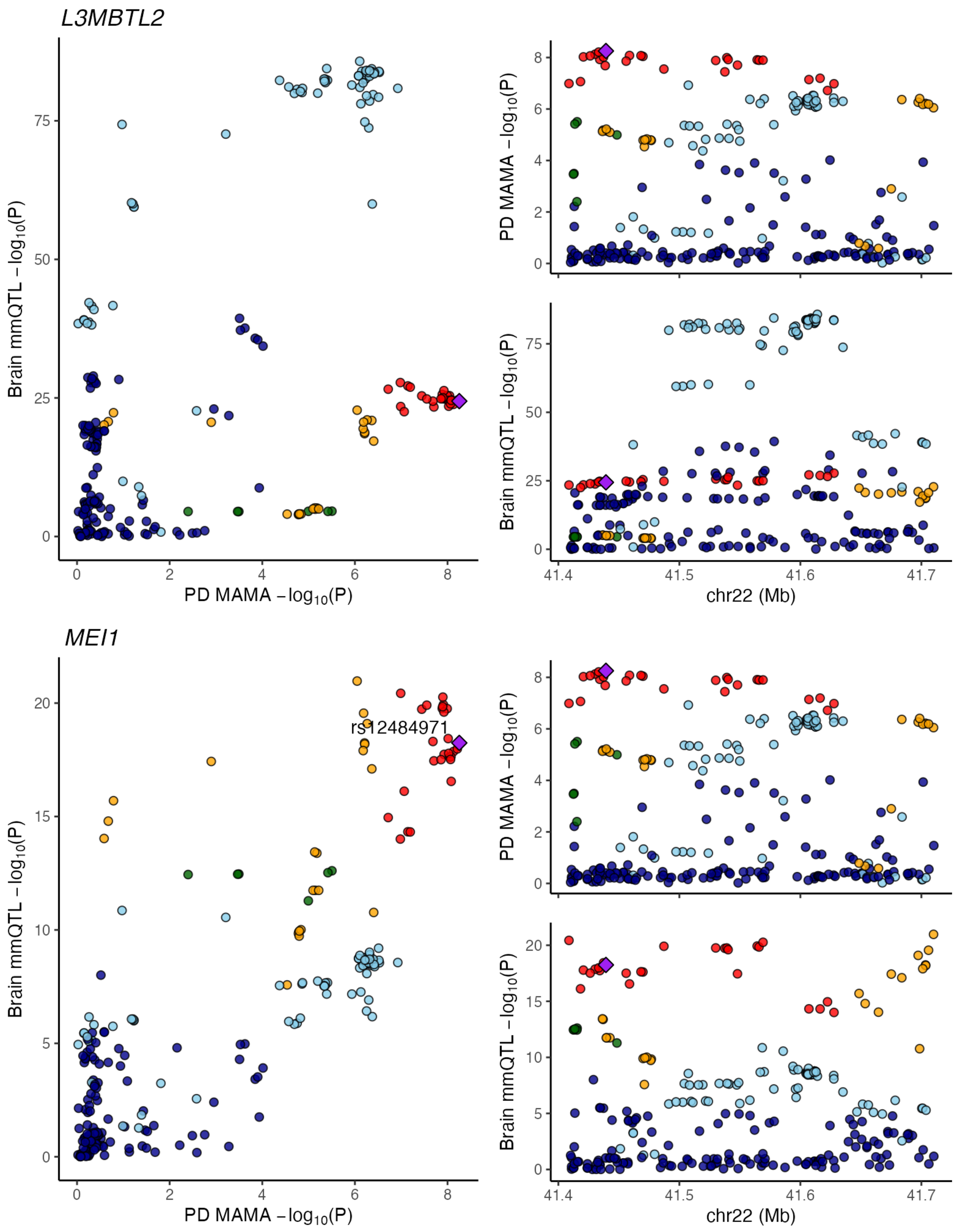

L3MBTL2
Brain mmQTL −log10(P)
PD MAMA −log10(P)
chr22 (Mb)
MEI1
rs12484971

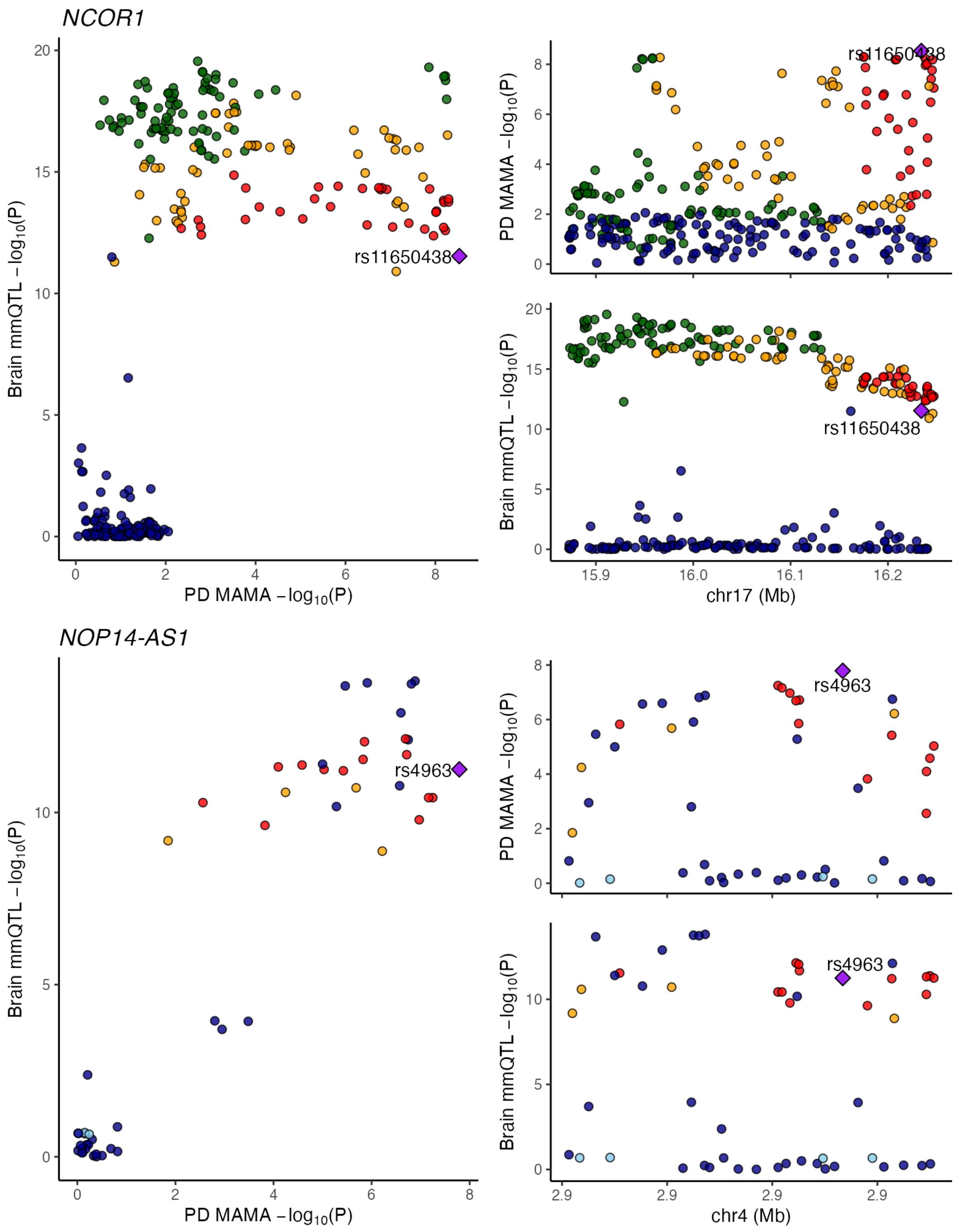

NCOR1
rs11650438
Brain mmQTL $-\log_{10}(P)$
PD MAMA $-\log_{10}(P)$
chr17 (Mb)
15.9
16.0
16.1
16.2
NOP14-AS1
rs4963
chr4 (Mb)
2.9

PIGL

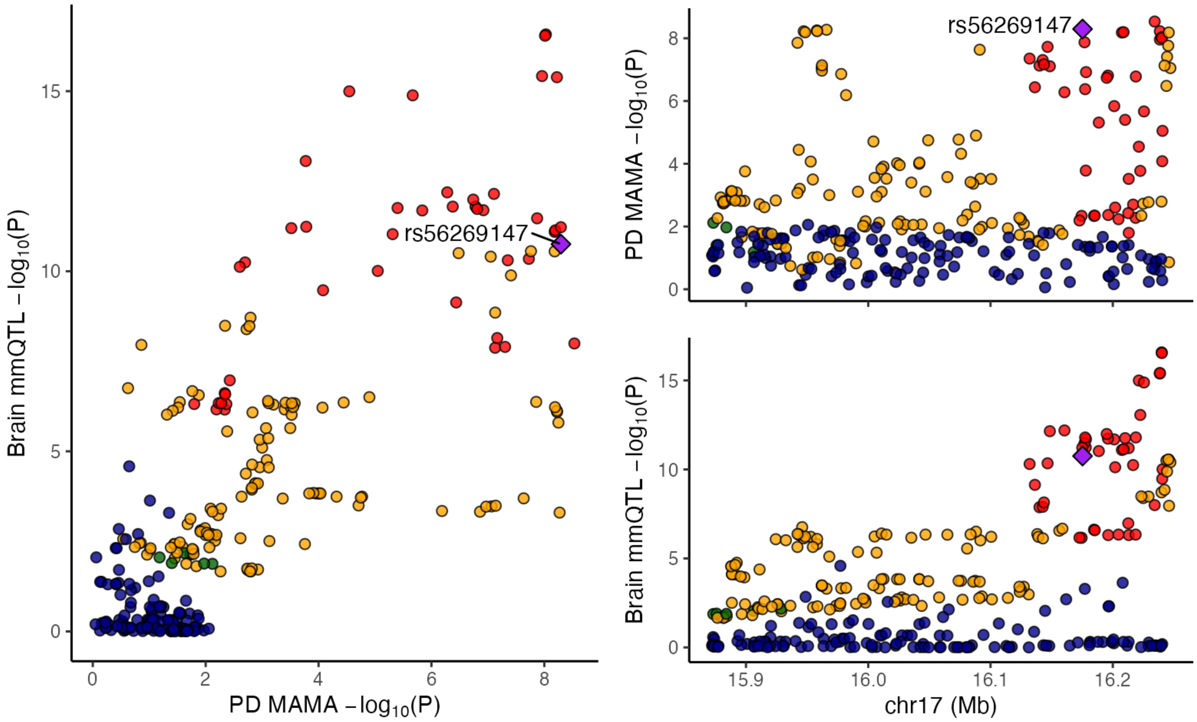
rs56269147
Brain mmQTL $-\log_{10}(P)$
PD MAMA $-\log_{10}(P)$
chr17 (Mb)

PMM1

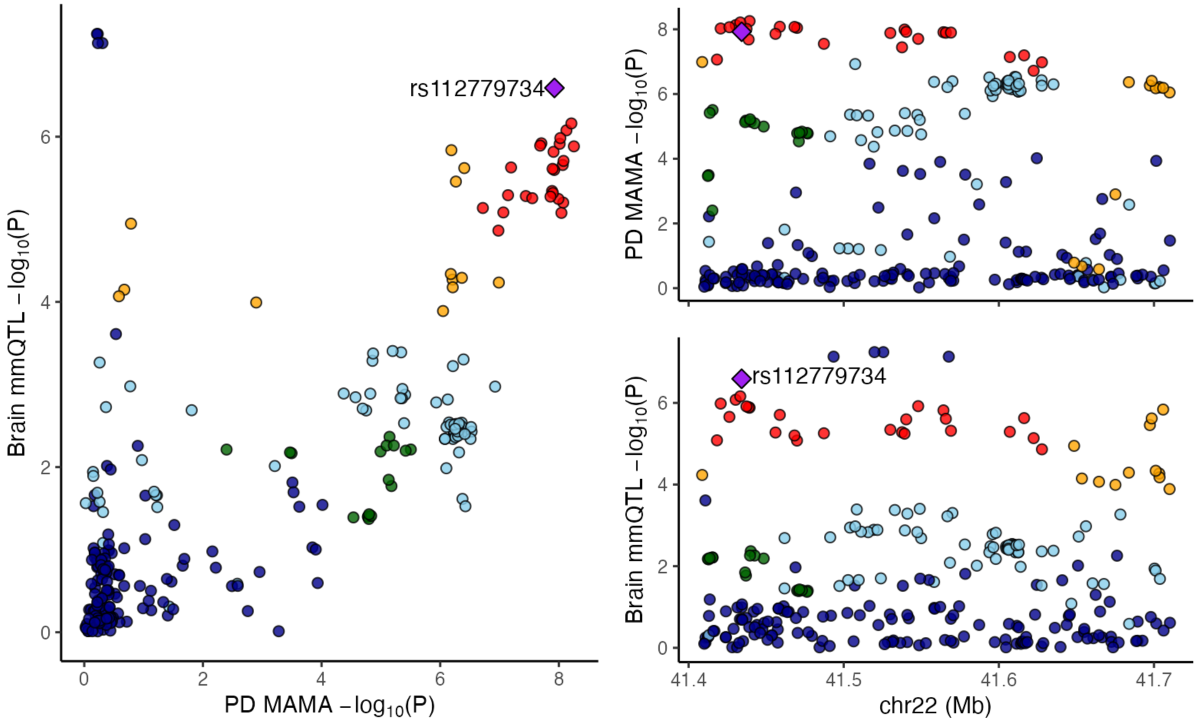
rs112779734
Brain mmQTL $-\log_{10}(P)$
PD MAMA $-\log_{10}(P)$
chr22 (Mb)

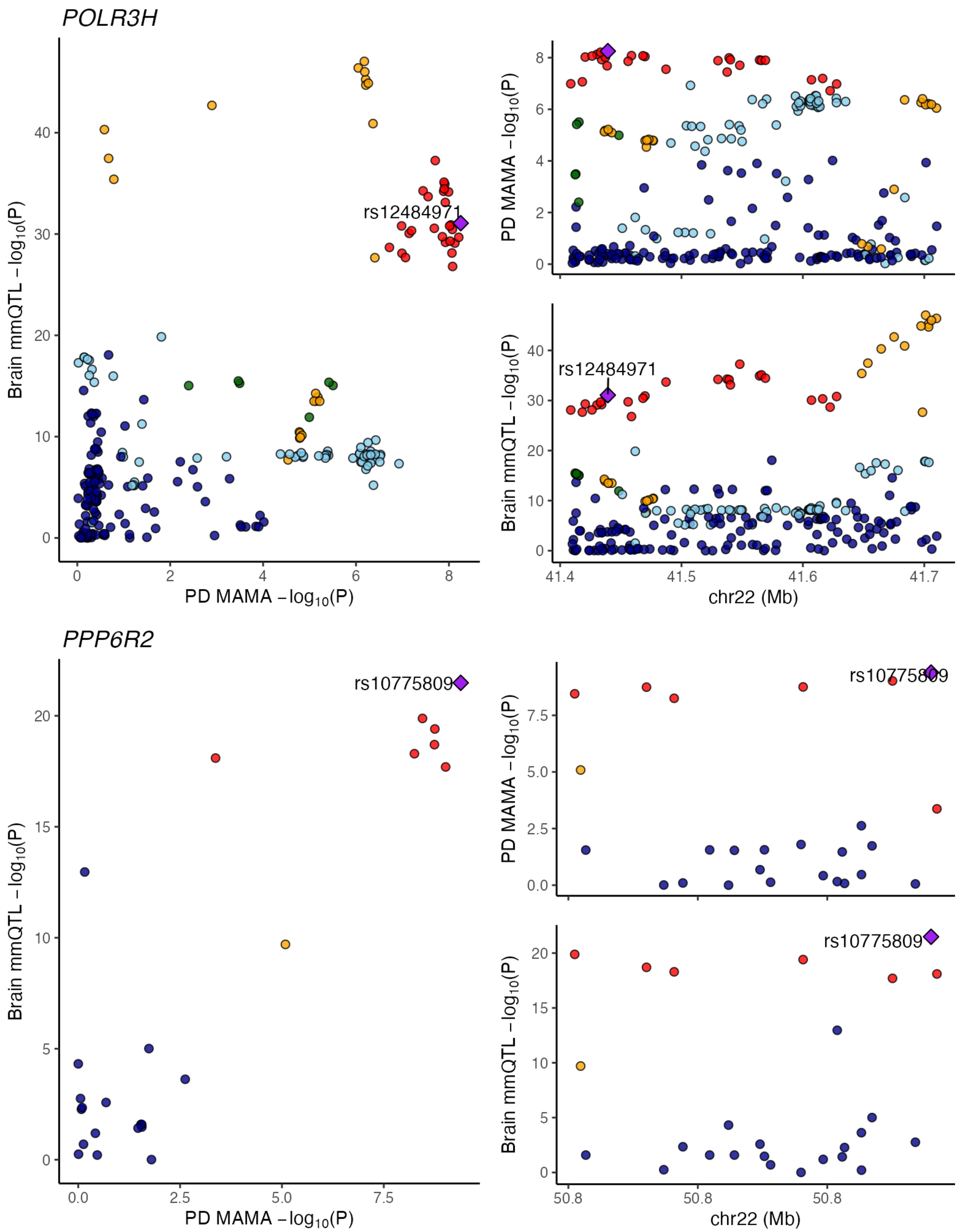

POLR3H
Brain mmQTL −log10(P)
PD MAMA −log10(P)
rs12484971
PD MAMA −log10(P)
Brain mmQTL −log10(P)
rs12484971
chr22 (Mb)
41.4
41.5
41.6
41.7
PPP6R2
Brain mmQTL −log10(P)
PD MAMA −log10(P)
rs10775809
PD MAMA −log10(P)
rs10775809
Brain mmQTL −log10(P)
rs10775809
chr22 (Mb)
50.8
50.8
50.8

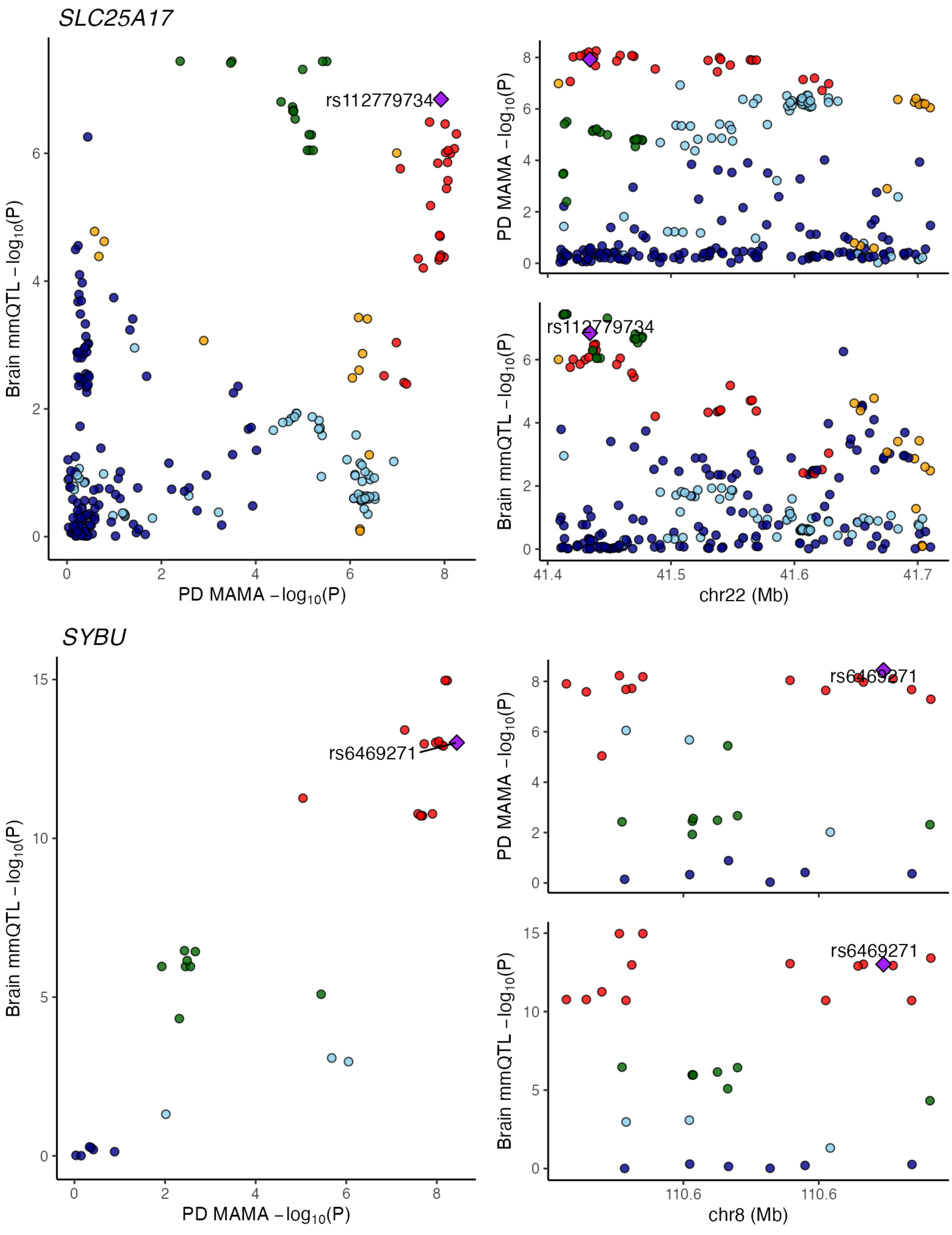

SLC25A17
rs112779734
Brain mmQTL −log10(P)
PD MAMA −log10(P)
chr22 (Mb)
SYBU
rs6469271
chr8 (Mb)

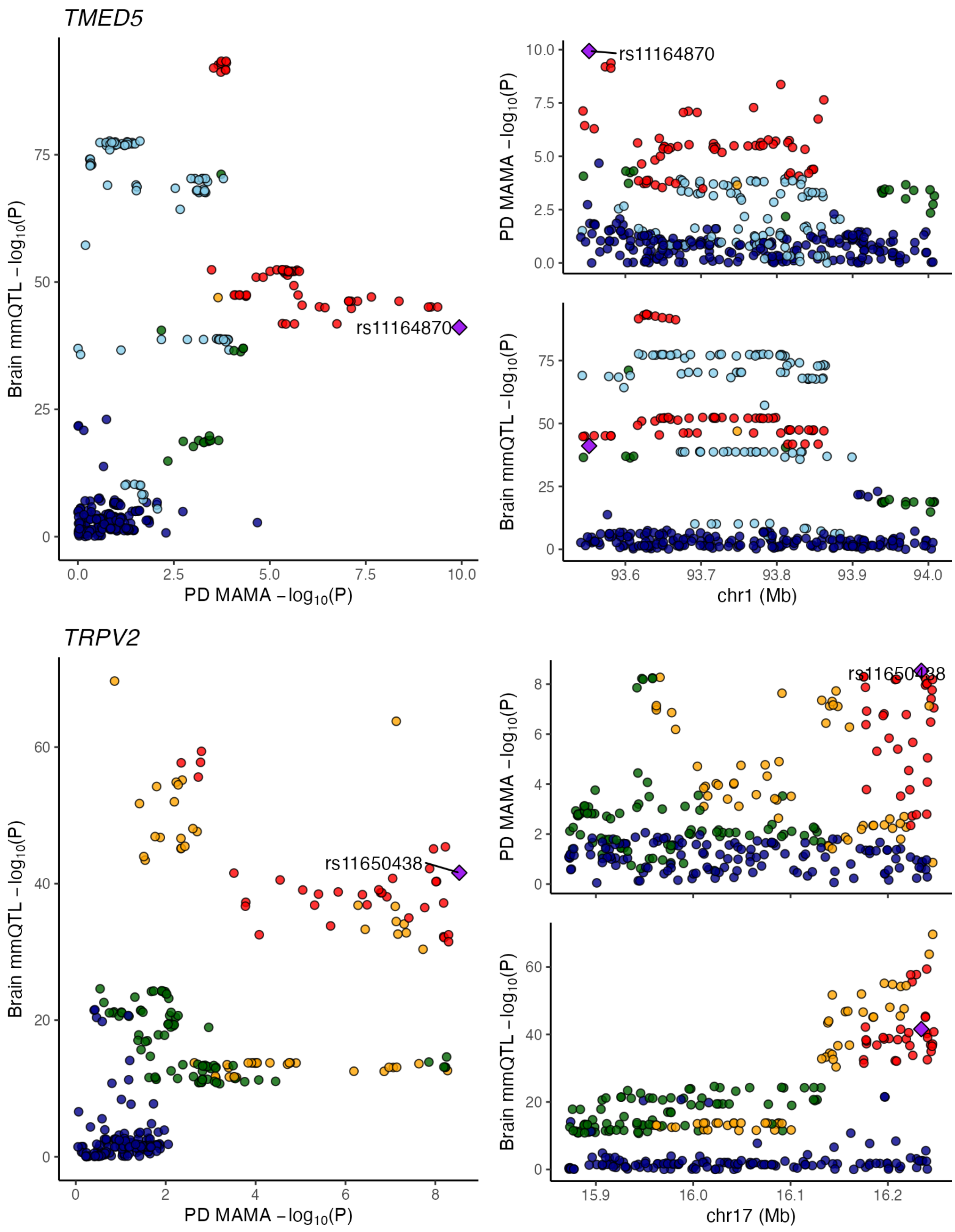
TMED5
Brain mmQTL −log10(P)
PD MAMA −log10(P)
rs11164870
chr1 (Mb)
TRPV2
rs11650438
chr17 (Mb)

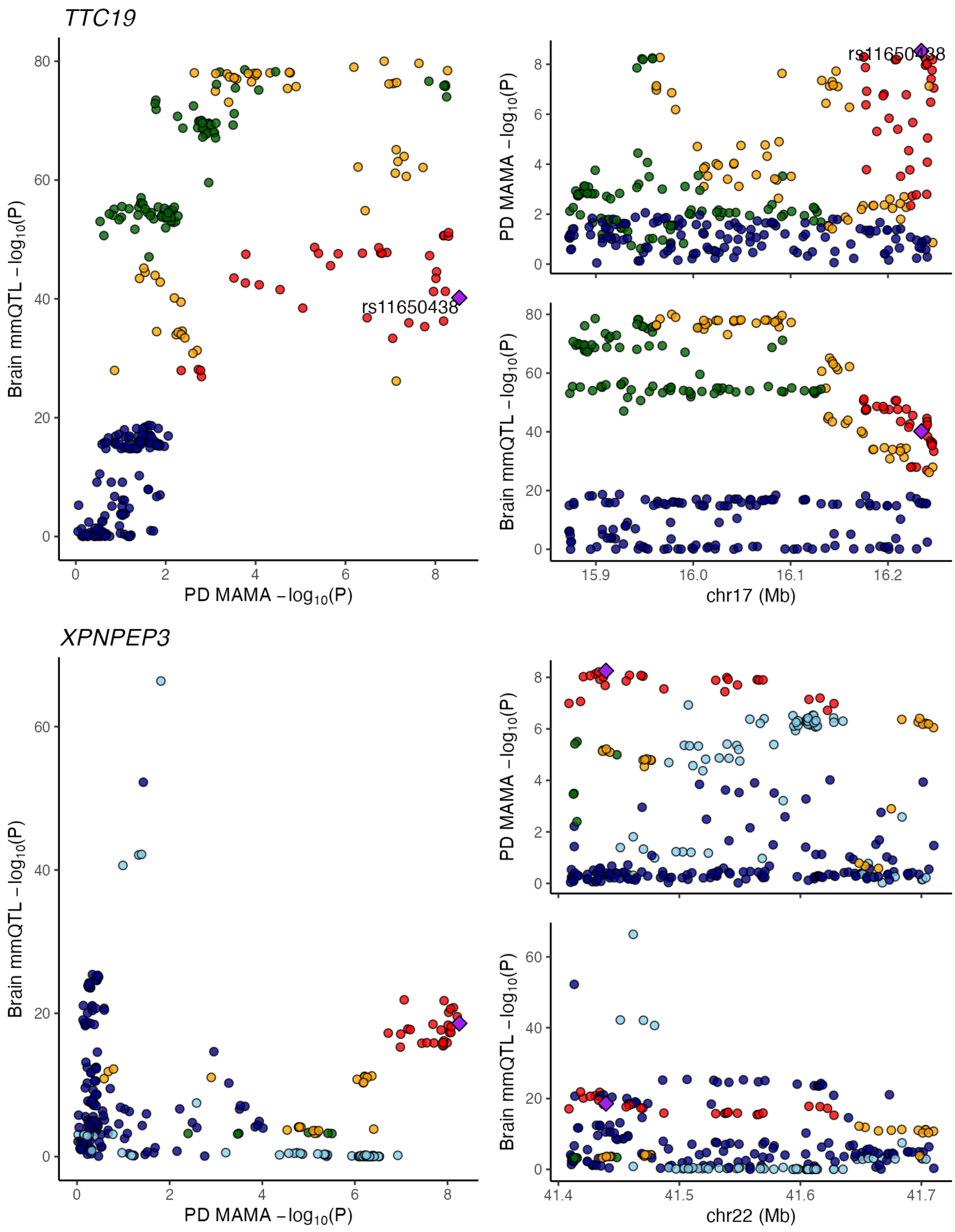

TTC19
Brain mmQTL −log10(P)
PD MAMA −log10(P)
rs11650438
chr17 (Mb)
XPNPEP3
chr22 (Mb)

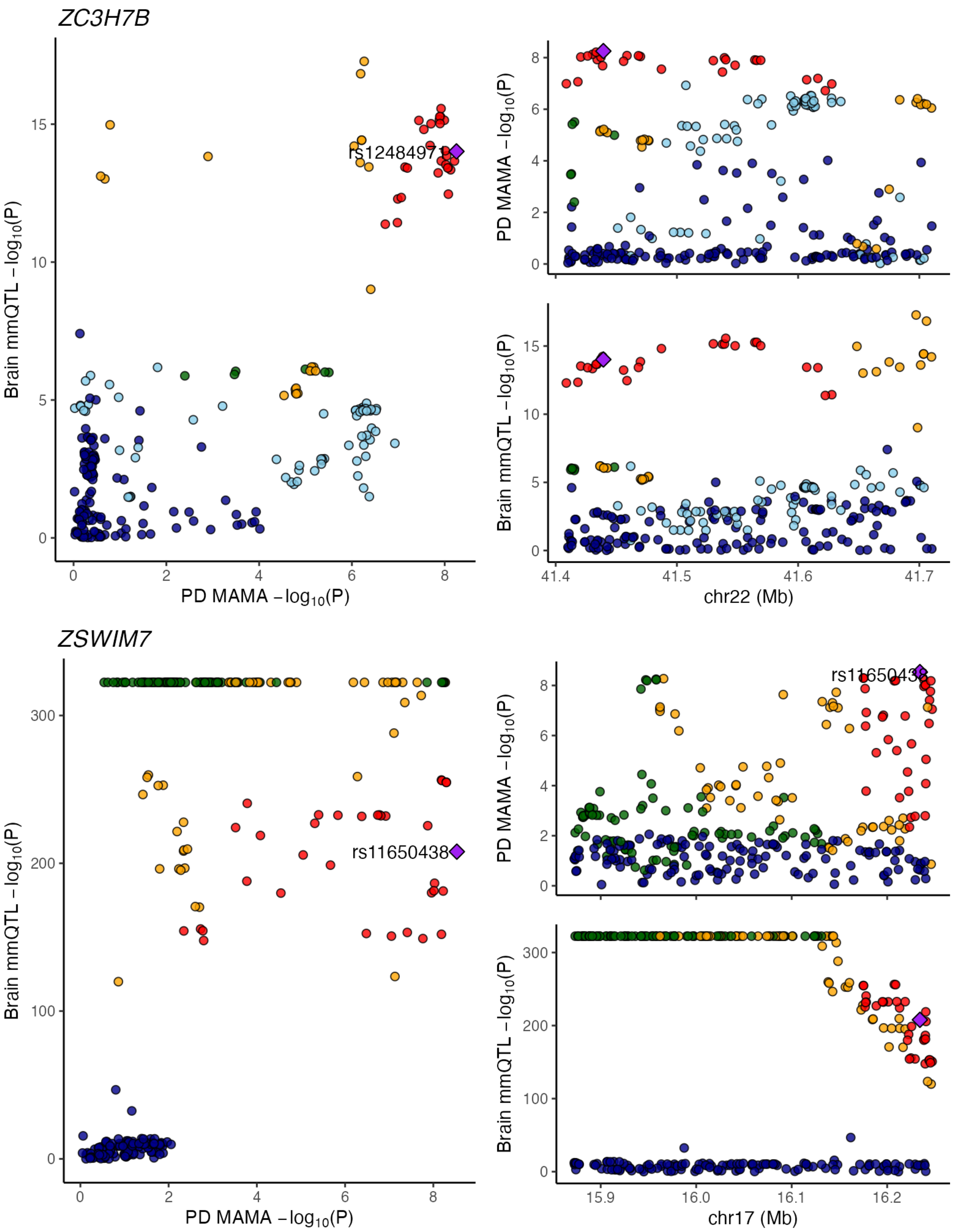
ZC3H7B
Brain mmQTL −log10(P)
PD MAMA −log10(P)
chr22 (Mb)
ZSWIM7
rs11650438
Brain mmQTL −log10(P)
PD MAMA −log10(P)
chr17 (Mb)

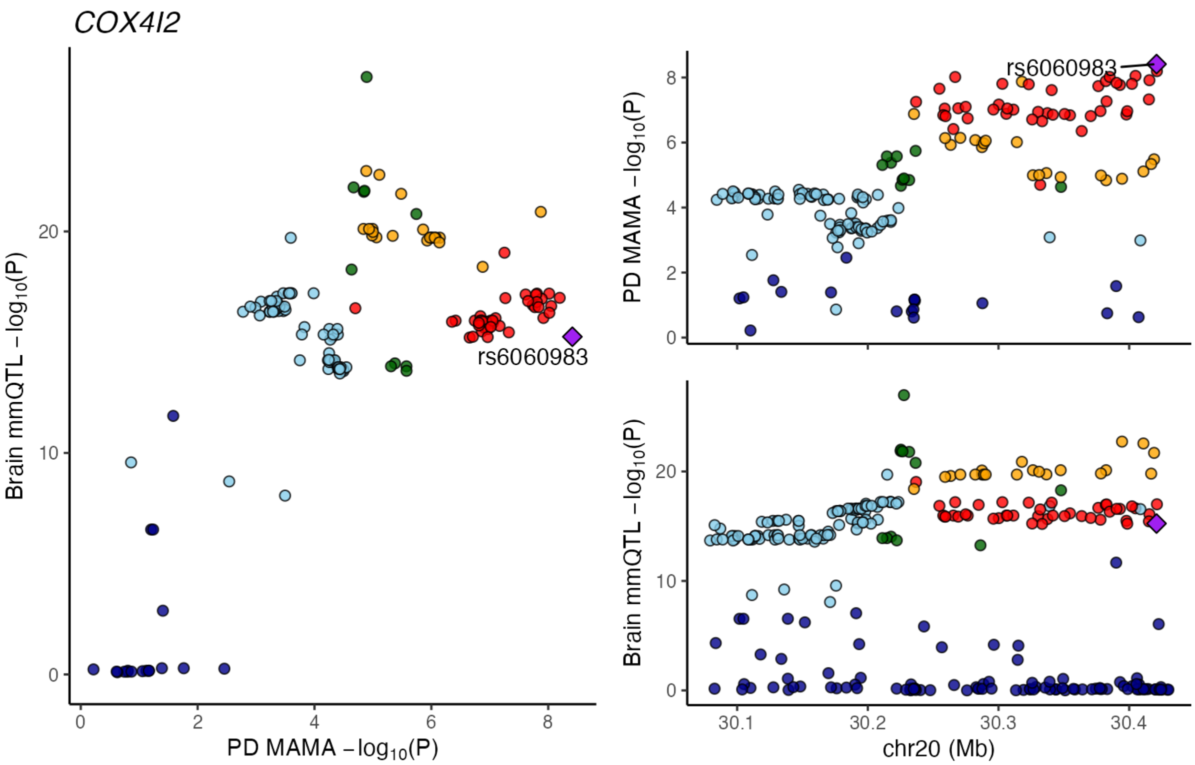

COX4I2
Brain mmQTL −log10(P)
PD MAMA −log10(P)
rs6060983
PD MAMA −log10(P)
rs6060983
Brain mmQTL −log10(P)
chr20 (Mb)
30.1
30.2
30.3
30.4

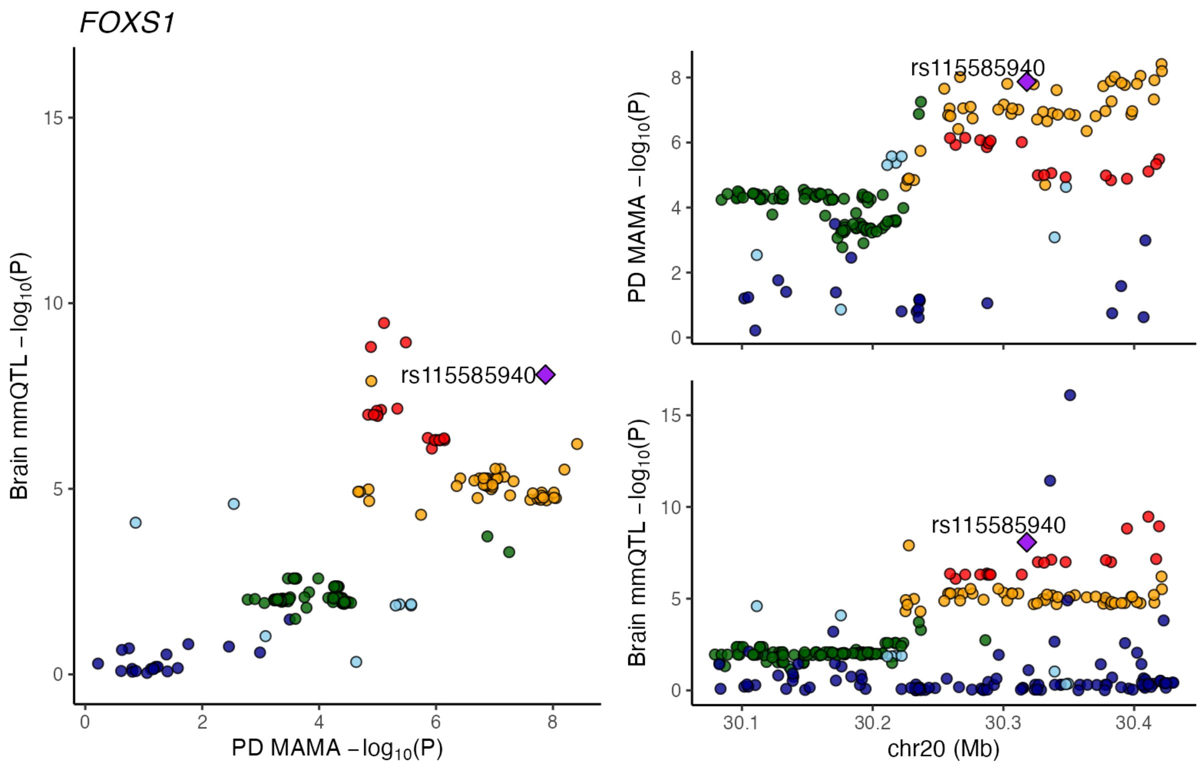

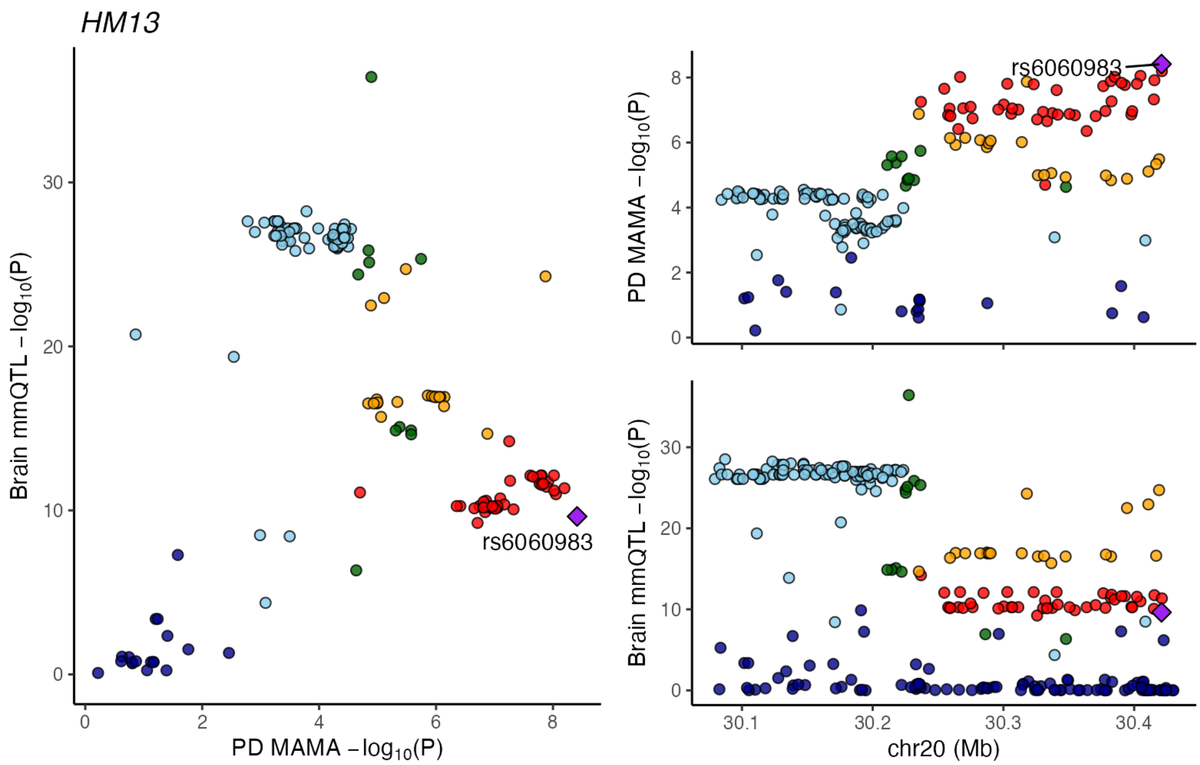

Supplementary Figure 8 - LocusCompare Plots of novel loci comparing PD MAMA association and brain eQTL results. Left is the LocusCompare plot between the PD MAMA GWAS results and eQTL results, where x-axis is the $-\log_{10}$ two-sided P value of the meta-analysis GWAS result and y-axis is the $-\log_{10}$ two-sided P value of the multi-ancestry brain eQTL. Right are two locuszoom plots, with the GWAS results on top and eQTL results on the bottom. The x-axis represents the basepair-position in hg19 while the y-axis represents the $-\log_{10}$ two-sided P value of the GWAS or eQTL results. Violet diamond denotes the lead SNP determined by the highest -log10 p-value. Linkage disequilibrium reference is based on the 1000 Genome dataset, EUR populations. Brain eQTL data is from the meta-analysis study by Zeng et. al 2022. Lead SNP was determined by summing the p-values of the two studies and selecting the lowest sum. All GWAS P values are from random-effects tests with the exception of *COX4I2*, *FOXS1*, and *HM13*, which are from MR-MEGA as the relevant locus were only identified in MR-MEGA. The plots only include eQTL order 1 to improve visibility.

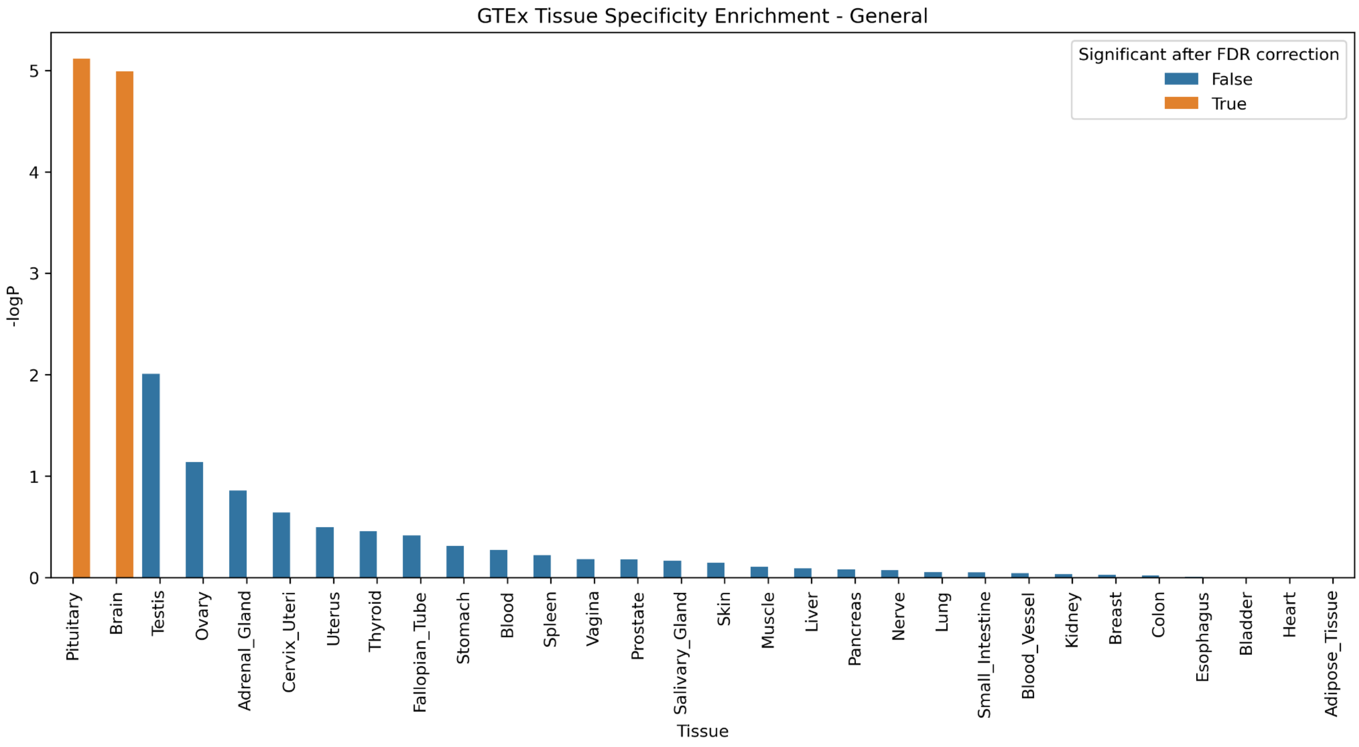

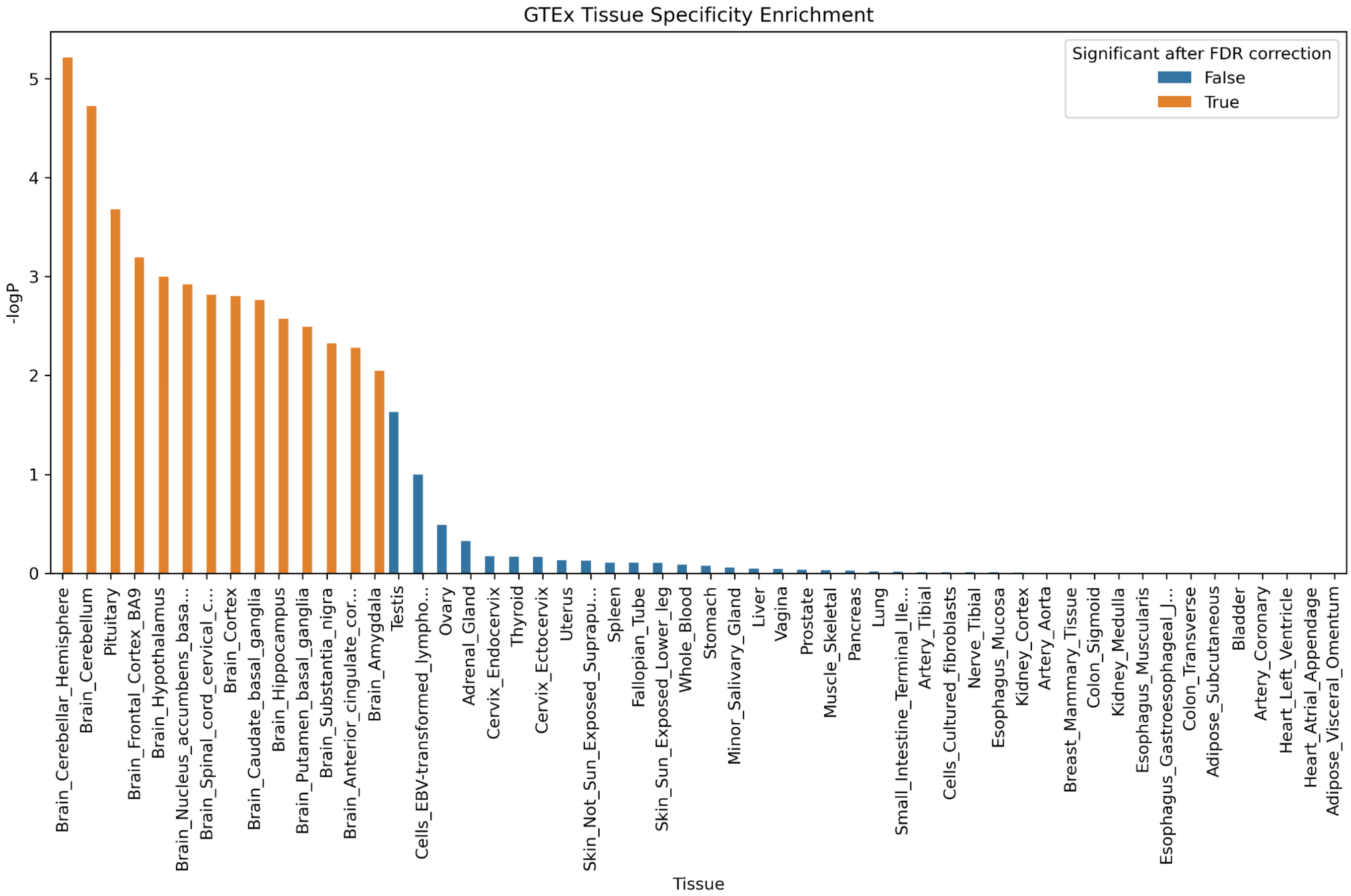

Supplementary Figure 9 - MAGMA GTEx tissue expression enrichment by tissue from FUMA. The x-axis shows the different GTEx tissues while the y-axis shows the two-sided p value of MAGMA gene-property analyses in $-\log_{10}P$ scale. P values were not corrected for multiple tests but tissues significant after false-discovery rate correction are highlighted as orange. Top is the 30 general tissue level. Bottom is the 53 detailed tissue level.

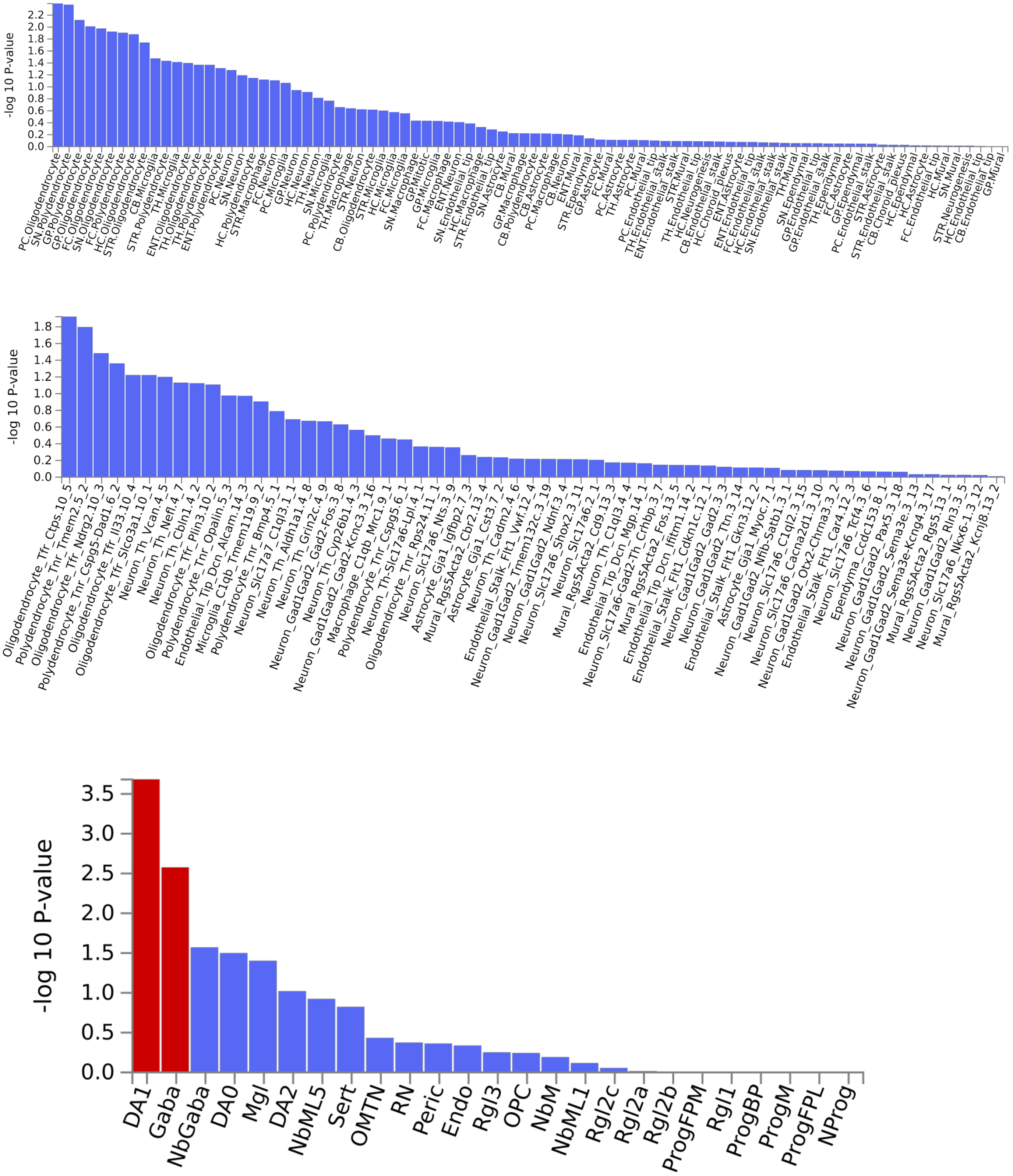

Supplementary Figure 10 - MAGMA expression enrichment from single cell RNAseq data. The x-axis shows the different cell types while the y-axis shows the -log_{10} two-tailed p-value for the MAGMA enrichment test. Top: murine cells from DropViz - all level 1 cell type class; center: murine substantia nigra cells from DropViz - level 2 cell type subcluster; Bottom: human midbrain cells (La Manno et al. 2016. GEO: GSE76381)

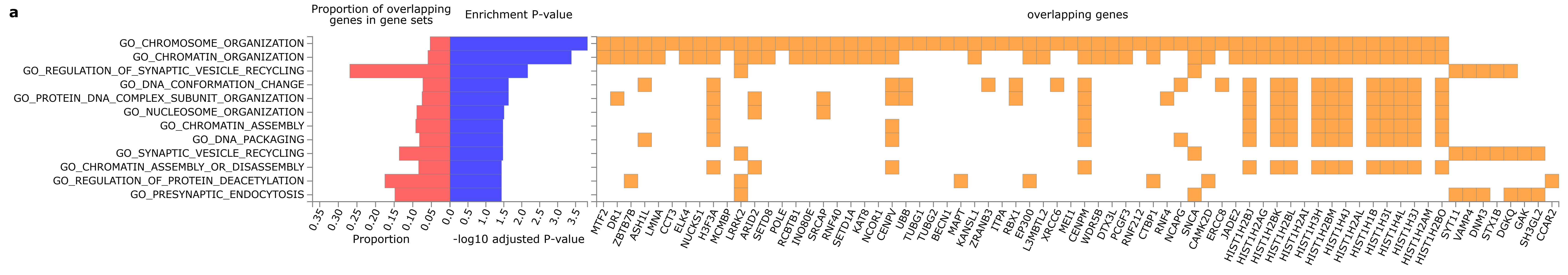
a
Proportion of overlapping genes in gene sets
Enrichment P-value
overlapping genes
GO_CHROMOSOME_ORGANIZATION
GO_CHROMATIN_ORGANIZATION
GO_REGULATION_OF_SYNAPTIC_VESICLE_RECYCLING
GO_DNA_CONFORMATION_CHANGE
GO_PROTEIN_DNA_COMPLEX_SUBUNIT_ORGANIZATION
GO_NUCLEOSOME_ORGANIZATION
GO_CHROMATIN_ASSEMBLY
GO_DNA_PACKAGING
GO_SYNAPTIC_VESICLE_RECYCLING
GO_CHROMATIN_ASSEMBLY_OR_DISASSEMBLY
GO_REGULATION_OF_PROTEIN_DEACETYLATION
GO_PRESYNAPTIC_ENDOCYTOSIS
0.35 0.30 0.25 0.20 0.15 0.10 0.05 0.0
Proportion
0.5 1.0 1.5 2.0 2.5 3.0 3.5
-log10 adjusted P-value
MTF2 DR1 ZBTB7B ASH1L LMNA CCT3 ELK4 NUCKS1 H3F3A MCMBP LRRK2 ARID2 SETD8 POLE RCBTB1 INO80E SRCAP RNF40 SETD1A KAT8 NCOR1 CENPV UBB TUBG1 TUBG2 BECN1 MAPT KANSL1 ZRANB3 ITPA RBX1 EP300 L3MBTL2 XRCC6 MEI1 CENPM WDR5B DTX3L PCGF3 RNF212 CTBP1 RNF4 NCAPG SNCA CAMK2D ERCC8 JADE2 HIST1H2BJ HIST1H2AG HIST1H2BK HIST1H2BL HIST1H2AI HIST1H3H HIST1H2BM HIST1H4J HIST1H2AL HIST1H1B HIST1H3I HIST1H4L HIST1H3J HIST1H2AM HIST1H2BO SYT11 VAMP4 DNM3 STX1B DGKQ GAK SH3GL2 CCAR2

b

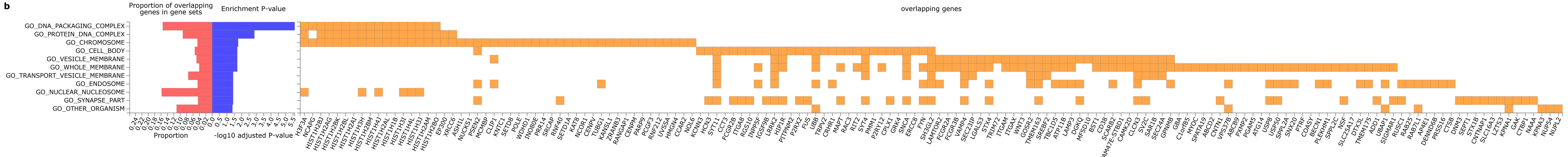

Proportion of overlapping genes in gene sets
Enrichment P-value
overlapping genes
GO_DNA_PACKAGING_COMPLEX
GO_PROTEIN_DNA_COMPLEX
GO_CHROMOSOME
GO_CELL_BODY
GO_VESICLE_MEMBRANE
GO_WHOLE_MEMBRANE
GO_TRANSPORT_VESICLE_MEMBRANE
GO_ENDOSOME
GO_NUCLEAR_NUCLEOSOME
GO_SYNAPSE_PART
GO_OTHER_ORGANISM
Proportion
-log10 adjusted P-value

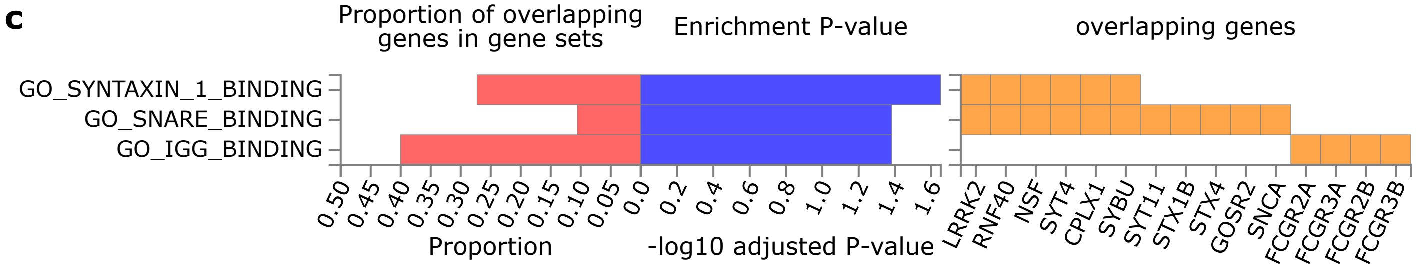
c
Proportion of overlapping genes in gene sets
Enrichment P-value
overlapping genes
GO_SYNTAXIN_1_BINDING
GO_SNARE_BINDING
GO_IGG_BINDING
0.50 0.45 0.40 0.35 0.30 0.25 0.20 0.15 0.10 0.05 0.0
0.2 0.4 0.6 0.8 1.0 1.2 1.4 1.6
Proportion
-log10 adjusted P-value
LRRK2 RNF40 NSF SYT4 CPLX1 SYBU SYT11 STX1B STX4 GOSR2 SNCA FCGR2A FCGR3A FCGR2B FCGR3B

d

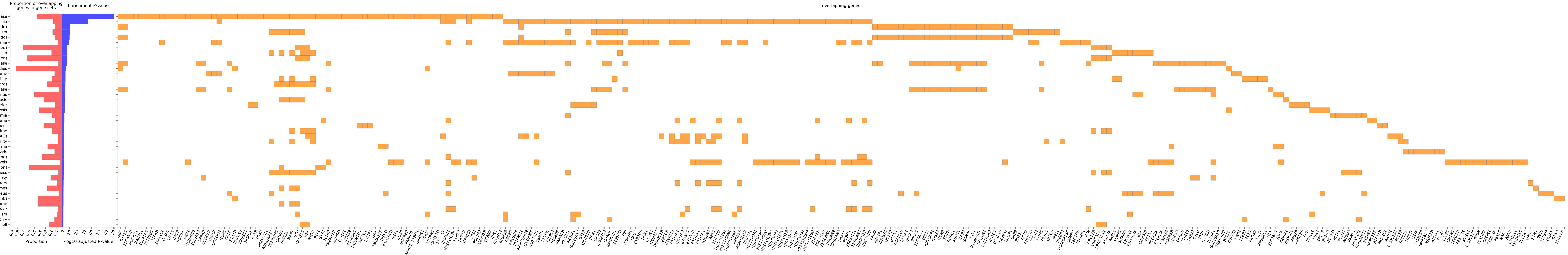
Proportion of overlapping genes in gene sets
Enrichment P-value
overlapping genes
Parkinson's disease
Autism spectrum disorder or schizophrenia
Body fat distribution (leg fat ratio)
Neuroticism
Body fat distribution (trunk fat ratio)
Schizophrenia
Handedness (Right-handed vs. non-right-handed)
General factor of neuroticism
Handedness (Left-handed vs. non-left-handed)
Inflammatory bowel disease
Dementia with Lewy bodies
Tourette syndrome
Mood instability
Alcohol use disorder (total score)
Crohn's disease
Polymyositis
Idiopathic pulmonary fibrosis
Bipolar I disorder
Immunoglobulin light chain (AL) amyloidosis
Craniofacial microsomia
Squamous cell lung carcinoma
Subjective response to lithium treatment
Reaction time
Intelligence (MTAG)
General cognitive ability
Limited cutaneous systemic scleroderma
Calcium levels
Sarcoidosis (Lofgren's syndrome vs non-Lofgren's syndrome)
Blood protein levels
Accelerometer-based physical activity measurement (average acceleration)
Male-pattern baldness
Leprosy
Lung cancer in ever smokers
Subcortical brain region volumes
Systemic lupus erythematosus
Response to metformin (IC50)
Intracranial volume
Lung cancer
Neuroticism
Feeling worry
Sense of smell
Proportion
-log10 adjusted P-value

Supplementary Figure 11 - FUMA GENE2FUNC analysis of FUMA SNP2GENE mapped genes. The plots show enrichment analysis in a. biological processes b. cellular components c. molecular function d. GWAS Catalog

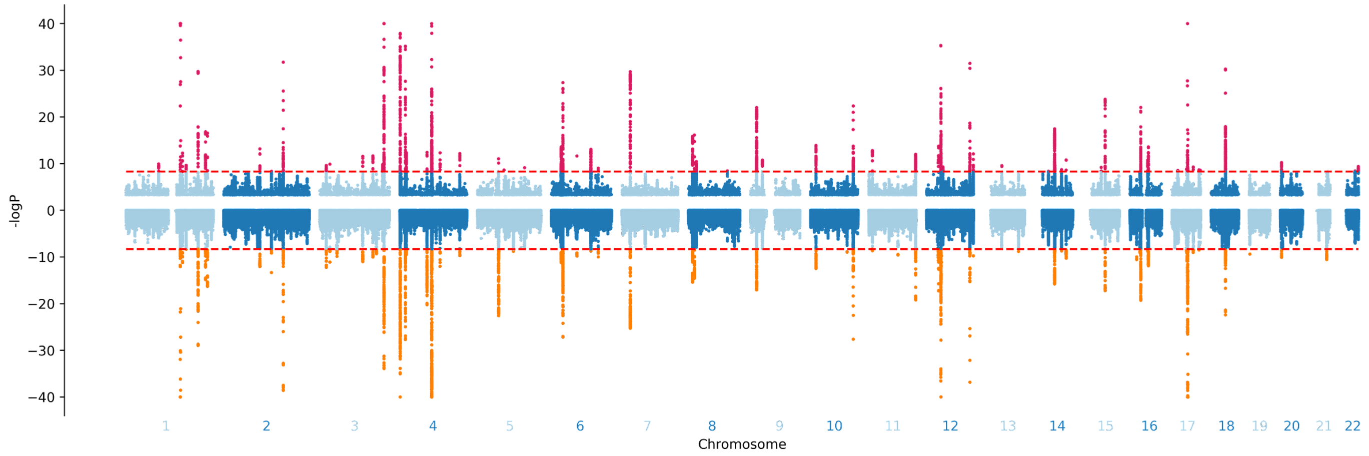

Supplementary Figure 12 - Miami plot of multi-ancestry random-effect meta-analysis (top) and Nalls et al. 2019 European single ancestry meta-analysis (bottom). The x-axis shows chromosome and base pair positions of each variant tested. The y-axis shows the two-sided P value in the -$\log_{10}$ scale, above 0 showing the -$\log_{10}$ value of the multi-ancestry random-effect meta-analysis while below 0 showing $\log_{10}$ value of the European-only meta-analysis. Significant variants in multi-ancestry and European-only analyses are highlighted in red and orange respectively.
