# Supplementary material for: Multi-ancestry genome-wide association meta-analysis of Parkinson’s disease
Source: Nat Genet. 2023 Dec 28;56(1):27–36. doi: 10.1038/s41588-023-01584-8 (PMC10786718; doi:10.1038/s41588-023-01584-8)
Supplement: Supplementary file 2 — Reporting Summary [file 41588_2023_1584_MOESM2_ESM.pdf]

nature portfolio

Corresponding author(s): Jonggeol Jeffrey Kim, Cornelis Blauwendraat, Mike Nalls, Jia Nee Foo, Ignacio Mata

Last updated by author(s): Aug 3, 2023



# Reporting Summary

Nature Portfolio wishes to improve the reproducibility of the work that we publish. This form provides structure for consistency and transparency in reporting. For further information on Nature Portfolio policies, see our Editorial Policies and the Editorial Policy Checklist.

## Statistics

For all statistical analyses, confirm that the following items are present in the figure legend, table legend, main text, or Methods section.

| n/a | Confirmed | |
|---|---|---|
| ☐ | ☒ | The exact sample size (*n*) for each experimental group/condition, given as a discrete number and unit of measurement |
| ☐ | ☒ | A statement on whether measurements were taken from distinct samples or whether the same sample was measured repeatedly |
| ☐ | ☒ | The statistical test(s) used AND whether they are one- or two-sided<br>*Only common tests should be described solely by name; describe more complex techniques in the Methods section.* |
| ☐ | ☒ | A description of all covariates tested |
| ☐ | ☒ | A description of any assumptions or corrections, such as tests of normality and adjustment for multiple comparisons |
| ☐ | ☒ | A full description of the statistical parameters including central tendency (e.g. means) or other basic estimates (e.g. regression coefficient) AND variation (e.g. standard deviation) or associated estimates of uncertainty (e.g. confidence intervals) |
| ☐ | ☒ | For null hypothesis testing, the test statistic (e.g. *F*, *t*, *r*) with confidence intervals, effect sizes, degrees of freedom and *P* value noted<br>*Give P values as exact values whenever suitable.* |
| ☒ | ☐ | For Bayesian analysis, information on the choice of priors and Markov chain Monte Carlo settings |
| ☒ | ☐ | For hierarchical and complex designs, identification of the appropriate level for tests and full reporting of outcomes |
| ☐ | ☒ | Estimates of effect sizes (e.g. Cohen's *d*, Pearson's *r*), indicating how they were calculated |

*Our web collection on statistics for biologists contains articles on many of the points above.*

## Software and code

Policy information about availability of computer code

**Data collection**

No software was used.

**Data analysis**

Data harmonization was done on Python 3.7 and CrossMap. Meta-analyses were done in PLINK 1.9 and Meta-Regression of Multi-Ethnic Genetic Association (MR-MEGA). Putative burden analysis was done using population-specific/shared causal variants (PESCA). Gene-ontology and tissue enrichment tests were done in Functional Mapping and Annotation (FUMA) and Multi-marker Analysis of GenoMic Annotation (MAGMA). Variant level annotation was done using ANNOVAR through FUMA. Plots and other miscellaneous analyses were done in Python or R. Analysis scripts are available on Github: https://github.com/GP2code/GP2-Multiancestry-metaGWAS and deposited deposited on Zenodo under doi:10.5281/zenodo.8045547.

Programs and their respective versions:
PLINK v1.9
MR-MEGA v0.2
FUMA v1.3.8
MAGMA v1.08
ANNOVAR-last updated on Dec 5 2016
PESCA v0.3
Python 3.7
R 4.2.0

R packages used:
LDlinkR v1.1.217

LocusCompareR

Python packages:
gwaslab v3.3.11
seaborn v0.11.2
matplotlib v3.5.1

For manuscripts utilizing custom algorithms or software that are central to the research but not yet described in published literature, software must be made available to editors and reviewers. We strongly encourage code deposition in a community repository (e.g. GitHub). See the Nature Portfolio guidelines for submitting code & software for further information.

## Data

Policy information about availability of data

All manuscripts must include a data availability statement. This statement should provide the following information, where applicable:

- Accession codes, unique identifiers, or web links for publicly available datasets
- A description of any restrictions on data availability
- For clinical datasets or third party data, please ensure that the statement adheres to our policy

GWAS summary statistics for Foo et al. 2020 and Loesch et al. 2020 are available upon request to the respective authors. The UKBB genotype and phenotype data are available through the UKBB web portal https://www.ukbiobank.ac.uk/. FinnGen summary statistics are available through the FinnGen website https://www.finngen.fi/. GWAS summary statistics for 23andMe datasets (post-Chang and data included in Chang et al. 2017 and Nalls et al. 2014) will be made available through 23andMe to qualified researchers under an agreement with 23andMe that protects the privacy of the 23andMe participants. Please visit research.23andme.com/collaborate/#publication for more information and to apply to access the data. An immediately accessible version of the multi-ancestry summary statistics is available on the Neurodegenerative Disease knowledge Portal (https://ndkp.hugeamp.org/) excluding Nalls et al. 2014, 23andMe post-Chang et al. 2017 and Web-Based Study of Parkinson's Disease (PDWBS) but including all analyzed SNPs. Same summary statistics are also available at AMP-PD (https://amp-pd.org/) under GP2 Tier 1 access and GWAS Catalog under accession code GCST90275127 (http://ftp.ebi.ac.uk/pub/databases/gwas/summary_statistics/GCST90275001-GCST90276000/GCST90275127/). After applying with 23andMe, the full summary statistics including all analyzed SNPs and samples in this GWAS meta-analysis will be accessible to the approved researcher(s). MSigDb is available at http://software.broadinstitute.org/gsea/msigdb/. GTEx is available at https://gtexportal.org/home/. Multi-ancestry brain eQTL data from Zeng et al. 2020 are available at https://hoffmg01.hpc.mssm.edu/brema/. eQTL/mQTL/caQTL data used for SMR outside of MetaBrain and eQTLGen are available at https://yanglab.westlake.edu.cn/software/smr/#DataResource. MetaBrain eQTL data are available at https://www.metabrain.nl/. eQTLGen data are available at https://www.eqtlgen.org/. pQTL data from Wingo et al. are available upon request to the respective author. UK Biobank-Nightingale metabolomic data used for SMR are available at https://gwas.mrcieu.ac.uk/.

## Research involving human participants, their data, or biological material

Policy information about studies with human participants or human data. See also policy information about sex, gender (identity/presentation), and sexual orientation and race, ethnicity and racism.

| | |
|---|---|
| Reporting on sex and gender | All data used in this analysis used biological sex as a covariate in their respective studies. |
| Reporting on race, ethnicity, or other socially relevant groupings | We used data from four ancestrally-distinct populations: European, East Asian, African, and Latin American. Ancestry was defined as continent of ancestral origin defined by an individual's genotype information. We did not use any socially constructed variable such as race and ethnicity as a proxy for ancestry. As we used summary-level data as-is, we did not make specific ancestry determinations as the respective studies made the determinations using their individual-level data. For 23andMe data, ancestry was determined using a genotype-based pipeline consisting of a support vector machine and a hidden Markov model, followed by a logistic classifier to differentiate Latinos from African Americans. |
| Population characteristics | Data included participants with Parkinson's disease and control participants. As we used data as-is, we do not describe any additional population characteristics and they can be found in their respective manuscripts |
| Recruitment | We did not recruit participants for this study. However, data from 23andMe participants were collected through self-report which may influence the results via self-report bias. |
| Ethics oversight | All self-reported PD cases and controls from 23andMe provided informed consent and participated in the research online, under a protocol approved by the external AAHRPP-accredited IRB, Ethical & Independent Review Services (E&I Review). Participants were included in the analysis on the basis of consent status as checked at the time data analyses were initiated. The name of the IRB at the time of the approval was Ethical & Independent Review Services. Ethical & Independent Review Services was recently acquired, and its new name as of July 2022 is Salus IRB (https://www.versiticlinicaltrials.org/salusirb). |

Note that full information on the approval of the study protocol must also be provided in the manuscript.

# Field-specific reporting

Please select the one below that is the best fit for your research. If you are not sure, read the appropriate sections before making your selection.

☒ Life sciences ☐ Behavioural & social sciences ☐ Ecological, evolutionary & environmental sciences

For a reference copy of the document with all sections, see nature.com/documents/nr-reporting-summary-flat.pdf

# Life sciences study design

All studies must disclose on these points even when the disclosure is negative.

| | |
|---|---|
| Sample size | We collected datasets from as many datasets as available. As a meta-analysis of 7 different studies/analyses, this work is the largest Parkinson's disease Genome-Wide meta-analysis to-date with 49049 cases, 18618 proxy-cases, and 2458063 controls. |
| Data exclusions | For 23andMe datasets, only data that met the quality control criteria and were unrelated were included in the analysis. For inclusion, samples needed a minimal call rate of 98.5%. Only data from unrelated participants were used to minimize bias from relatedness. We used data from other studies as-is, but each respective studies performed their own quality-control procedures, including removing related participants and low genotype quality samples. |
| Replication | We used a single joint meta-analysis study design to maximize statistical power. No replication samples were available as all available datasets were used in the meta-analysis. |
| Randomization | The experimental groups were divided into Parkinson's disease cases and controls. All data used were adjusted for age, sex, population structure-principal components to account for population stratification. |
| Blinding | Blinding is not relevant to study as this is a meta-analysis of observational genetic studies and not a randomized experiment. |

# Reporting for specific materials, systems and methods

We require information from authors about some types of materials, experimental systems and methods used in many studies. Here, indicate whether each material, system or method listed is relevant to your study. If you are not sure if a list item applies to your research, read the appropriate section before selecting a response.

## Materials & experimental systems

| n/a | Involved in the study | |
|---|---|---|
| ☒ | ☐ | Antibodies |
| ☒ | ☐ | Eukaryotic cell lines |
| ☒ | ☐ | Palaeontology and archaeology |
| ☒ | ☐ | Animals and other organisms |
| ☒ | ☐ | Clinical data |
| ☒ | ☐ | Dual use research of concern |
| ☒ | ☐ | Plants |

## Methods

| n/a | Involved in the study | |
|---|---|---|
| ☒ | ☐ | ChIP-seq |
| ☒ | ☐ | Flow cytometry |
| ☒ | ☐ | MRI-based neuroimaging |
