# Supplementary figures and images for: Multi-ancestry genome-wide association meta-analysis of Parkinson’s disease (part 2 of 2)
Source: Nat Genet. 2023 Dec 28;56(1):27–36. doi: 10.1038/s41588-023-01584-8 (PMC10786718; doi:10.1038/s41588-023-01584-8)

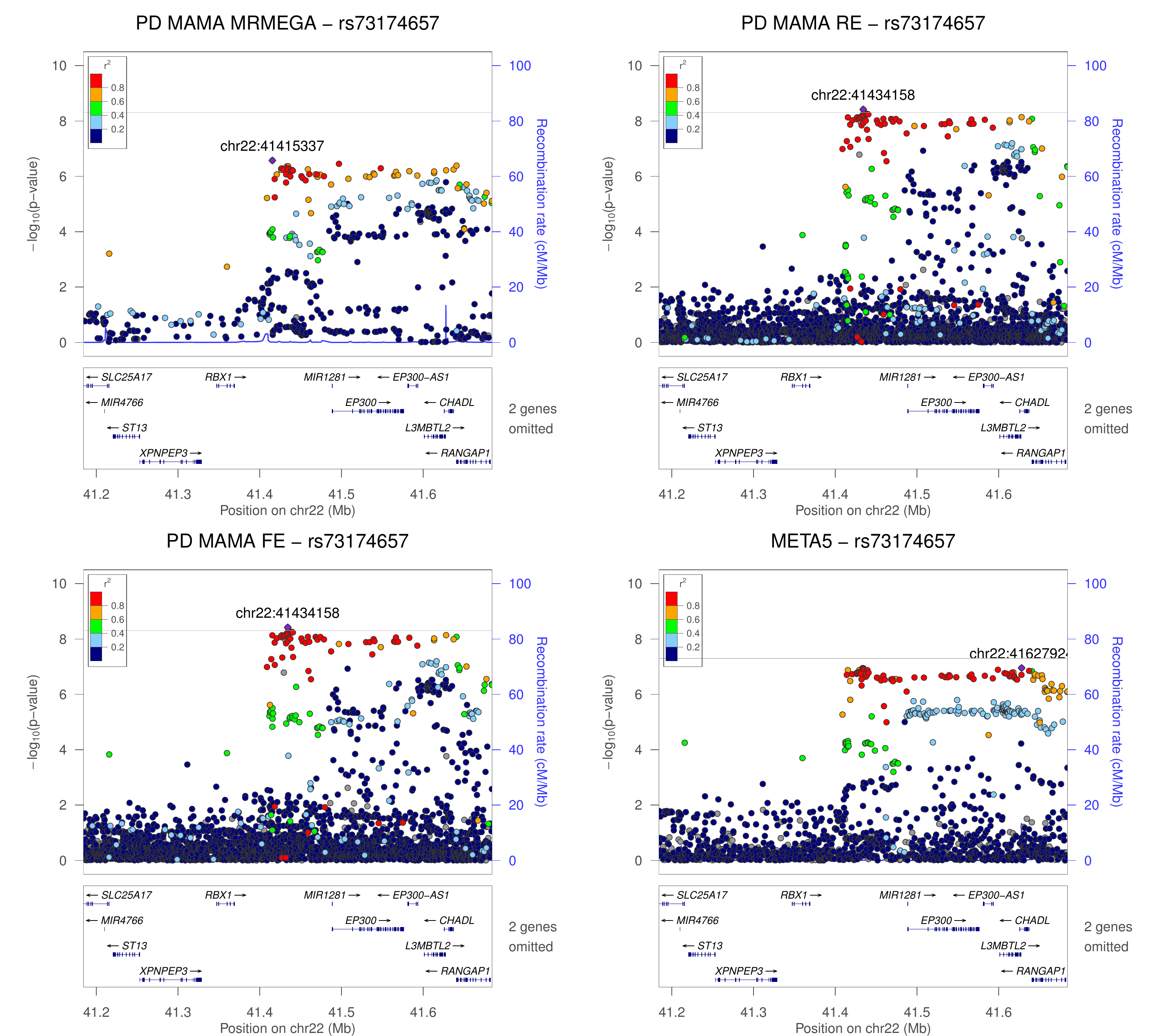

Supplement: Supplementary file 5 — This includes LocusZoom plots of all known European loci as well as novel loci. Each file contains four LocusZoom plots: PD MAMA MR-MEGA/RE/FE/ (MR-MEGA/random-effect/fixed-effect) and META5 (European-only meta-analysis from Nalls et al. 1). [file 41588_2023_1584_MOESM5_ESM.zip › LocusZoom plots of nominated novel loci/chr22_41184158-41684158.png]

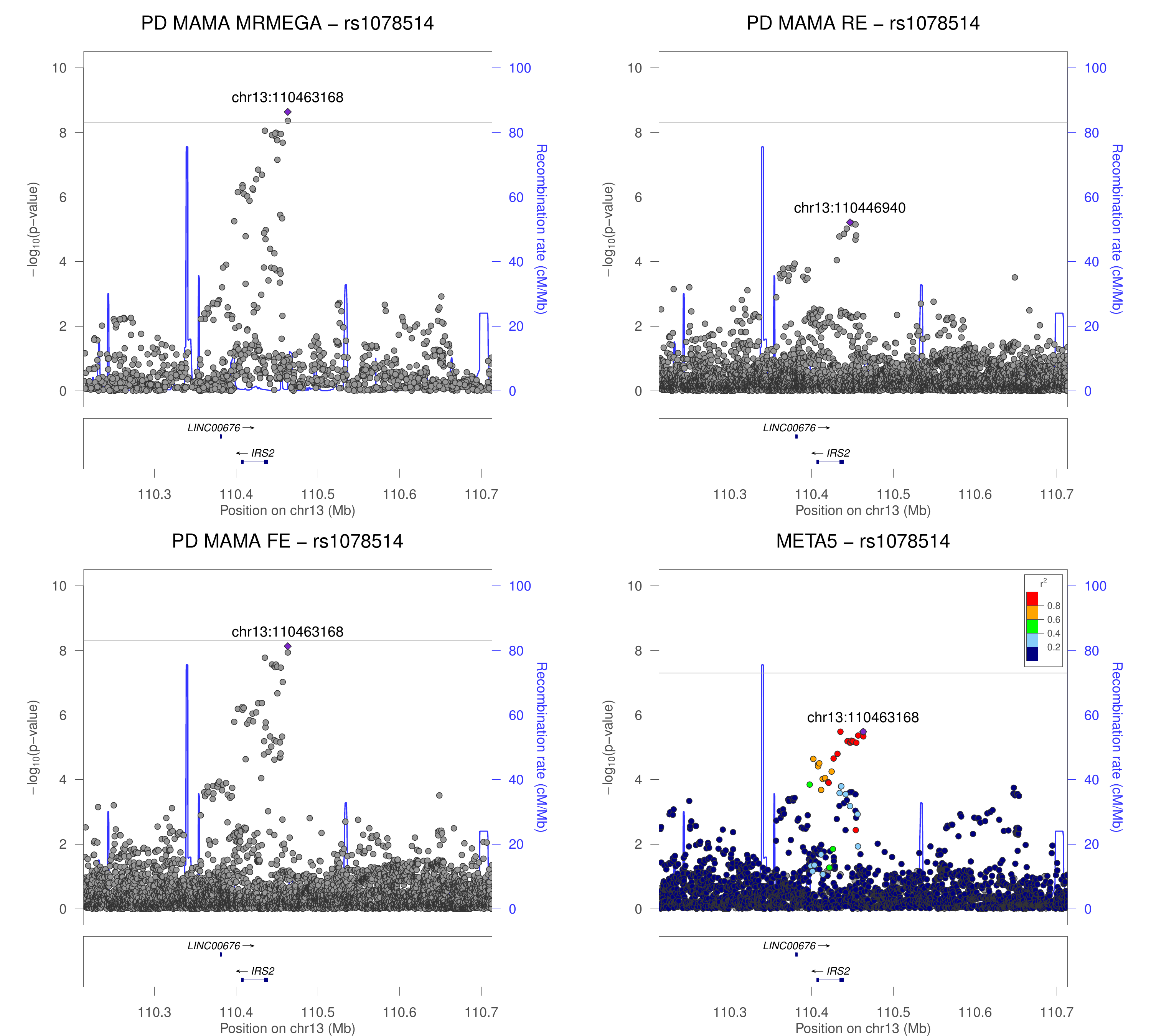

Supplement: Supplementary file 5 — This includes LocusZoom plots of all known European loci as well as novel loci. Each file contains four LocusZoom plots: PD MAMA MR-MEGA/RE/FE/ (MR-MEGA/random-effect/fixed-effect) and META5 (European-only meta-analysis from Nalls et al. 1). [file 41588_2023_1584_MOESM5_ESM.zip › LocusZoom plots of nominated novel loci/chr13_110213168-110713168.png]
