# Supplementary figures and images for: Multi-ancestry genome-wide association meta-analysis of Parkinson’s disease (part 1 of 2)
Source: Nat Genet. 2023 Dec 28;56(1):27–36. doi: 10.1038/s41588-023-01584-8 (PMC10786718; doi:10.1038/s41588-023-01584-8)

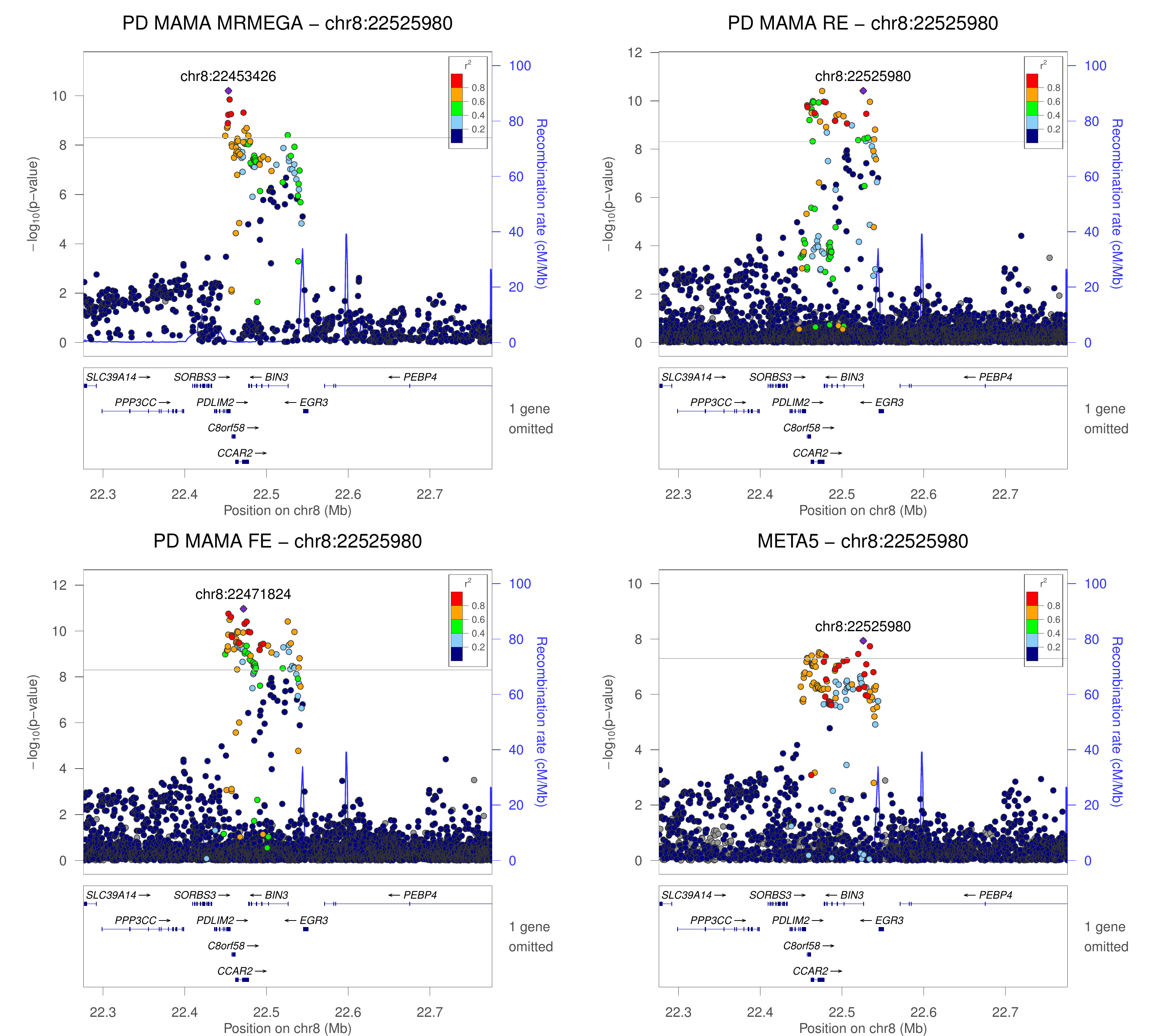

Supplement: Supplementary file 5 — This includes LocusZoom plots of all known European loci as well as novel loci. Each file contains four LocusZoom plots: PD MAMA MR-MEGA/RE/FE/ (MR-MEGA/random-effect/fixed-effect) and META5 (European-only meta-analysis from Nalls et al. 1). [file 41588_2023_1584_MOESM5_ESM.zip › LocusZoom plots of known EUR risk variants/chr8_22275980-22775980.png]

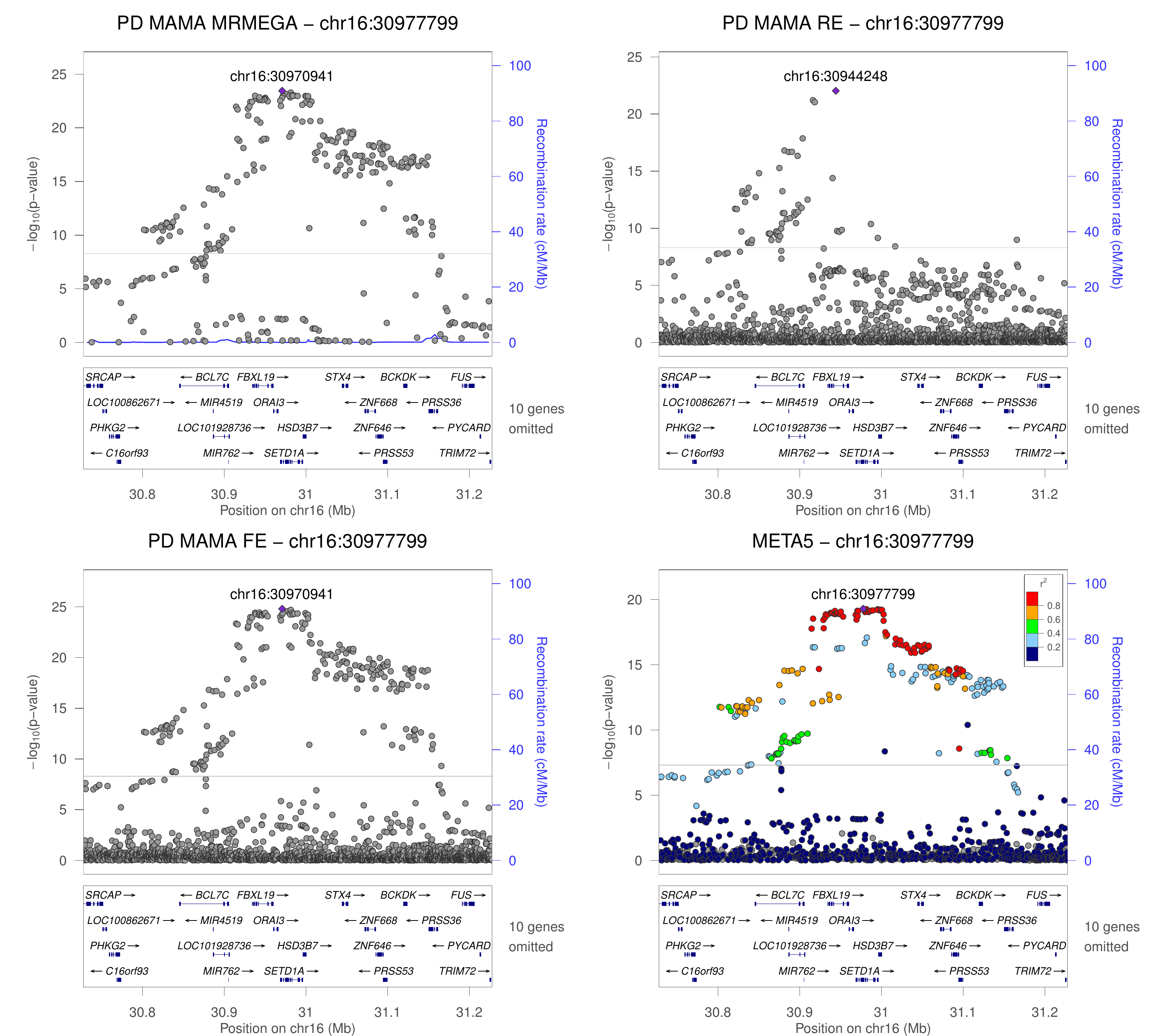

Supplement: Supplementary file 5 — This includes LocusZoom plots of all known European loci as well as novel loci. Each file contains four LocusZoom plots: PD MAMA MR-MEGA/RE/FE/ (MR-MEGA/random-effect/fixed-effect) and META5 (European-only meta-analysis from Nalls et al. 1). [file 41588_2023_1584_MOESM5_ESM.zip › LocusZoom plots of known EUR risk variants/chr16_30727799-31227799.png]

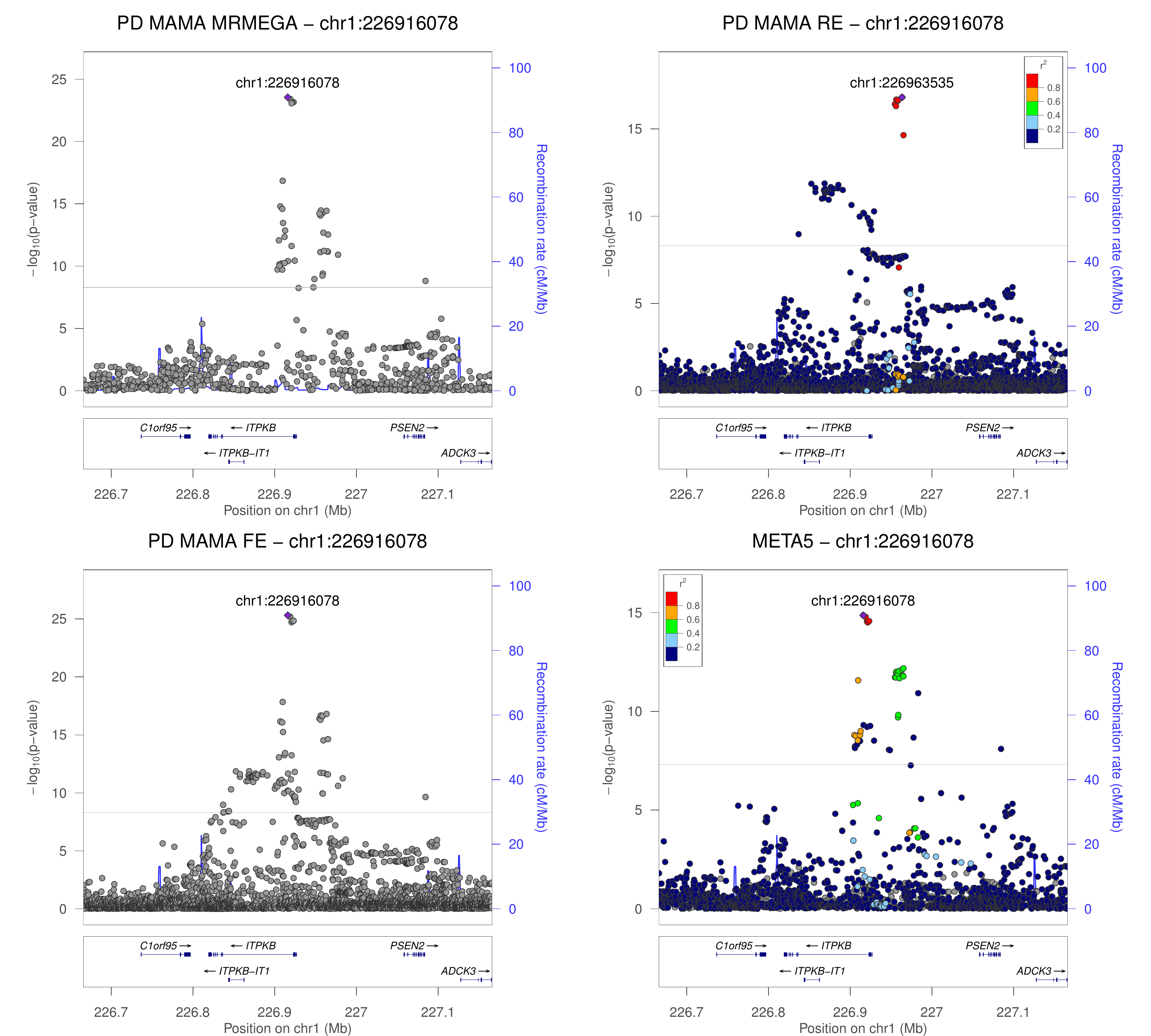

Supplement: Supplementary file 5 — This includes LocusZoom plots of all known European loci as well as novel loci. Each file contains four LocusZoom plots: PD MAMA MR-MEGA/RE/FE/ (MR-MEGA/random-effect/fixed-effect) and META5 (European-only meta-analysis from Nalls et al. 1). [file 41588_2023_1584_MOESM5_ESM.zip › LocusZoom plots of known EUR risk variants/chr1_226666078-227166078.png]

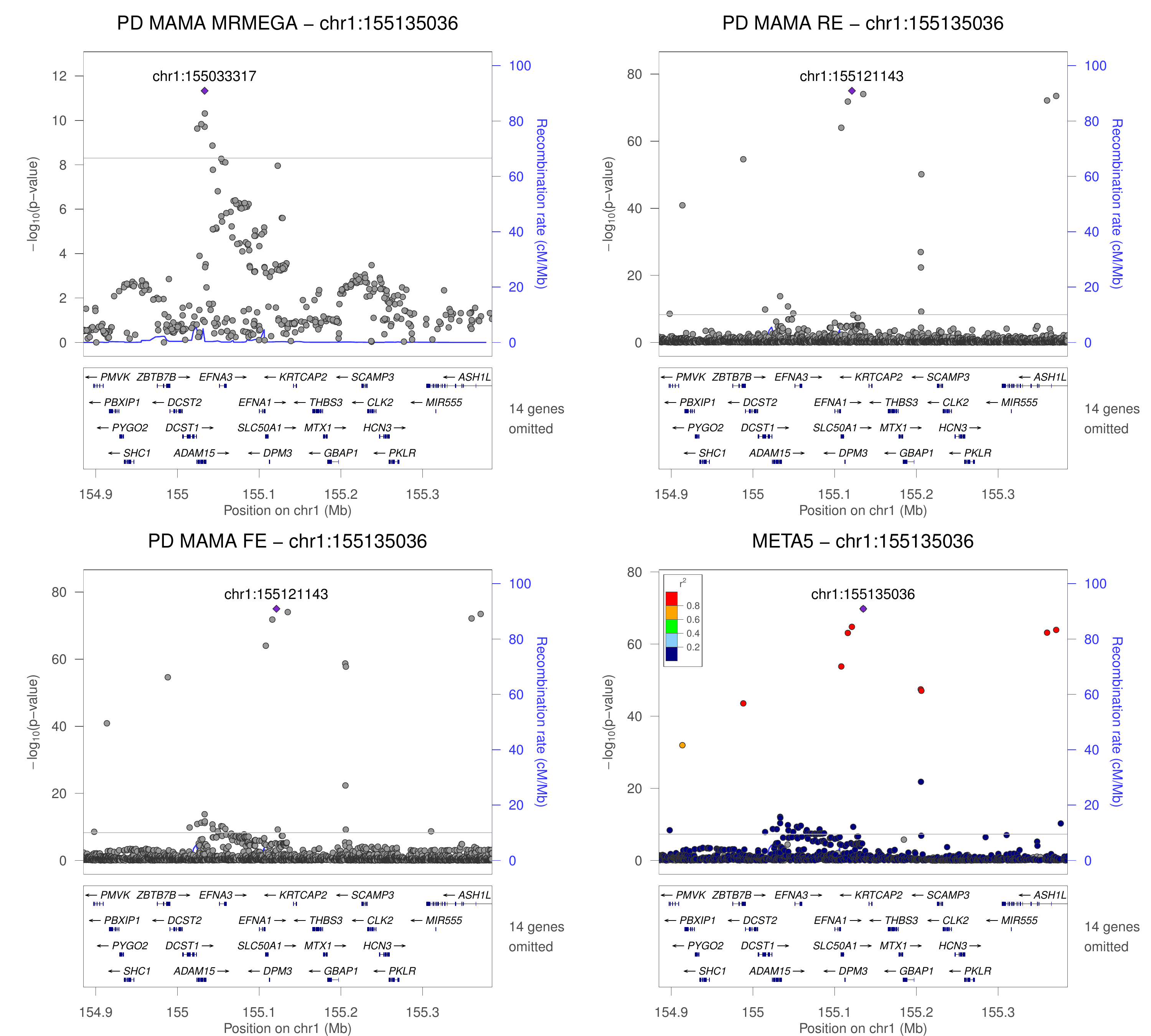

Supplement: Supplementary file 5 — This includes LocusZoom plots of all known European loci as well as novel loci. Each file contains four LocusZoom plots: PD MAMA MR-MEGA/RE/FE/ (MR-MEGA/random-effect/fixed-effect) and META5 (European-only meta-analysis from Nalls et al. 1). [file 41588_2023_1584_MOESM5_ESM.zip › LocusZoom plots of known EUR risk variants/chr1_154885036-155385036.png]

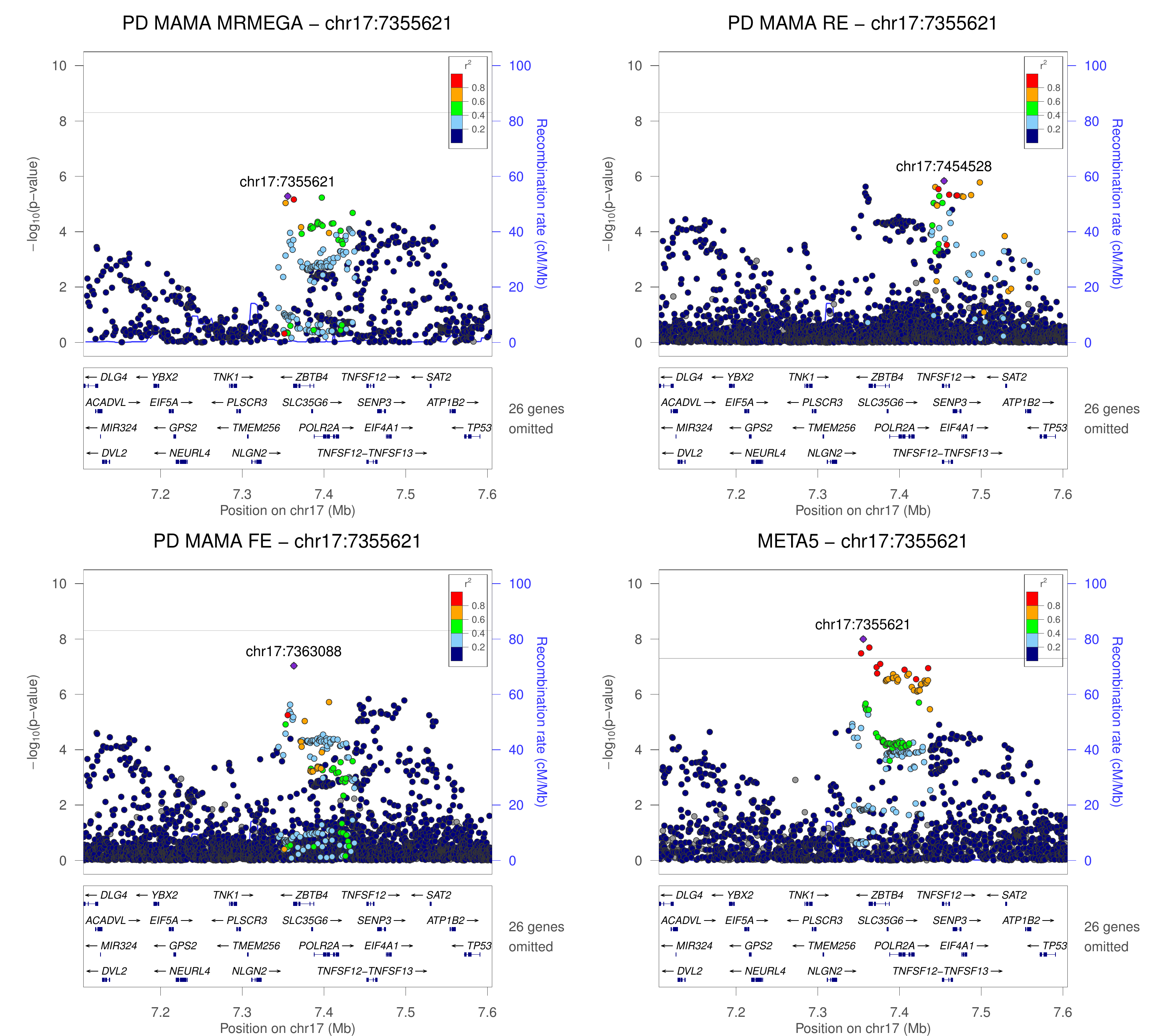

Supplement: Supplementary file 5 — This includes LocusZoom plots of all known European loci as well as novel loci. Each file contains four LocusZoom plots: PD MAMA MR-MEGA/RE/FE/ (MR-MEGA/random-effect/fixed-effect) and META5 (European-only meta-analysis from Nalls et al. 1). [file 41588_2023_1584_MOESM5_ESM.zip › LocusZoom plots of known EUR risk variants/chr17_7105621-7605621.png]

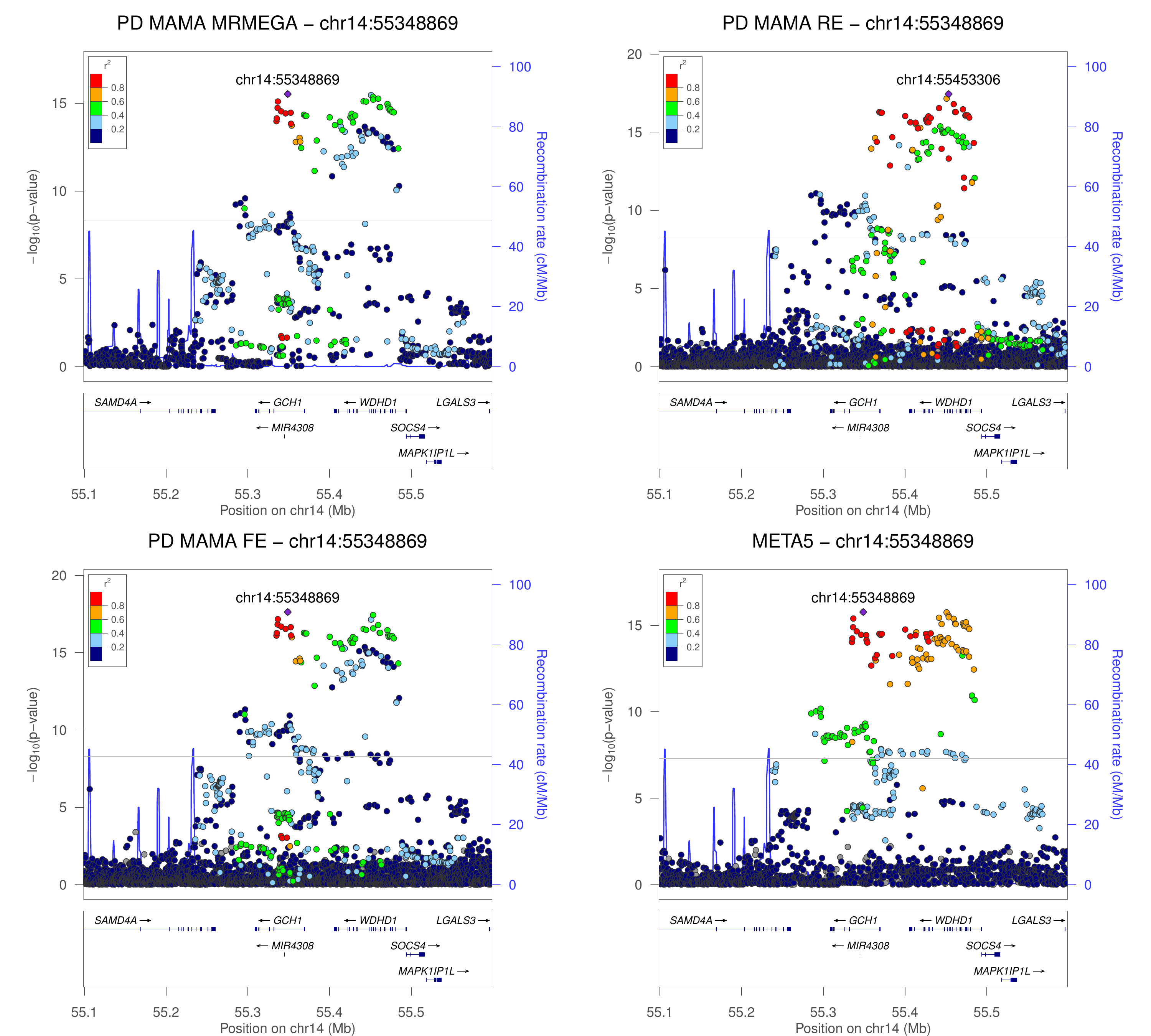

Supplement: Supplementary file 5 — This includes LocusZoom plots of all known European loci as well as novel loci. Each file contains four LocusZoom plots: PD MAMA MR-MEGA/RE/FE/ (MR-MEGA/random-effect/fixed-effect) and META5 (European-only meta-analysis from Nalls et al. 1). [file 41588_2023_1584_MOESM5_ESM.zip › LocusZoom plots of known EUR risk variants/chr14_55098869-55598869.png]

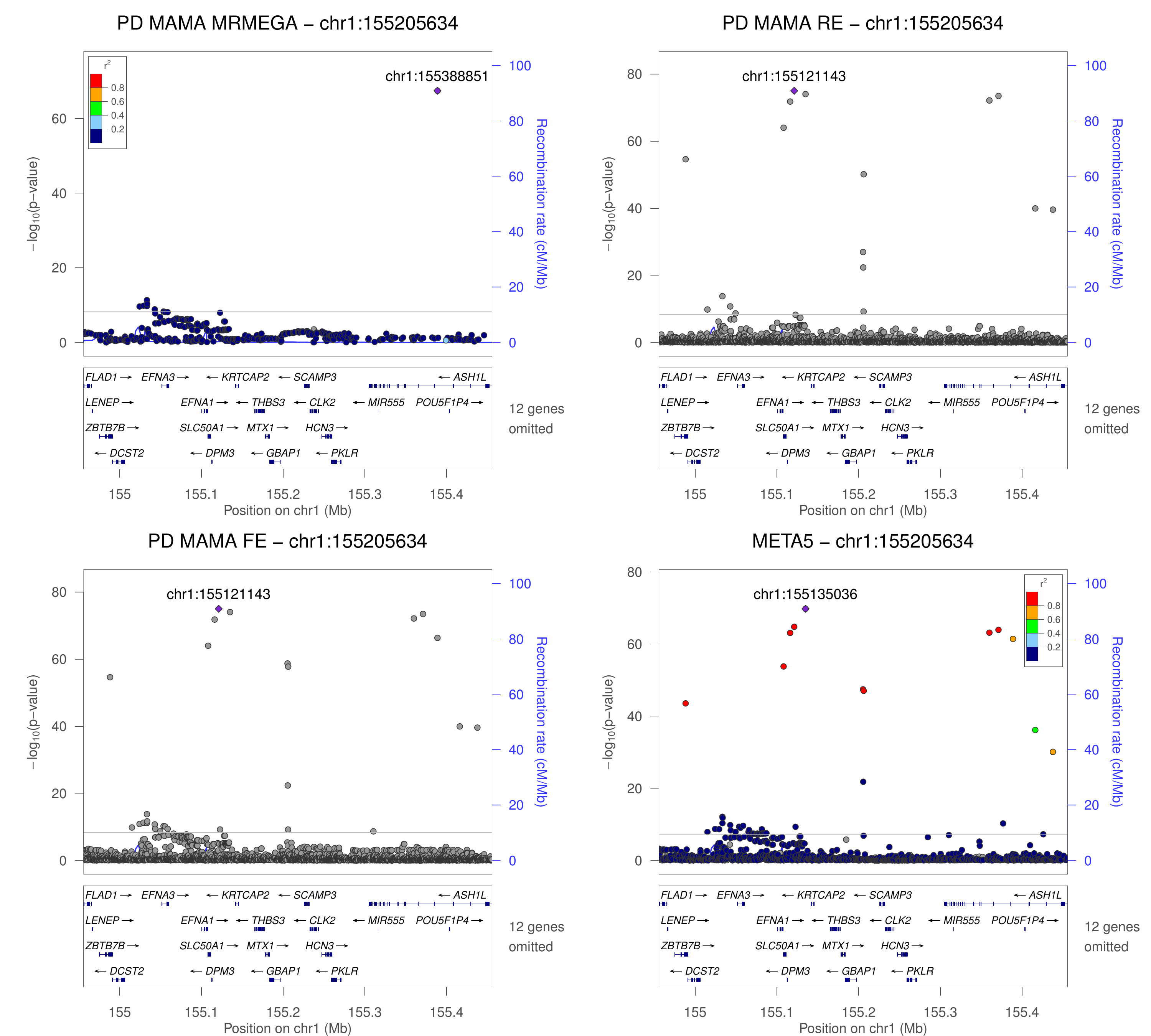

Supplement: Supplementary file 5 — This includes LocusZoom plots of all known European loci as well as novel loci. Each file contains four LocusZoom plots: PD MAMA MR-MEGA/RE/FE/ (MR-MEGA/random-effect/fixed-effect) and META5 (European-only meta-analysis from Nalls et al. 1). [file 41588_2023_1584_MOESM5_ESM.zip › LocusZoom plots of known EUR risk variants/chr1_154955634-155455634.png]

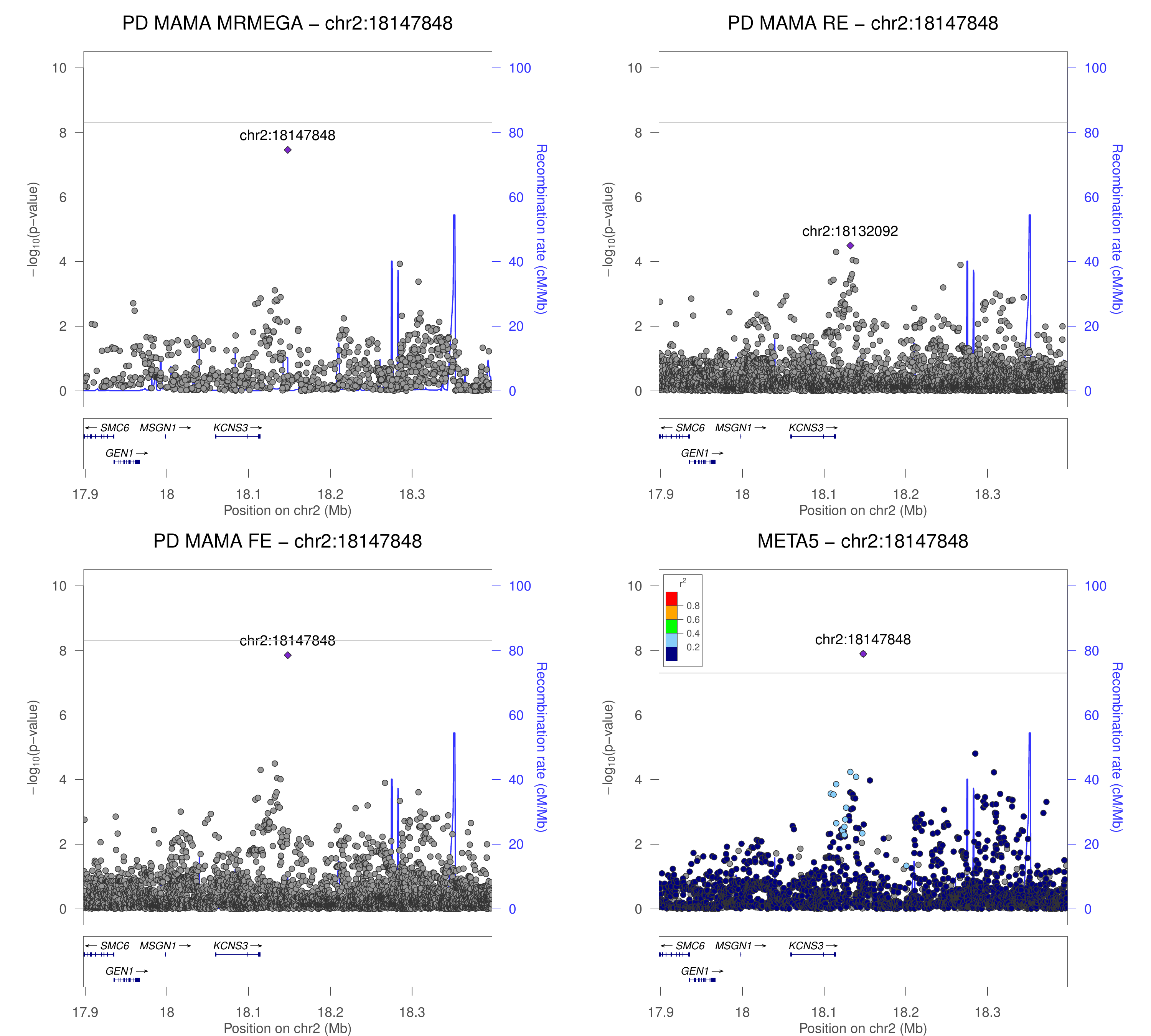

Supplement: Supplementary file 5 — This includes LocusZoom plots of all known European loci as well as novel loci. Each file contains four LocusZoom plots: PD MAMA MR-MEGA/RE/FE/ (MR-MEGA/random-effect/fixed-effect) and META5 (European-only meta-analysis from Nalls et al. 1). [file 41588_2023_1584_MOESM5_ESM.zip › LocusZoom plots of known EUR risk variants/chr2_17897848-18397848.png]

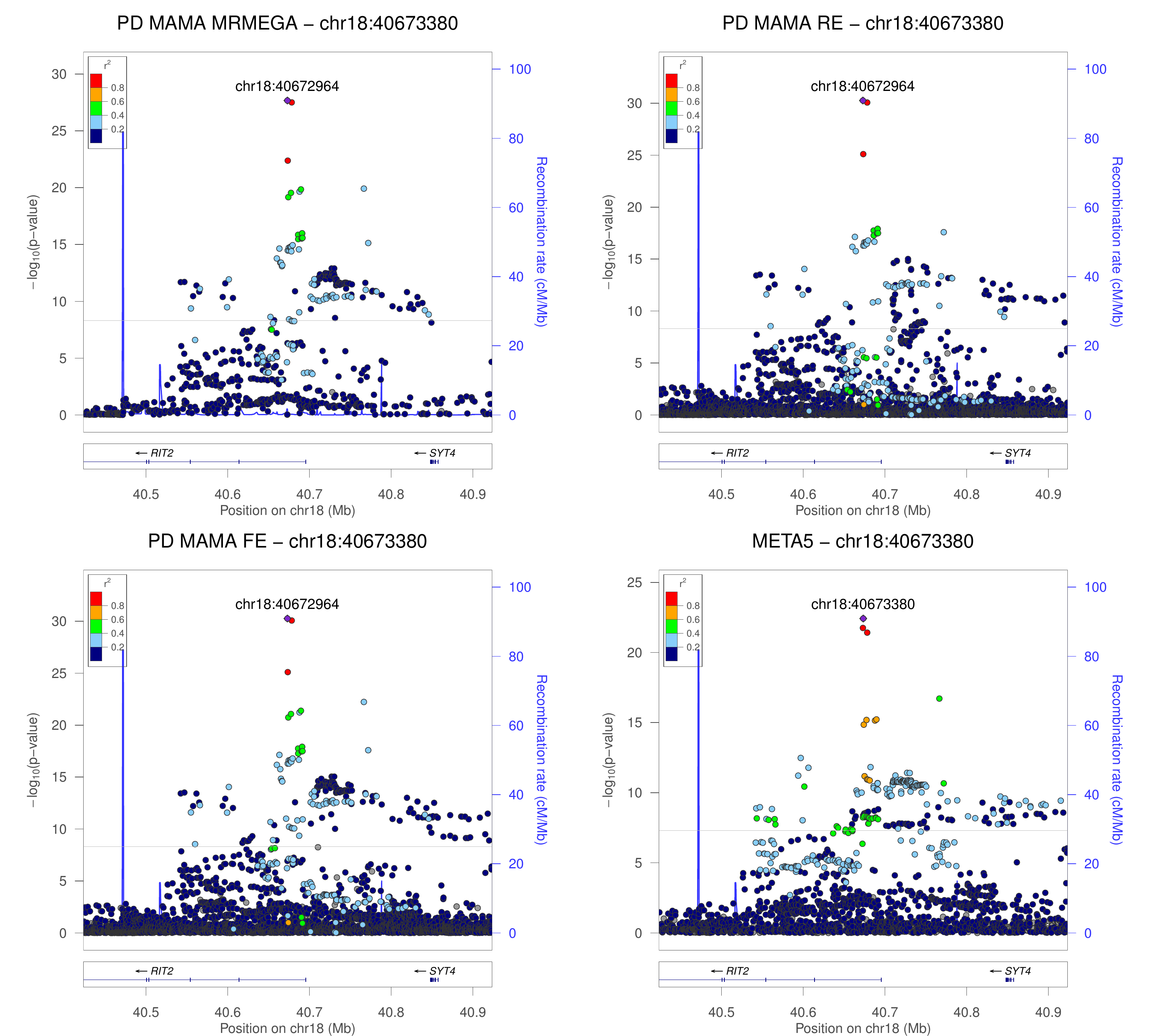

Supplement: Supplementary file 5 — This includes LocusZoom plots of all known European loci as well as novel loci. Each file contains four LocusZoom plots: PD MAMA MR-MEGA/RE/FE/ (MR-MEGA/random-effect/fixed-effect) and META5 (European-only meta-analysis from Nalls et al. 1). [file 41588_2023_1584_MOESM5_ESM.zip › LocusZoom plots of known EUR risk variants/chr18_40423380-40923380.png]

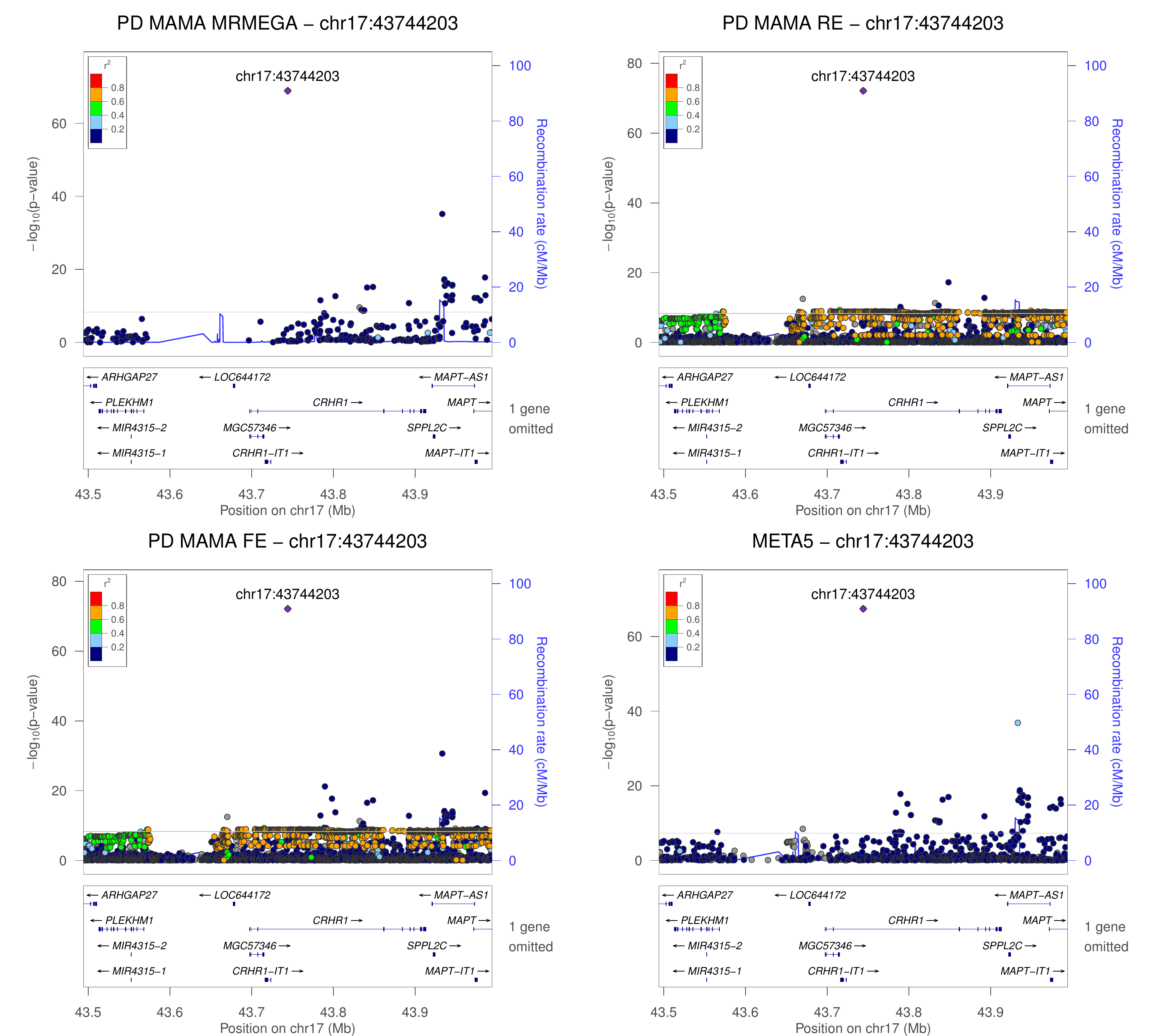

Supplement: Supplementary file 5 — This includes LocusZoom plots of all known European loci as well as novel loci. Each file contains four LocusZoom plots: PD MAMA MR-MEGA/RE/FE/ (MR-MEGA/random-effect/fixed-effect) and META5 (European-only meta-analysis from Nalls et al. 1). [file 41588_2023_1584_MOESM5_ESM.zip › LocusZoom plots of known EUR risk variants/chr17_43494203-43994203.png]

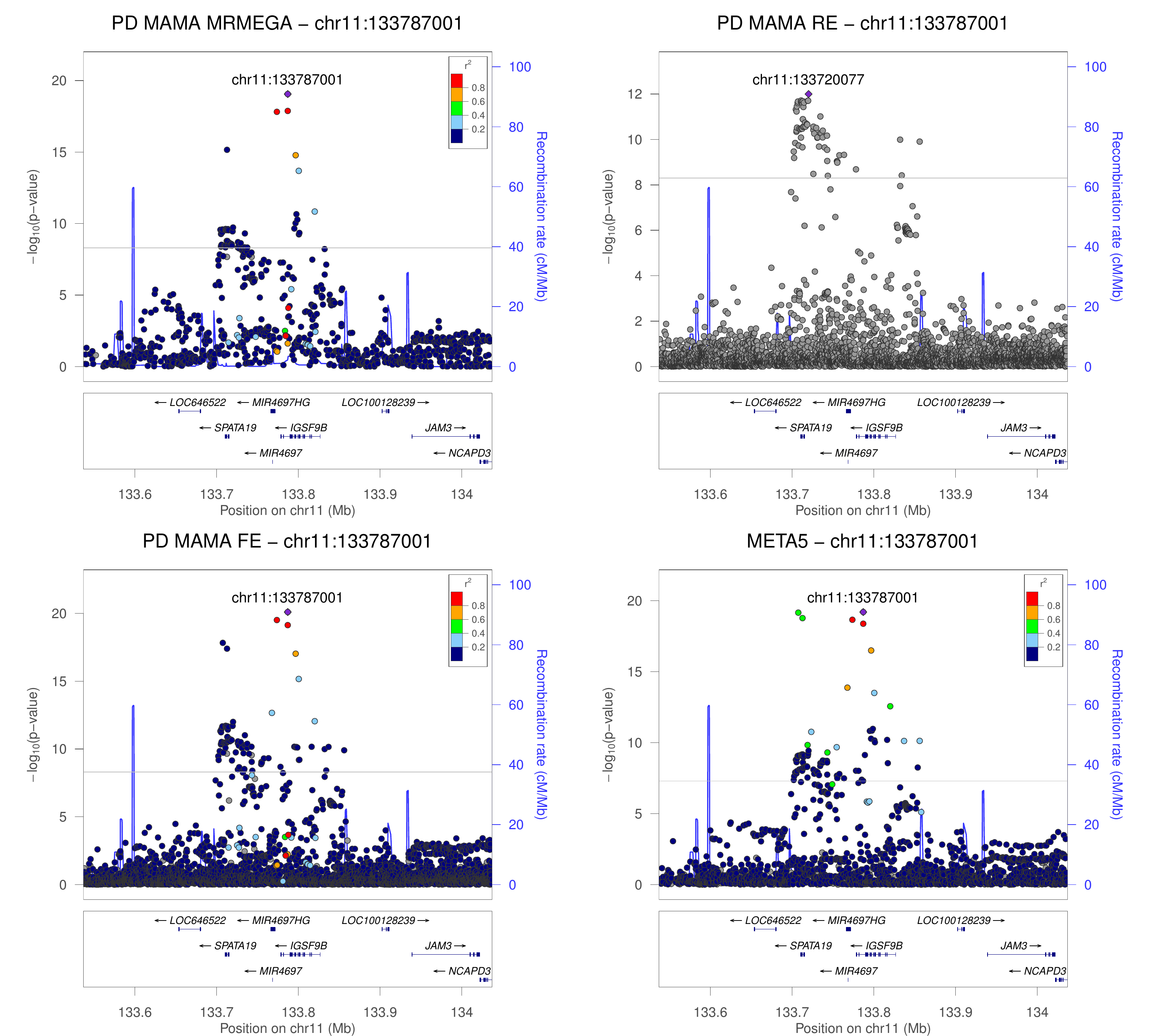

Supplement: Supplementary file 5 — This includes LocusZoom plots of all known European loci as well as novel loci. Each file contains four LocusZoom plots: PD MAMA MR-MEGA/RE/FE/ (MR-MEGA/random-effect/fixed-effect) and META5 (European-only meta-analysis from Nalls et al. 1). [file 41588_2023_1584_MOESM5_ESM.zip › LocusZoom plots of known EUR risk variants/chr11_133537001-134037001.png]

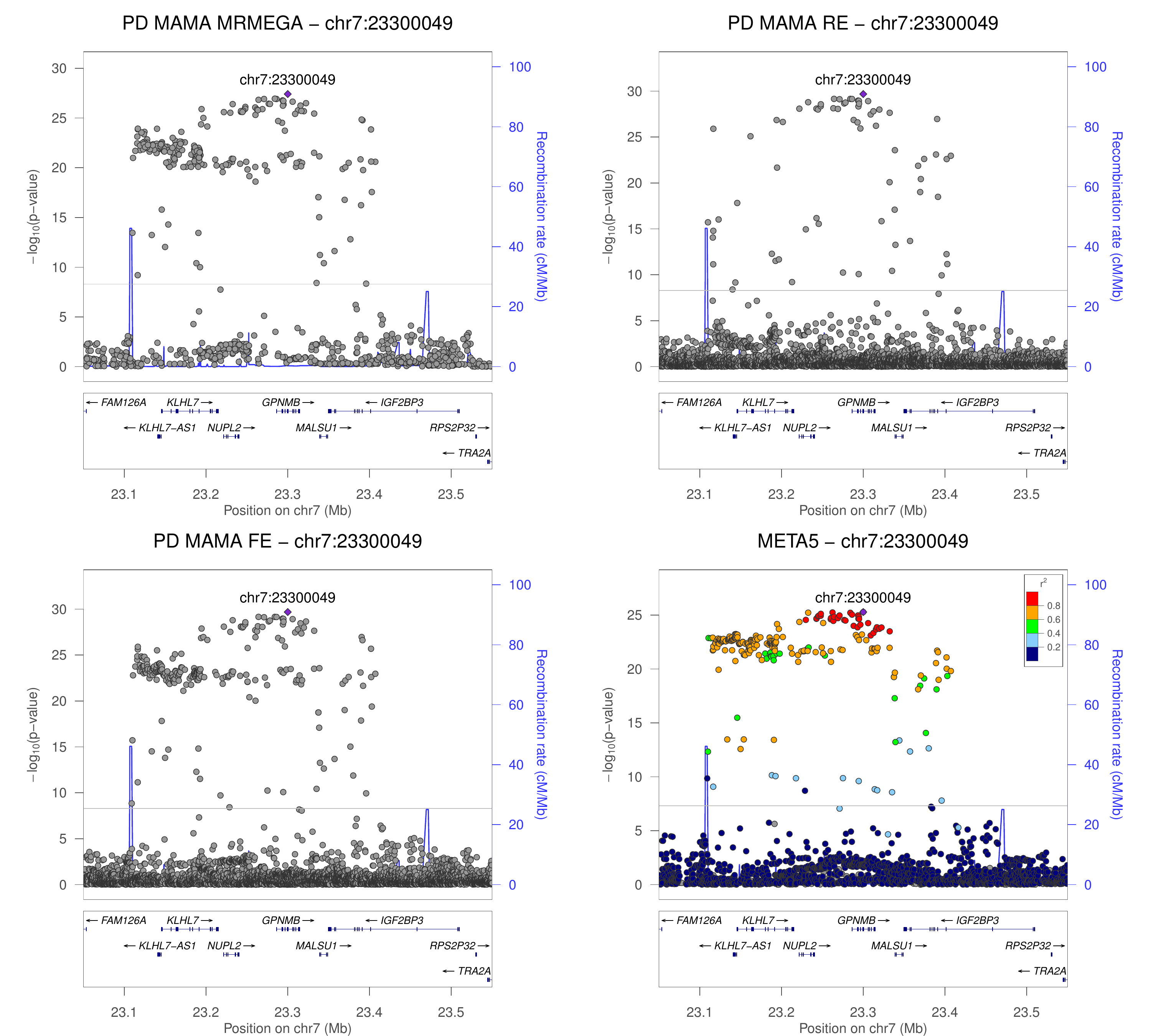

Supplement: Supplementary file 5 — This includes LocusZoom plots of all known European loci as well as novel loci. Each file contains four LocusZoom plots: PD MAMA MR-MEGA/RE/FE/ (MR-MEGA/random-effect/fixed-effect) and META5 (European-only meta-analysis from Nalls et al. 1). [file 41588_2023_1584_MOESM5_ESM.zip › LocusZoom plots of known EUR risk variants/chr7_23050049-23550049.png]

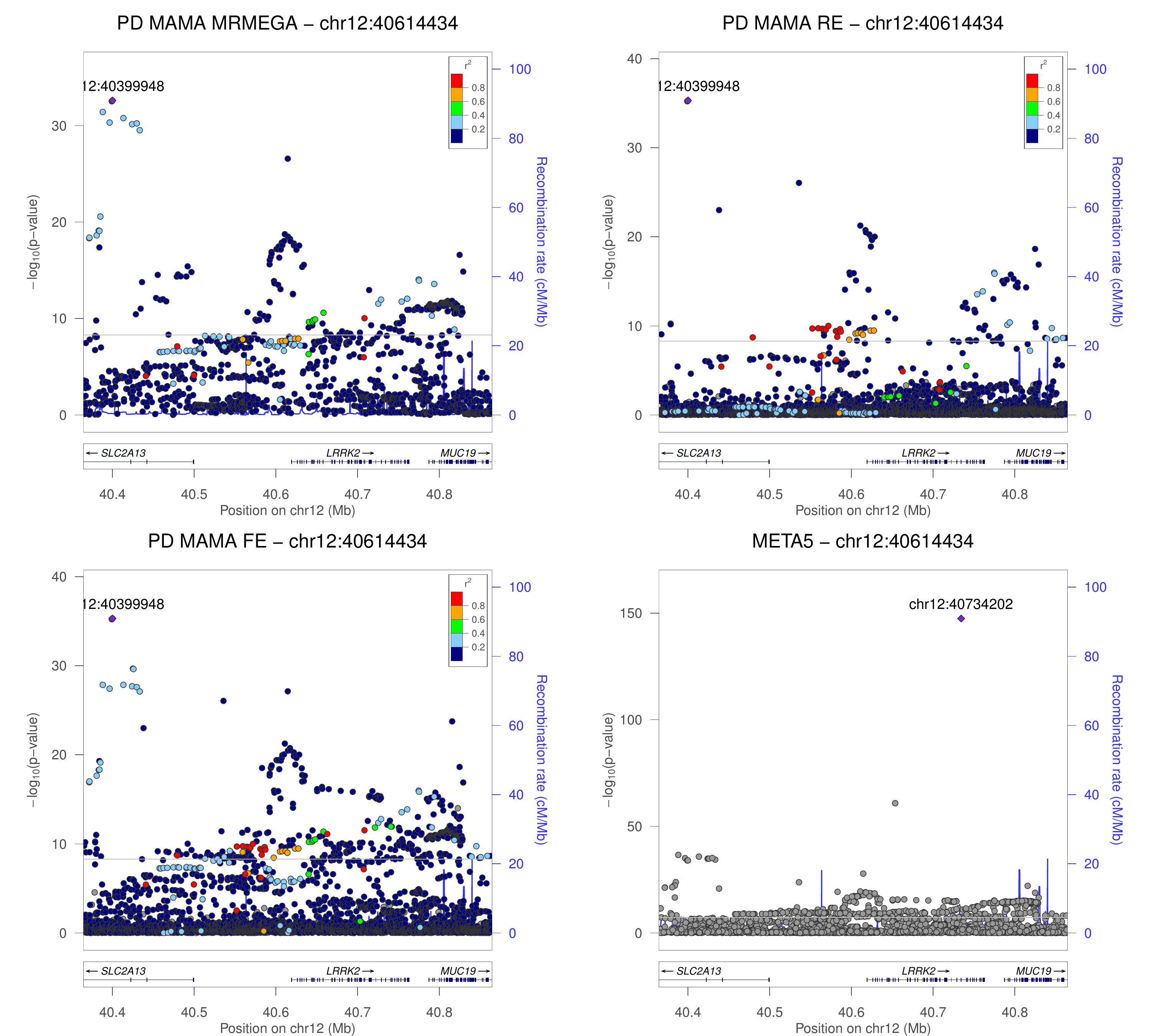

Supplement: Supplementary file 5 — This includes LocusZoom plots of all known European loci as well as novel loci. Each file contains four LocusZoom plots: PD MAMA MR-MEGA/RE/FE/ (MR-MEGA/random-effect/fixed-effect) and META5 (European-only meta-analysis from Nalls et al. 1). [file 41588_2023_1584_MOESM5_ESM.zip › LocusZoom plots of known EUR risk variants/chr12_40364434-40864434.png]

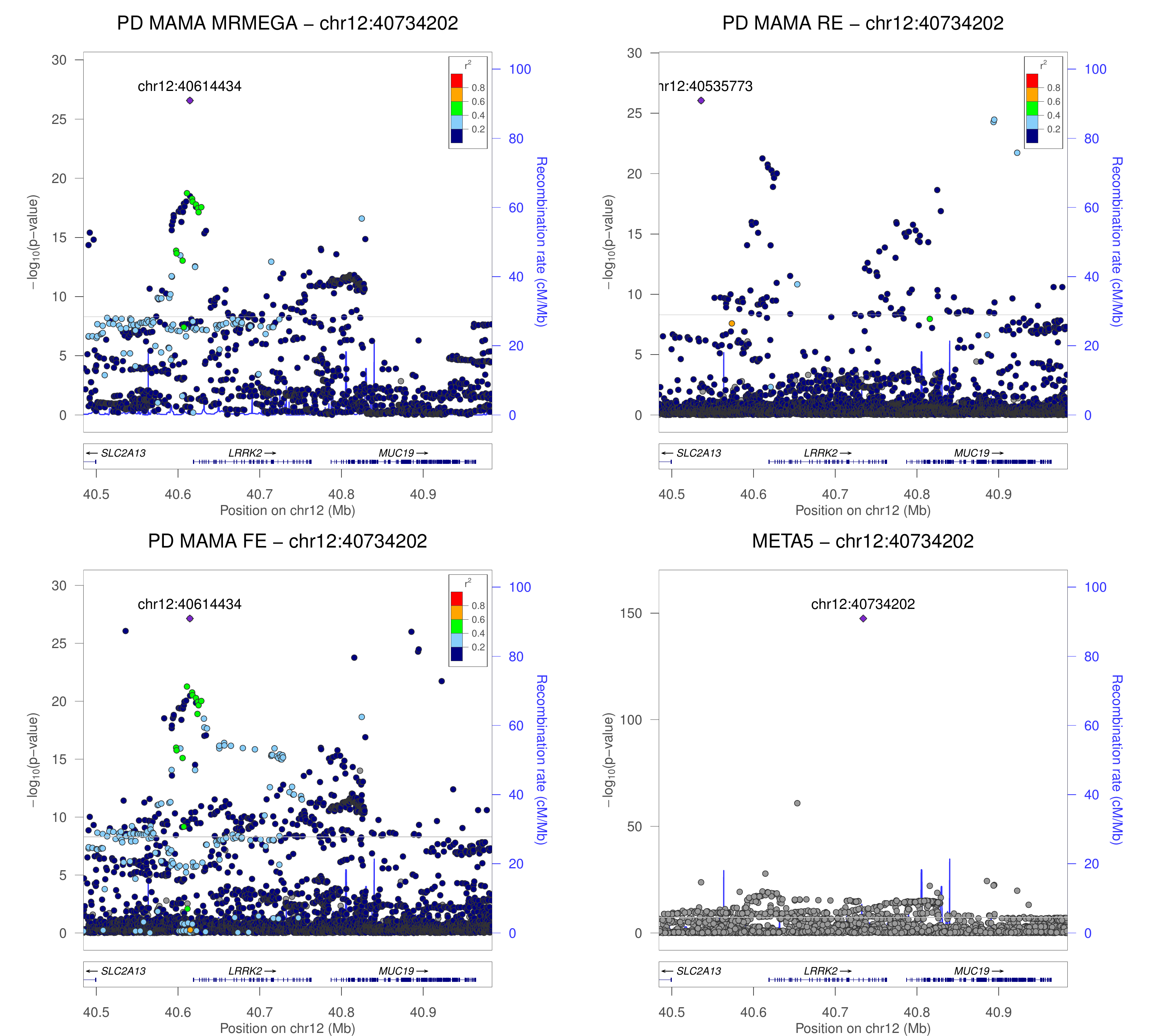

Supplement: Supplementary file 5 — This includes LocusZoom plots of all known European loci as well as novel loci. Each file contains four LocusZoom plots: PD MAMA MR-MEGA/RE/FE/ (MR-MEGA/random-effect/fixed-effect) and META5 (European-only meta-analysis from Nalls et al. 1). [file 41588_2023_1584_MOESM5_ESM.zip › LocusZoom plots of known EUR risk variants/chr12_40484202-40984202.png]

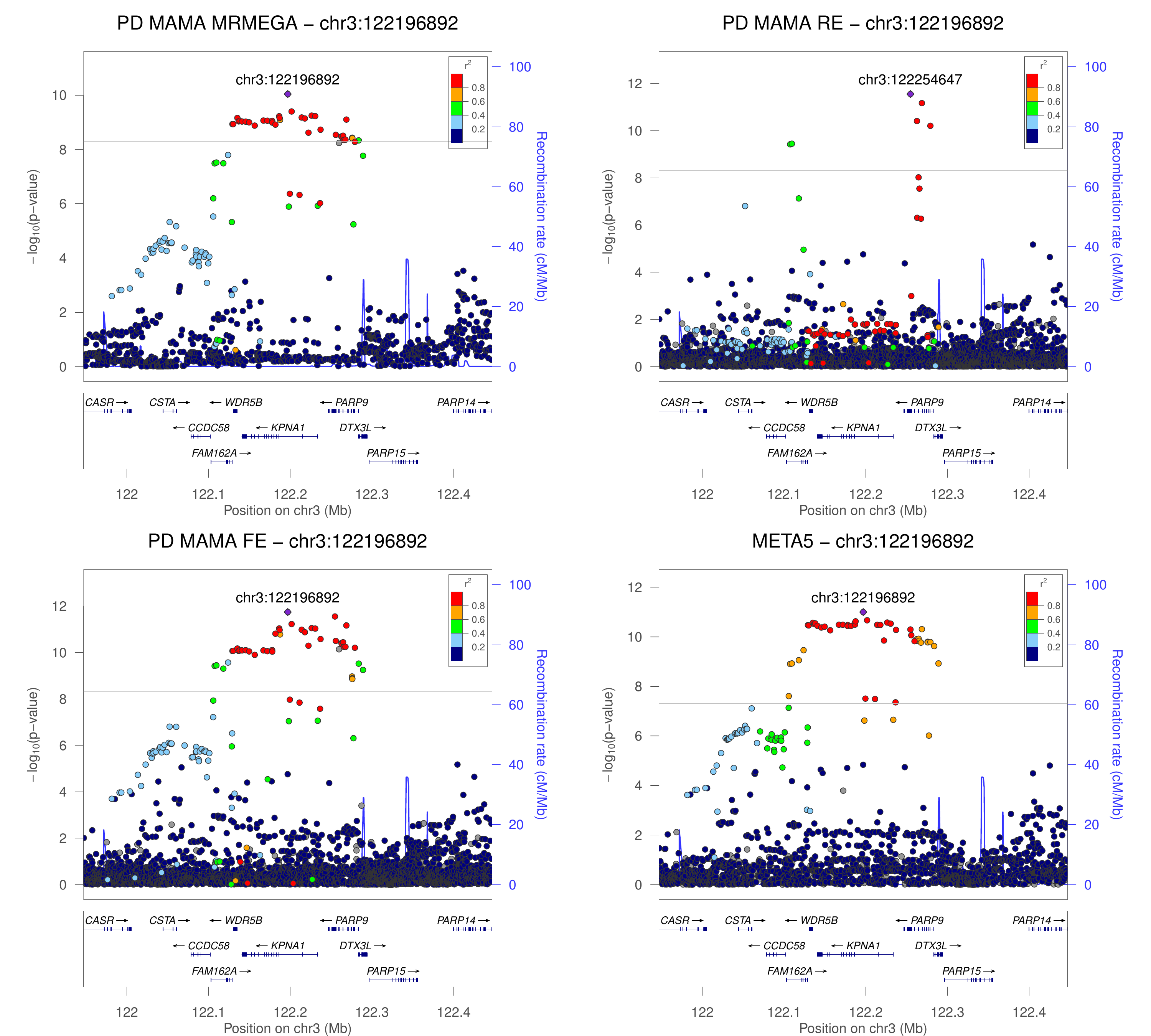

Supplement: Supplementary file 5 — This includes LocusZoom plots of all known European loci as well as novel loci. Each file contains four LocusZoom plots: PD MAMA MR-MEGA/RE/FE/ (MR-MEGA/random-effect/fixed-effect) and META5 (European-only meta-analysis from Nalls et al. 1). [file 41588_2023_1584_MOESM5_ESM.zip › LocusZoom plots of known EUR risk variants/chr3_121946892-122446892.png]

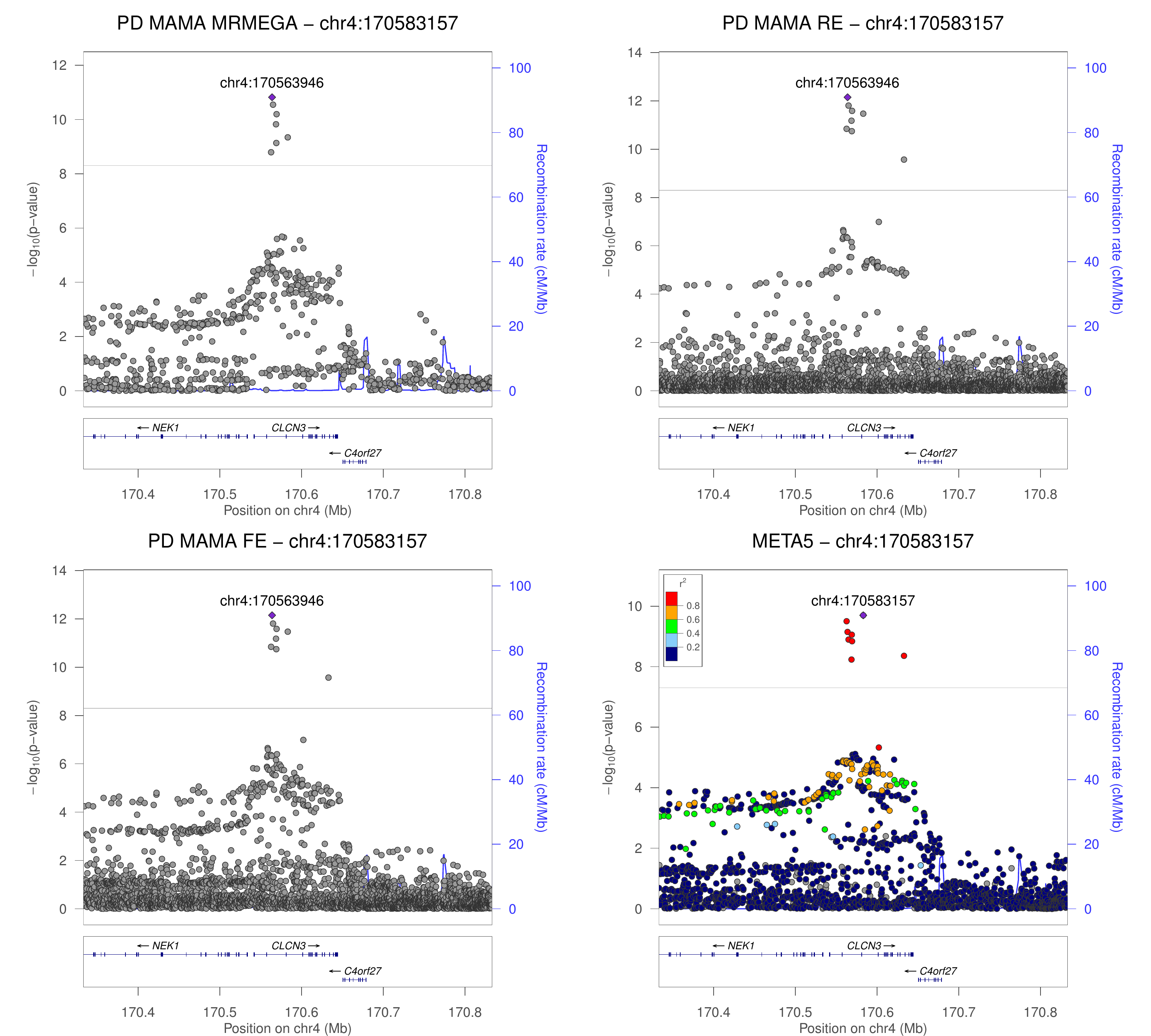

Supplement: Supplementary file 5 — This includes LocusZoom plots of all known European loci as well as novel loci. Each file contains four LocusZoom plots: PD MAMA MR-MEGA/RE/FE/ (MR-MEGA/random-effect/fixed-effect) and META5 (European-only meta-analysis from Nalls et al. 1). [file 41588_2023_1584_MOESM5_ESM.zip › LocusZoom plots of known EUR risk variants/chr4_170333157-170833157.png]

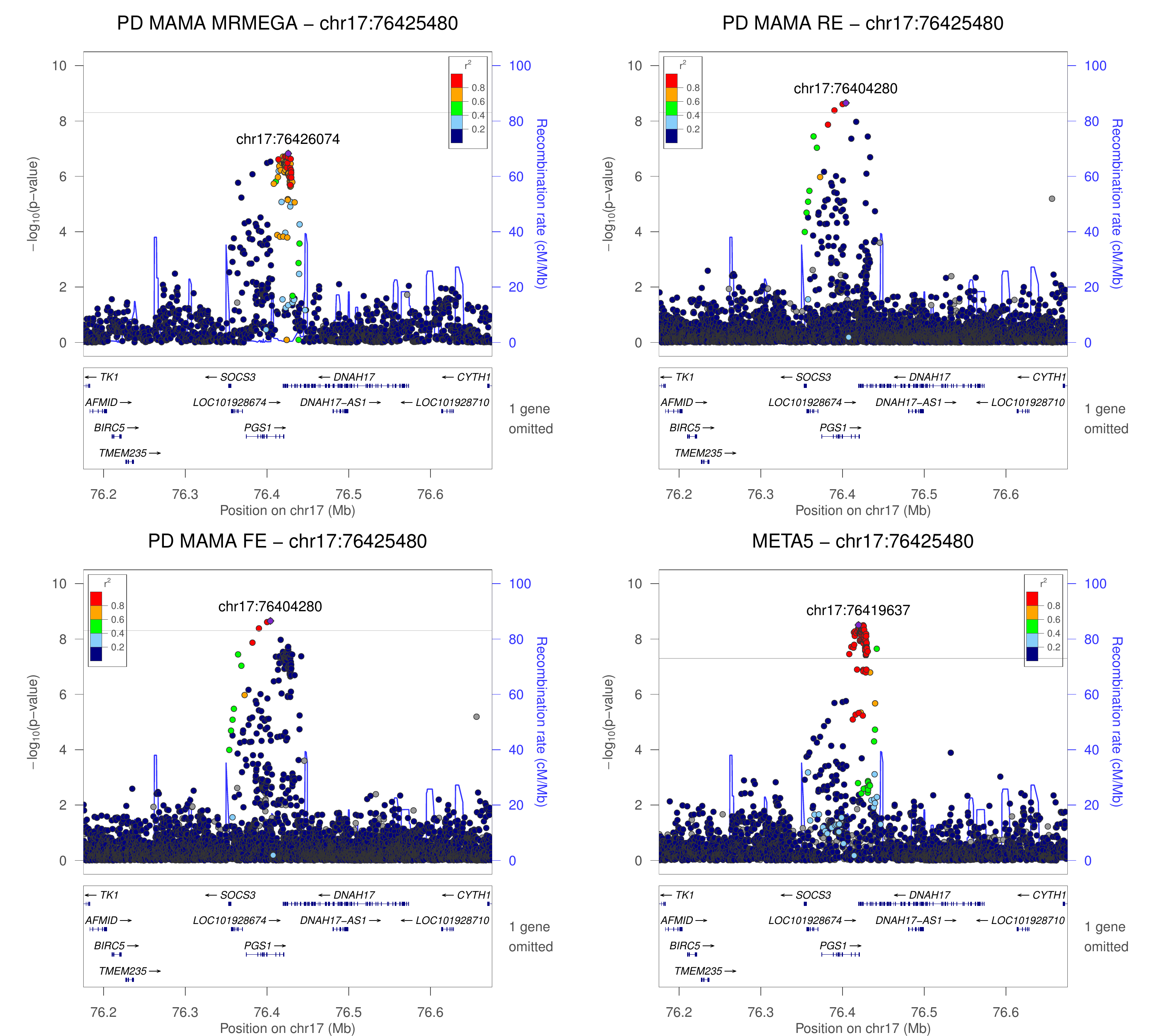

Supplement: Supplementary file 5 — This includes LocusZoom plots of all known European loci as well as novel loci. Each file contains four LocusZoom plots: PD MAMA MR-MEGA/RE/FE/ (MR-MEGA/random-effect/fixed-effect) and META5 (European-only meta-analysis from Nalls et al. 1). [file 41588_2023_1584_MOESM5_ESM.zip › LocusZoom plots of known EUR risk variants/chr17_76175480-76675480.png]

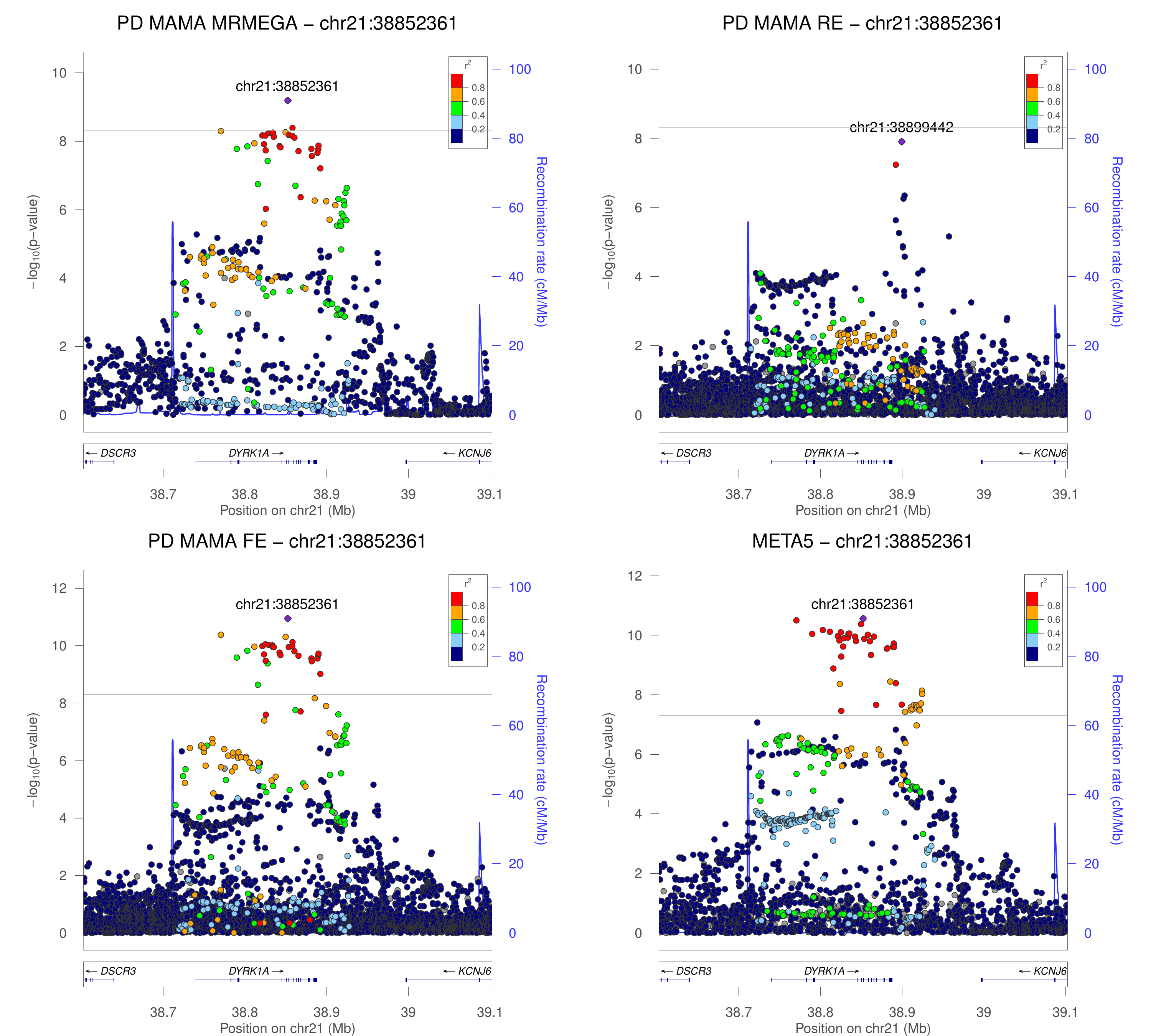

Supplement: Supplementary file 5 — This includes LocusZoom plots of all known European loci as well as novel loci. Each file contains four LocusZoom plots: PD MAMA MR-MEGA/RE/FE/ (MR-MEGA/random-effect/fixed-effect) and META5 (European-only meta-analysis from Nalls et al. 1). [file 41588_2023_1584_MOESM5_ESM.zip › LocusZoom plots of known EUR risk variants/chr21_38602361-39102361.png]

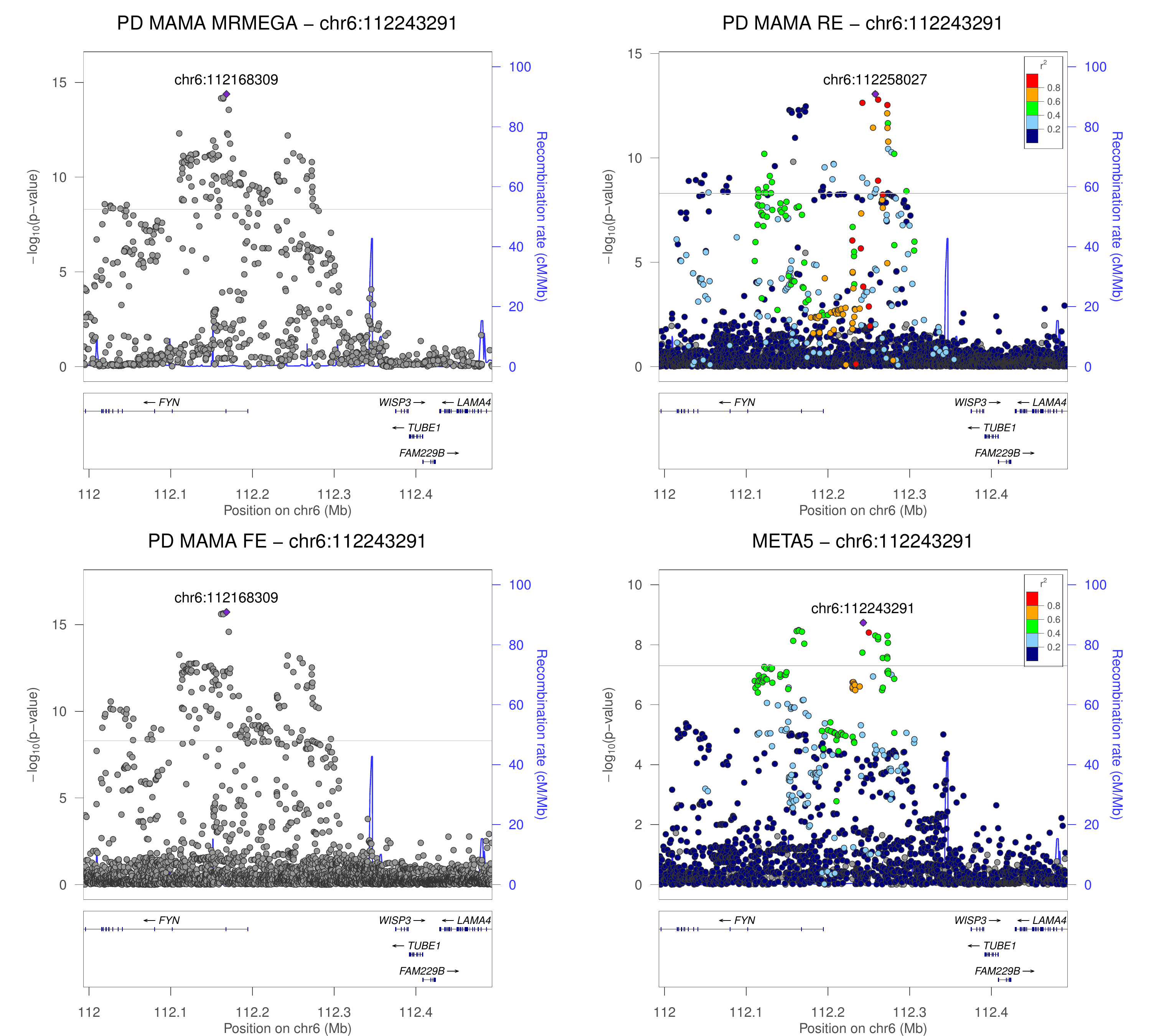

Supplement: Supplementary file 5 — This includes LocusZoom plots of all known European loci as well as novel loci. Each file contains four LocusZoom plots: PD MAMA MR-MEGA/RE/FE/ (MR-MEGA/random-effect/fixed-effect) and META5 (European-only meta-analysis from Nalls et al. 1). [file 41588_2023_1584_MOESM5_ESM.zip › LocusZoom plots of known EUR risk variants/chr6_111993291-112493291.png]

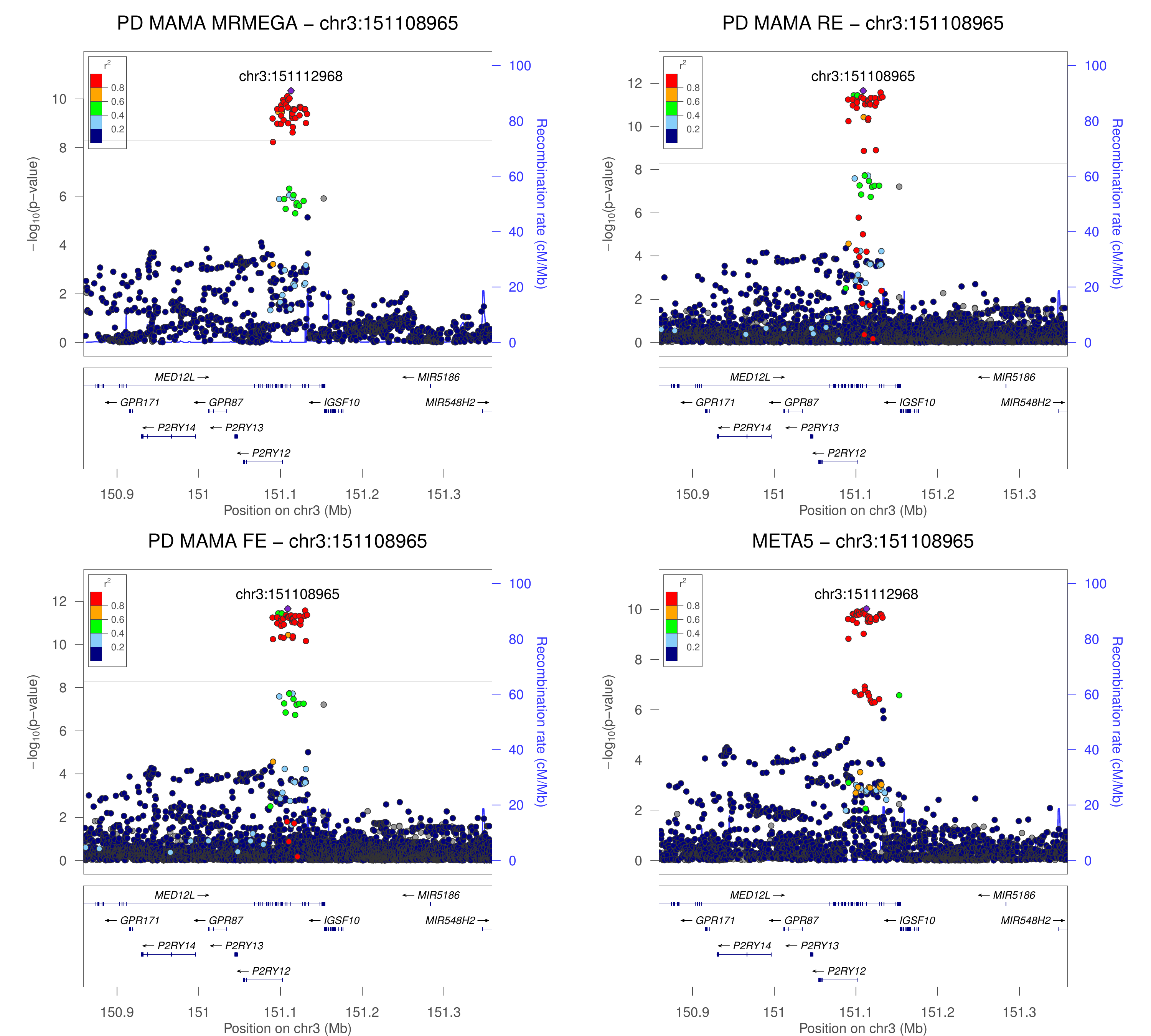

Supplement: Supplementary file 5 — This includes LocusZoom plots of all known European loci as well as novel loci. Each file contains four LocusZoom plots: PD MAMA MR-MEGA/RE/FE/ (MR-MEGA/random-effect/fixed-effect) and META5 (European-only meta-analysis from Nalls et al. 1). [file 41588_2023_1584_MOESM5_ESM.zip › LocusZoom plots of known EUR risk variants/chr3_150858965-151358965.png]

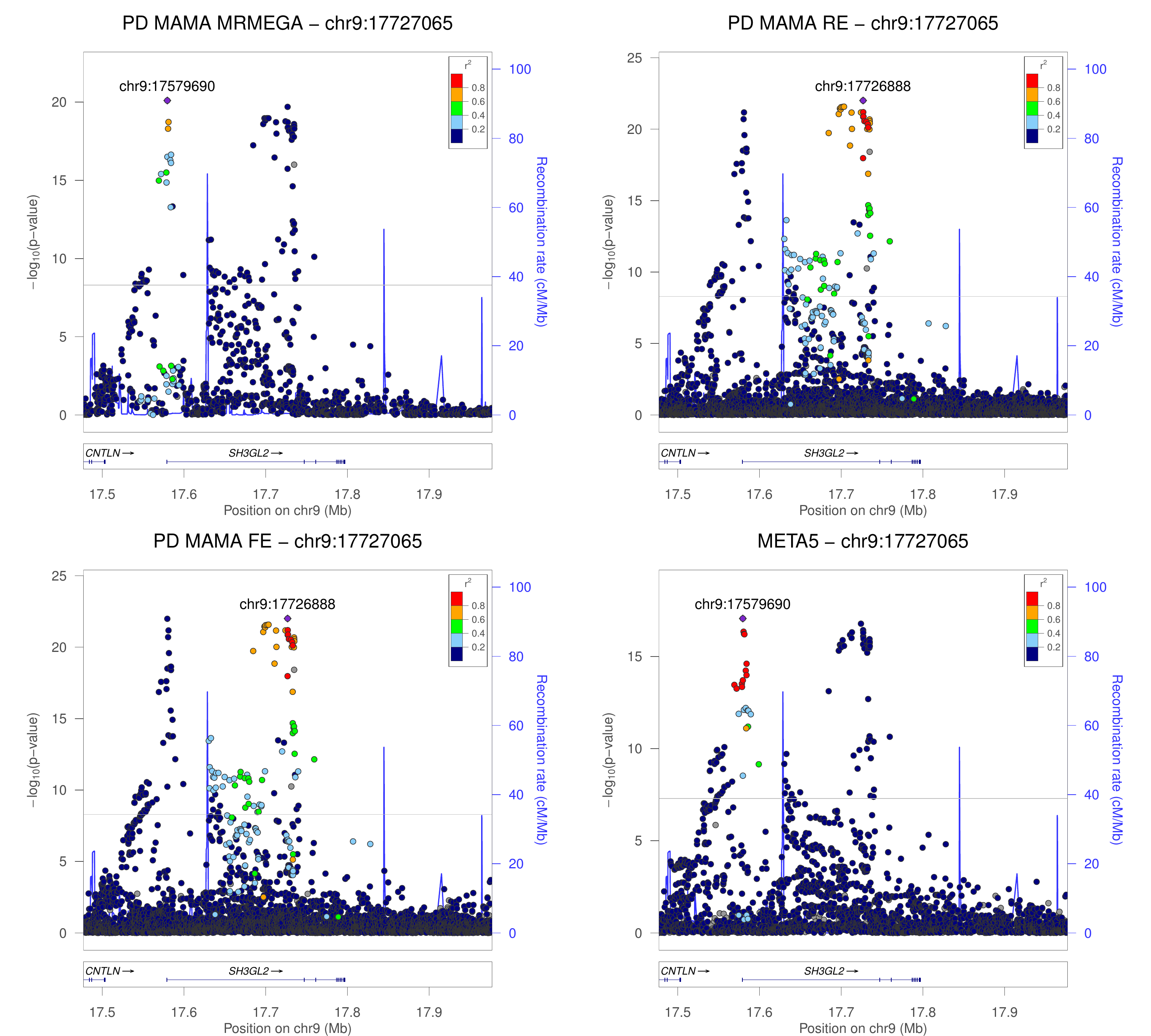

Supplement: Supplementary file 5 — This includes LocusZoom plots of all known European loci as well as novel loci. Each file contains four LocusZoom plots: PD MAMA MR-MEGA/RE/FE/ (MR-MEGA/random-effect/fixed-effect) and META5 (European-only meta-analysis from Nalls et al. 1). [file 41588_2023_1584_MOESM5_ESM.zip › LocusZoom plots of known EUR risk variants/chr9_17477065-17977065.png]

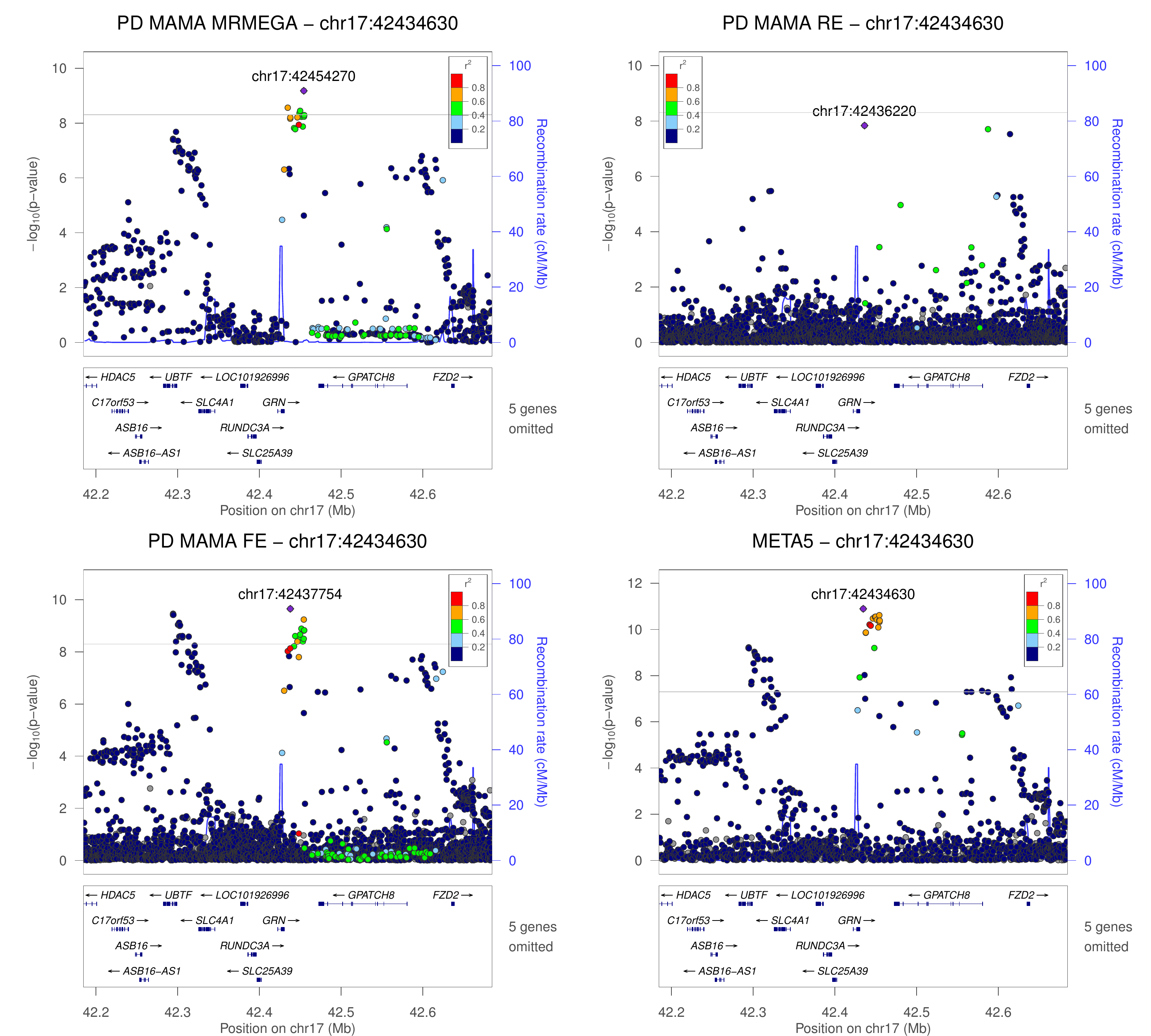

Supplement: Supplementary file 5 — This includes LocusZoom plots of all known European loci as well as novel loci. Each file contains four LocusZoom plots: PD MAMA MR-MEGA/RE/FE/ (MR-MEGA/random-effect/fixed-effect) and META5 (European-only meta-analysis from Nalls et al. 1). [file 41588_2023_1584_MOESM5_ESM.zip › LocusZoom plots of known EUR risk variants/chr17_42184630-42684630.png]

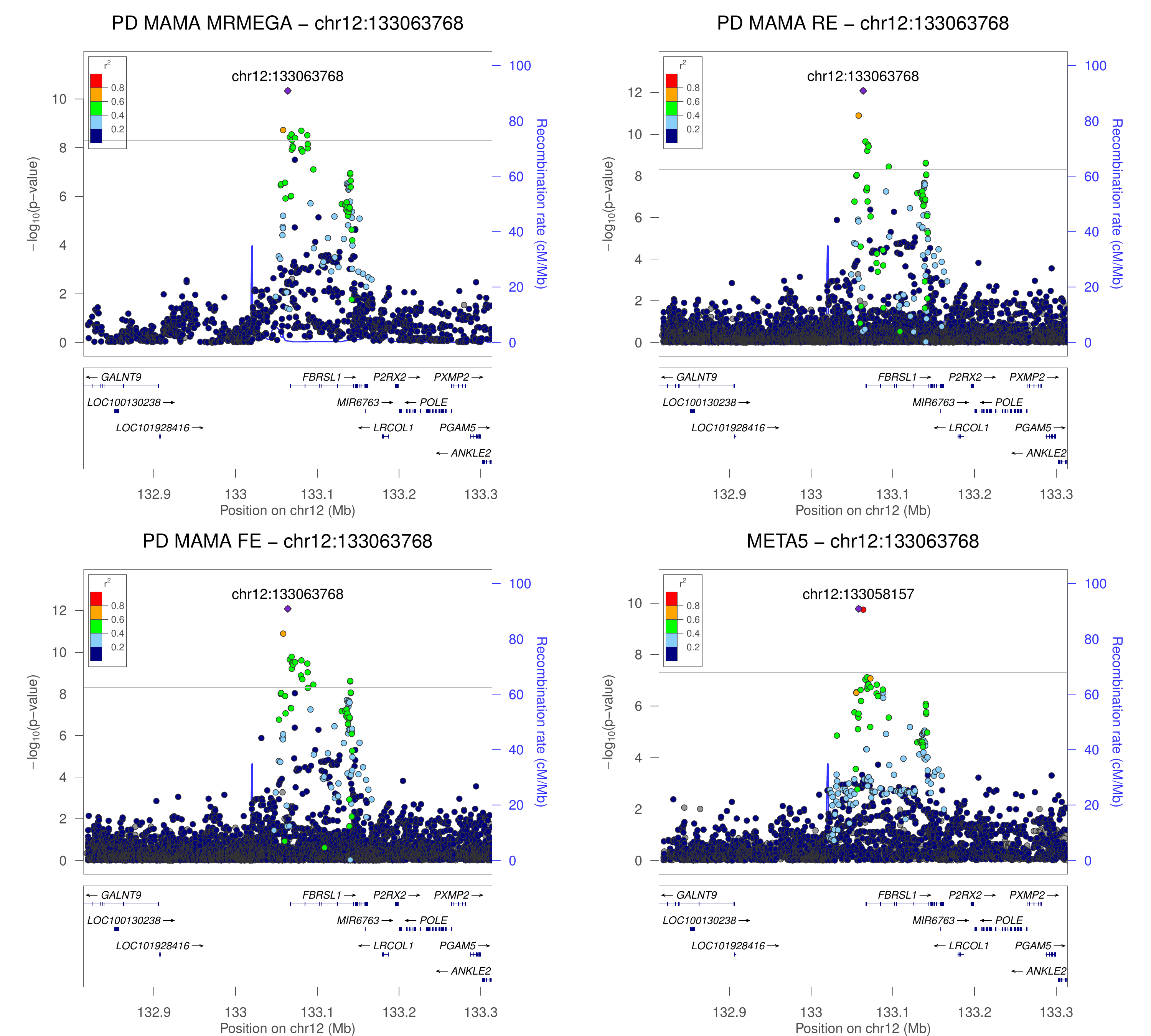

Supplement: Supplementary file 5 — This includes LocusZoom plots of all known European loci as well as novel loci. Each file contains four LocusZoom plots: PD MAMA MR-MEGA/RE/FE/ (MR-MEGA/random-effect/fixed-effect) and META5 (European-only meta-analysis from Nalls et al. 1). [file 41588_2023_1584_MOESM5_ESM.zip › LocusZoom plots of known EUR risk variants/chr12_132813768-133313768.png]

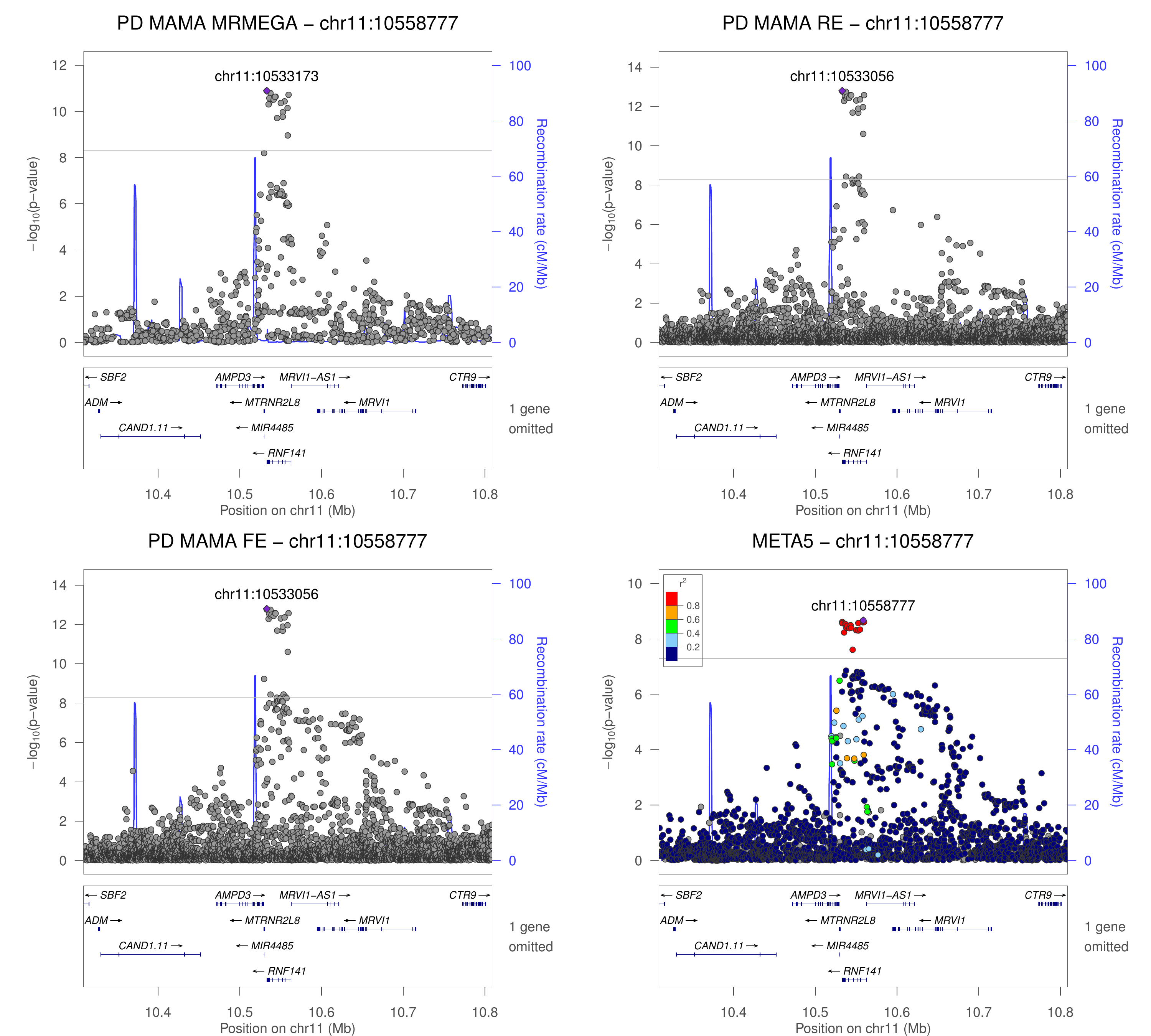

Supplement: Supplementary file 5 — This includes LocusZoom plots of all known European loci as well as novel loci. Each file contains four LocusZoom plots: PD MAMA MR-MEGA/RE/FE/ (MR-MEGA/random-effect/fixed-effect) and META5 (European-only meta-analysis from Nalls et al. 1). [file 41588_2023_1584_MOESM5_ESM.zip › LocusZoom plots of known EUR risk variants/chr11_10308777-10808777.png]

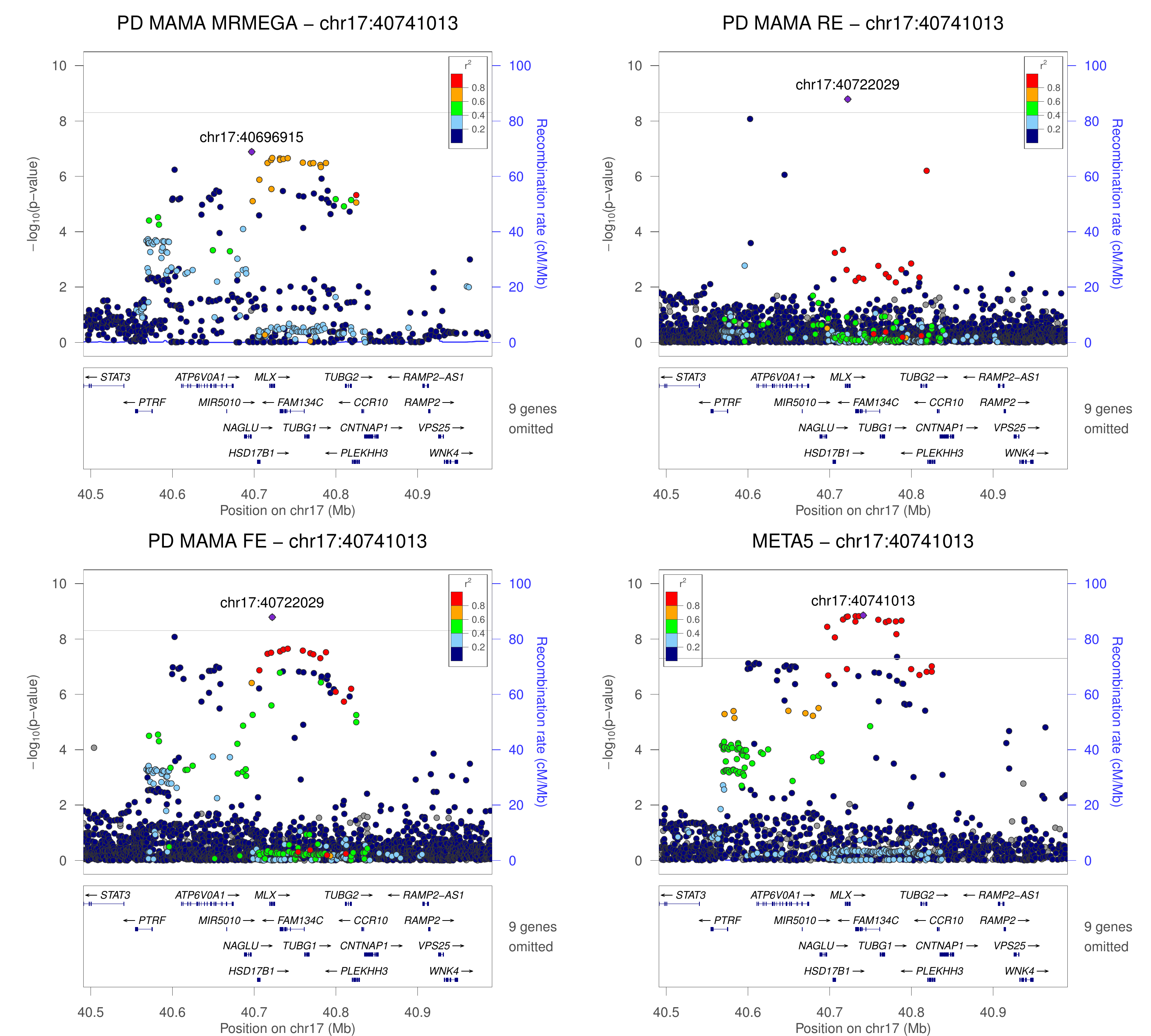

Supplement: Supplementary file 5 — This includes LocusZoom plots of all known European loci as well as novel loci. Each file contains four LocusZoom plots: PD MAMA MR-MEGA/RE/FE/ (MR-MEGA/random-effect/fixed-effect) and META5 (European-only meta-analysis from Nalls et al. 1). [file 41588_2023_1584_MOESM5_ESM.zip › LocusZoom plots of known EUR risk variants/chr17_40491013-40991013.png]

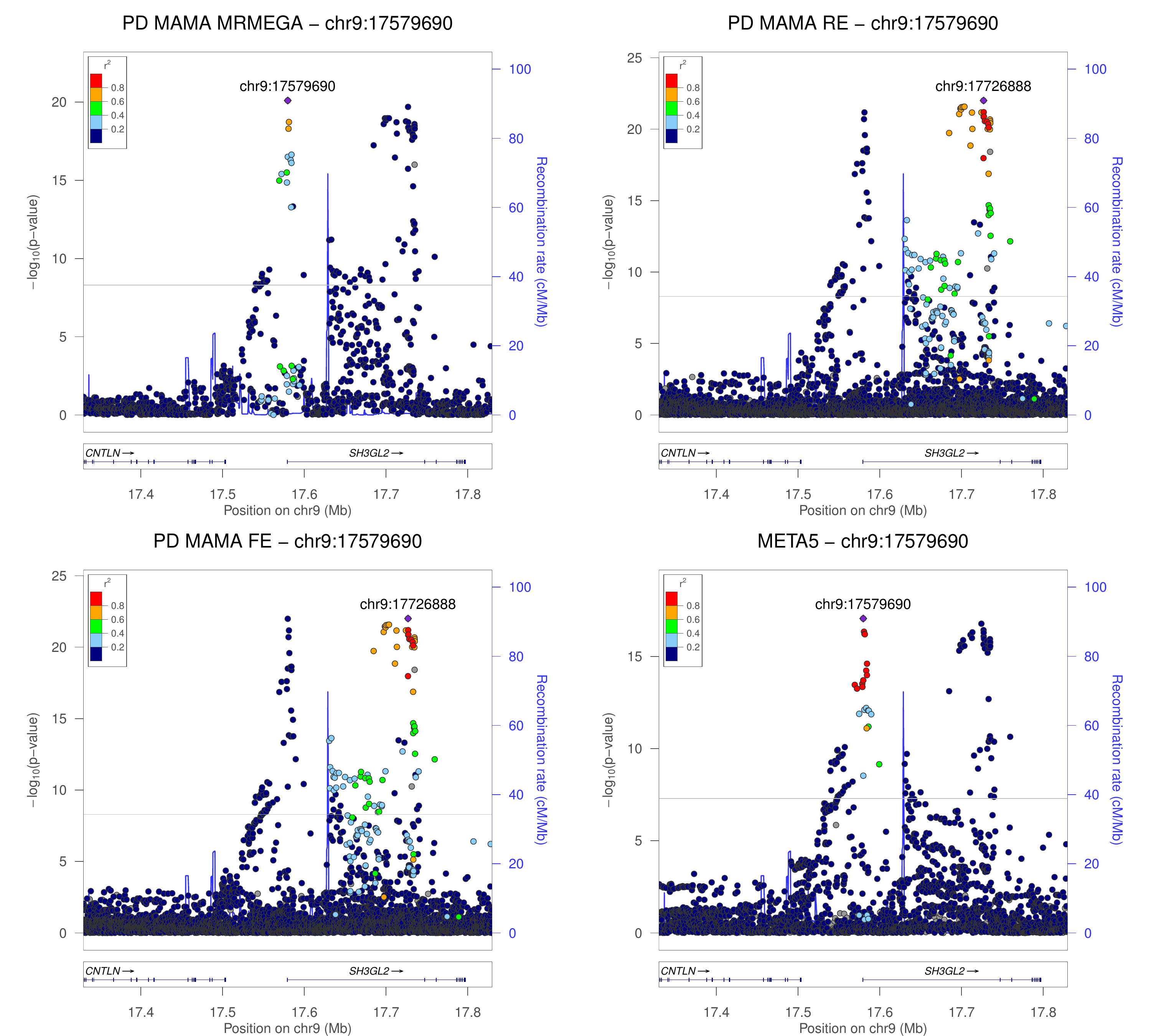

Supplement: Supplementary file 5 — This includes LocusZoom plots of all known European loci as well as novel loci. Each file contains four LocusZoom plots: PD MAMA MR-MEGA/RE/FE/ (MR-MEGA/random-effect/fixed-effect) and META5 (European-only meta-analysis from Nalls et al. 1). [file 41588_2023_1584_MOESM5_ESM.zip › LocusZoom plots of known EUR risk variants/chr9_17329690-17829690.png]

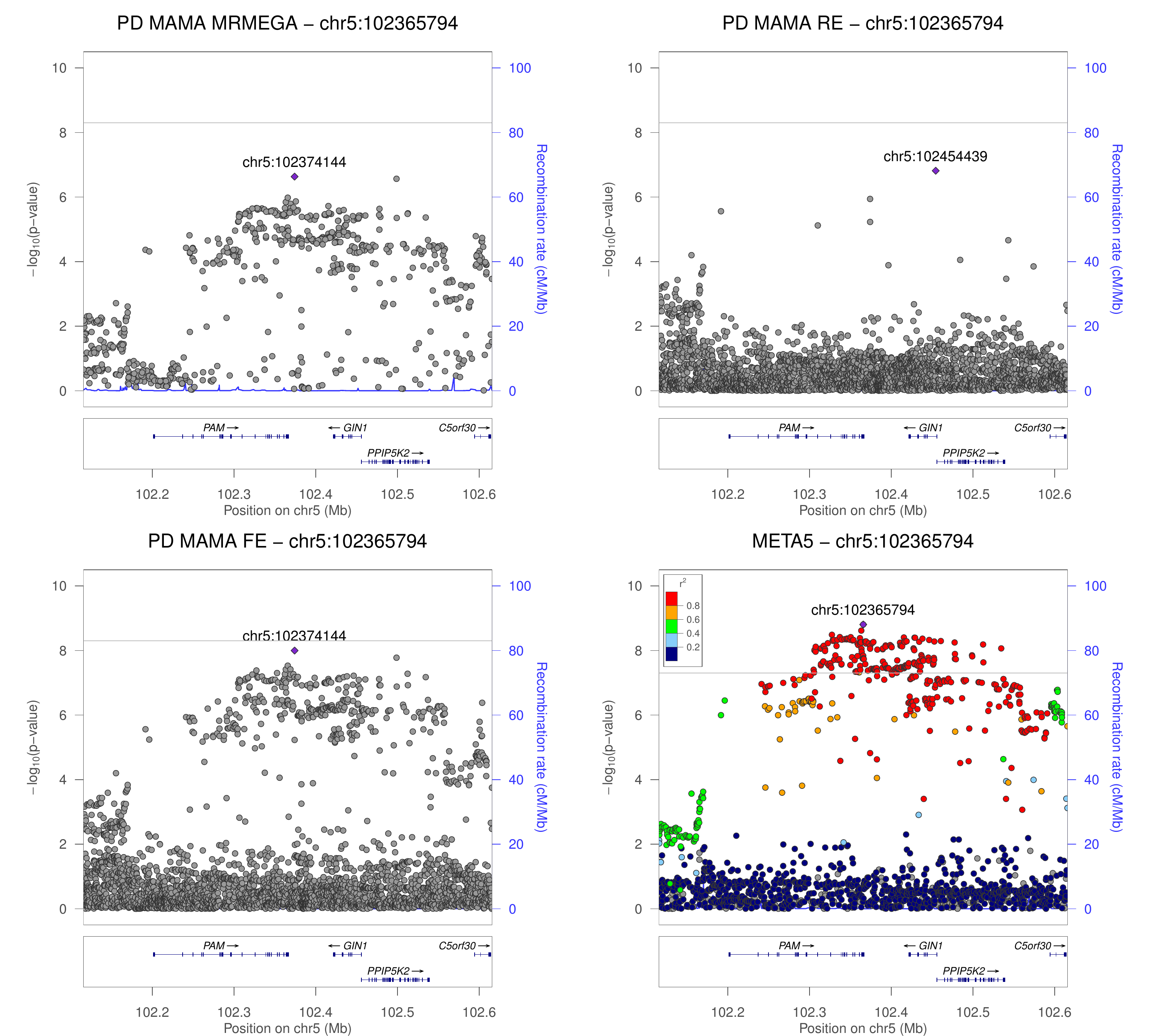

Supplement: Supplementary file 5 — This includes LocusZoom plots of all known European loci as well as novel loci. Each file contains four LocusZoom plots: PD MAMA MR-MEGA/RE/FE/ (MR-MEGA/random-effect/fixed-effect) and META5 (European-only meta-analysis from Nalls et al. 1). [file 41588_2023_1584_MOESM5_ESM.zip › LocusZoom plots of known EUR risk variants/chr5_102115794-102615794.png]

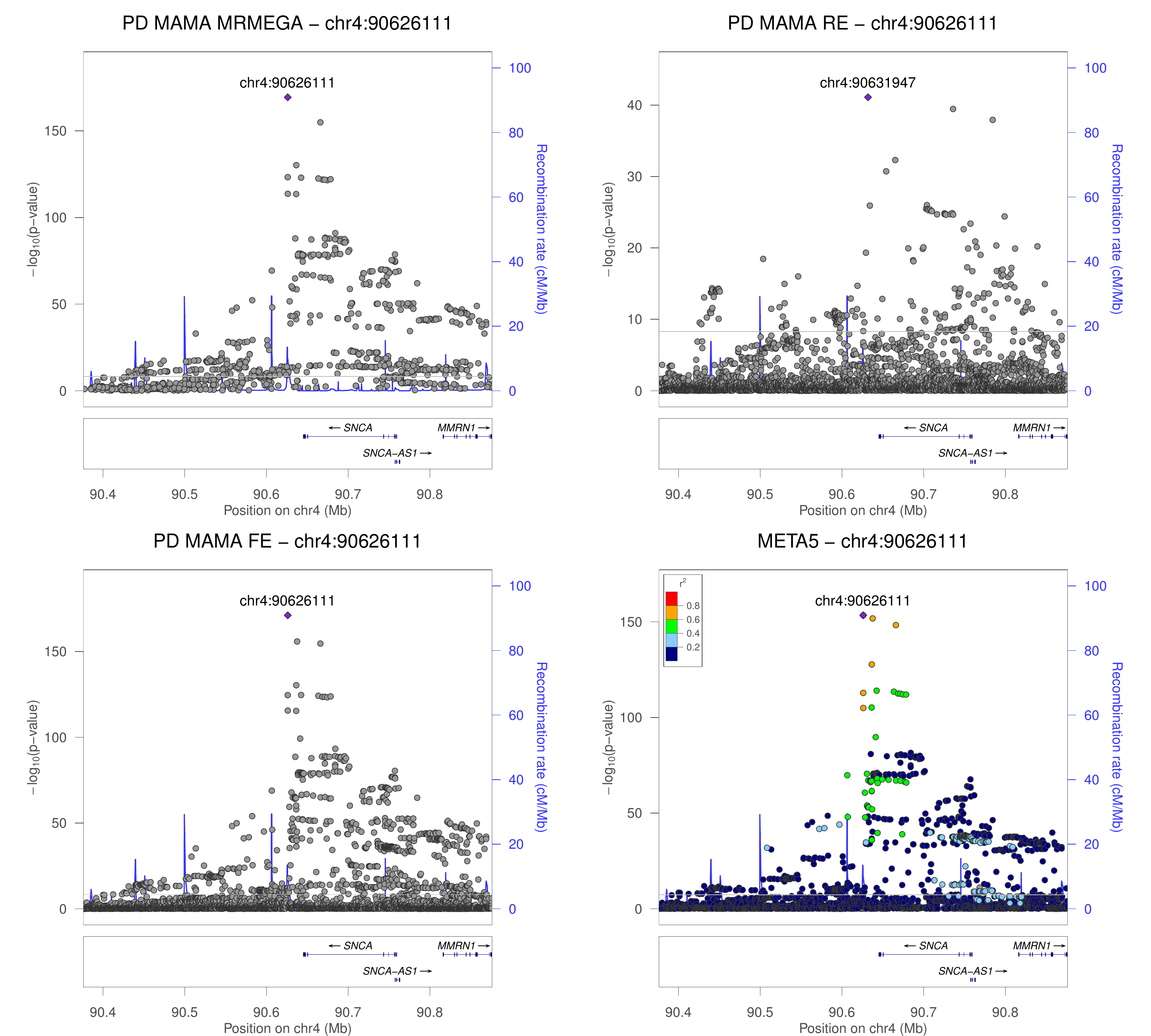

Supplement: Supplementary file 5 — This includes LocusZoom plots of all known European loci as well as novel loci. Each file contains four LocusZoom plots: PD MAMA MR-MEGA/RE/FE/ (MR-MEGA/random-effect/fixed-effect) and META5 (European-only meta-analysis from Nalls et al. 1). [file 41588_2023_1584_MOESM5_ESM.zip › LocusZoom plots of known EUR risk variants/chr4_90376111-90876111.png]

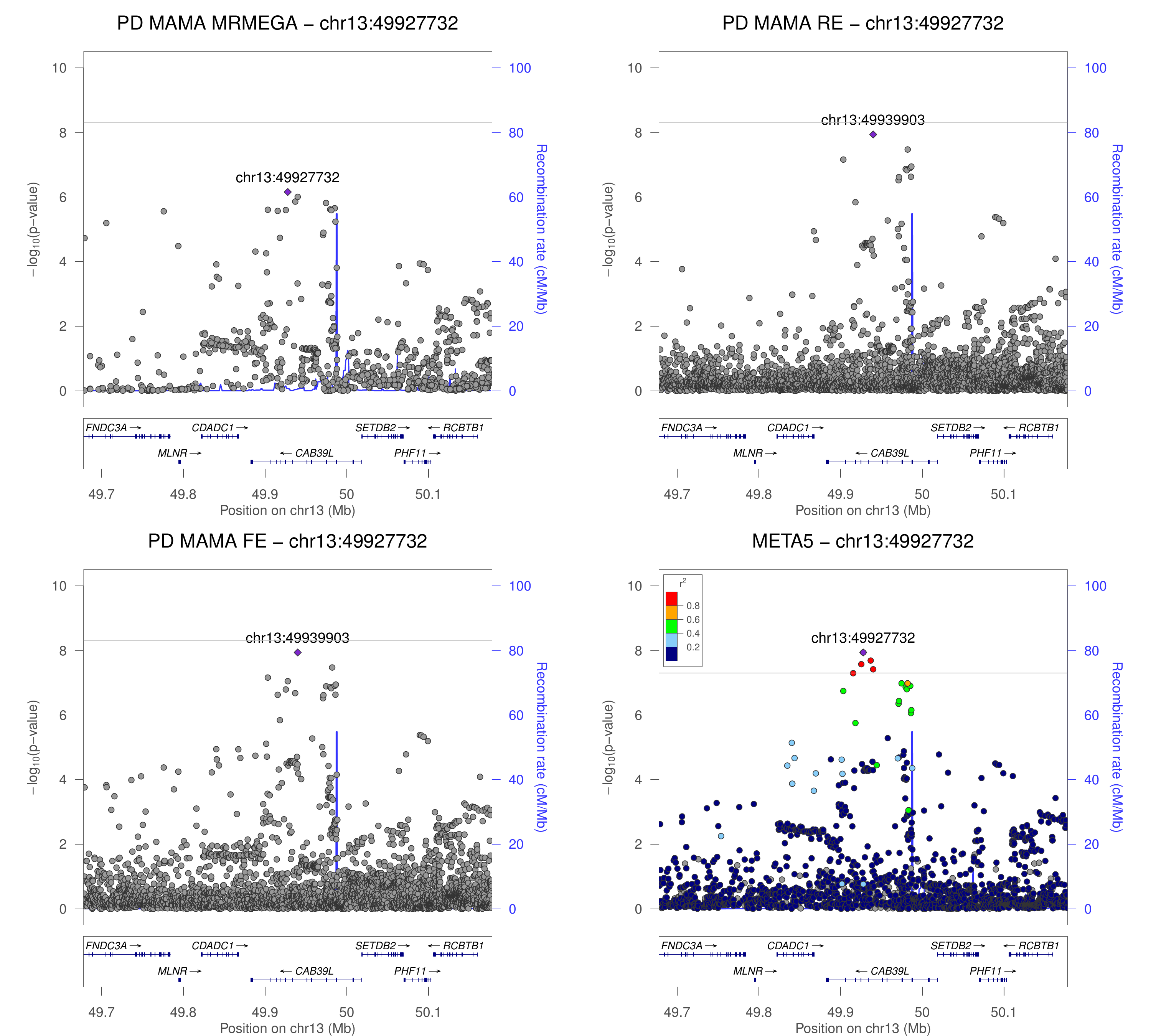

Supplement: Supplementary file 5 — This includes LocusZoom plots of all known European loci as well as novel loci. Each file contains four LocusZoom plots: PD MAMA MR-MEGA/RE/FE/ (MR-MEGA/random-effect/fixed-effect) and META5 (European-only meta-analysis from Nalls et al. 1). [file 41588_2023_1584_MOESM5_ESM.zip › LocusZoom plots of known EUR risk variants/chr13_49677732-50177732.png]

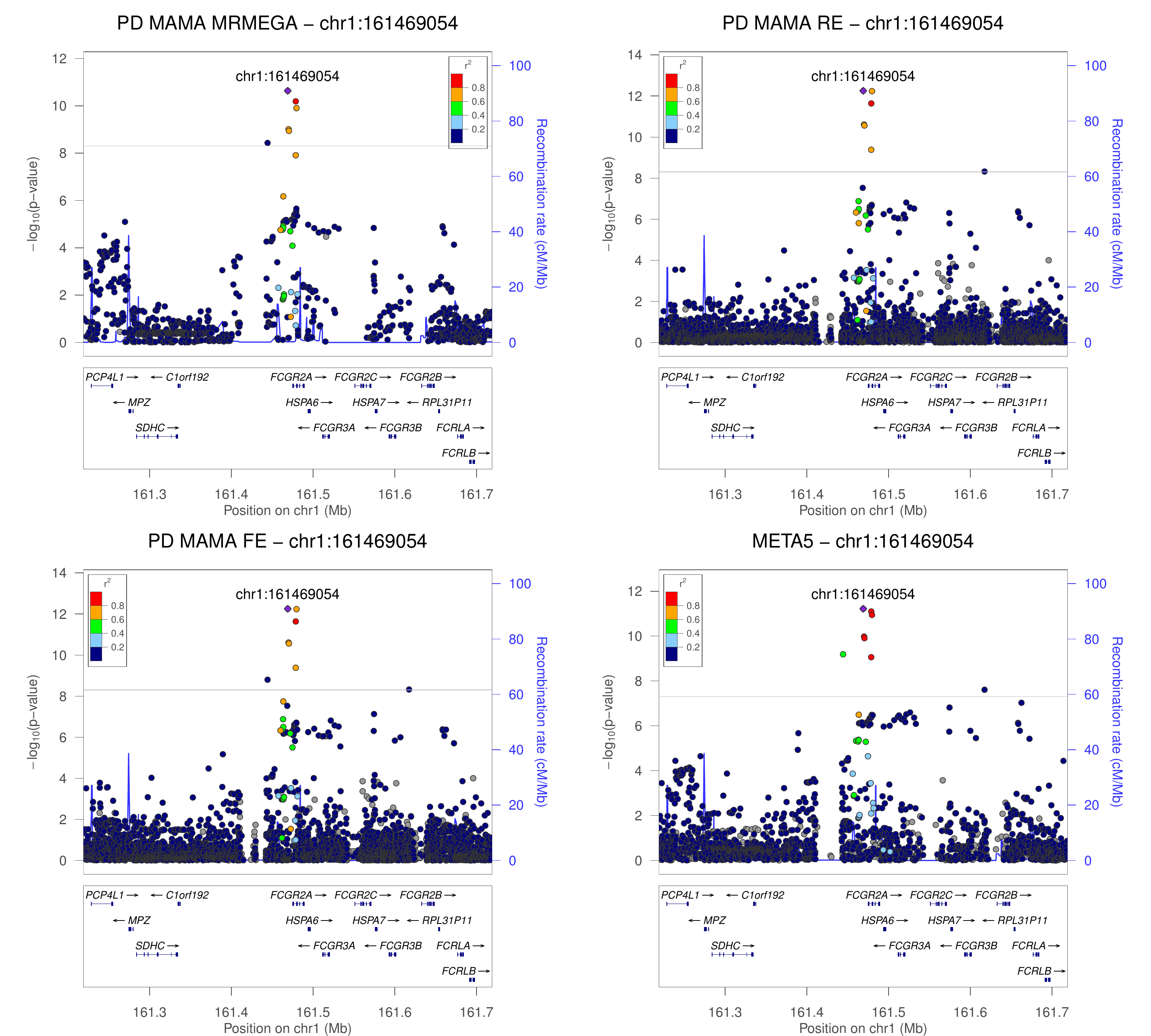

Supplement: Supplementary file 5 — This includes LocusZoom plots of all known European loci as well as novel loci. Each file contains four LocusZoom plots: PD MAMA MR-MEGA/RE/FE/ (MR-MEGA/random-effect/fixed-effect) and META5 (European-only meta-analysis from Nalls et al. 1). [file 41588_2023_1584_MOESM5_ESM.zip › LocusZoom plots of known EUR risk variants/chr1_161219054-161719054.png]

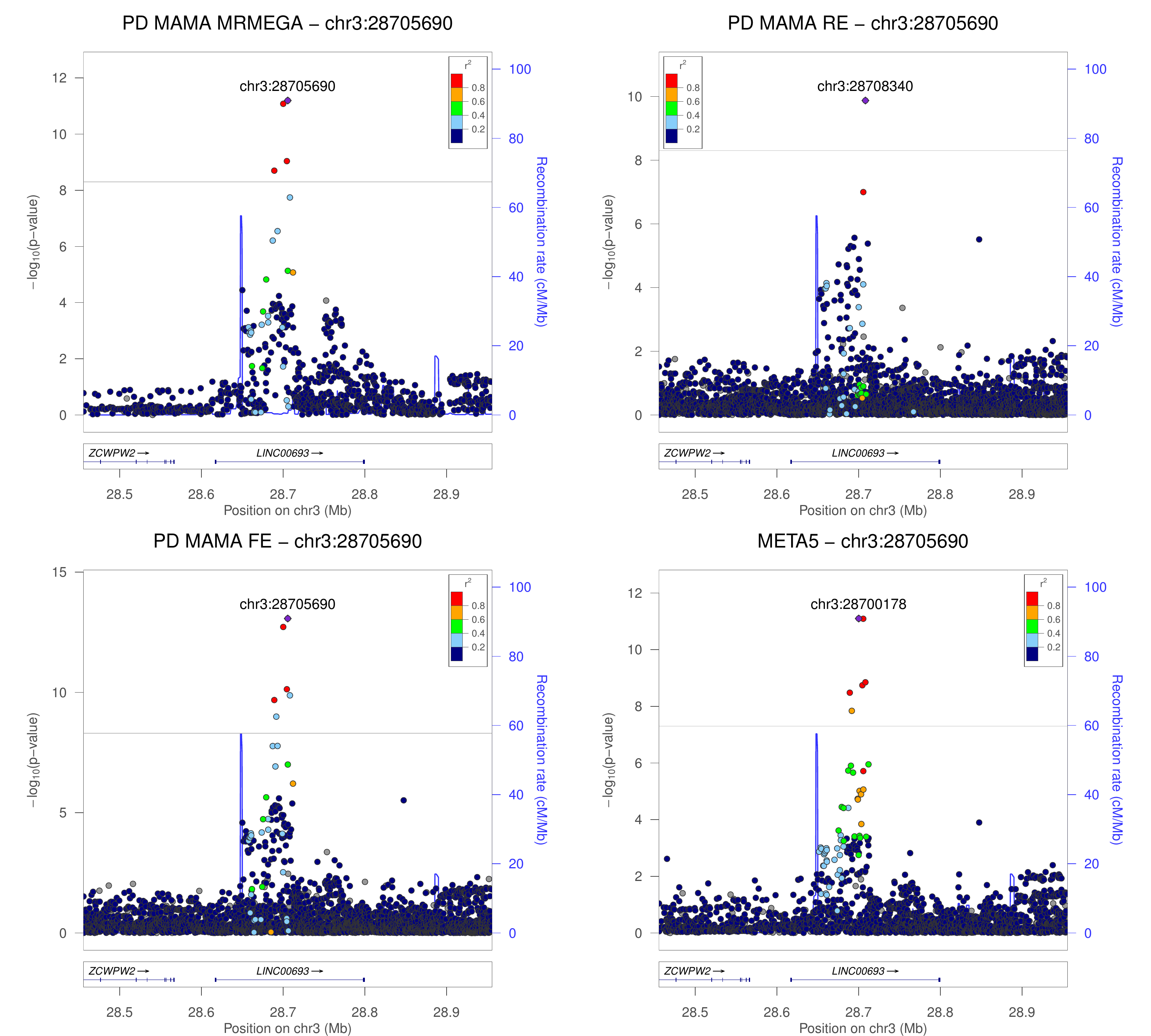

Supplement: Supplementary file 5 — This includes LocusZoom plots of all known European loci as well as novel loci. Each file contains four LocusZoom plots: PD MAMA MR-MEGA/RE/FE/ (MR-MEGA/random-effect/fixed-effect) and META5 (European-only meta-analysis from Nalls et al. 1). [file 41588_2023_1584_MOESM5_ESM.zip › LocusZoom plots of known EUR risk variants/chr3_28455690-28955690.png]

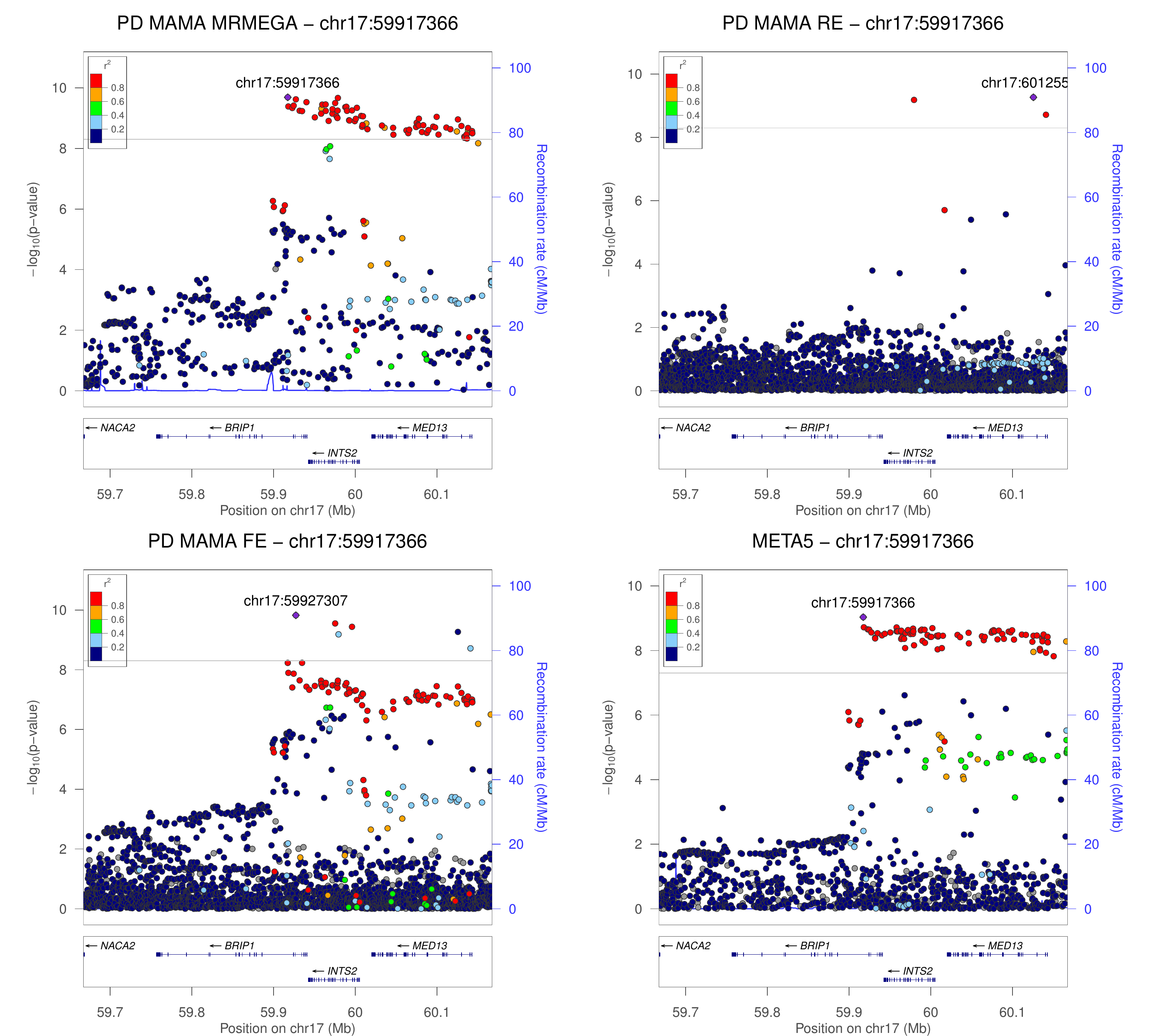

Supplement: Supplementary file 5 — This includes LocusZoom plots of all known European loci as well as novel loci. Each file contains four LocusZoom plots: PD MAMA MR-MEGA/RE/FE/ (MR-MEGA/random-effect/fixed-effect) and META5 (European-only meta-analysis from Nalls et al. 1). [file 41588_2023_1584_MOESM5_ESM.zip › LocusZoom plots of known EUR risk variants/chr17_59667366-60167366.png]

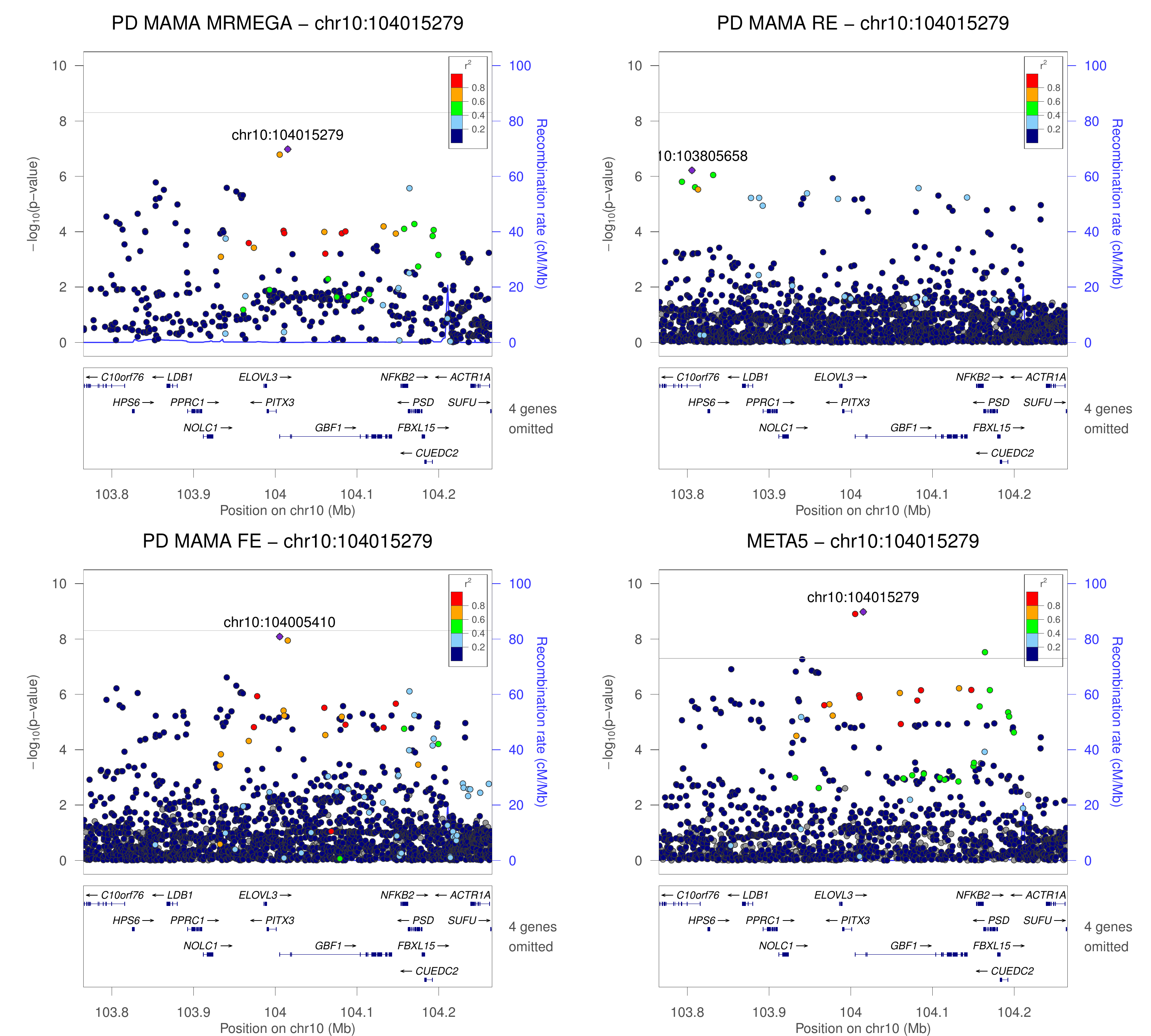

Supplement: Supplementary file 5 — This includes LocusZoom plots of all known European loci as well as novel loci. Each file contains four LocusZoom plots: PD MAMA MR-MEGA/RE/FE/ (MR-MEGA/random-effect/fixed-effect) and META5 (European-only meta-analysis from Nalls et al. 1). [file 41588_2023_1584_MOESM5_ESM.zip › LocusZoom plots of known EUR risk variants/chr10_103765279-104265279.png]

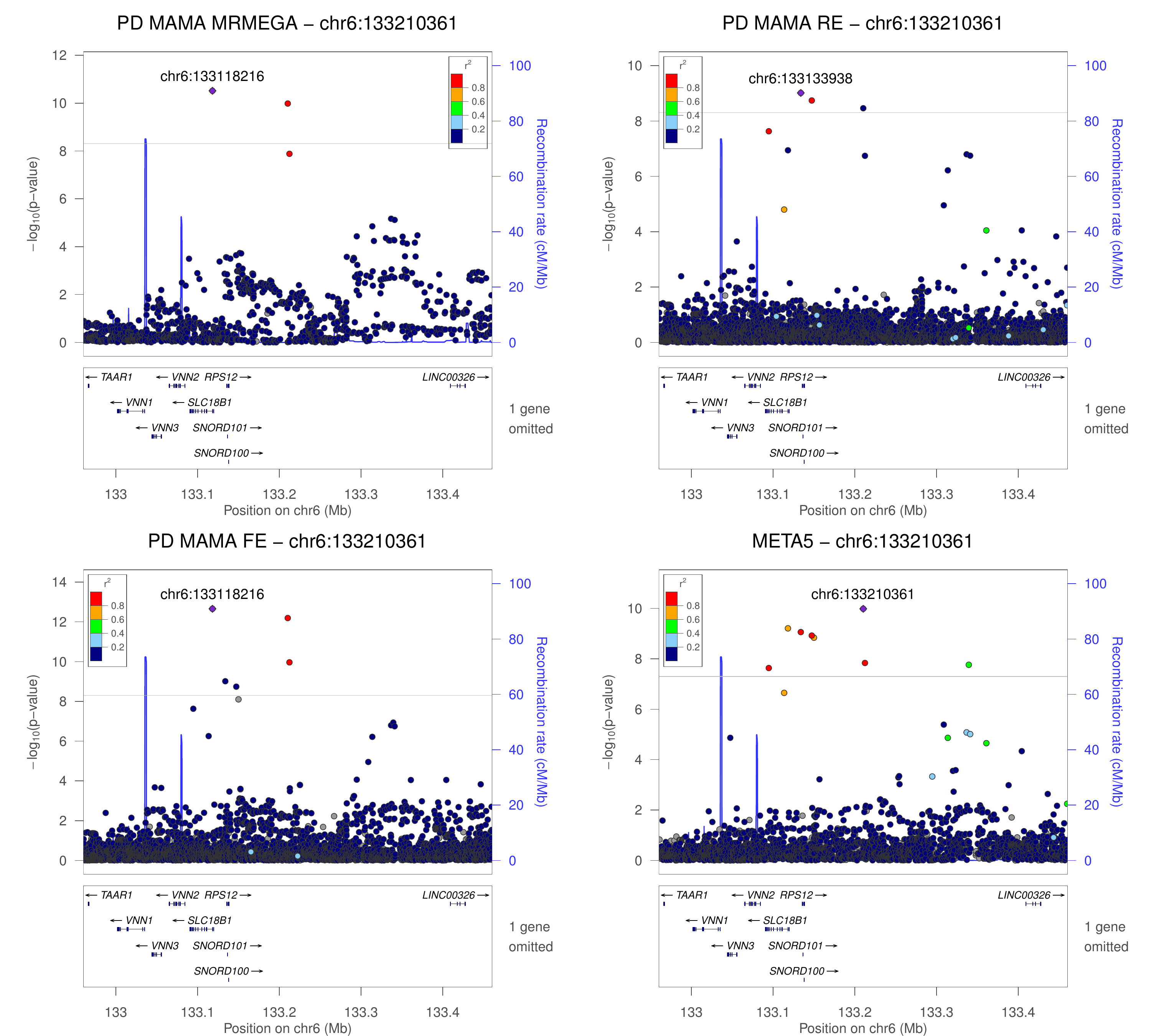

Supplement: Supplementary file 5 — This includes LocusZoom plots of all known European loci as well as novel loci. Each file contains four LocusZoom plots: PD MAMA MR-MEGA/RE/FE/ (MR-MEGA/random-effect/fixed-effect) and META5 (European-only meta-analysis from Nalls et al. 1). [file 41588_2023_1584_MOESM5_ESM.zip › LocusZoom plots of known EUR risk variants/chr6_132960361-133460361.png]

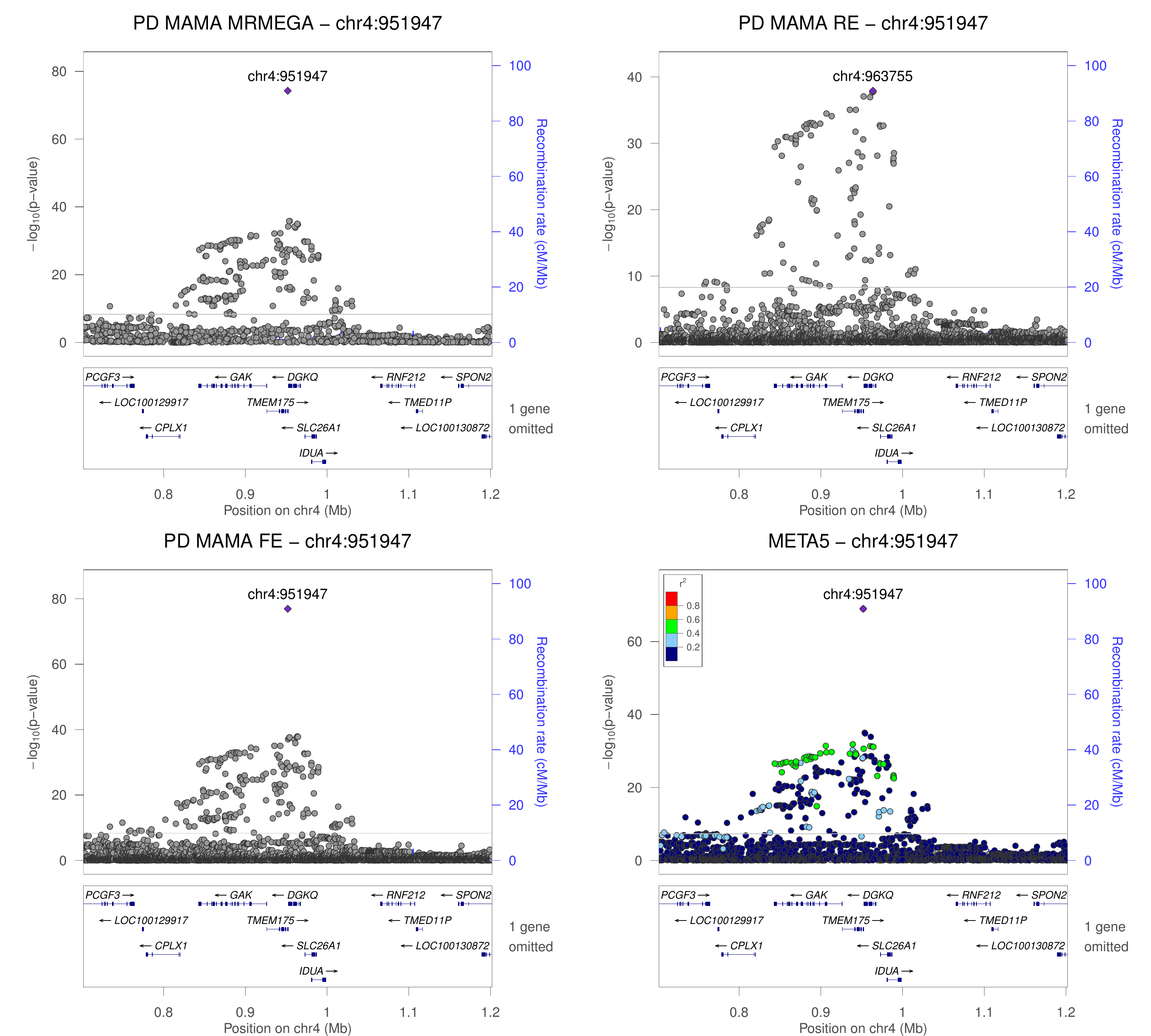

Supplement: Supplementary file 5 — This includes LocusZoom plots of all known European loci as well as novel loci. Each file contains four LocusZoom plots: PD MAMA MR-MEGA/RE/FE/ (MR-MEGA/random-effect/fixed-effect) and META5 (European-only meta-analysis from Nalls et al. 1). [file 41588_2023_1584_MOESM5_ESM.zip › LocusZoom plots of known EUR risk variants/chr4_701947-1201947.png]

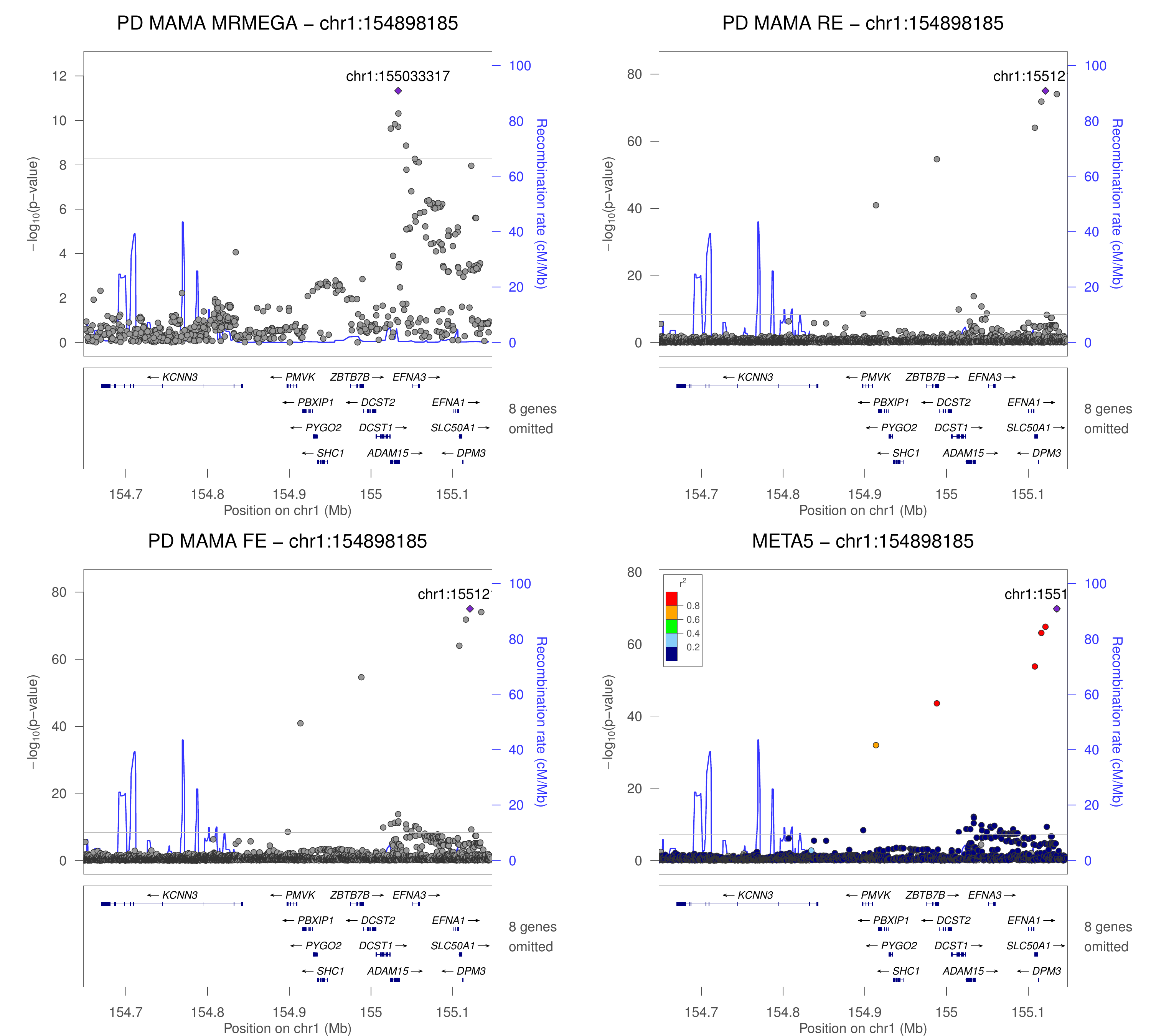

Supplement: Supplementary file 5 — This includes LocusZoom plots of all known European loci as well as novel loci. Each file contains four LocusZoom plots: PD MAMA MR-MEGA/RE/FE/ (MR-MEGA/random-effect/fixed-effect) and META5 (European-only meta-analysis from Nalls et al. 1). [file 41588_2023_1584_MOESM5_ESM.zip › LocusZoom plots of known EUR risk variants/chr1_154648185-155148185.png]

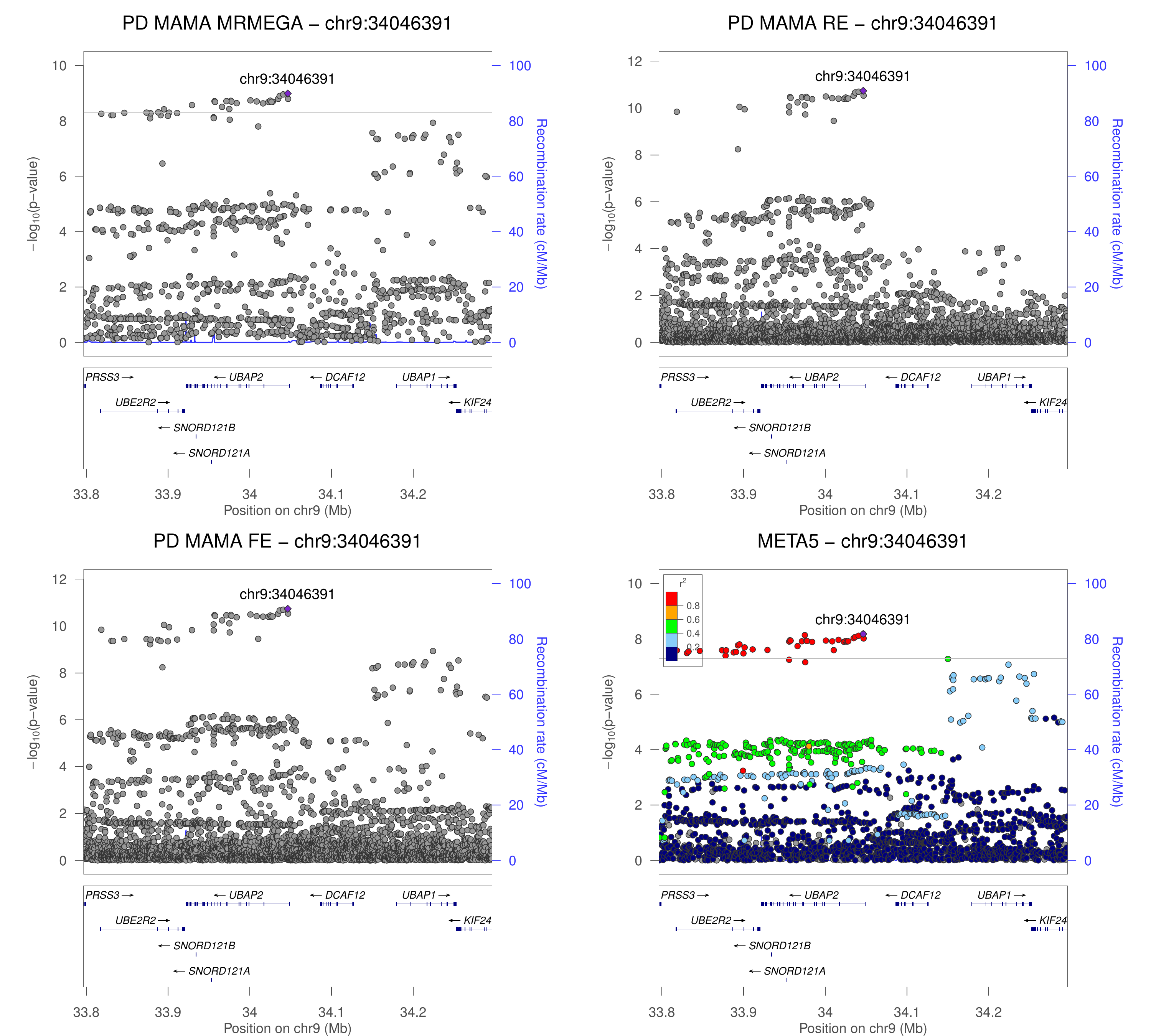

Supplement: Supplementary file 5 — This includes LocusZoom plots of all known European loci as well as novel loci. Each file contains four LocusZoom plots: PD MAMA MR-MEGA/RE/FE/ (MR-MEGA/random-effect/fixed-effect) and META5 (European-only meta-analysis from Nalls et al. 1). [file 41588_2023_1584_MOESM5_ESM.zip › LocusZoom plots of known EUR risk variants/chr9_33796391-34296391.png]

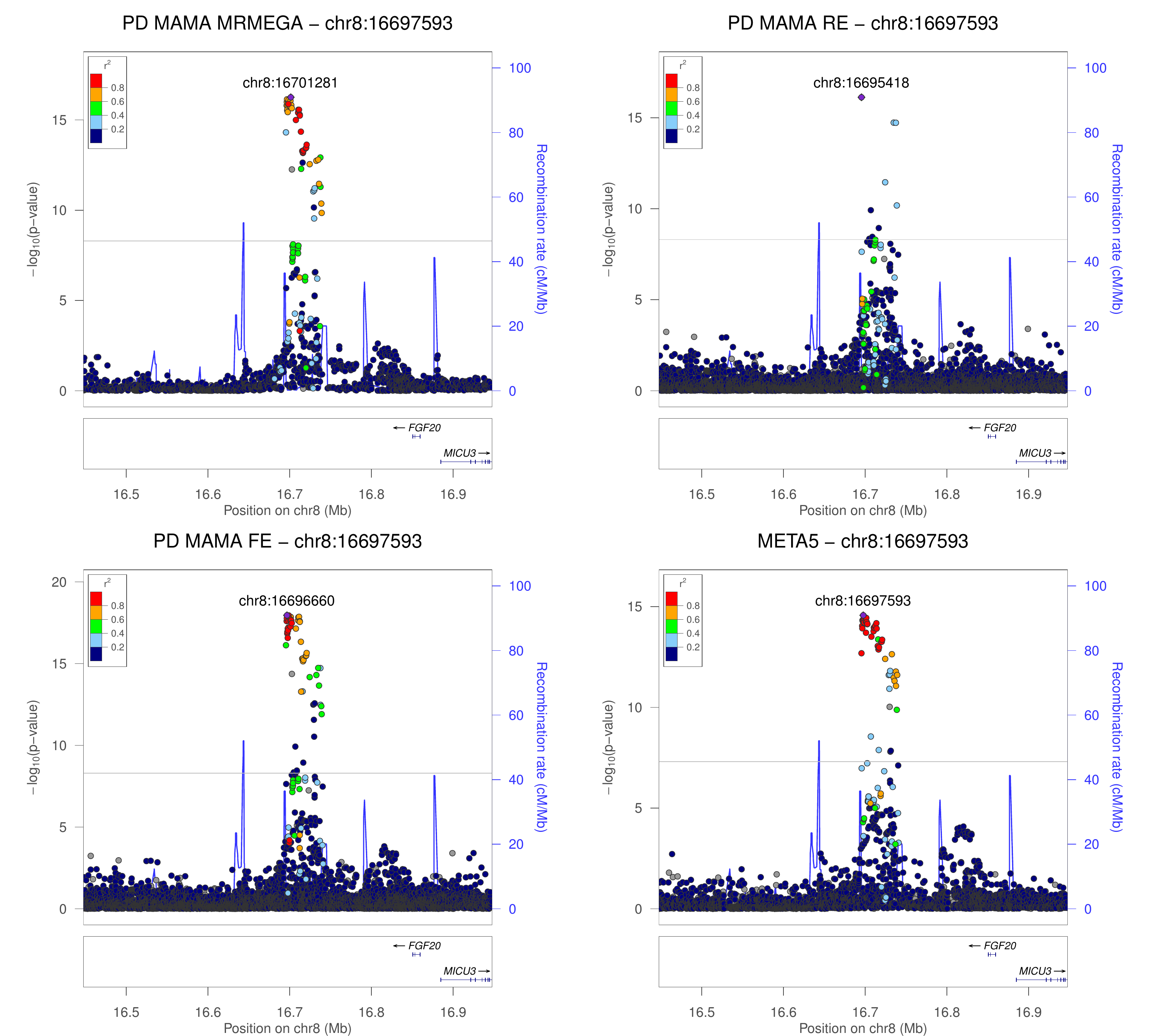

Supplement: Supplementary file 5 — This includes LocusZoom plots of all known European loci as well as novel loci. Each file contains four LocusZoom plots: PD MAMA MR-MEGA/RE/FE/ (MR-MEGA/random-effect/fixed-effect) and META5 (European-only meta-analysis from Nalls et al. 1). [file 41588_2023_1584_MOESM5_ESM.zip › LocusZoom plots of known EUR risk variants/chr8_16447593-16947593.png]

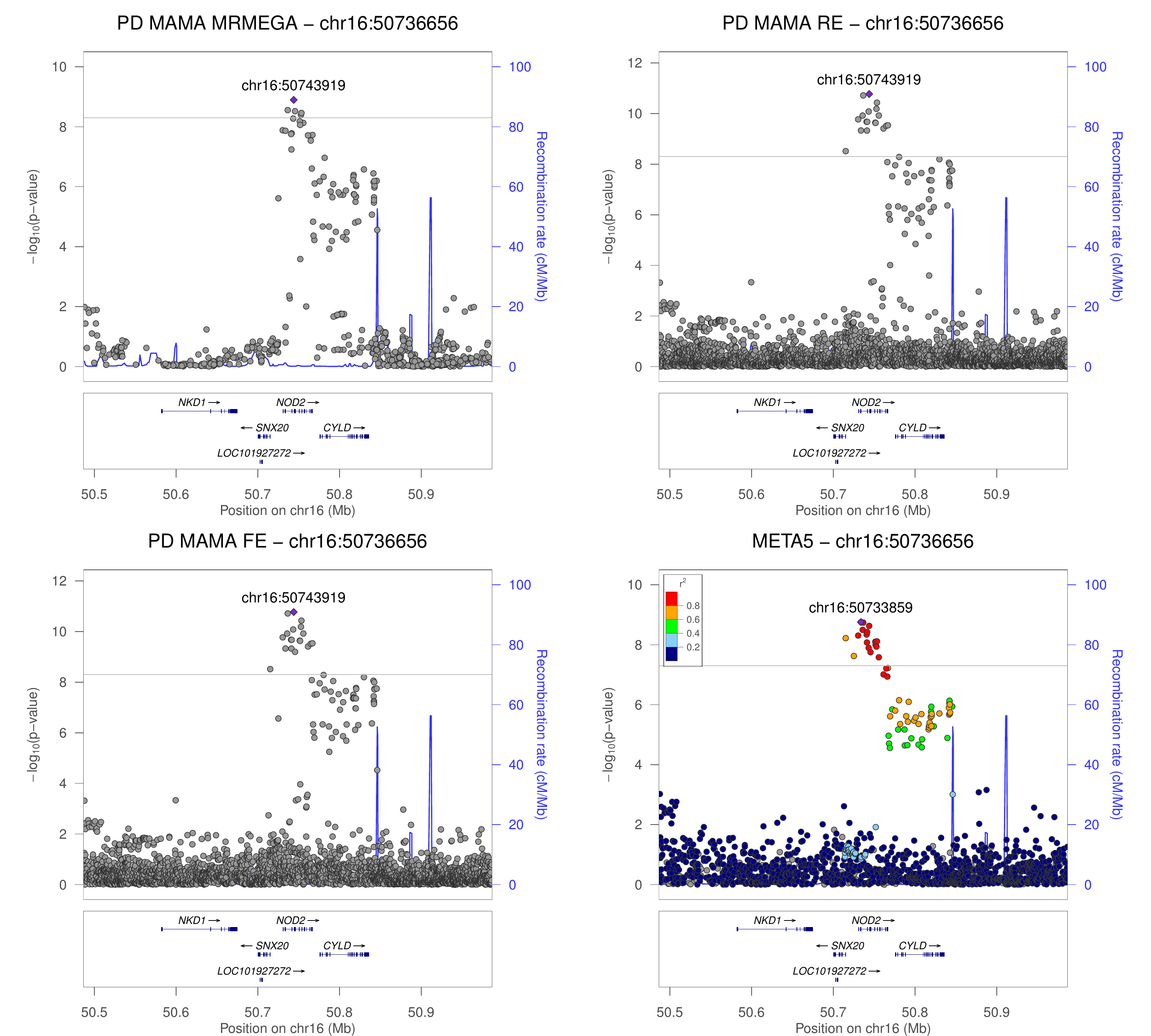

Supplement: Supplementary file 5 — This includes LocusZoom plots of all known European loci as well as novel loci. Each file contains four LocusZoom plots: PD MAMA MR-MEGA/RE/FE/ (MR-MEGA/random-effect/fixed-effect) and META5 (European-only meta-analysis from Nalls et al. 1). [file 41588_2023_1584_MOESM5_ESM.zip › LocusZoom plots of known EUR risk variants/chr16_50486656-50986656.png]

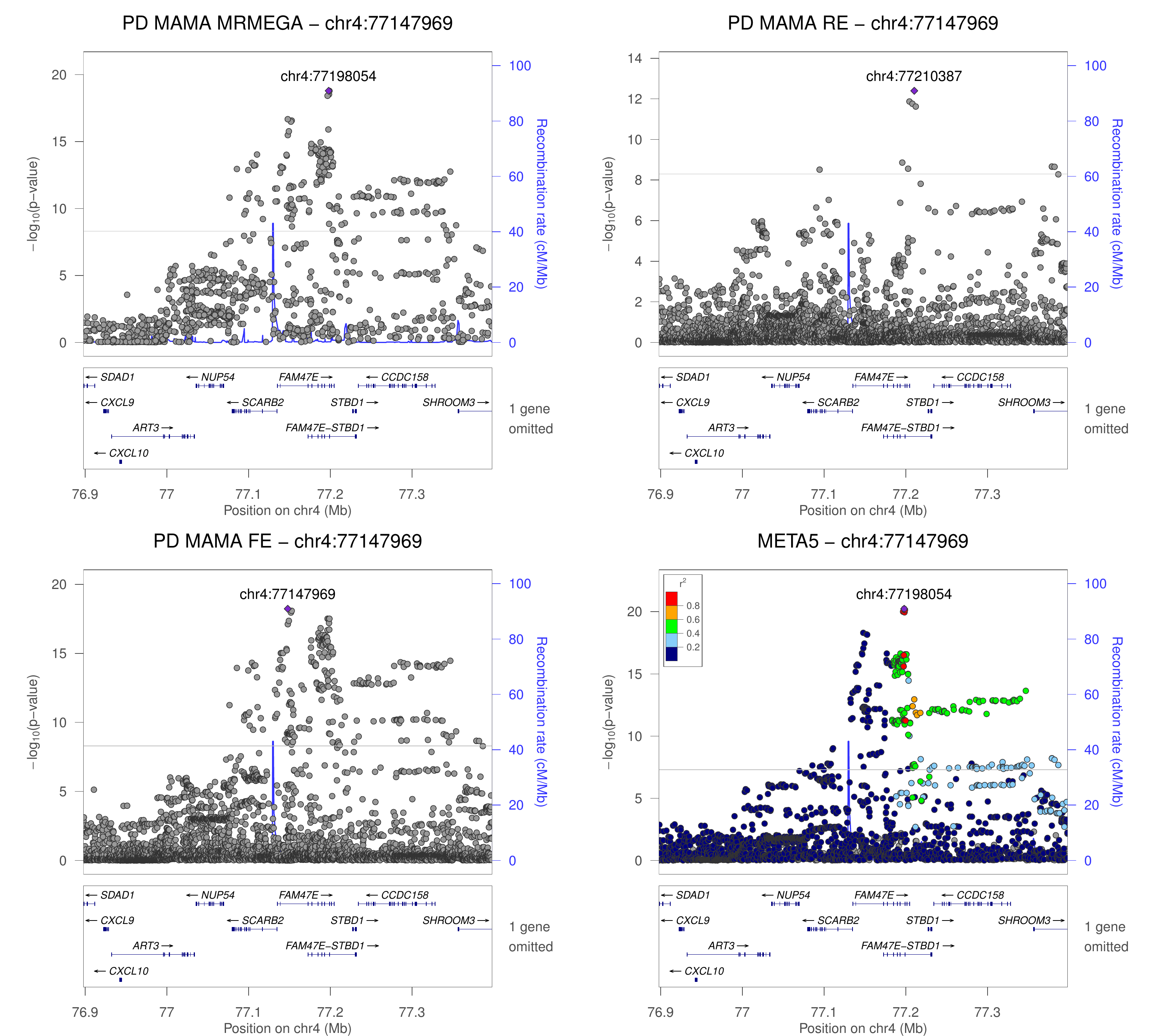

Supplement: Supplementary file 5 — This includes LocusZoom plots of all known European loci as well as novel loci. Each file contains four LocusZoom plots: PD MAMA MR-MEGA/RE/FE/ (MR-MEGA/random-effect/fixed-effect) and META5 (European-only meta-analysis from Nalls et al. 1). [file 41588_2023_1584_MOESM5_ESM.zip › LocusZoom plots of known EUR risk variants/chr4_76897969-77397969.png]

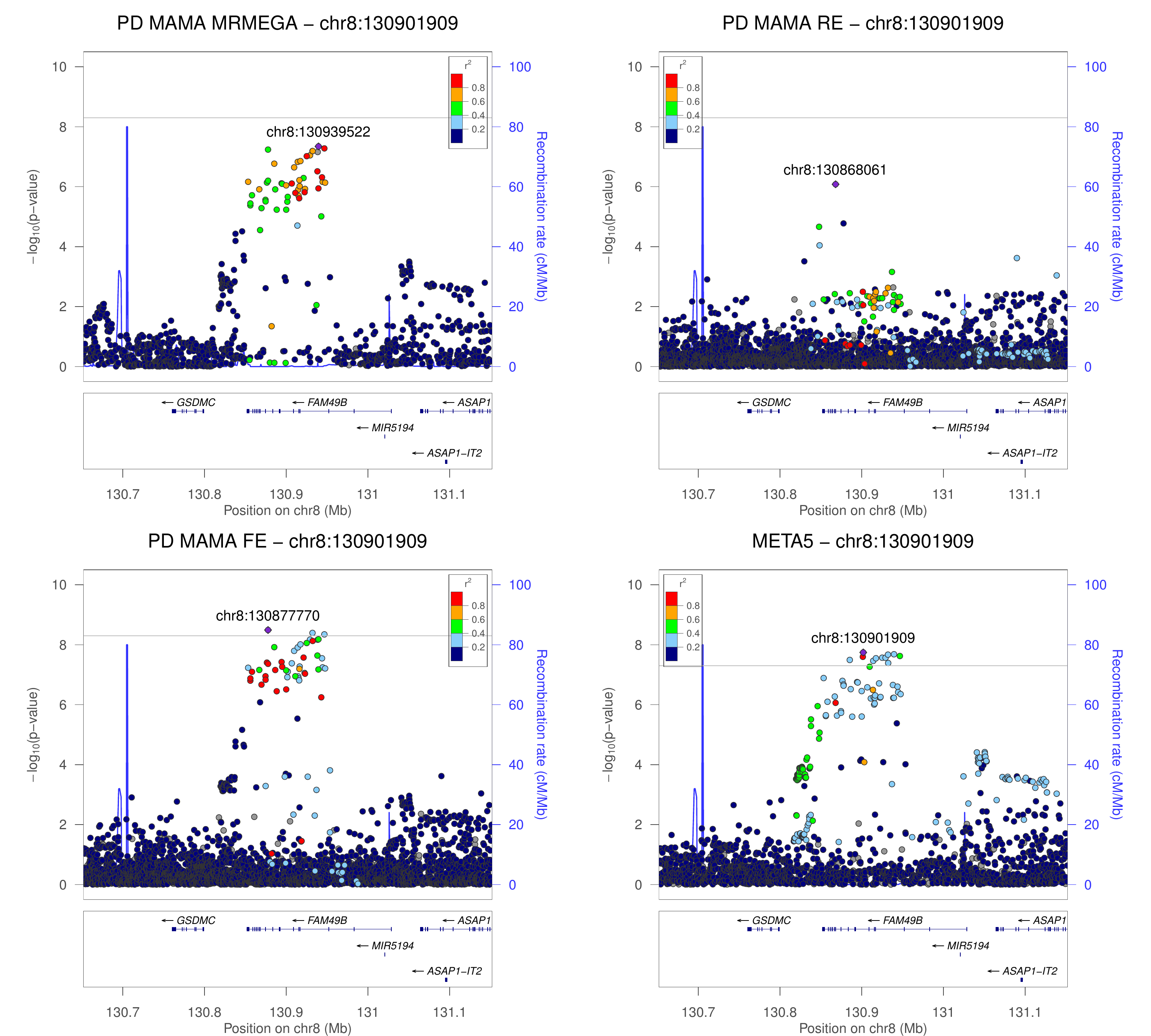

Supplement: Supplementary file 5 — This includes LocusZoom plots of all known European loci as well as novel loci. Each file contains four LocusZoom plots: PD MAMA MR-MEGA/RE/FE/ (MR-MEGA/random-effect/fixed-effect) and META5 (European-only meta-analysis from Nalls et al. 1). [file 41588_2023_1584_MOESM5_ESM.zip › LocusZoom plots of known EUR risk variants/chr8_130651909-131151909.png]

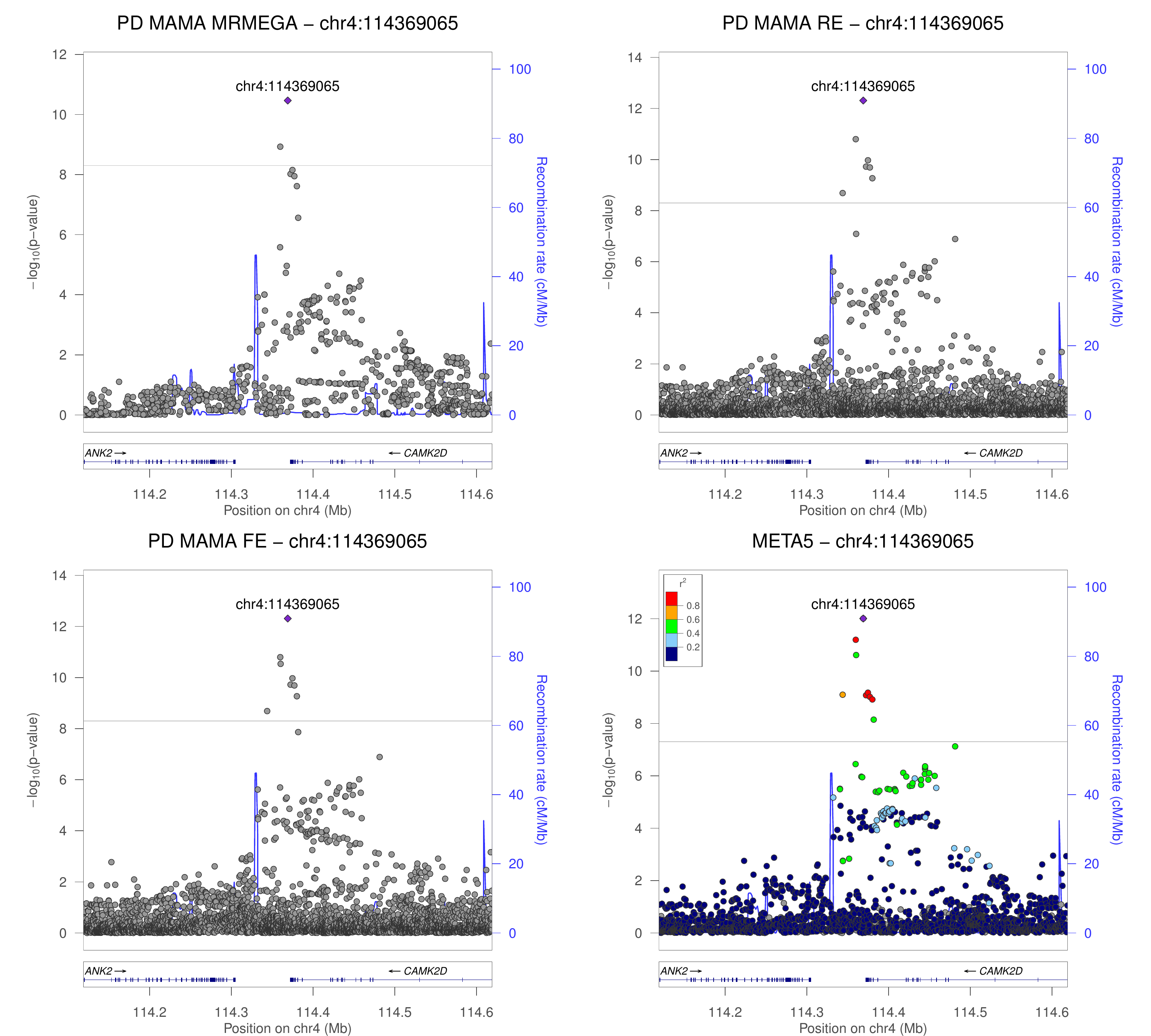

Supplement: Supplementary file 5 — This includes LocusZoom plots of all known European loci as well as novel loci. Each file contains four LocusZoom plots: PD MAMA MR-MEGA/RE/FE/ (MR-MEGA/random-effect/fixed-effect) and META5 (European-only meta-analysis from Nalls et al. 1). [file 41588_2023_1584_MOESM5_ESM.zip › LocusZoom plots of known EUR risk variants/chr4_114119065-114619065.png]

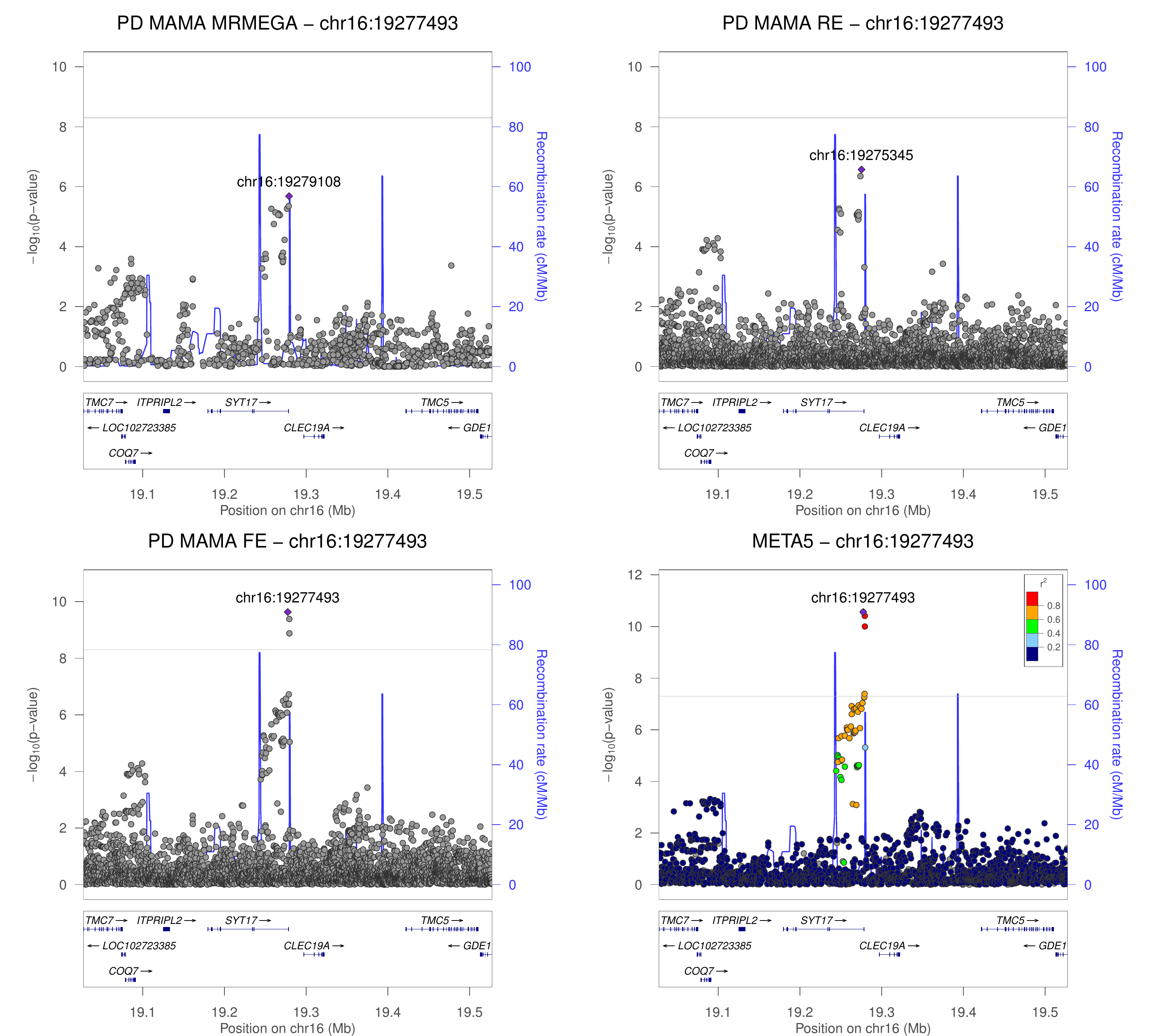

Supplement: Supplementary file 5 — This includes LocusZoom plots of all known European loci as well as novel loci. Each file contains four LocusZoom plots: PD MAMA MR-MEGA/RE/FE/ (MR-MEGA/random-effect/fixed-effect) and META5 (European-only meta-analysis from Nalls et al. 1). [file 41588_2023_1584_MOESM5_ESM.zip › LocusZoom plots of known EUR risk variants/chr16_19027493-19527493.png]

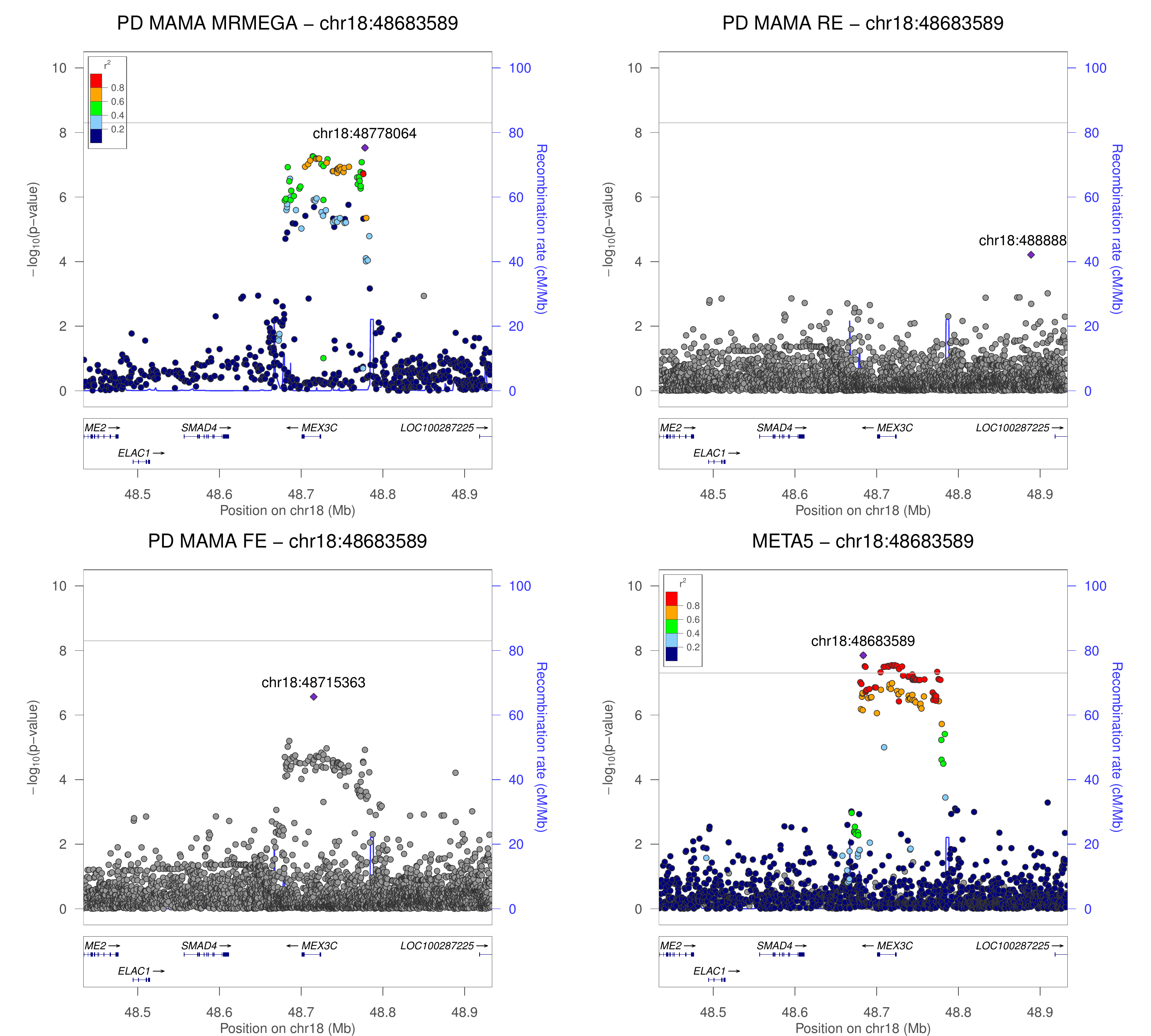

Supplement: Supplementary file 5 — This includes LocusZoom plots of all known European loci as well as novel loci. Each file contains four LocusZoom plots: PD MAMA MR-MEGA/RE/FE/ (MR-MEGA/random-effect/fixed-effect) and META5 (European-only meta-analysis from Nalls et al. 1). [file 41588_2023_1584_MOESM5_ESM.zip › LocusZoom plots of known EUR risk variants/chr18_48433589-48933589.png]

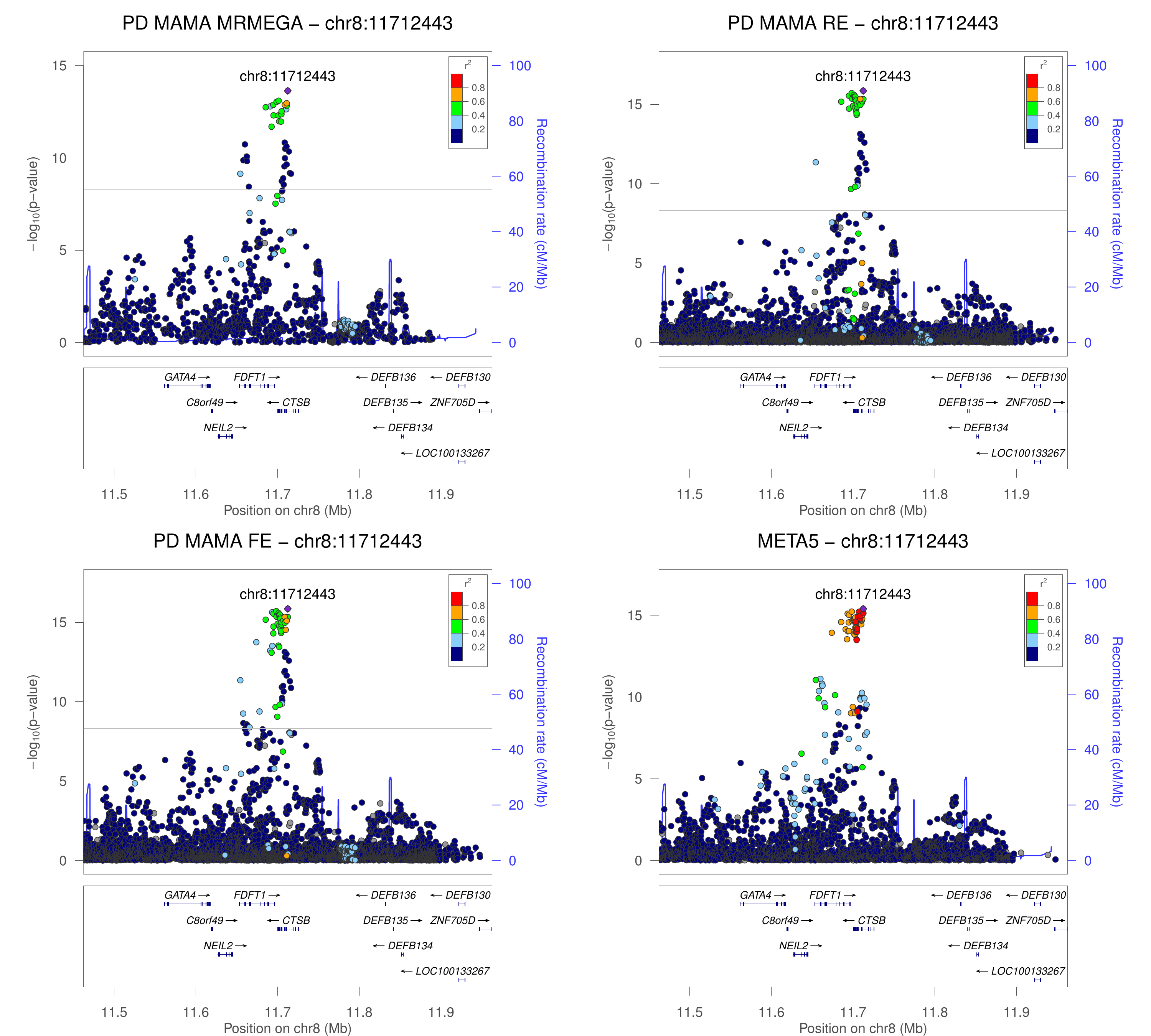

Supplement: Supplementary file 5 — This includes LocusZoom plots of all known European loci as well as novel loci. Each file contains four LocusZoom plots: PD MAMA MR-MEGA/RE/FE/ (MR-MEGA/random-effect/fixed-effect) and META5 (European-only meta-analysis from Nalls et al. 1). [file 41588_2023_1584_MOESM5_ESM.zip › LocusZoom plots of known EUR risk variants/chr8_11462443-11962443.png]

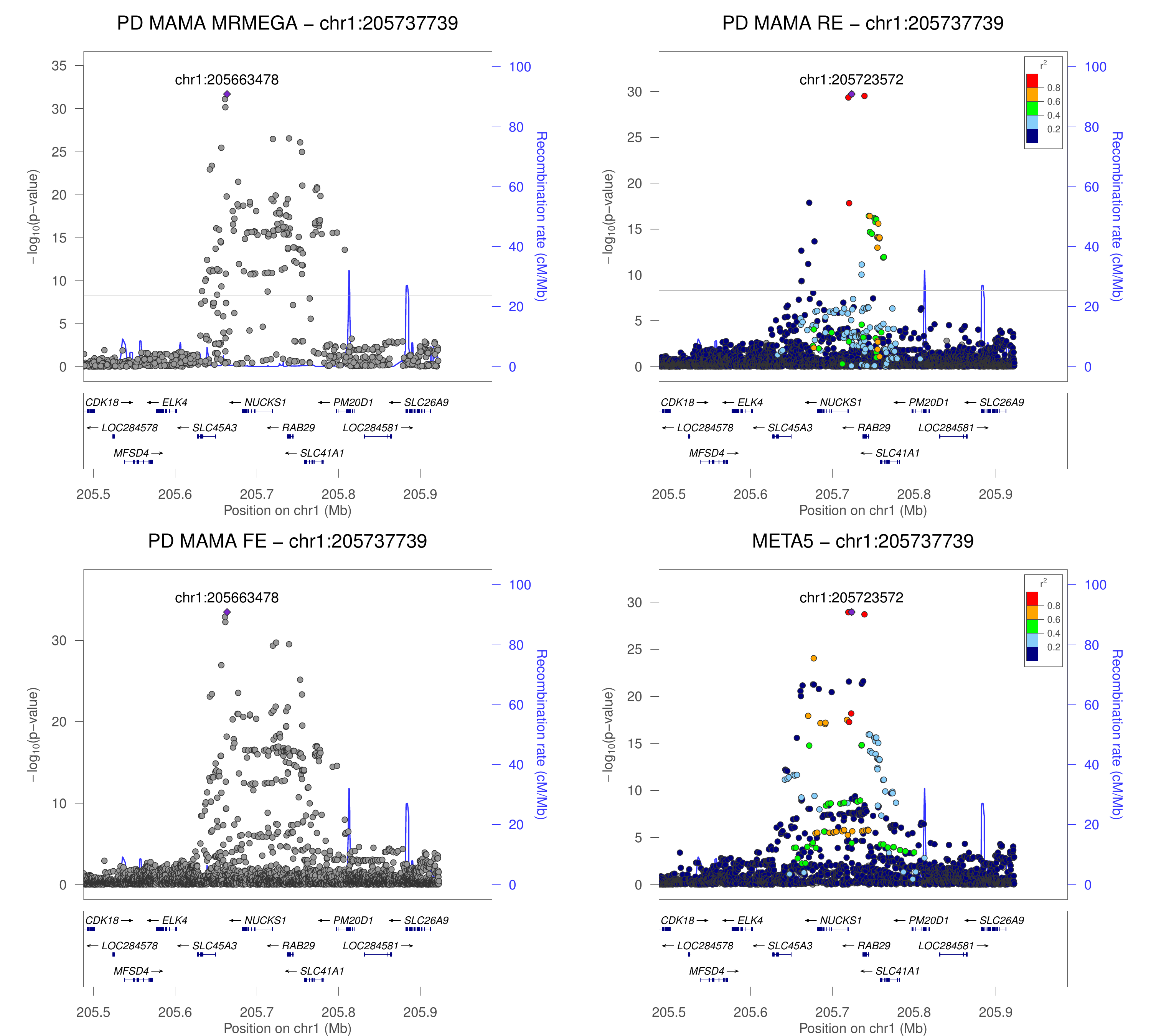

Supplement: Supplementary file 5 — This includes LocusZoom plots of all known European loci as well as novel loci. Each file contains four LocusZoom plots: PD MAMA MR-MEGA/RE/FE/ (MR-MEGA/random-effect/fixed-effect) and META5 (European-only meta-analysis from Nalls et al. 1). [file 41588_2023_1584_MOESM5_ESM.zip › LocusZoom plots of known EUR risk variants/chr1_205487739-205987739.png]

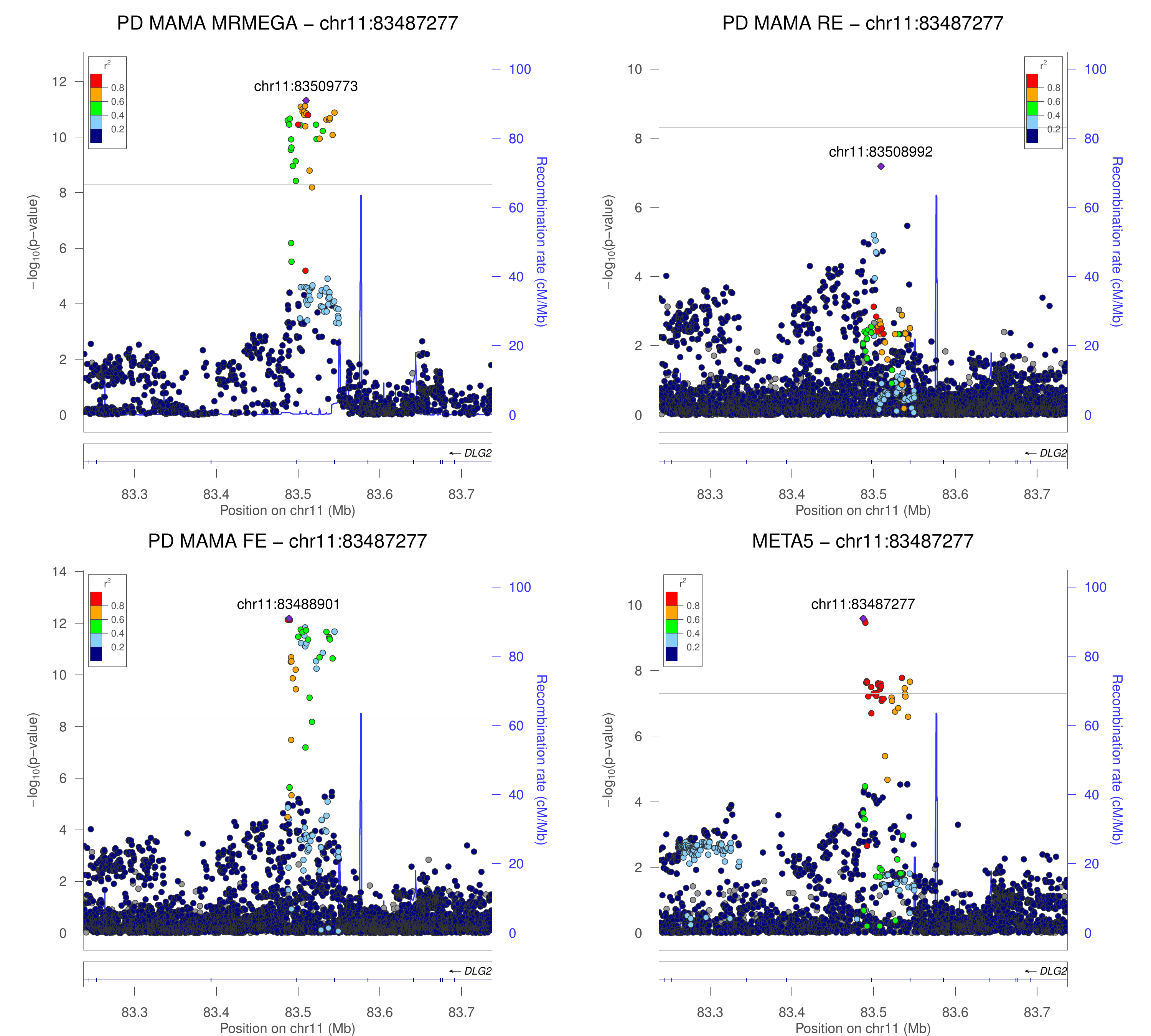

Supplement: Supplementary file 5 — This includes LocusZoom plots of all known European loci as well as novel loci. Each file contains four LocusZoom plots: PD MAMA MR-MEGA/RE/FE/ (MR-MEGA/random-effect/fixed-effect) and META5 (European-only meta-analysis from Nalls et al. 1). [file 41588_2023_1584_MOESM5_ESM.zip › LocusZoom plots of known EUR risk variants/chr11_83237277-83737277.png]

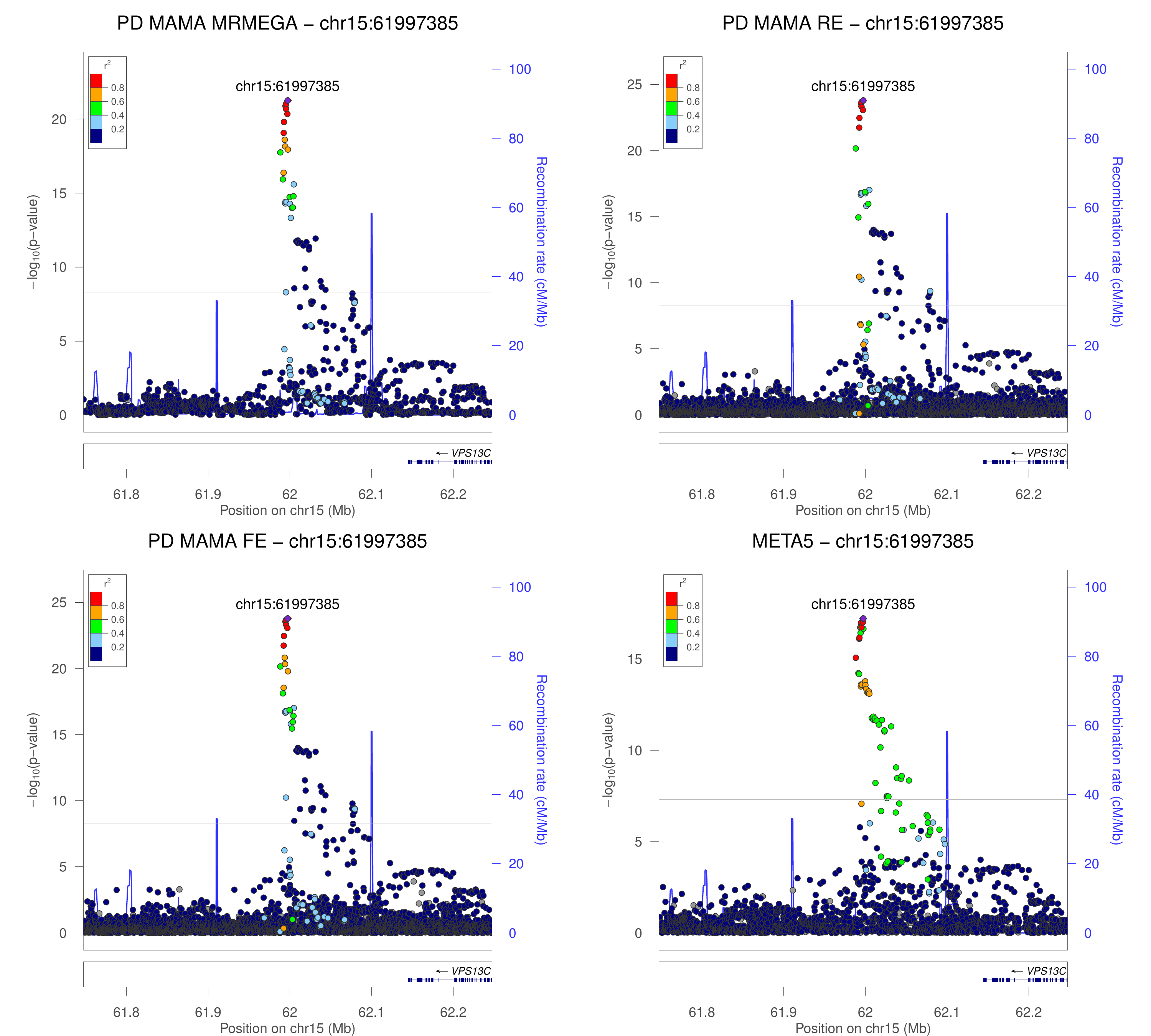

Supplement: Supplementary file 5 — This includes LocusZoom plots of all known European loci as well as novel loci. Each file contains four LocusZoom plots: PD MAMA MR-MEGA/RE/FE/ (MR-MEGA/random-effect/fixed-effect) and META5 (European-only meta-analysis from Nalls et al. 1). [file 41588_2023_1584_MOESM5_ESM.zip › LocusZoom plots of known EUR risk variants/chr15_61747385-62247385.png]

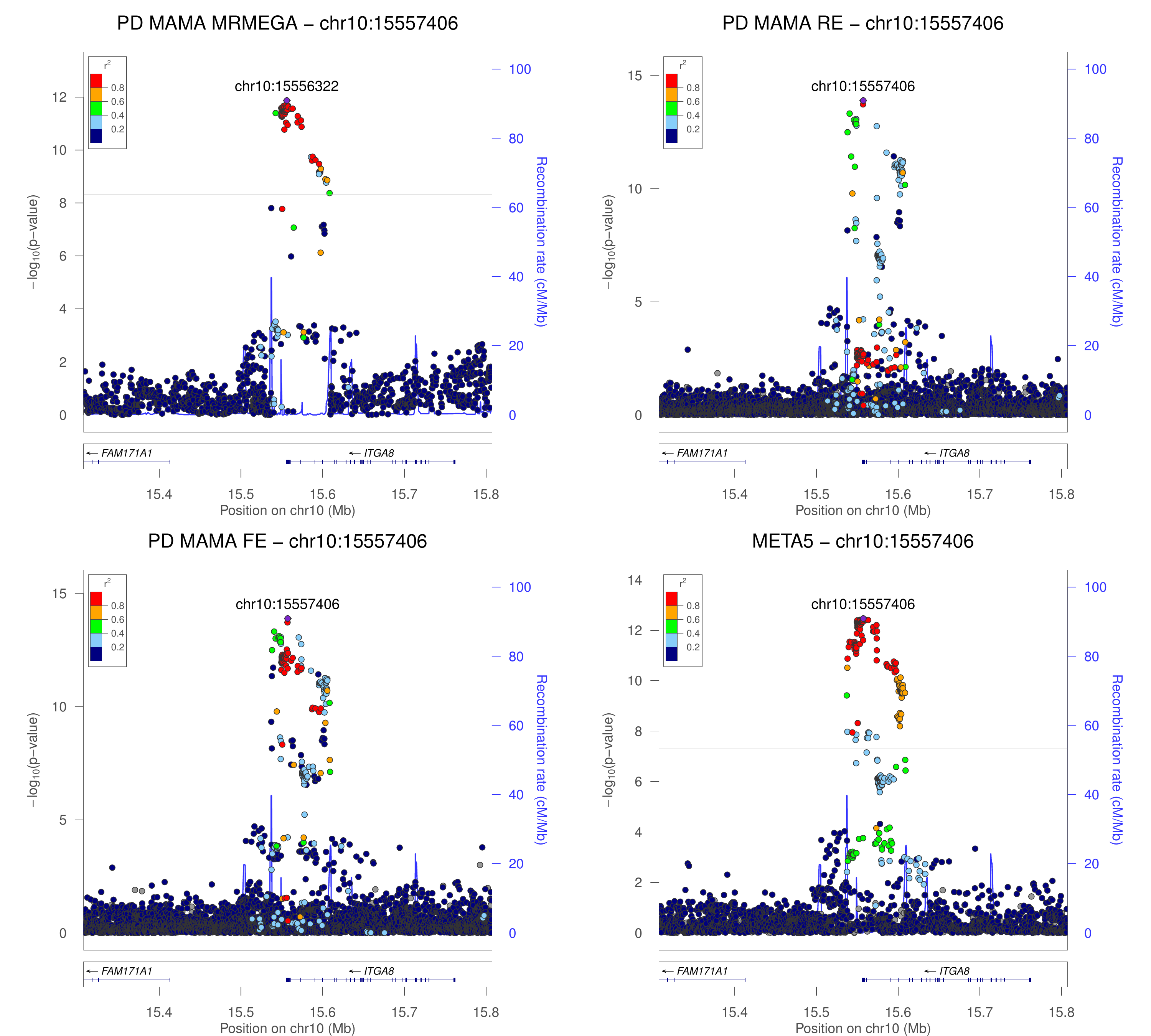

Supplement: Supplementary file 5 — This includes LocusZoom plots of all known European loci as well as novel loci. Each file contains four LocusZoom plots: PD MAMA MR-MEGA/RE/FE/ (MR-MEGA/random-effect/fixed-effect) and META5 (European-only meta-analysis from Nalls et al. 1). [file 41588_2023_1584_MOESM5_ESM.zip › LocusZoom plots of known EUR risk variants/chr10_15307406-15807406.png]

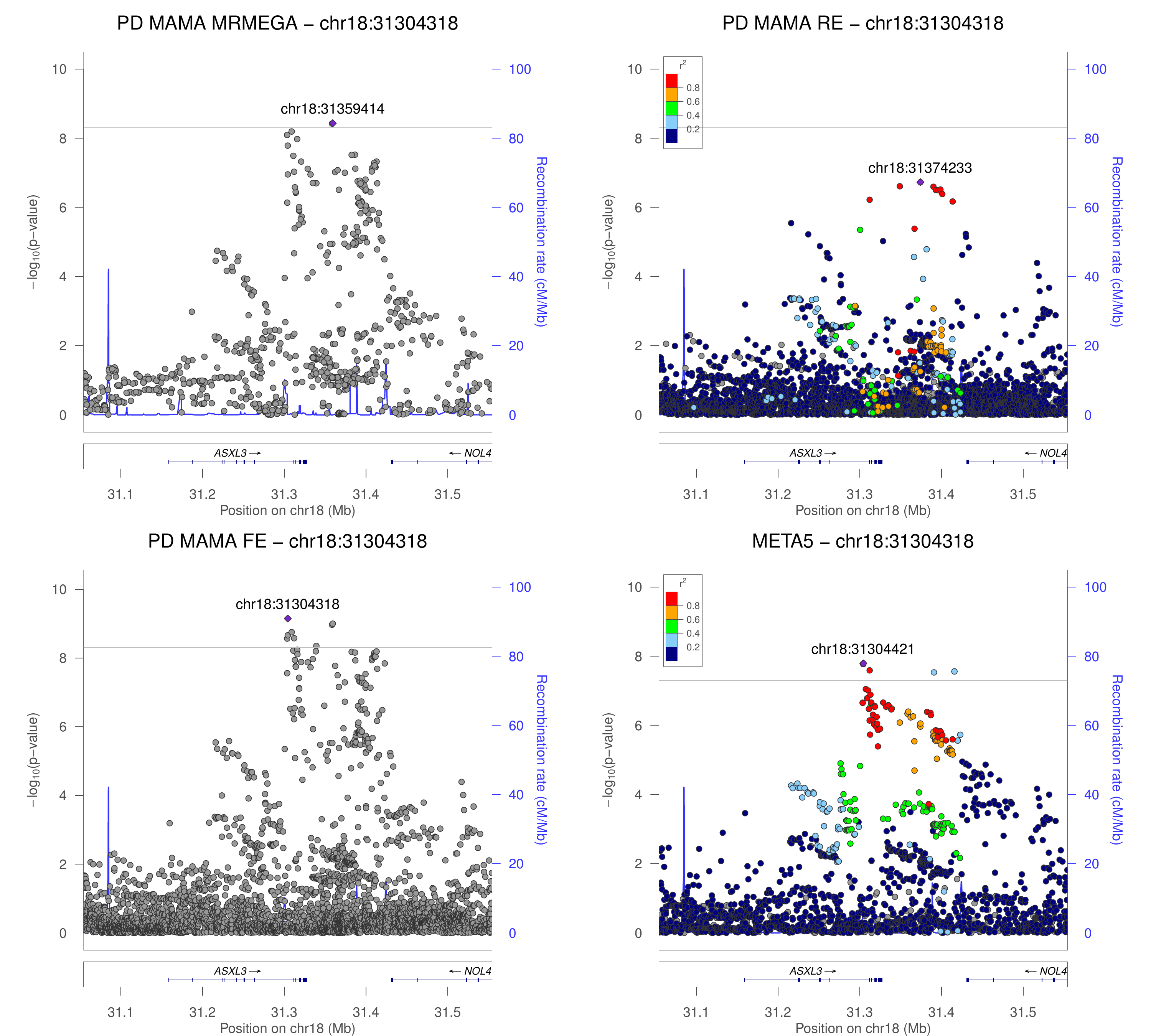

Supplement: Supplementary file 5 — This includes LocusZoom plots of all known European loci as well as novel loci. Each file contains four LocusZoom plots: PD MAMA MR-MEGA/RE/FE/ (MR-MEGA/random-effect/fixed-effect) and META5 (European-only meta-analysis from Nalls et al. 1). [file 41588_2023_1584_MOESM5_ESM.zip › LocusZoom plots of known EUR risk variants/chr18_31054318-31554318.png]

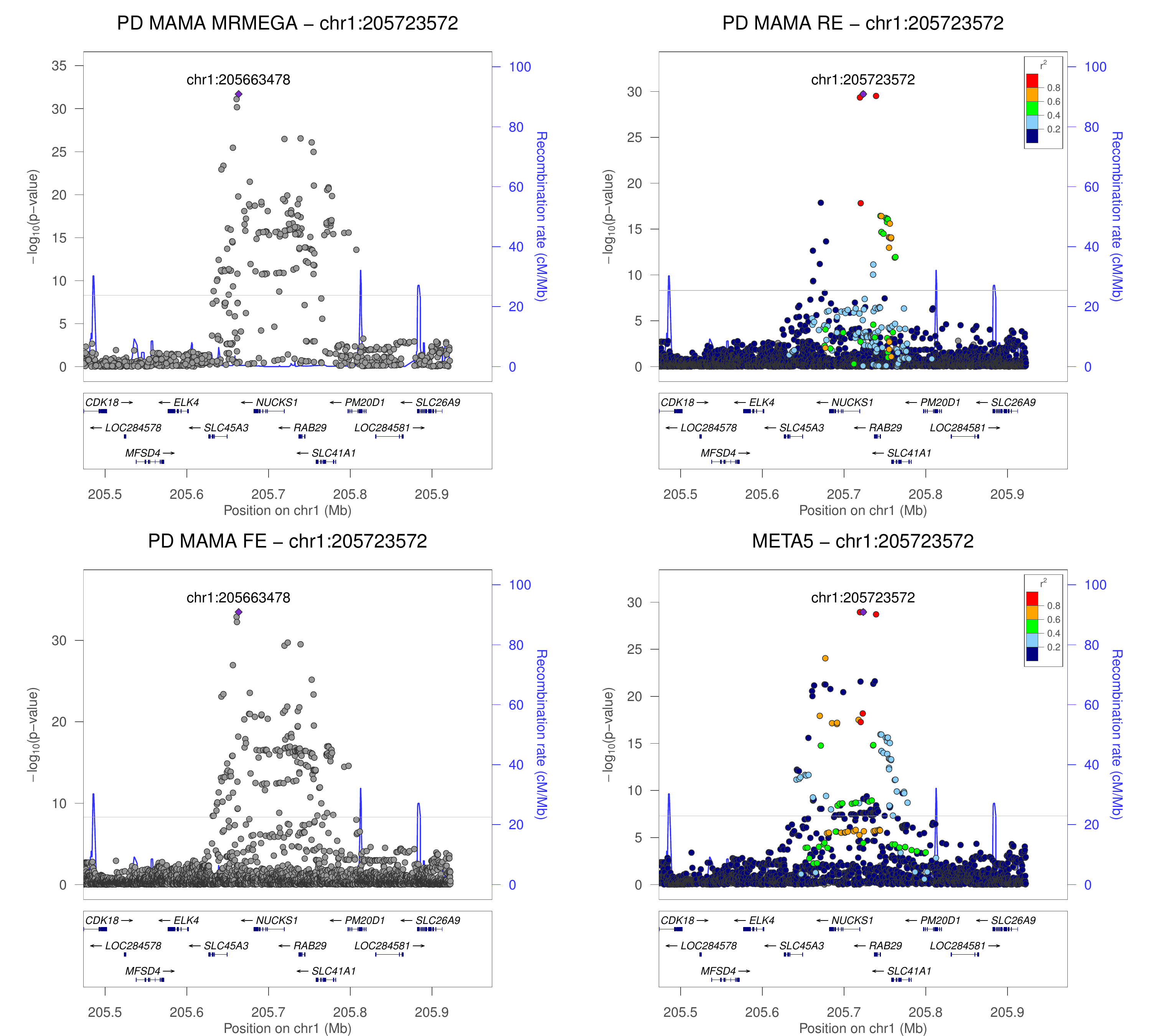

Supplement: Supplementary file 5 — This includes LocusZoom plots of all known European loci as well as novel loci. Each file contains four LocusZoom plots: PD MAMA MR-MEGA/RE/FE/ (MR-MEGA/random-effect/fixed-effect) and META5 (European-only meta-analysis from Nalls et al. 1). [file 41588_2023_1584_MOESM5_ESM.zip › LocusZoom plots of known EUR risk variants/chr1_205473572-205973572.png]

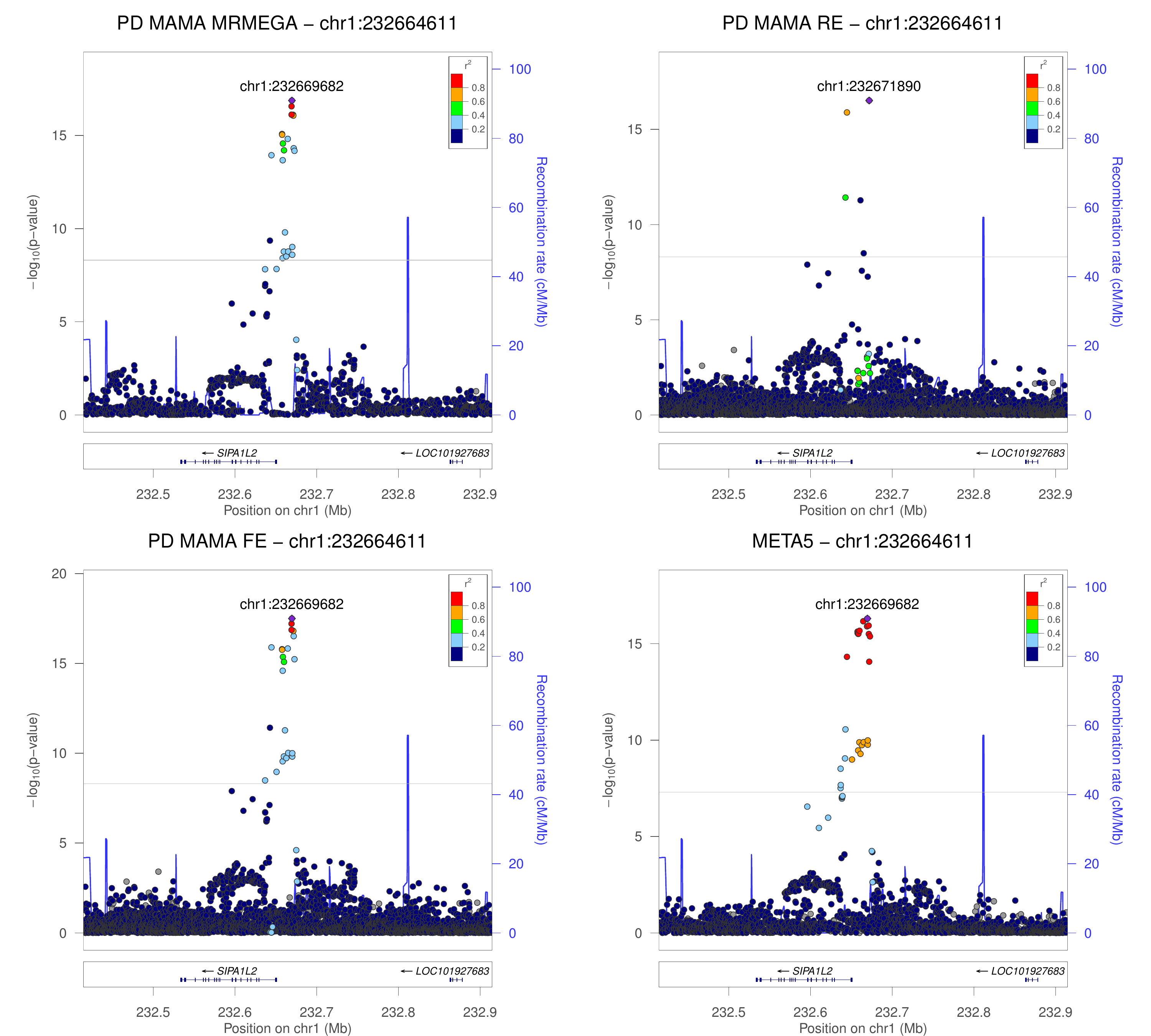

Supplement: Supplementary file 5 — This includes LocusZoom plots of all known European loci as well as novel loci. Each file contains four LocusZoom plots: PD MAMA MR-MEGA/RE/FE/ (MR-MEGA/random-effect/fixed-effect) and META5 (European-only meta-analysis from Nalls et al. 1). [file 41588_2023_1584_MOESM5_ESM.zip › LocusZoom plots of known EUR risk variants/chr1_232414611-232914611.png]

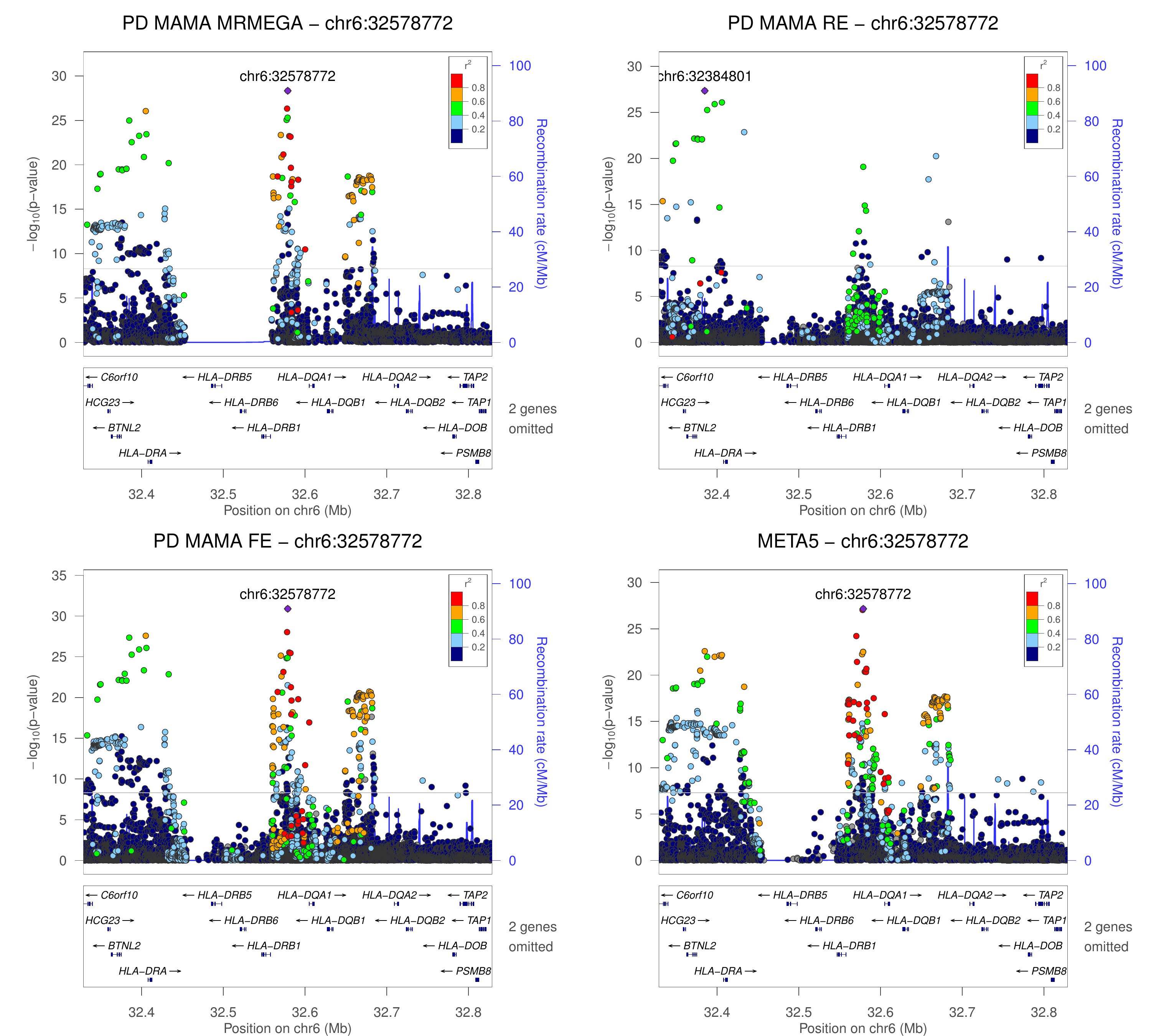

Supplement: Supplementary file 5 — This includes LocusZoom plots of all known European loci as well as novel loci. Each file contains four LocusZoom plots: PD MAMA MR-MEGA/RE/FE/ (MR-MEGA/random-effect/fixed-effect) and META5 (European-only meta-analysis from Nalls et al. 1). [file 41588_2023_1584_MOESM5_ESM.zip › LocusZoom plots of known EUR risk variants/chr6_32328772-32828772.png]

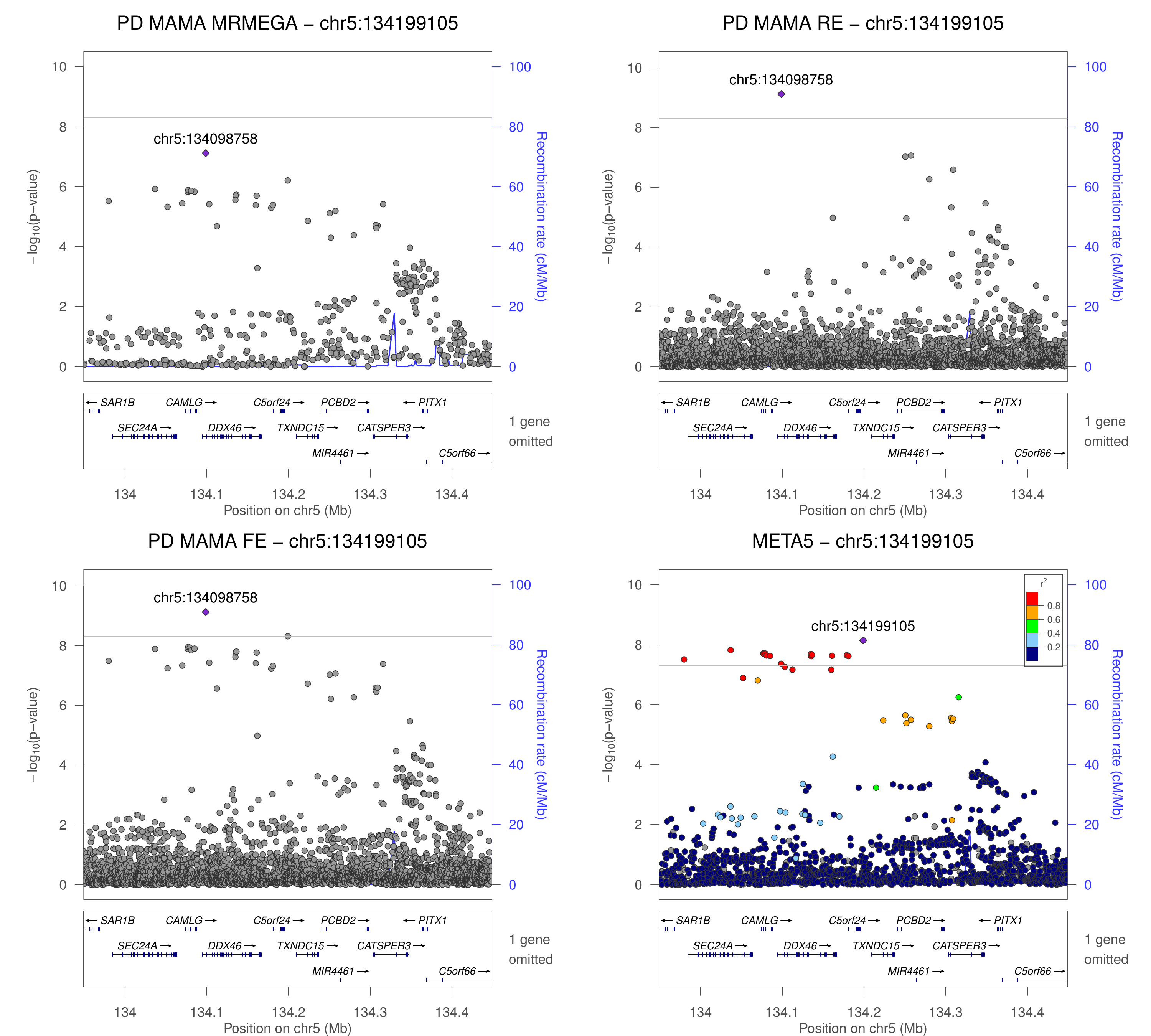

Supplement: Supplementary file 5 — This includes LocusZoom plots of all known European loci as well as novel loci. Each file contains four LocusZoom plots: PD MAMA MR-MEGA/RE/FE/ (MR-MEGA/random-effect/fixed-effect) and META5 (European-only meta-analysis from Nalls et al. 1). [file 41588_2023_1584_MOESM5_ESM.zip › LocusZoom plots of known EUR risk variants/chr5_133949105-134449105.png]

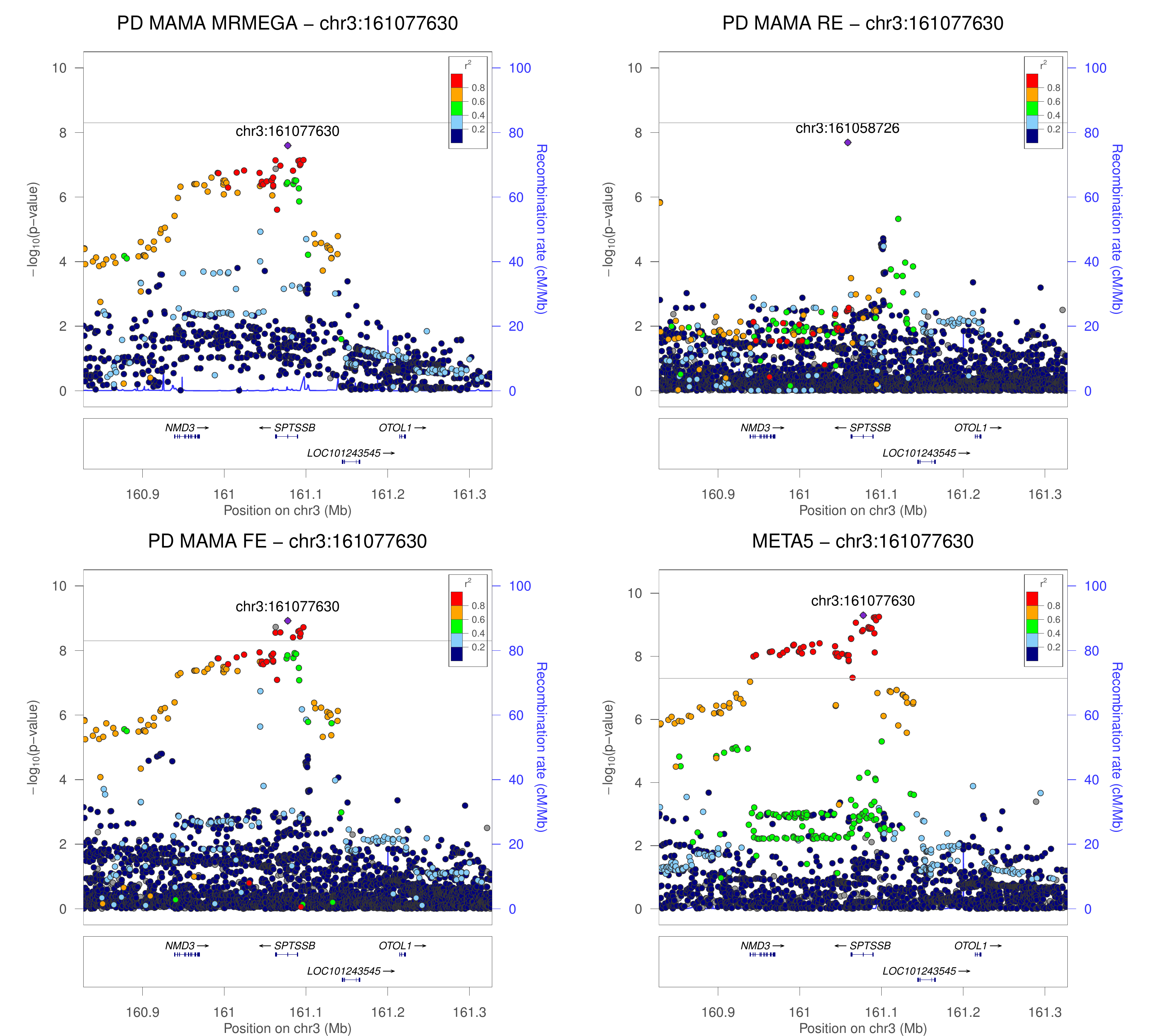

Supplement: Supplementary file 5 — This includes LocusZoom plots of all known European loci as well as novel loci. Each file contains four LocusZoom plots: PD MAMA MR-MEGA/RE/FE/ (MR-MEGA/random-effect/fixed-effect) and META5 (European-only meta-analysis from Nalls et al. 1). [file 41588_2023_1584_MOESM5_ESM.zip › LocusZoom plots of known EUR risk variants/chr3_160827630-161327630.png]

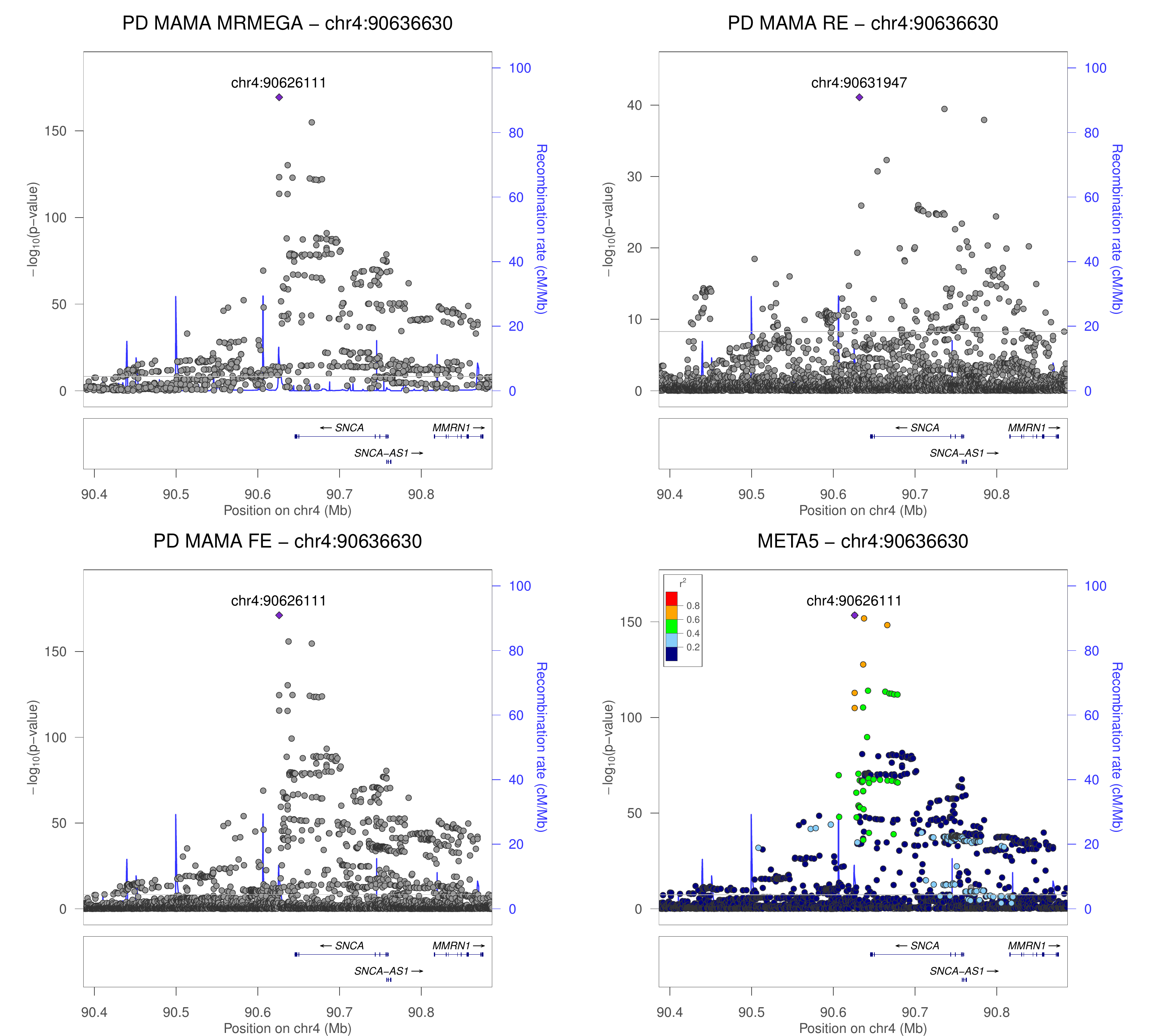

Supplement: Supplementary file 5 — This includes LocusZoom plots of all known European loci as well as novel loci. Each file contains four LocusZoom plots: PD MAMA MR-MEGA/RE/FE/ (MR-MEGA/random-effect/fixed-effect) and META5 (European-only meta-analysis from Nalls et al. 1). [file 41588_2023_1584_MOESM5_ESM.zip › LocusZoom plots of known EUR risk variants/chr4_90386630-90886630.png]

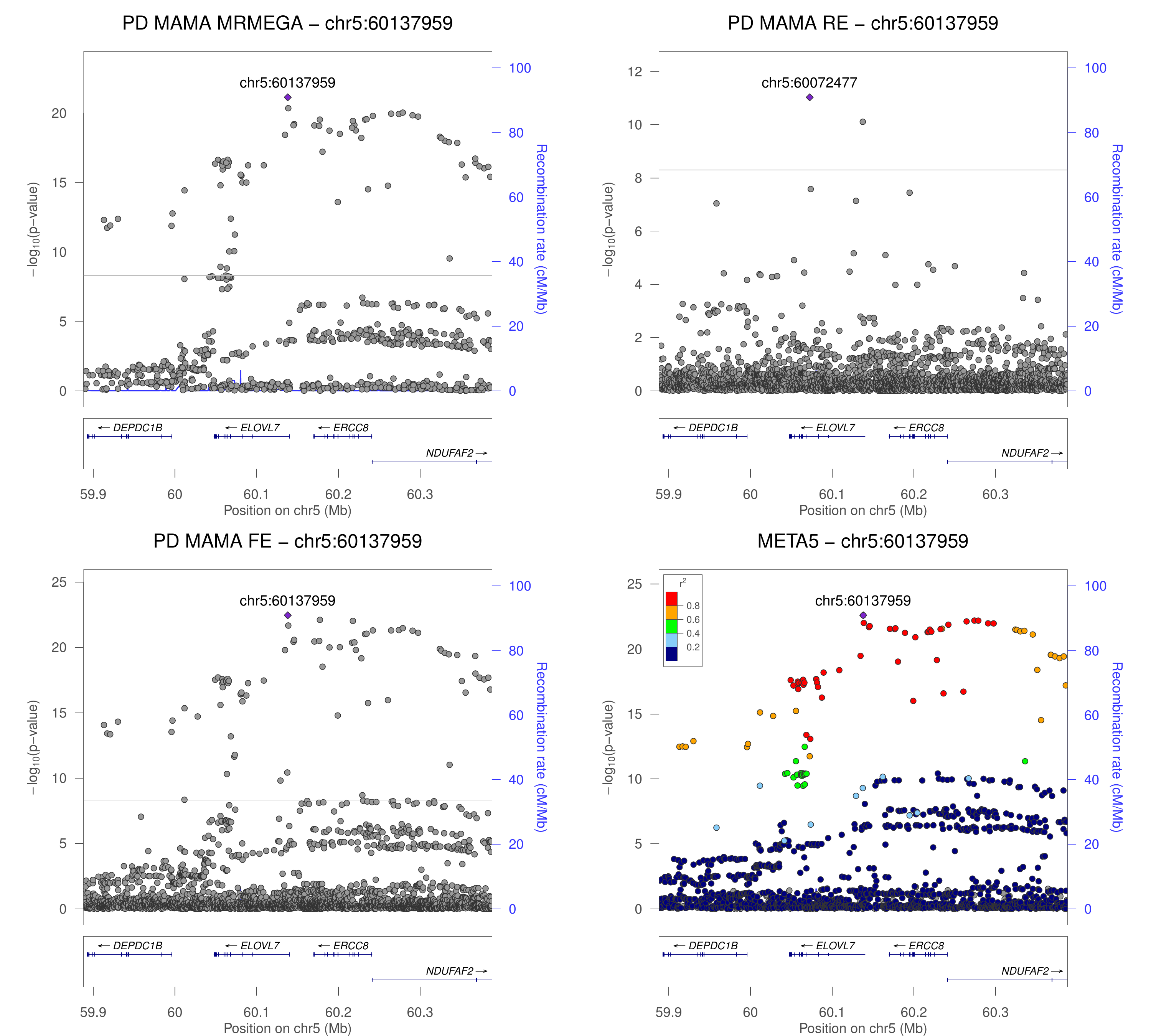

Supplement: Supplementary file 5 — This includes LocusZoom plots of all known European loci as well as novel loci. Each file contains four LocusZoom plots: PD MAMA MR-MEGA/RE/FE/ (MR-MEGA/random-effect/fixed-effect) and META5 (European-only meta-analysis from Nalls et al. 1). [file 41588_2023_1584_MOESM5_ESM.zip › LocusZoom plots of known EUR risk variants/chr5_59887959-60387959.png]

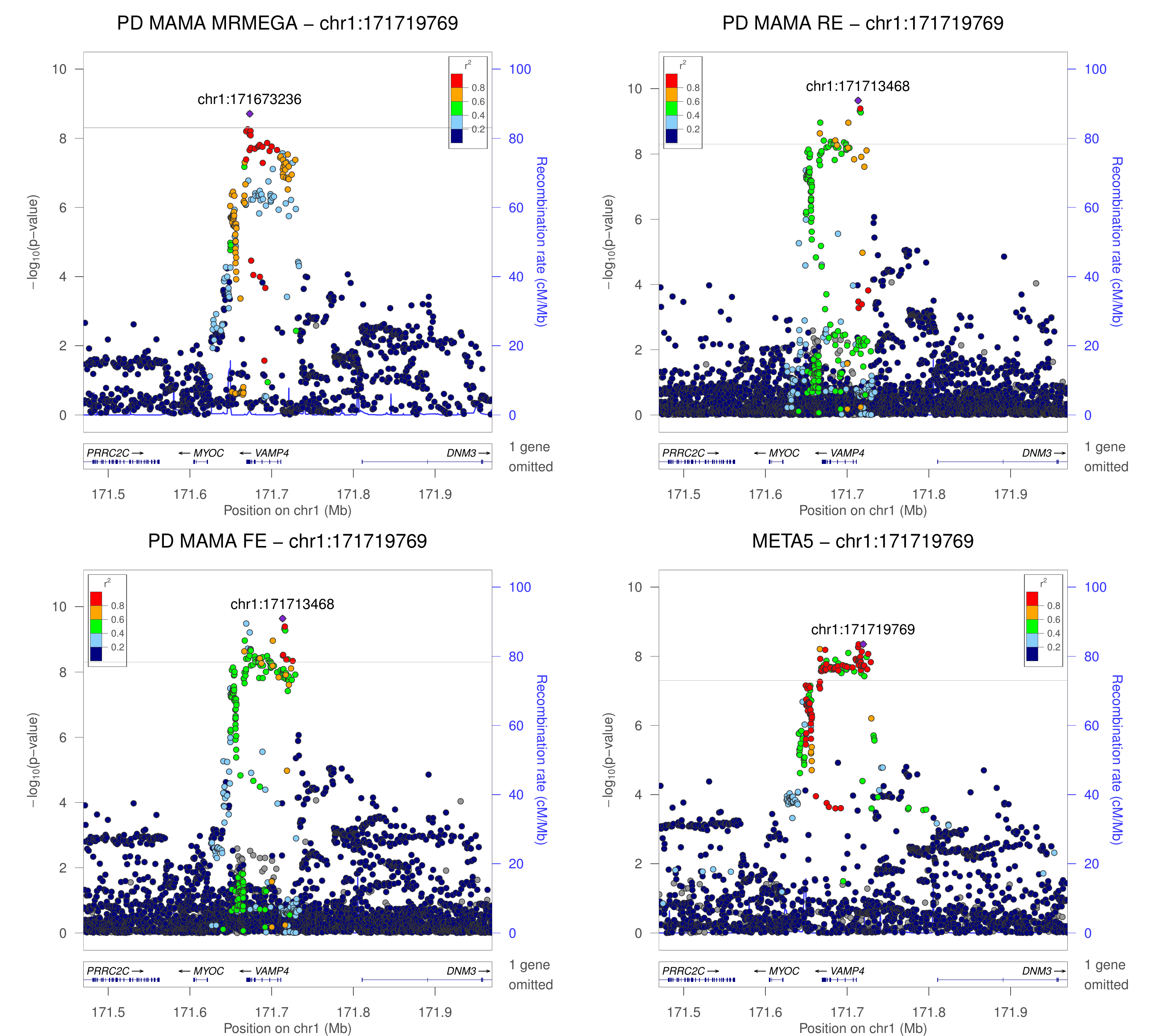

Supplement: Supplementary file 5 — This includes LocusZoom plots of all known European loci as well as novel loci. Each file contains four LocusZoom plots: PD MAMA MR-MEGA/RE/FE/ (MR-MEGA/random-effect/fixed-effect) and META5 (European-only meta-analysis from Nalls et al. 1). [file 41588_2023_1584_MOESM5_ESM.zip › LocusZoom plots of known EUR risk variants/chr1_171469769-171969769.png]

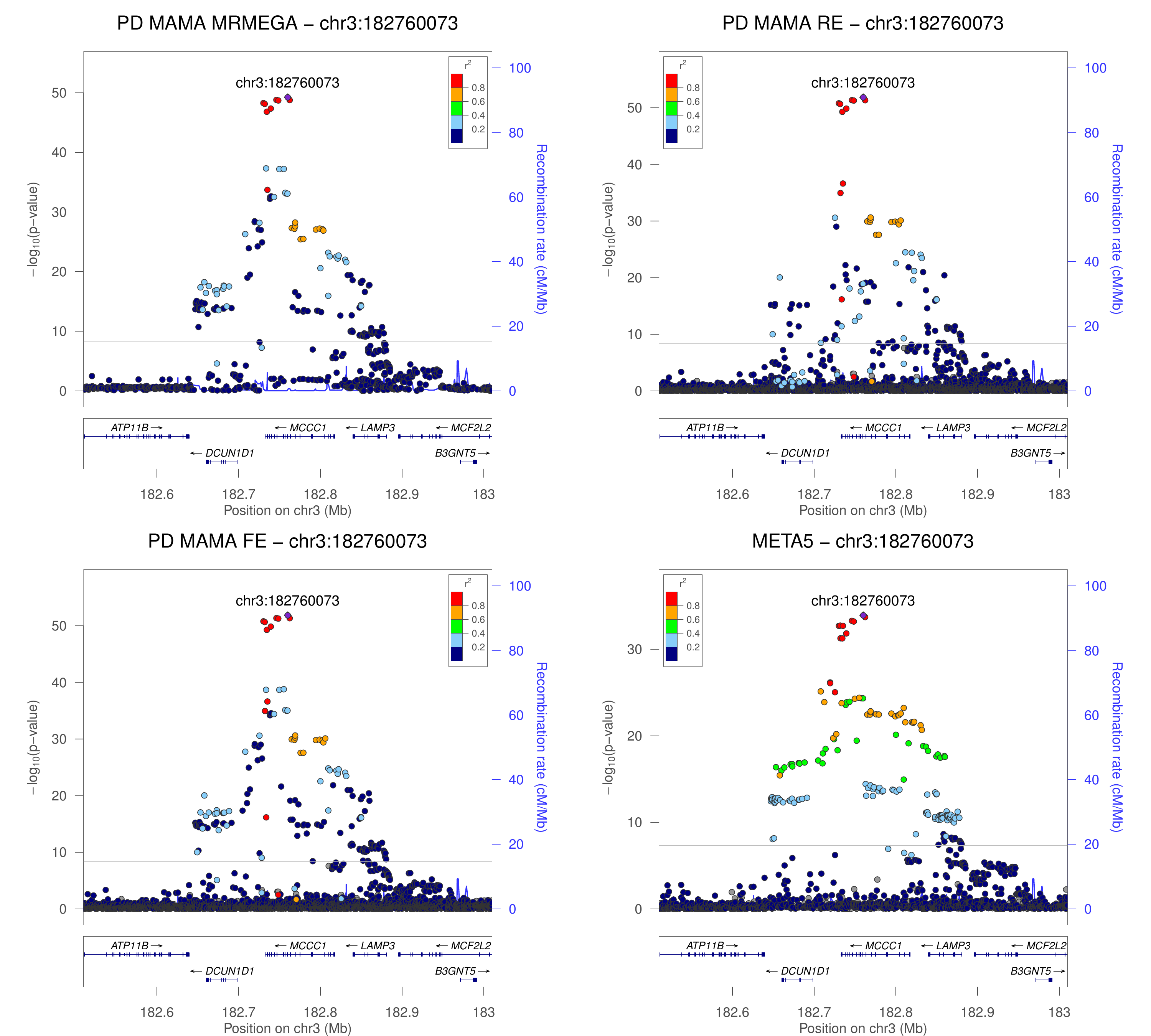

Supplement: Supplementary file 5 — This includes LocusZoom plots of all known European loci as well as novel loci. Each file contains four LocusZoom plots: PD MAMA MR-MEGA/RE/FE/ (MR-MEGA/random-effect/fixed-effect) and META5 (European-only meta-analysis from Nalls et al. 1). [file 41588_2023_1584_MOESM5_ESM.zip › LocusZoom plots of known EUR risk variants/chr3_182510073-183010073.png]

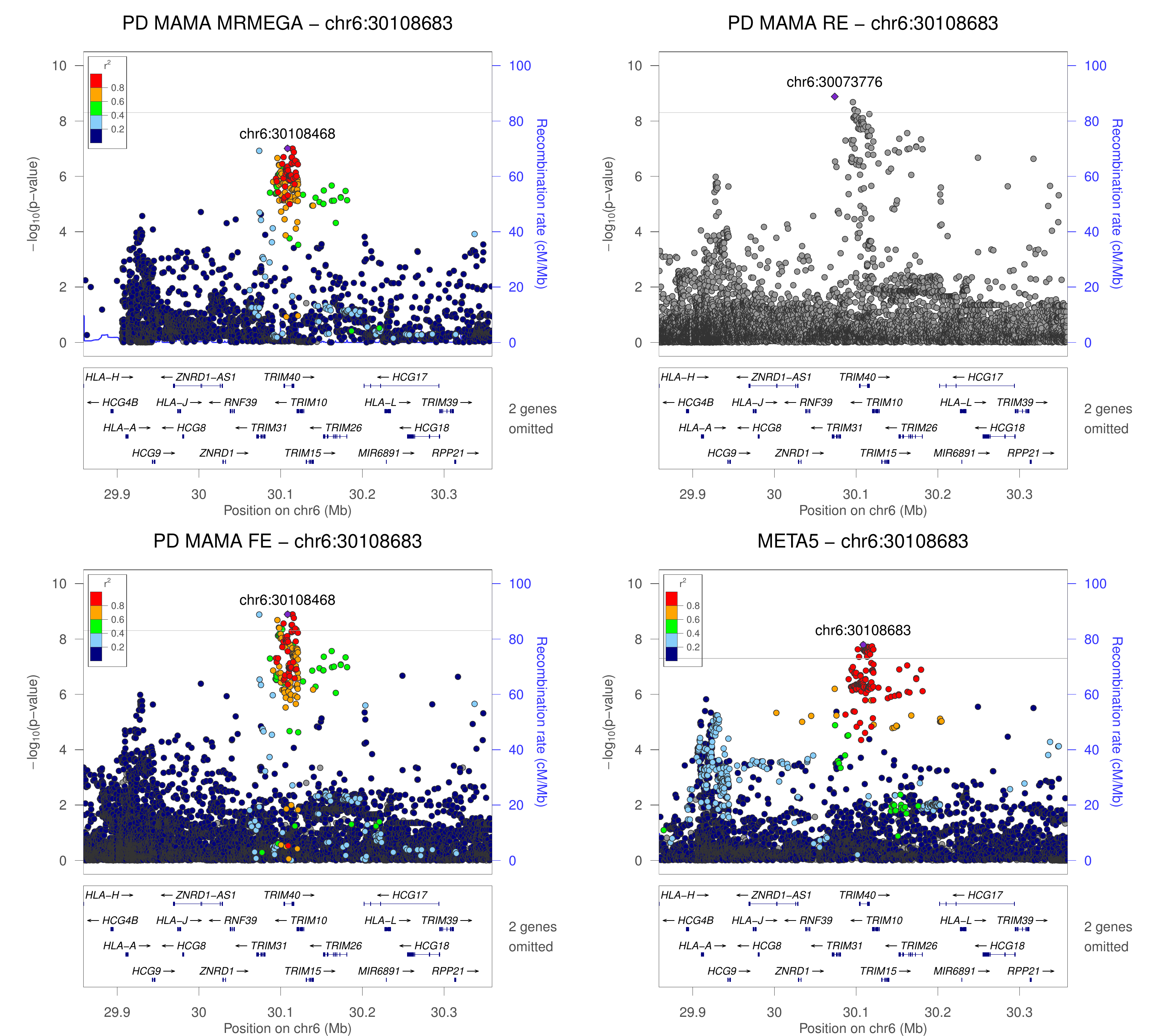

Supplement: Supplementary file 5 — This includes LocusZoom plots of all known European loci as well as novel loci. Each file contains four LocusZoom plots: PD MAMA MR-MEGA/RE/FE/ (MR-MEGA/random-effect/fixed-effect) and META5 (European-only meta-analysis from Nalls et al. 1). [file 41588_2023_1584_MOESM5_ESM.zip › LocusZoom plots of known EUR risk variants/chr6_29858683-30358683.png]

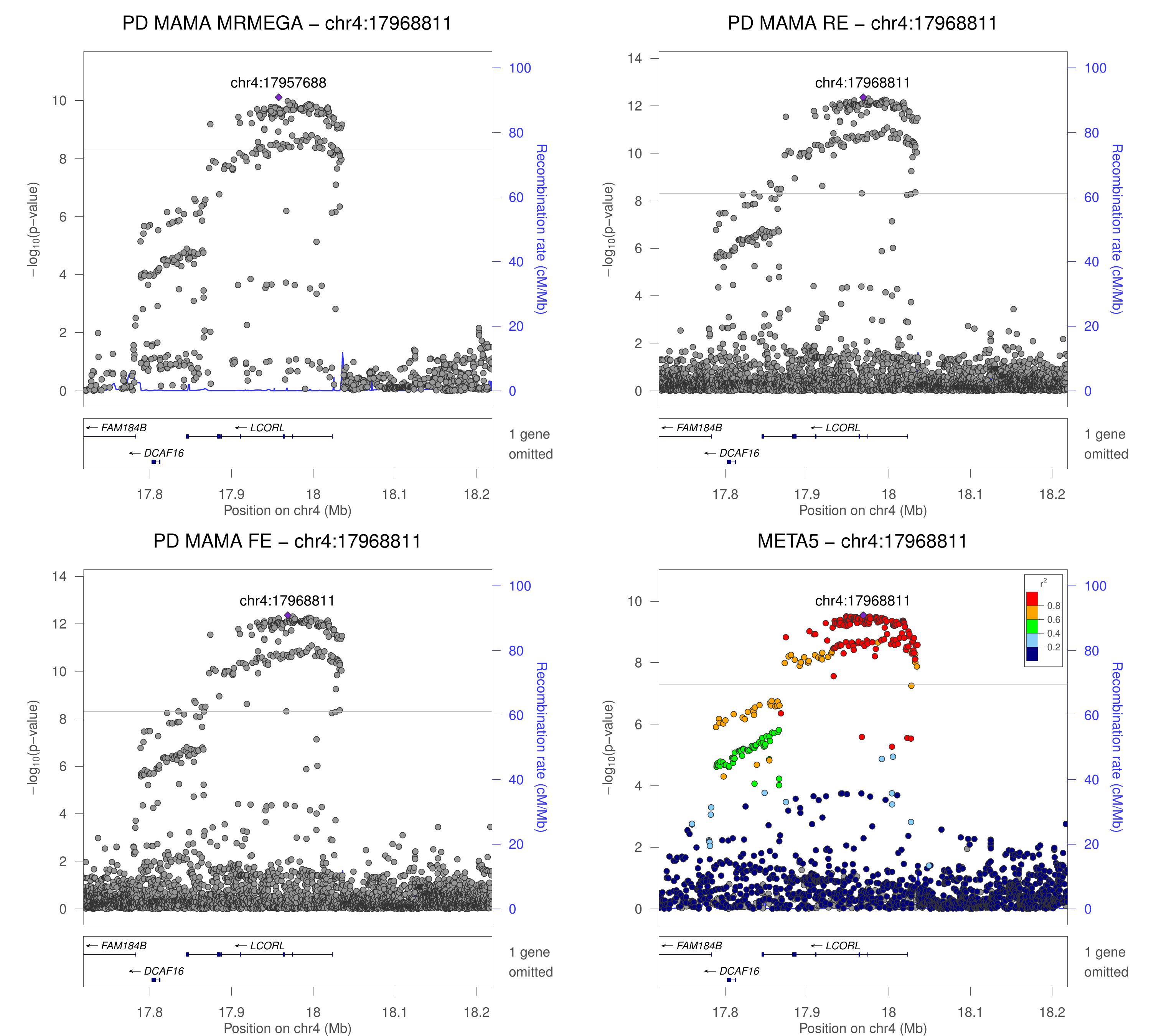

Supplement: Supplementary file 5 — This includes LocusZoom plots of all known European loci as well as novel loci. Each file contains four LocusZoom plots: PD MAMA MR-MEGA/RE/FE/ (MR-MEGA/random-effect/fixed-effect) and META5 (European-only meta-analysis from Nalls et al. 1). [file 41588_2023_1584_MOESM5_ESM.zip › LocusZoom plots of known EUR risk variants/chr4_17718811-18218811.png]

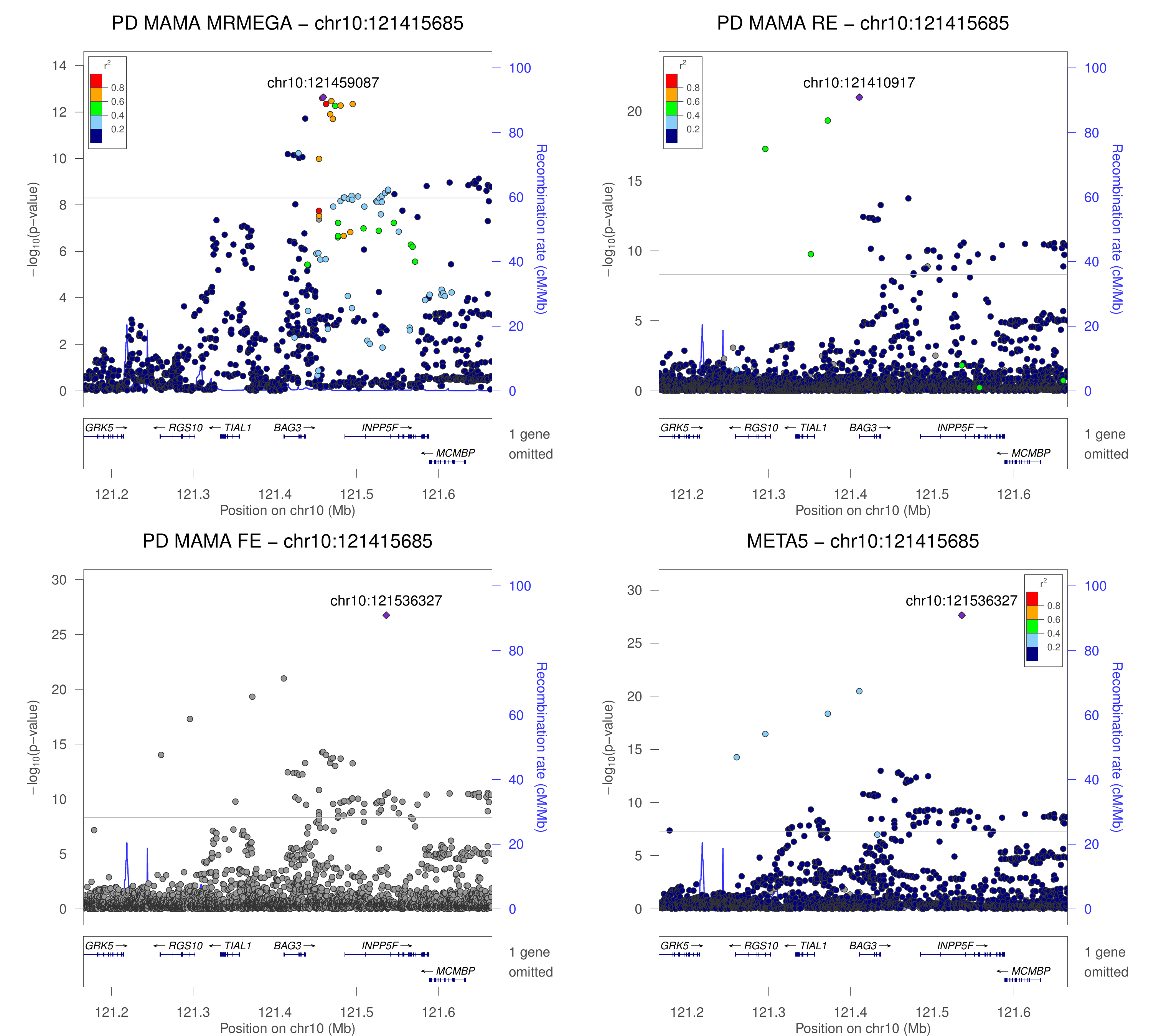

Supplement: Supplementary file 5 — This includes LocusZoom plots of all known European loci as well as novel loci. Each file contains four LocusZoom plots: PD MAMA MR-MEGA/RE/FE/ (MR-MEGA/random-effect/fixed-effect) and META5 (European-only meta-analysis from Nalls et al. 1). [file 41588_2023_1584_MOESM5_ESM.zip › LocusZoom plots of known EUR risk variants/chr10_121165685-121665685.png]

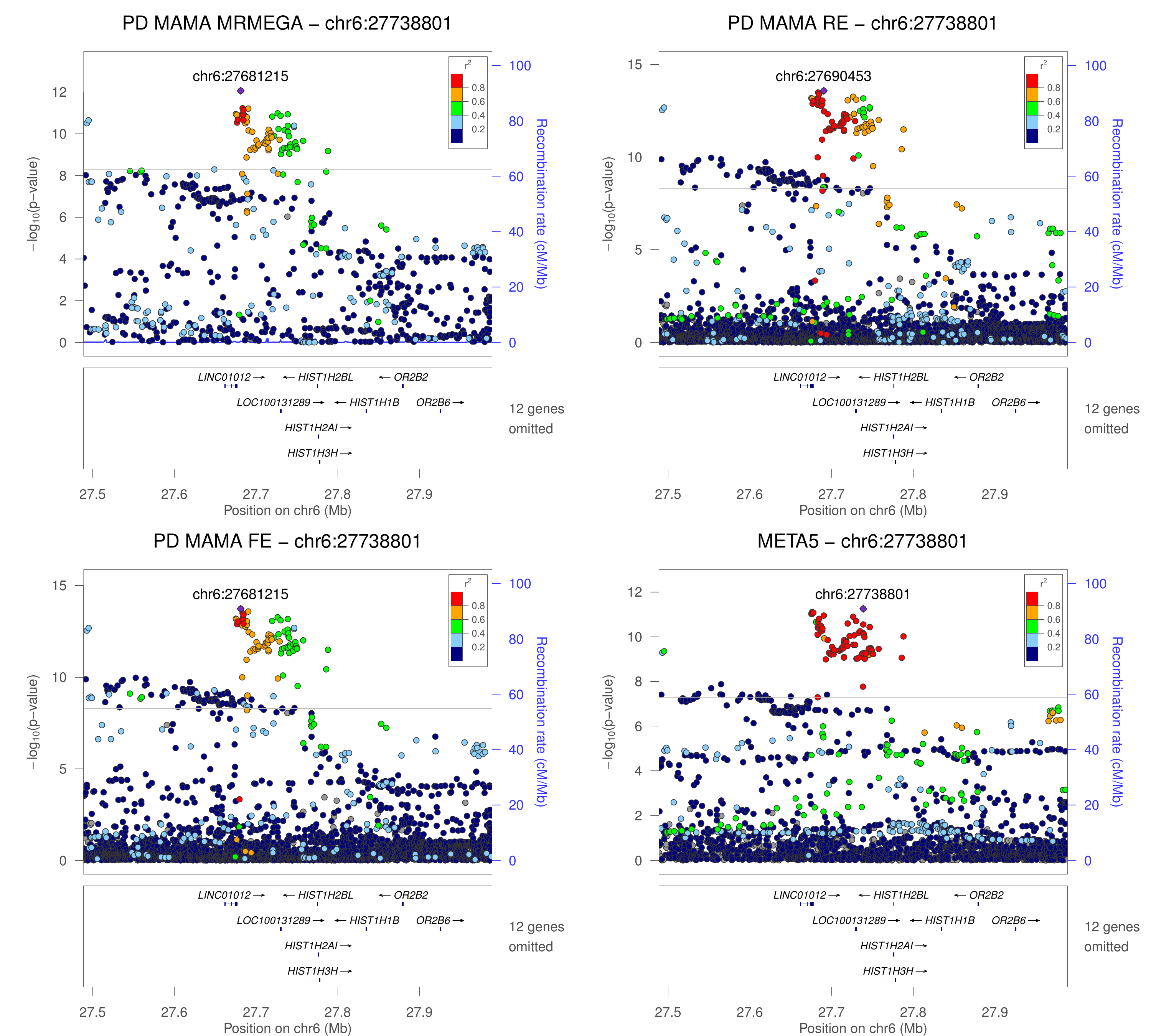

Supplement: Supplementary file 5 — This includes LocusZoom plots of all known European loci as well as novel loci. Each file contains four LocusZoom plots: PD MAMA MR-MEGA/RE/FE/ (MR-MEGA/random-effect/fixed-effect) and META5 (European-only meta-analysis from Nalls et al. 1). [file 41588_2023_1584_MOESM5_ESM.zip › LocusZoom plots of known EUR risk variants/chr6_27488801-27988801.png]

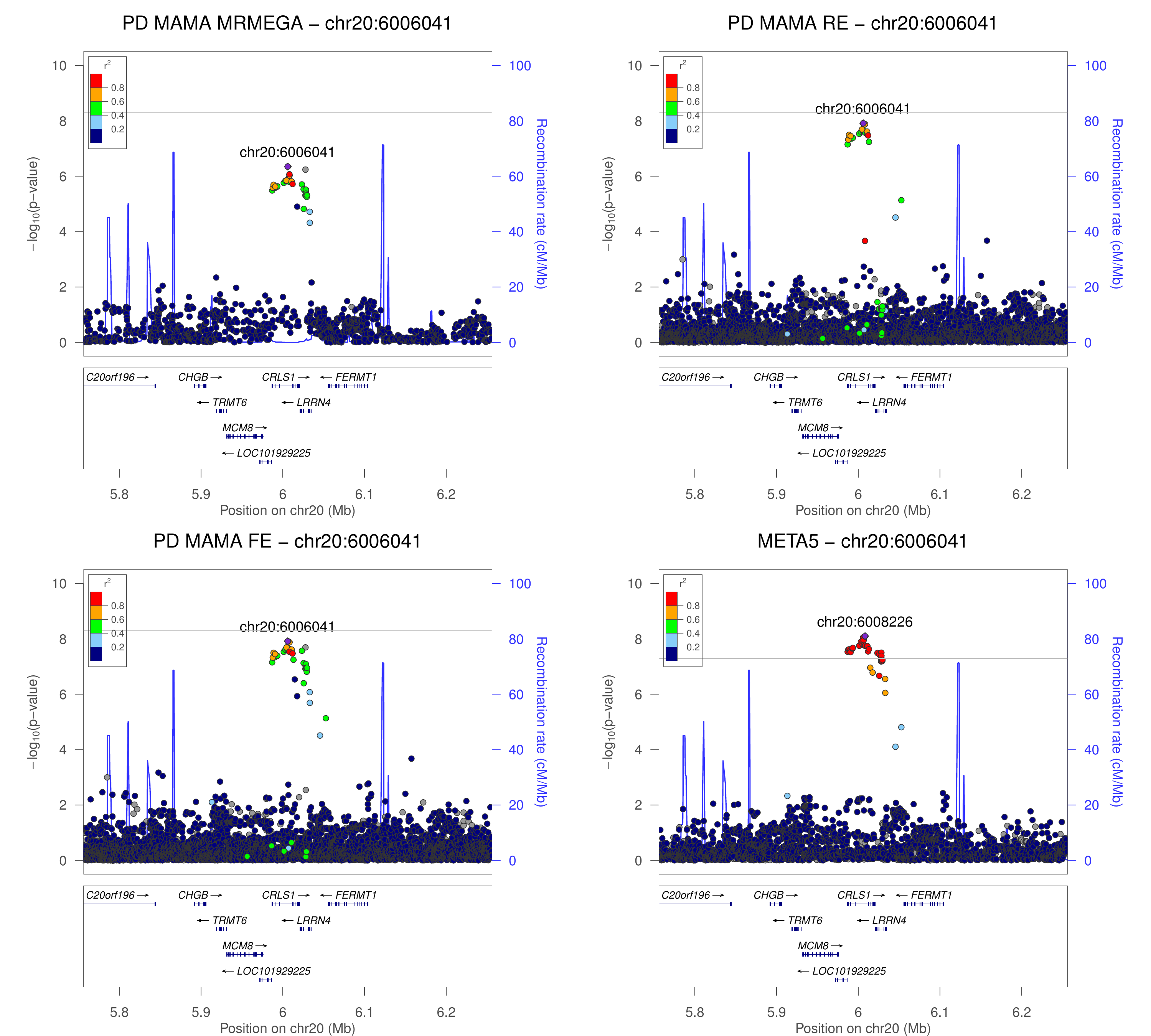

Supplement: Supplementary file 5 — This includes LocusZoom plots of all known European loci as well as novel loci. Each file contains four LocusZoom plots: PD MAMA MR-MEGA/RE/FE/ (MR-MEGA/random-effect/fixed-effect) and META5 (European-only meta-analysis from Nalls et al. 1). [file 41588_2023_1584_MOESM5_ESM.zip › LocusZoom plots of known EUR risk variants/chr20_5756041-6256041.png]

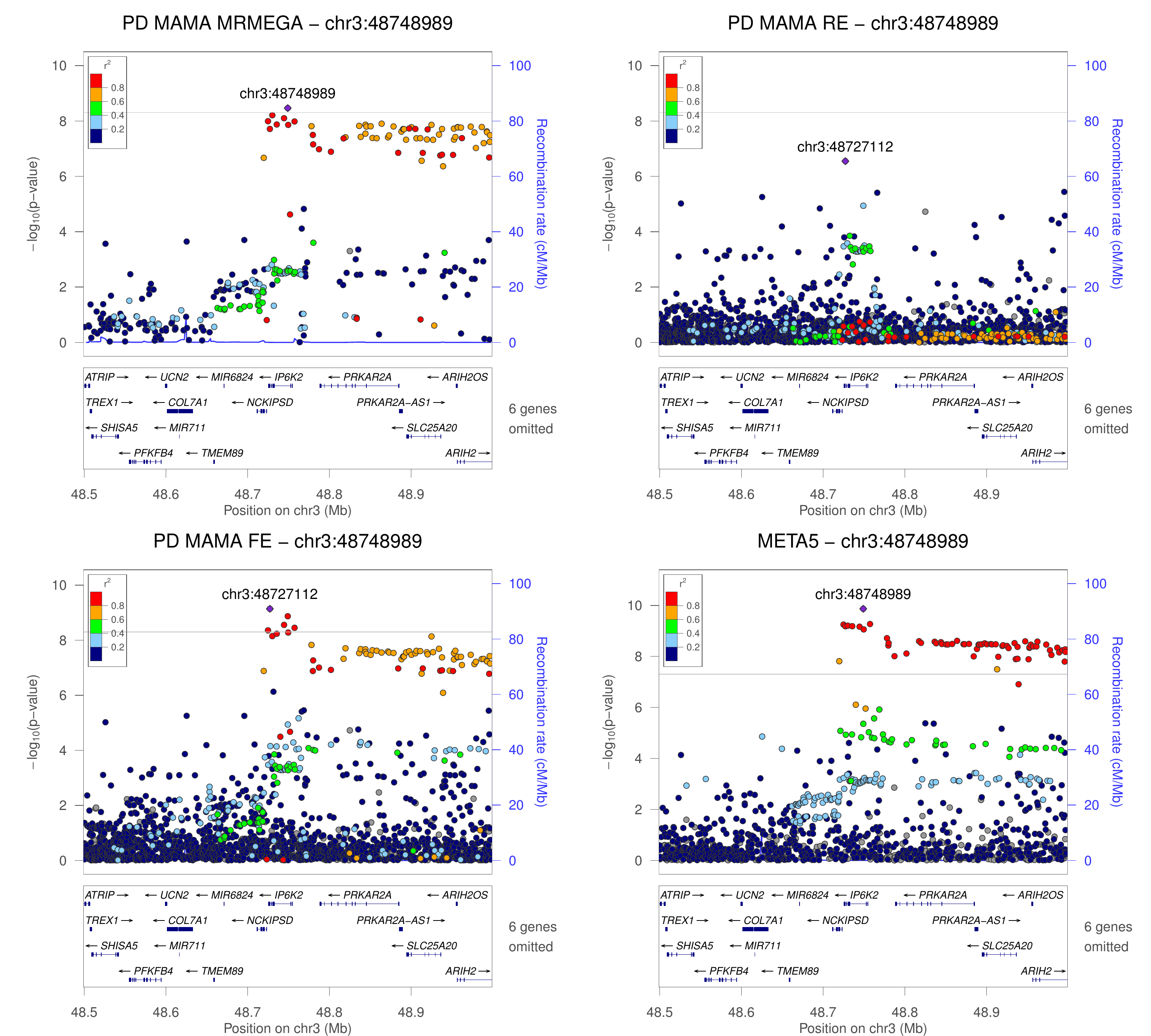

Supplement: Supplementary file 5 — This includes LocusZoom plots of all known European loci as well as novel loci. Each file contains four LocusZoom plots: PD MAMA MR-MEGA/RE/FE/ (MR-MEGA/random-effect/fixed-effect) and META5 (European-only meta-analysis from Nalls et al. 1). [file 41588_2023_1584_MOESM5_ESM.zip › LocusZoom plots of known EUR risk variants/chr3_48498989-48998989.png]

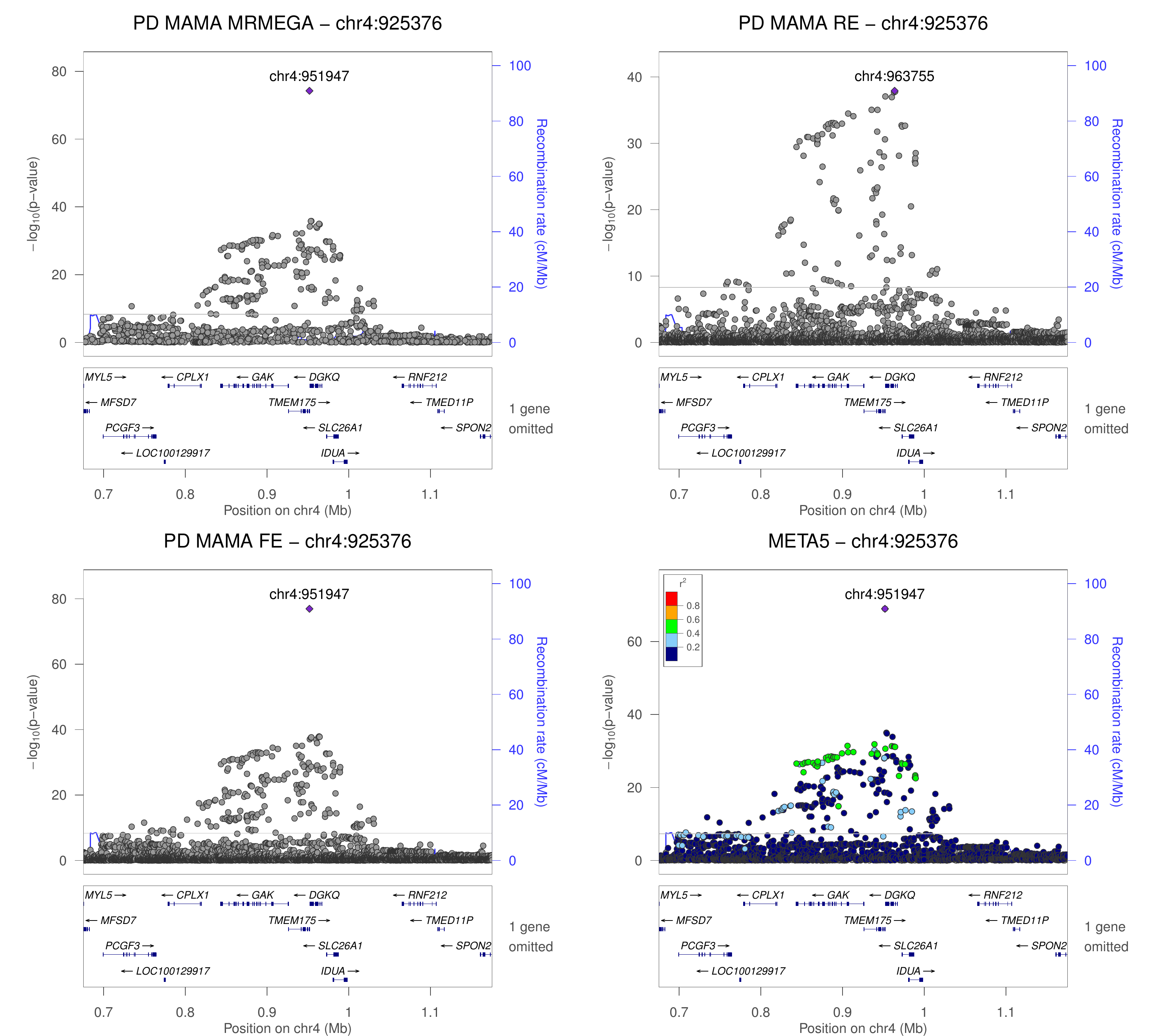

Supplement: Supplementary file 5 — This includes LocusZoom plots of all known European loci as well as novel loci. Each file contains four LocusZoom plots: PD MAMA MR-MEGA/RE/FE/ (MR-MEGA/random-effect/fixed-effect) and META5 (European-only meta-analysis from Nalls et al. 1). [file 41588_2023_1584_MOESM5_ESM.zip › LocusZoom plots of known EUR risk variants/chr4_675376-1175376.png]

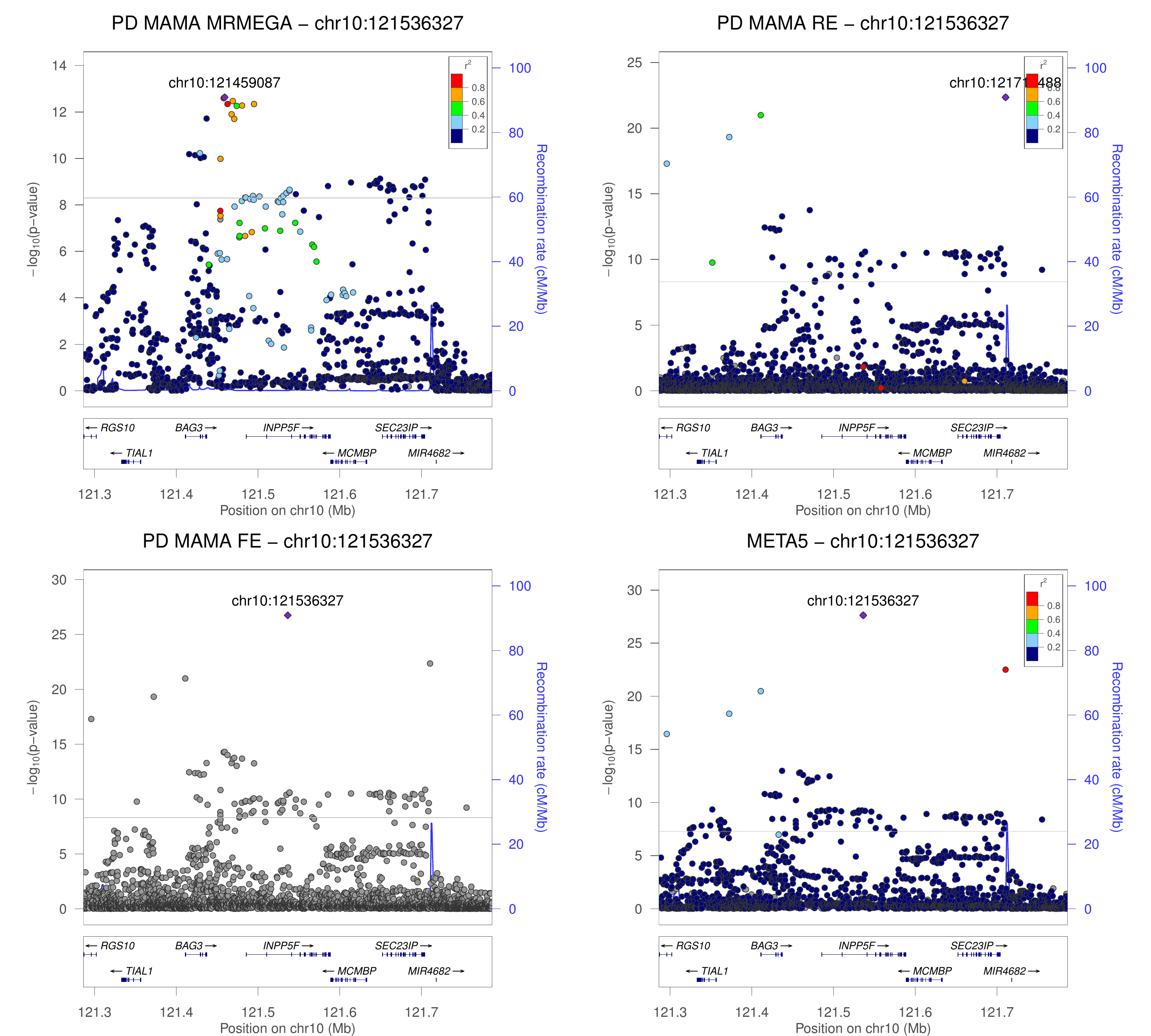

Supplement: Supplementary file 5 — This includes LocusZoom plots of all known European loci as well as novel loci. Each file contains four LocusZoom plots: PD MAMA MR-MEGA/RE/FE/ (MR-MEGA/random-effect/fixed-effect) and META5 (European-only meta-analysis from Nalls et al. 1). [file 41588_2023_1584_MOESM5_ESM.zip › LocusZoom plots of known EUR risk variants/chr10_121286327-121786327.png]

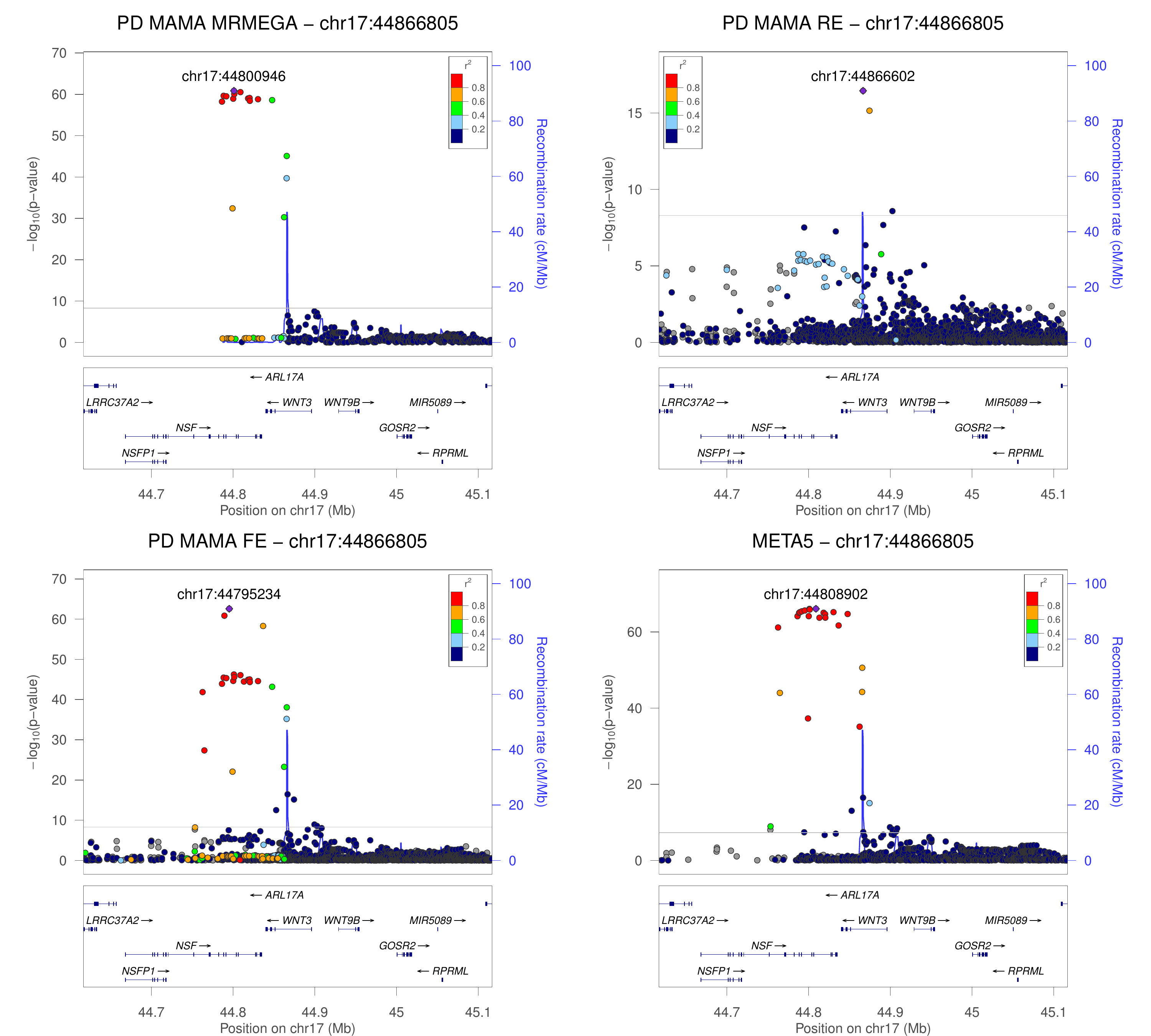

Supplement: Supplementary file 5 — This includes LocusZoom plots of all known European loci as well as novel loci. Each file contains four LocusZoom plots: PD MAMA MR-MEGA/RE/FE/ (MR-MEGA/random-effect/fixed-effect) and META5 (European-only meta-analysis from Nalls et al. 1). [file 41588_2023_1584_MOESM5_ESM.zip › LocusZoom plots of known EUR risk variants/chr17_44616805-45116805.png]

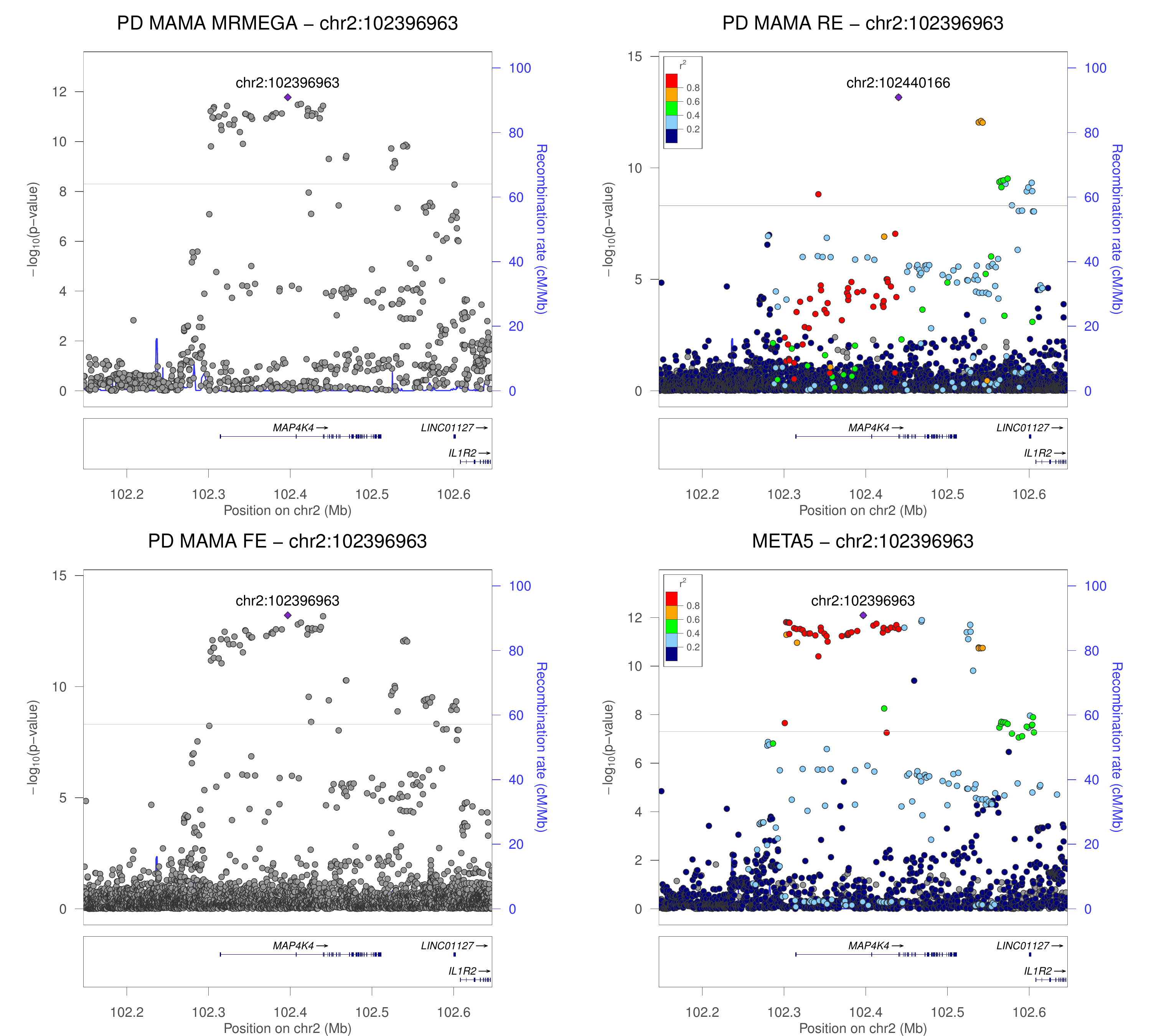

Supplement: Supplementary file 5 — This includes LocusZoom plots of all known European loci as well as novel loci. Each file contains four LocusZoom plots: PD MAMA MR-MEGA/RE/FE/ (MR-MEGA/random-effect/fixed-effect) and META5 (European-only meta-analysis from Nalls et al. 1). [file 41588_2023_1584_MOESM5_ESM.zip › LocusZoom plots of known EUR risk variants/chr2_102146963-102646963.png]

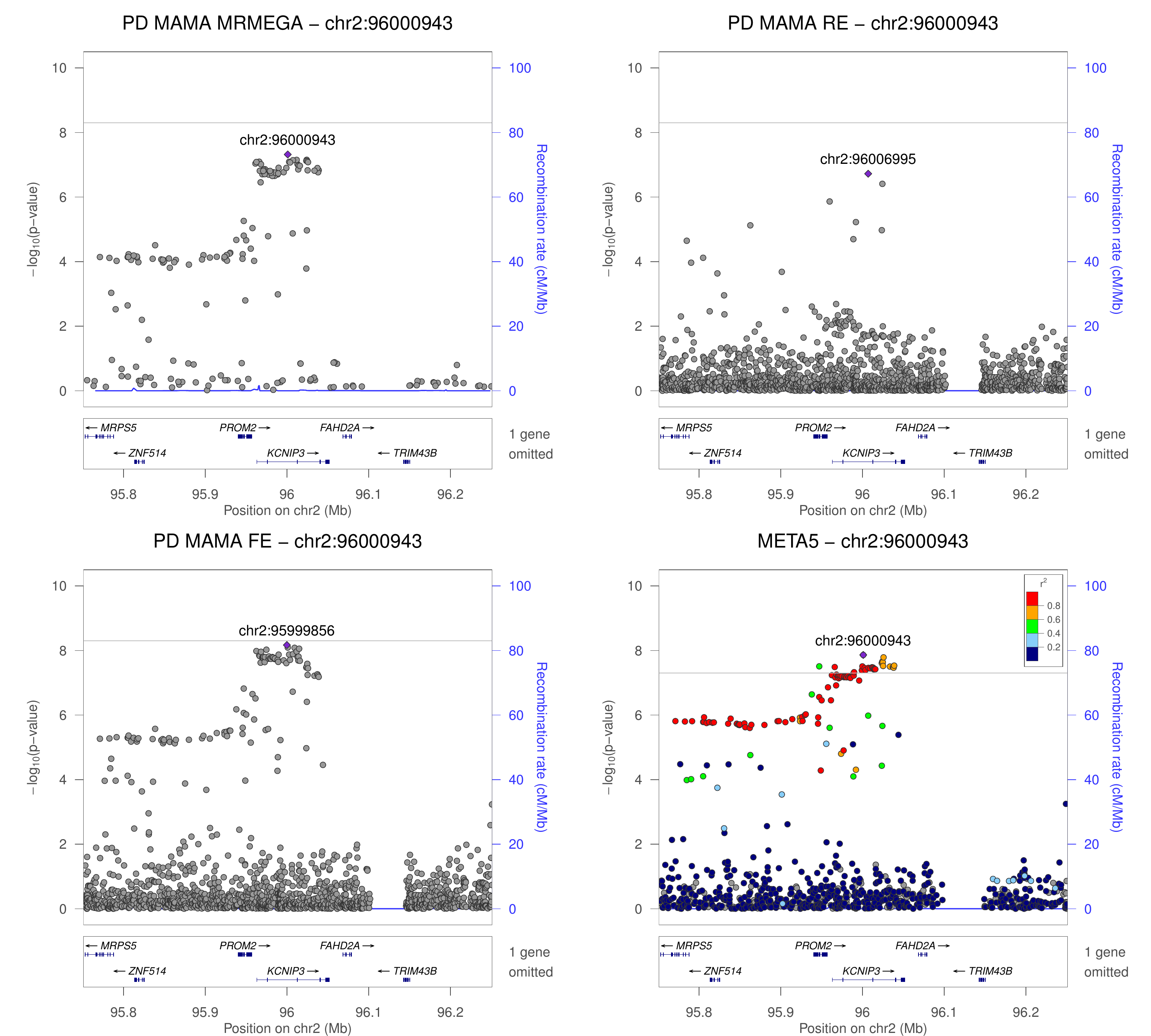

Supplement: Supplementary file 5 — This includes LocusZoom plots of all known European loci as well as novel loci. Each file contains four LocusZoom plots: PD MAMA MR-MEGA/RE/FE/ (MR-MEGA/random-effect/fixed-effect) and META5 (European-only meta-analysis from Nalls et al. 1). [file 41588_2023_1584_MOESM5_ESM.zip › LocusZoom plots of known EUR risk variants/chr2_95750943-96250943.png]

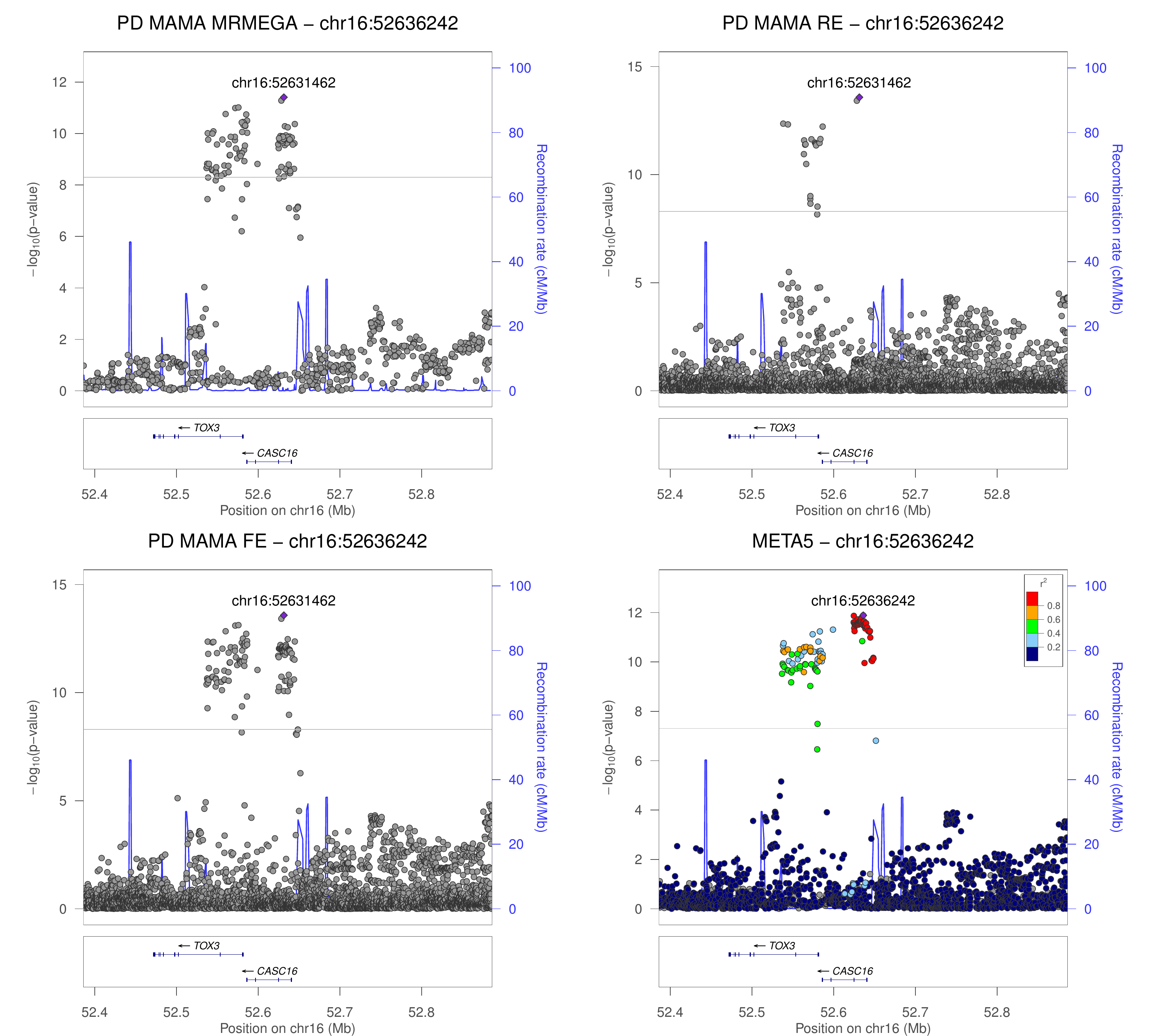

Supplement: Supplementary file 5 — This includes LocusZoom plots of all known European loci as well as novel loci. Each file contains four LocusZoom plots: PD MAMA MR-MEGA/RE/FE/ (MR-MEGA/random-effect/fixed-effect) and META5 (European-only meta-analysis from Nalls et al. 1). [file 41588_2023_1584_MOESM5_ESM.zip › LocusZoom plots of known EUR risk variants/chr16_52386242-52886242.png]

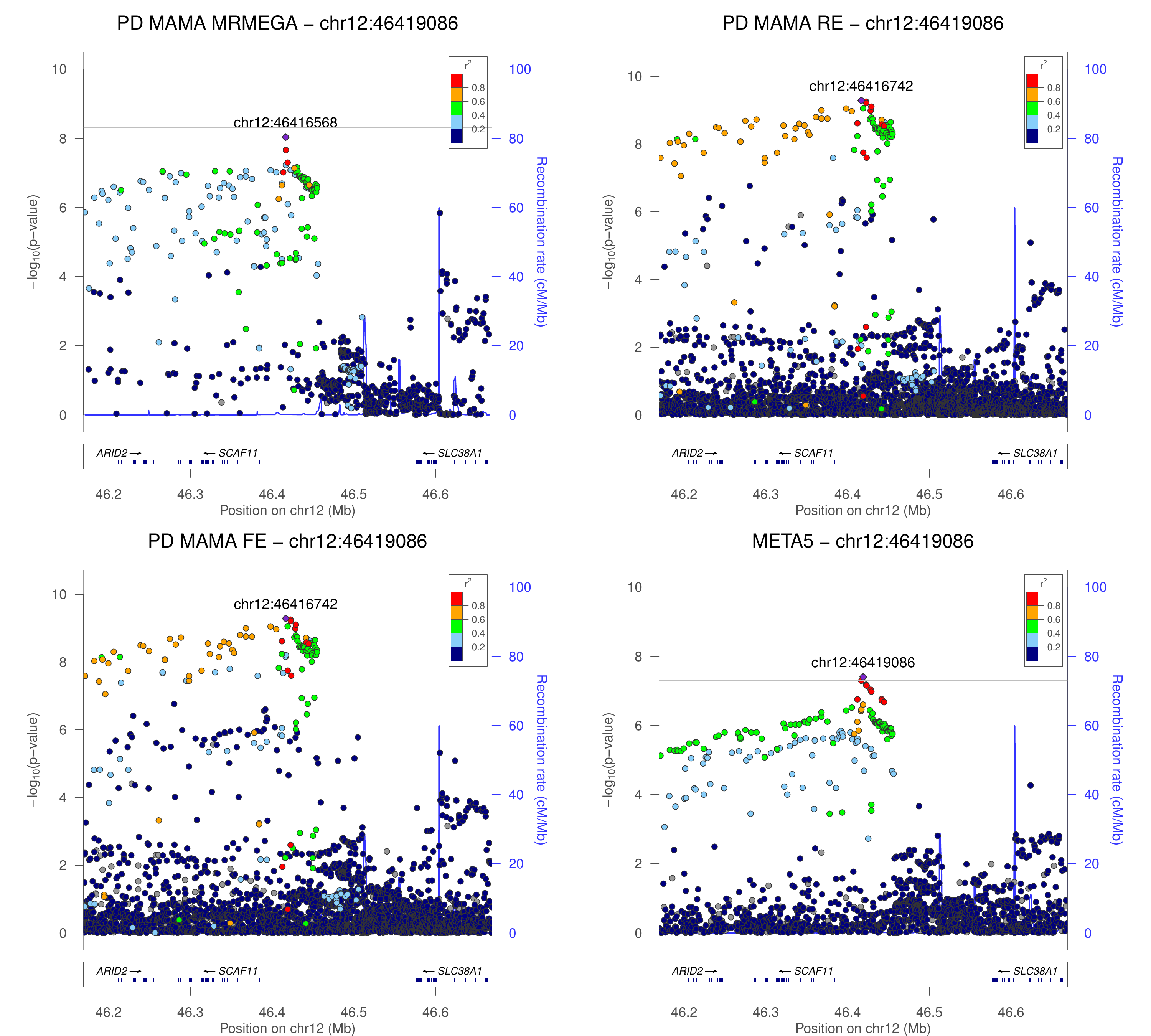

Supplement: Supplementary file 5 — This includes LocusZoom plots of all known European loci as well as novel loci. Each file contains four LocusZoom plots: PD MAMA MR-MEGA/RE/FE/ (MR-MEGA/random-effect/fixed-effect) and META5 (European-only meta-analysis from Nalls et al. 1). [file 41588_2023_1584_MOESM5_ESM.zip › LocusZoom plots of known EUR risk variants/chr12_46169086-46669086.png]

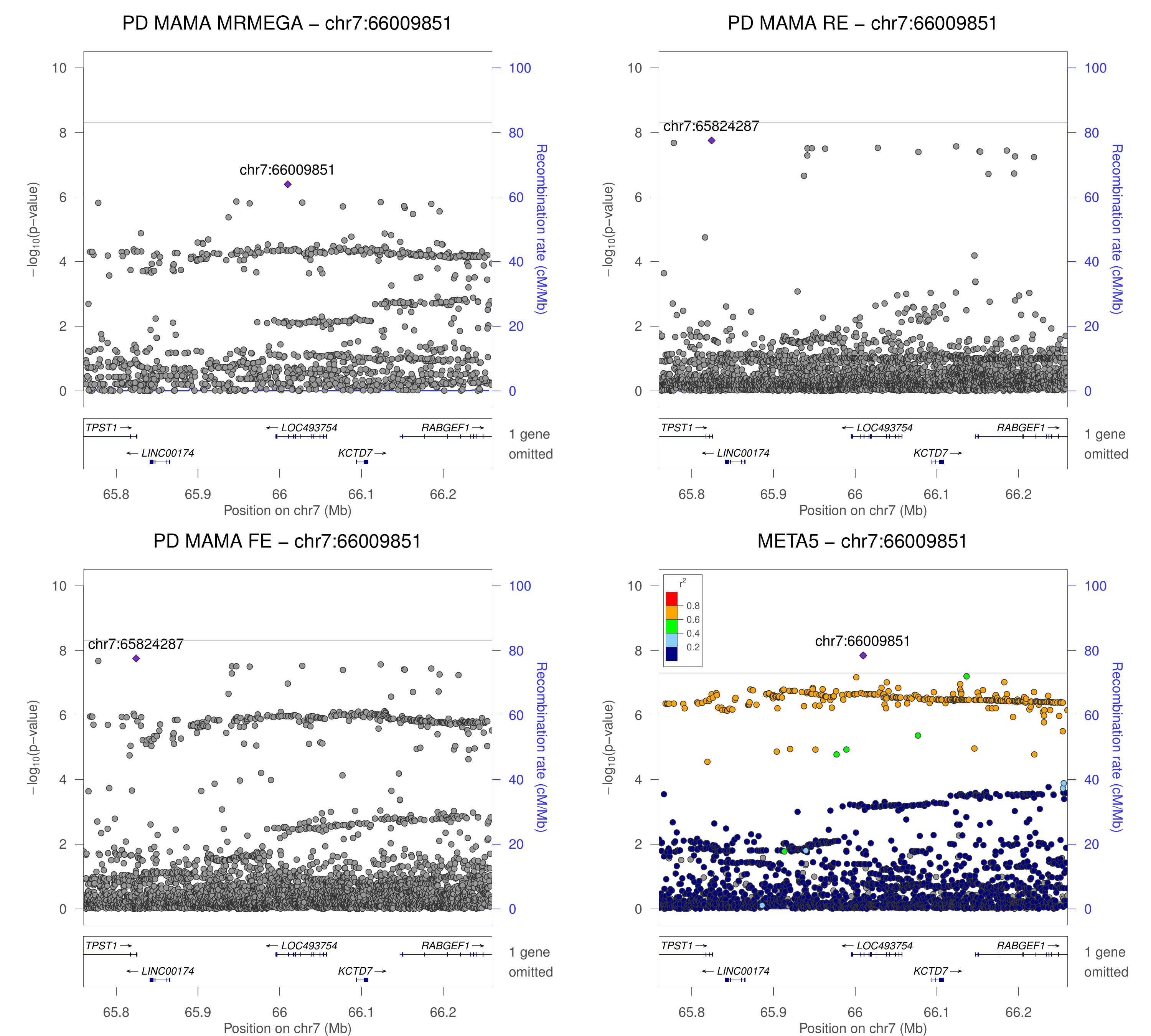

Supplement: Supplementary file 5 — This includes LocusZoom plots of all known European loci as well as novel loci. Each file contains four LocusZoom plots: PD MAMA MR-MEGA/RE/FE/ (MR-MEGA/random-effect/fixed-effect) and META5 (European-only meta-analysis from Nalls et al. 1). [file 41588_2023_1584_MOESM5_ESM.zip › LocusZoom plots of known EUR risk variants/chr7_65759851-66259851.png]

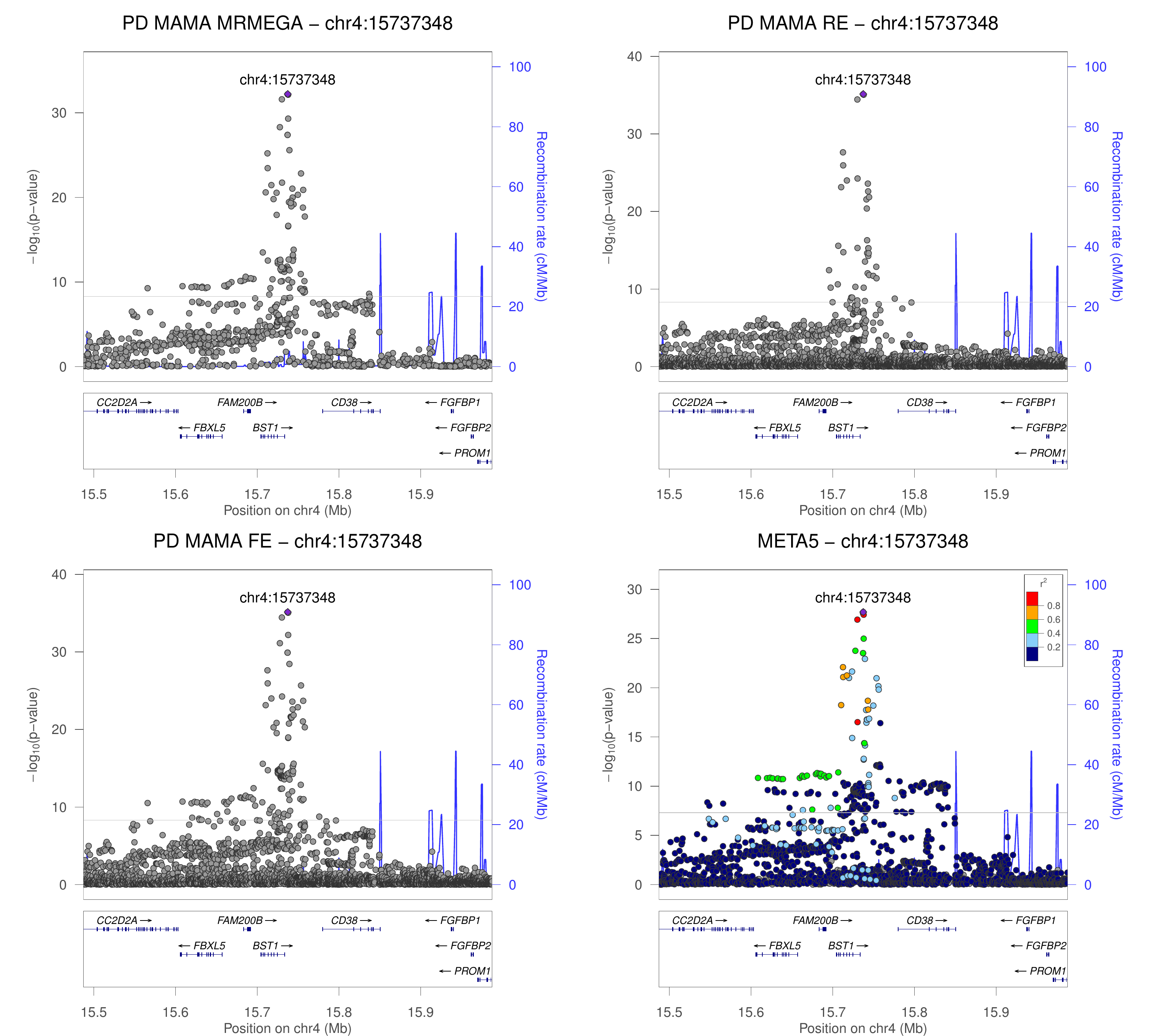

Supplement: Supplementary file 5 — This includes LocusZoom plots of all known European loci as well as novel loci. Each file contains four LocusZoom plots: PD MAMA MR-MEGA/RE/FE/ (MR-MEGA/random-effect/fixed-effect) and META5 (European-only meta-analysis from Nalls et al. 1). [file 41588_2023_1584_MOESM5_ESM.zip › LocusZoom plots of known EUR risk variants/chr4_15487348-15987348.png]

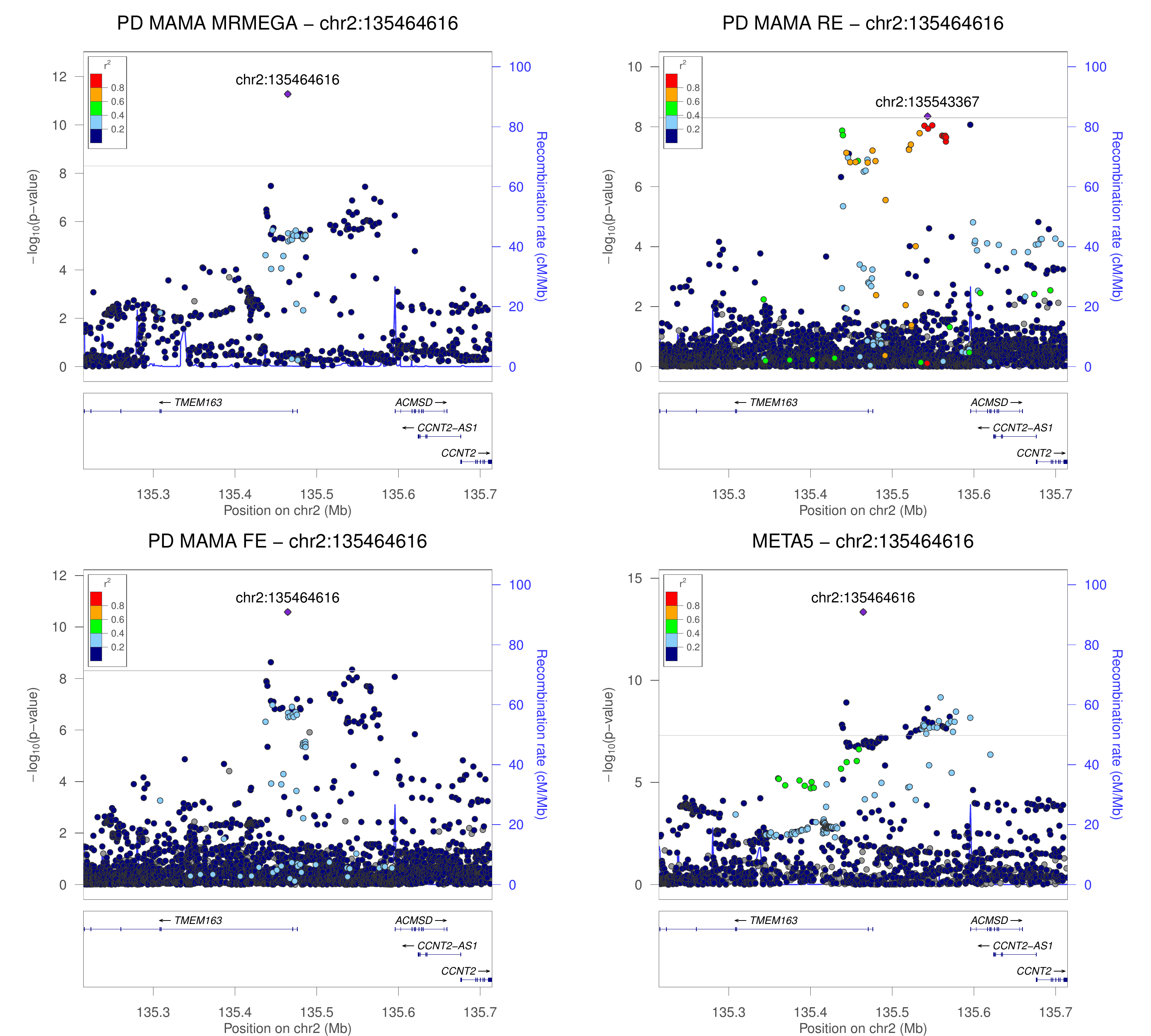

Supplement: Supplementary file 5 — This includes LocusZoom plots of all known European loci as well as novel loci. Each file contains four LocusZoom plots: PD MAMA MR-MEGA/RE/FE/ (MR-MEGA/random-effect/fixed-effect) and META5 (European-only meta-analysis from Nalls et al. 1). [file 41588_2023_1584_MOESM5_ESM.zip › LocusZoom plots of known EUR risk variants/chr2_135214616-135714616.png]

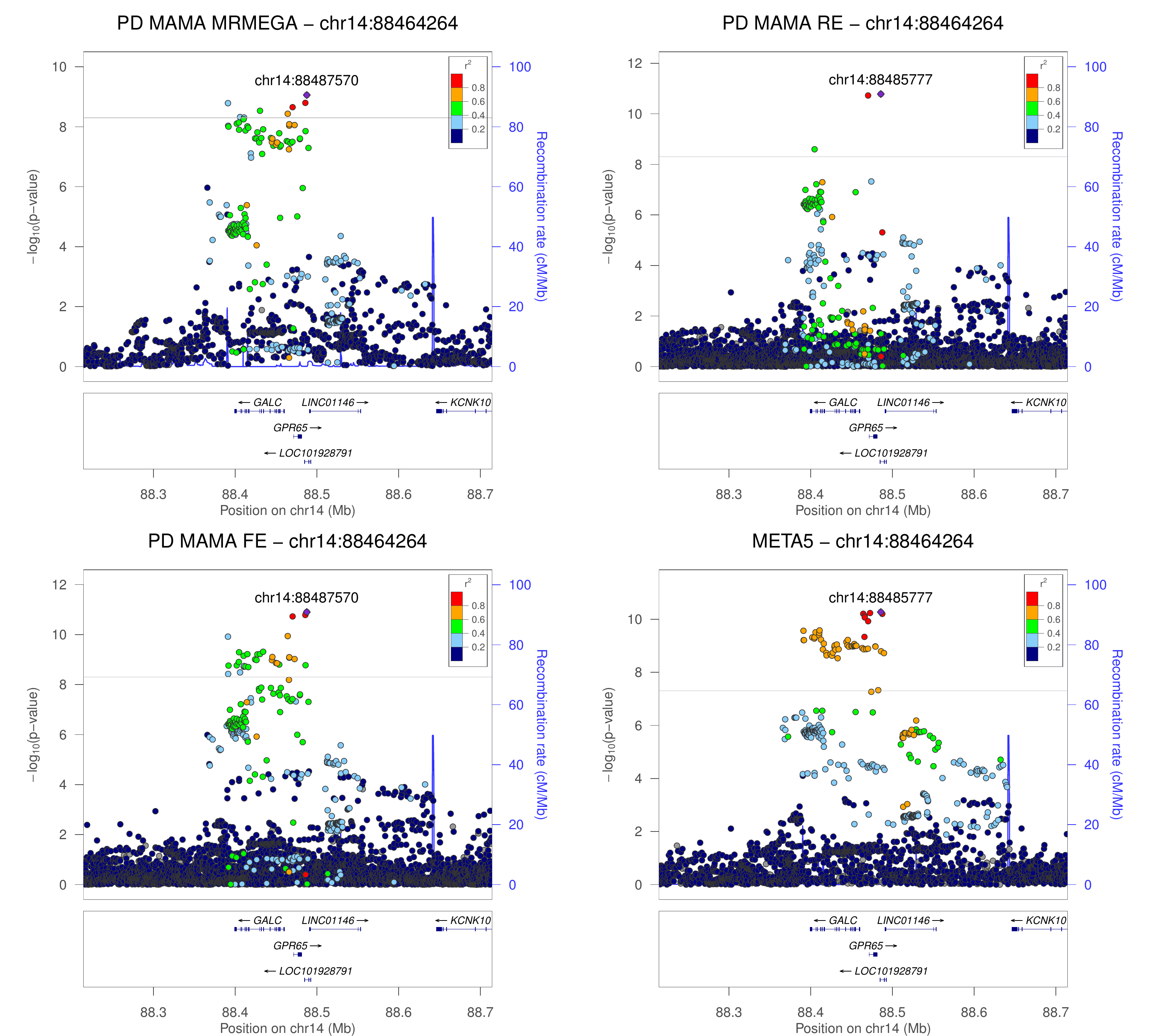

Supplement: Supplementary file 5 — This includes LocusZoom plots of all known European loci as well as novel loci. Each file contains four LocusZoom plots: PD MAMA MR-MEGA/RE/FE/ (MR-MEGA/random-effect/fixed-effect) and META5 (European-only meta-analysis from Nalls et al. 1). [file 41588_2023_1584_MOESM5_ESM.zip › LocusZoom plots of known EUR risk variants/chr14_88214264-88714264.png]

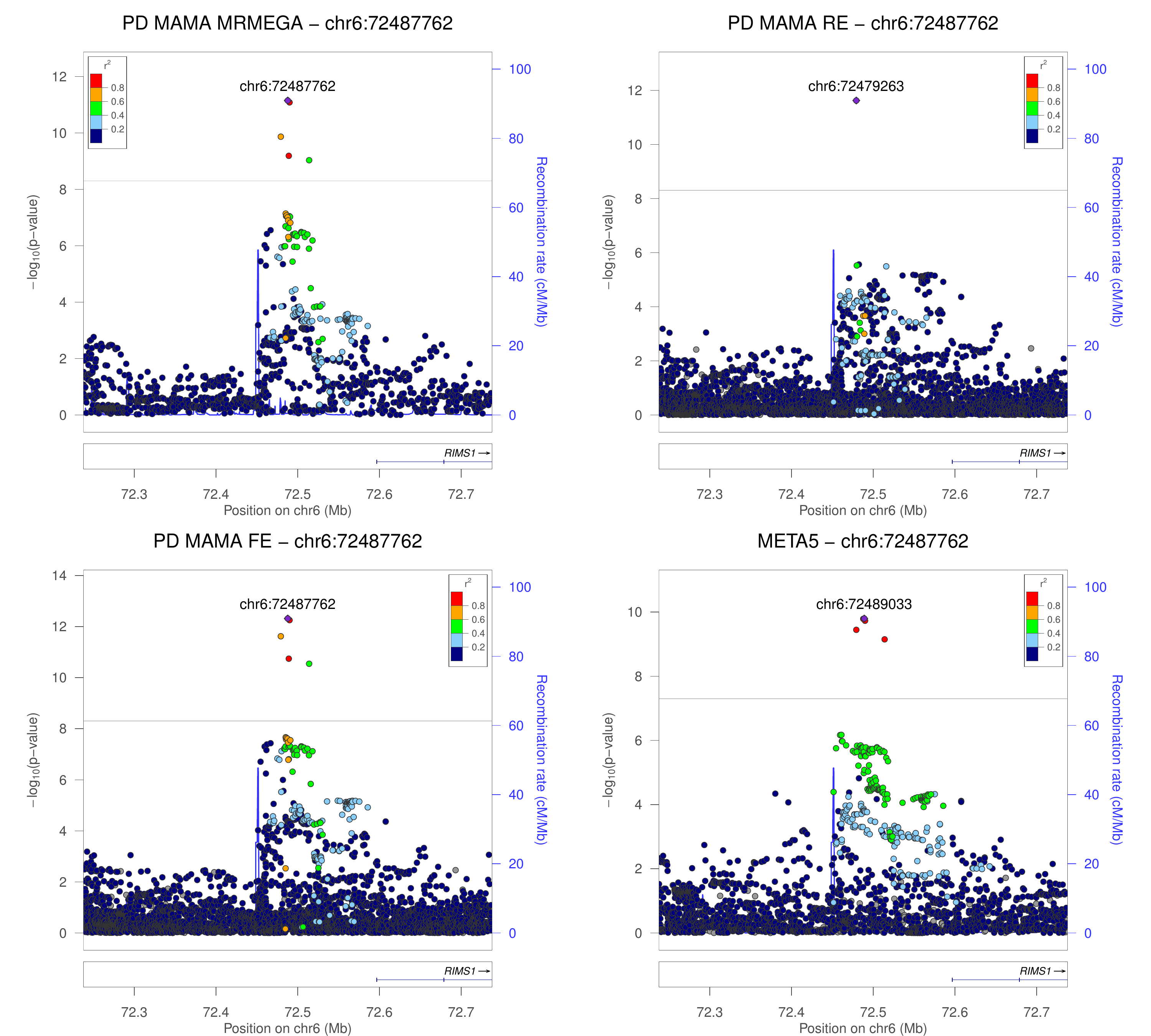

Supplement: Supplementary file 5 — This includes LocusZoom plots of all known European loci as well as novel loci. Each file contains four LocusZoom plots: PD MAMA MR-MEGA/RE/FE/ (MR-MEGA/random-effect/fixed-effect) and META5 (European-only meta-analysis from Nalls et al. 1). [file 41588_2023_1584_MOESM5_ESM.zip › LocusZoom plots of known EUR risk variants/chr6_72237762-72737762.png]

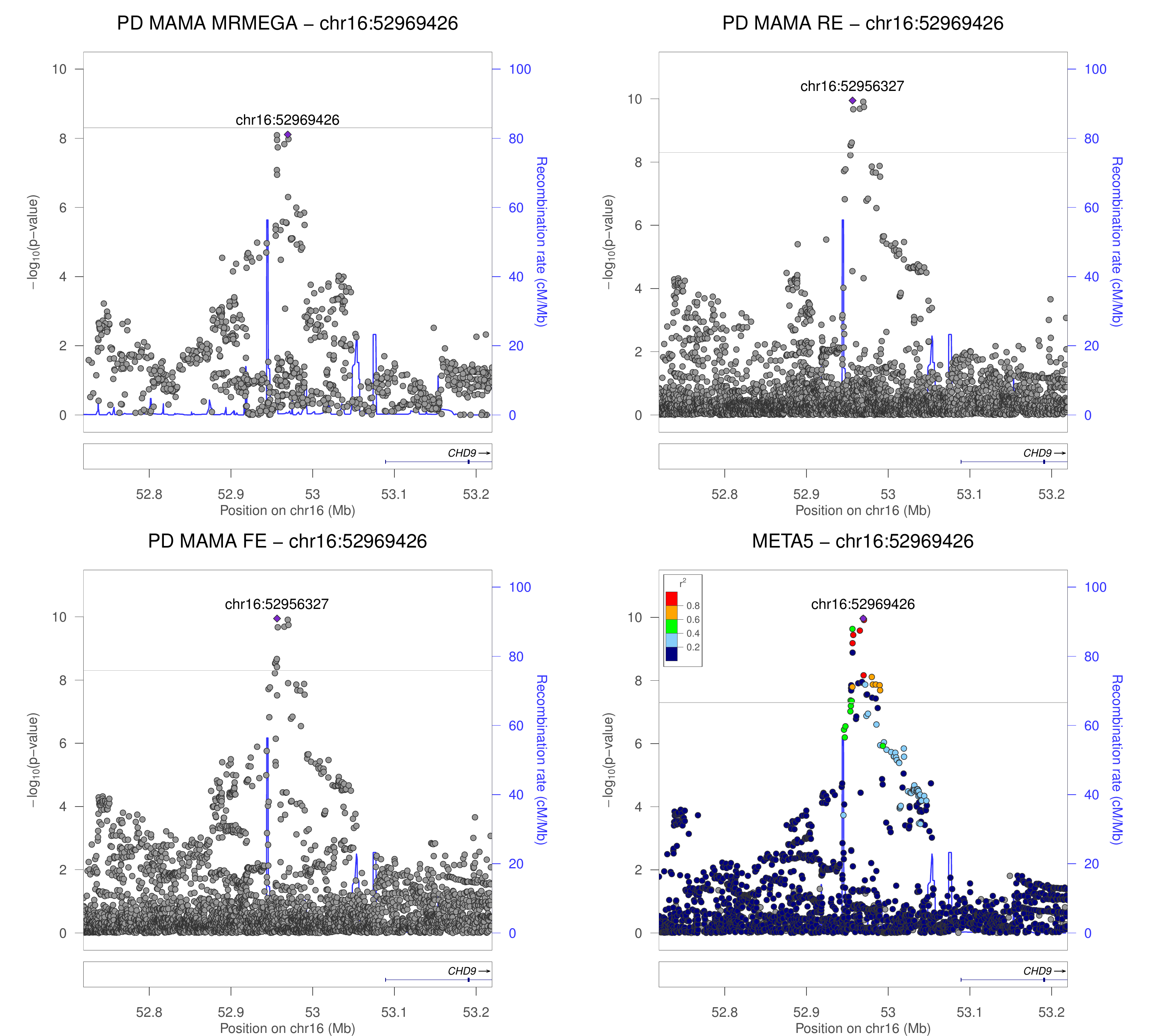

Supplement: Supplementary file 5 — This includes LocusZoom plots of all known European loci as well as novel loci. Each file contains four LocusZoom plots: PD MAMA MR-MEGA/RE/FE/ (MR-MEGA/random-effect/fixed-effect) and META5 (European-only meta-analysis from Nalls et al. 1). [file 41588_2023_1584_MOESM5_ESM.zip › LocusZoom plots of known EUR risk variants/chr16_52719426-53219426.png]

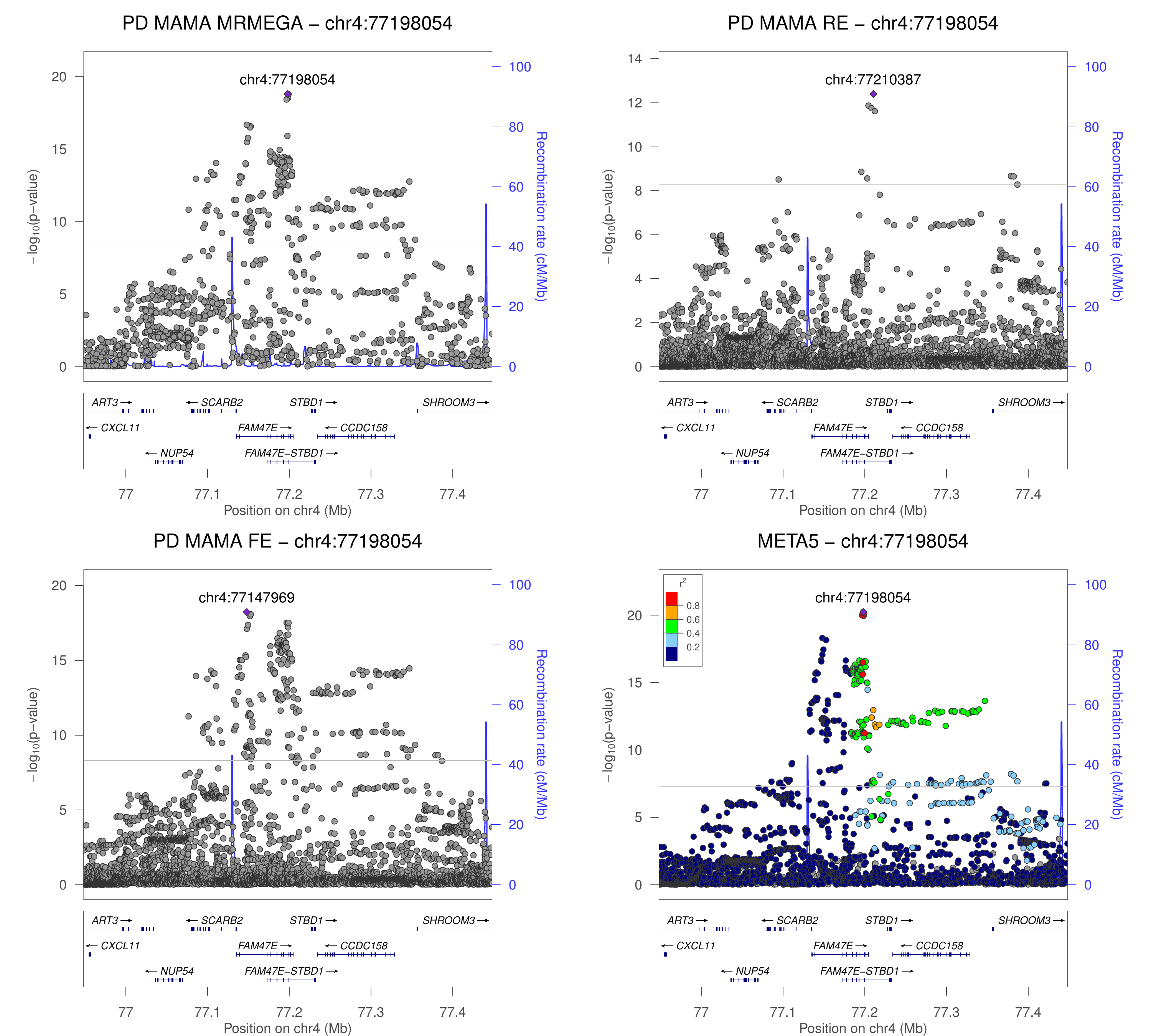

Supplement: Supplementary file 5 — This includes LocusZoom plots of all known European loci as well as novel loci. Each file contains four LocusZoom plots: PD MAMA MR-MEGA/RE/FE/ (MR-MEGA/random-effect/fixed-effect) and META5 (European-only meta-analysis from Nalls et al. 1). [file 41588_2023_1584_MOESM5_ESM.zip › LocusZoom plots of known EUR risk variants/chr4_76948054-77448054.png]

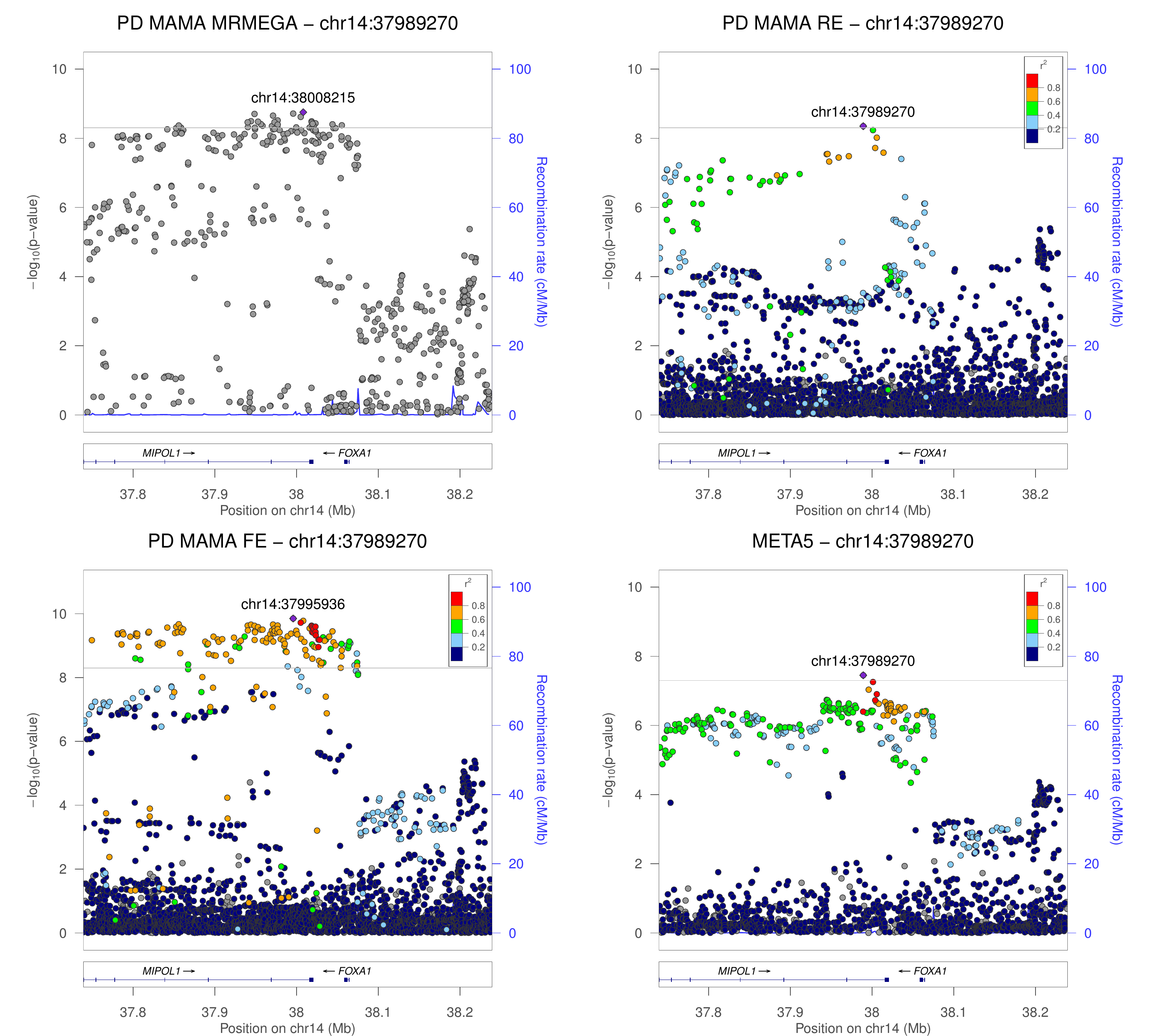

Supplement: Supplementary file 5 — This includes LocusZoom plots of all known European loci as well as novel loci. Each file contains four LocusZoom plots: PD MAMA MR-MEGA/RE/FE/ (MR-MEGA/random-effect/fixed-effect) and META5 (European-only meta-analysis from Nalls et al. 1). [file 41588_2023_1584_MOESM5_ESM.zip › LocusZoom plots of known EUR risk variants/chr14_37739270-38239270.png]

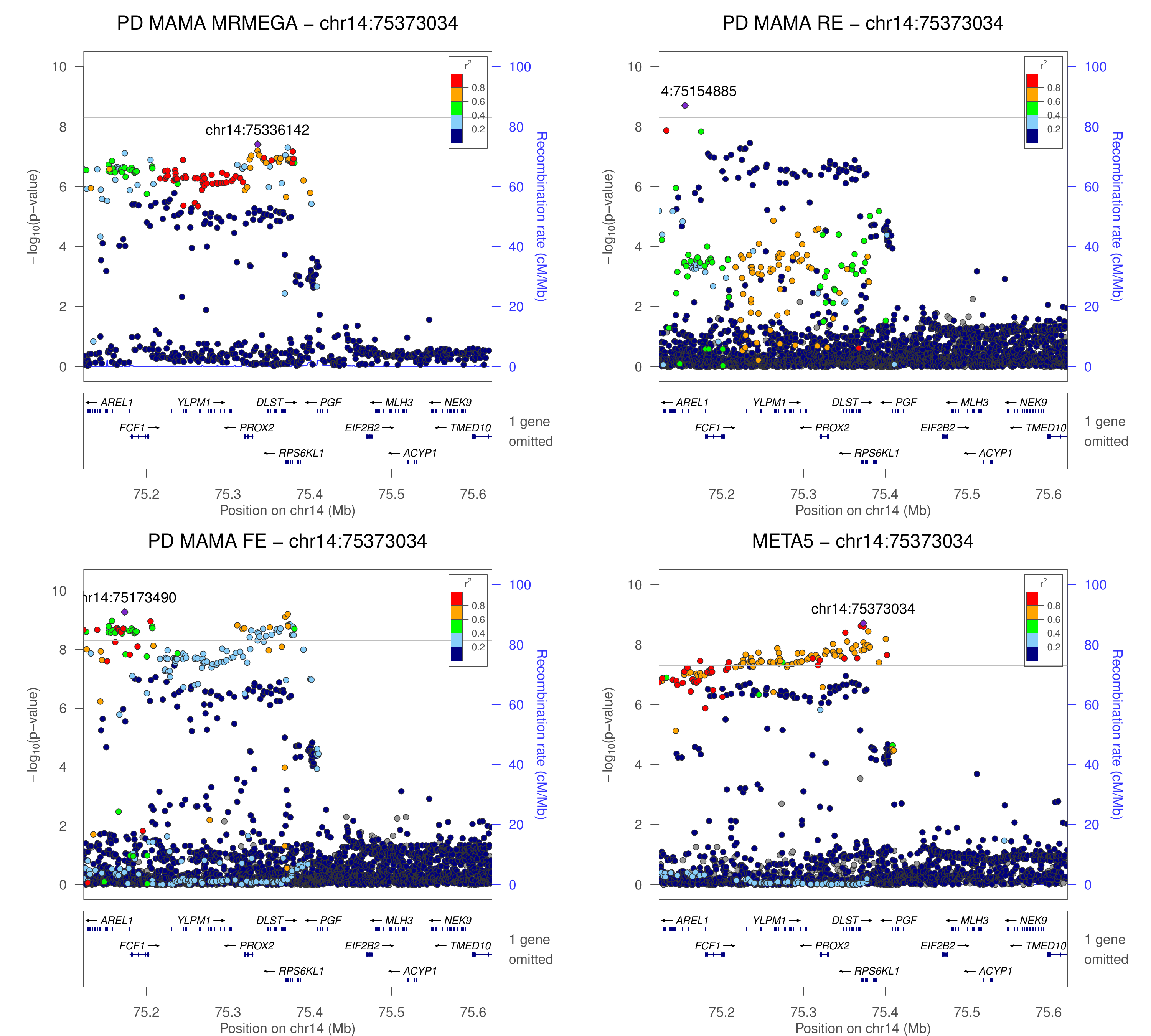

Supplement: Supplementary file 5 — This includes LocusZoom plots of all known European loci as well as novel loci. Each file contains four LocusZoom plots: PD MAMA MR-MEGA/RE/FE/ (MR-MEGA/random-effect/fixed-effect) and META5 (European-only meta-analysis from Nalls et al. 1). [file 41588_2023_1584_MOESM5_ESM.zip › LocusZoom plots of known EUR risk variants/chr14_75123034-75623034.png]

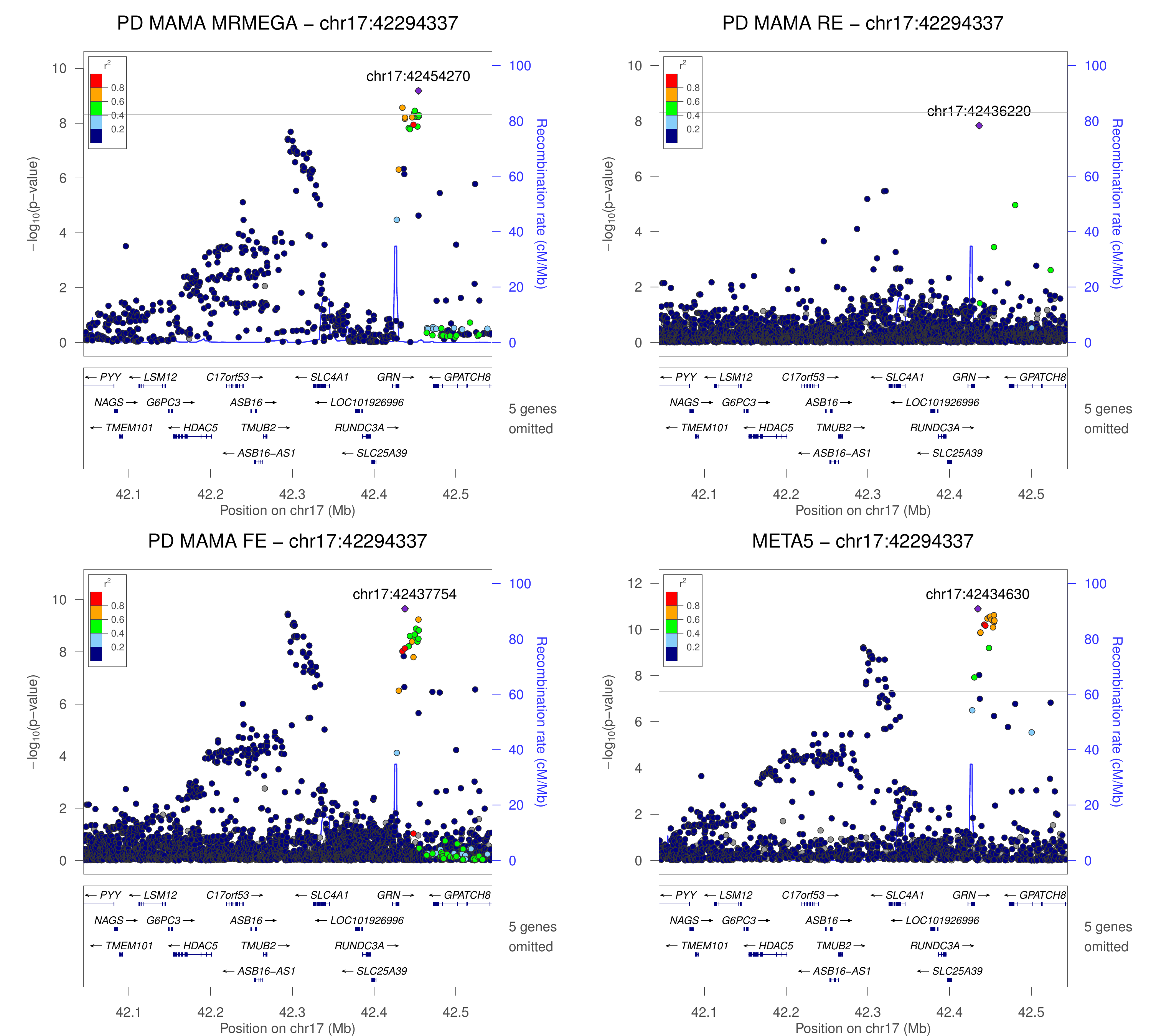

Supplement: Supplementary file 5 — This includes LocusZoom plots of all known European loci as well as novel loci. Each file contains four LocusZoom plots: PD MAMA MR-MEGA/RE/FE/ (MR-MEGA/random-effect/fixed-effect) and META5 (European-only meta-analysis from Nalls et al. 1). [file 41588_2023_1584_MOESM5_ESM.zip › LocusZoom plots of known EUR risk variants/chr17_42044337-42544337.png]

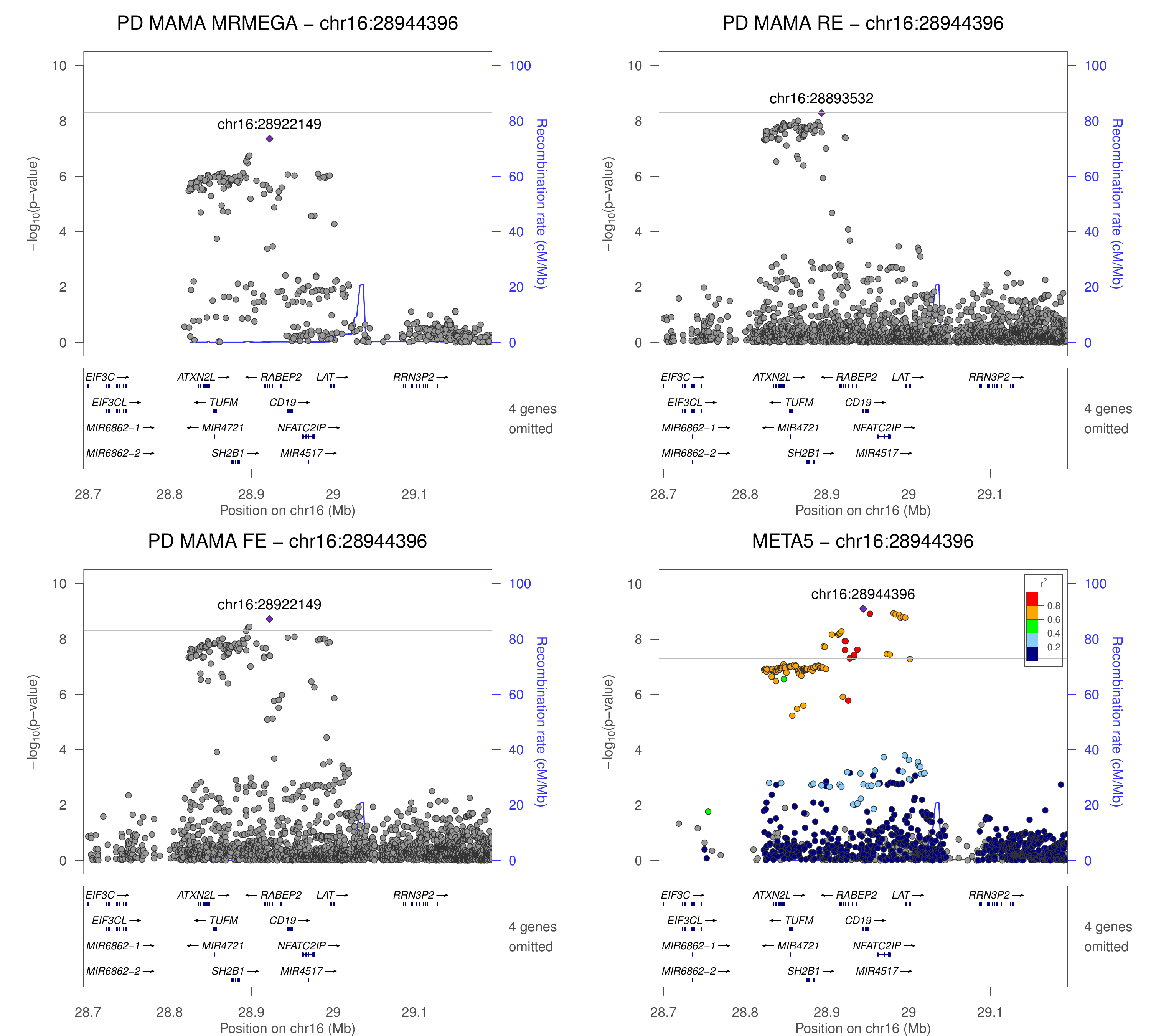

Supplement: Supplementary file 5 — This includes LocusZoom plots of all known European loci as well as novel loci. Each file contains four LocusZoom plots: PD MAMA MR-MEGA/RE/FE/ (MR-MEGA/random-effect/fixed-effect) and META5 (European-only meta-analysis from Nalls et al. 1). [file 41588_2023_1584_MOESM5_ESM.zip › LocusZoom plots of known EUR risk variants/chr16_28694396-29194396.png]

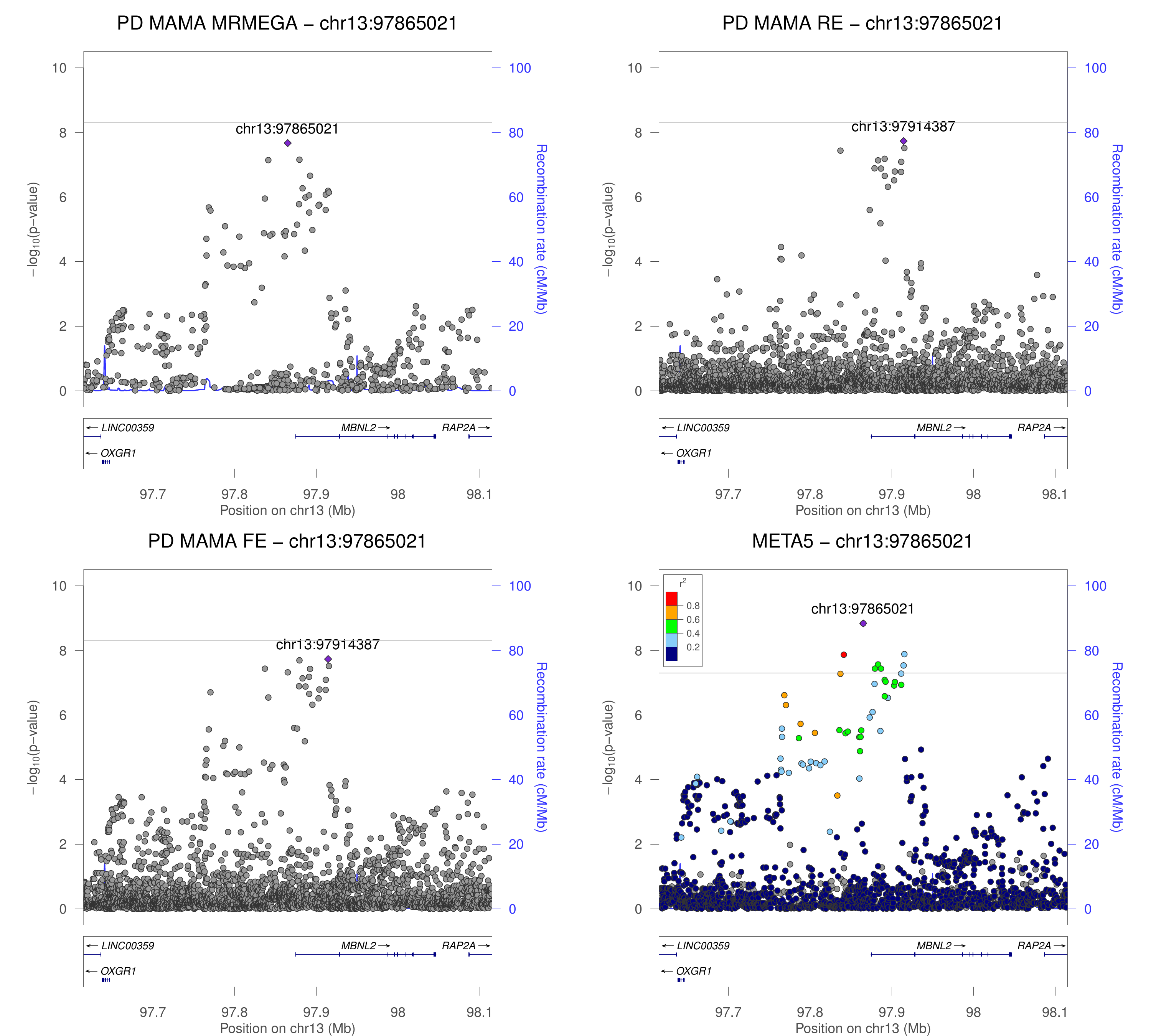

Supplement: Supplementary file 5 — This includes LocusZoom plots of all known European loci as well as novel loci. Each file contains four LocusZoom plots: PD MAMA MR-MEGA/RE/FE/ (MR-MEGA/random-effect/fixed-effect) and META5 (European-only meta-analysis from Nalls et al. 1). [file 41588_2023_1584_MOESM5_ESM.zip › LocusZoom plots of known EUR risk variants/chr13_97615021-98115021.png]

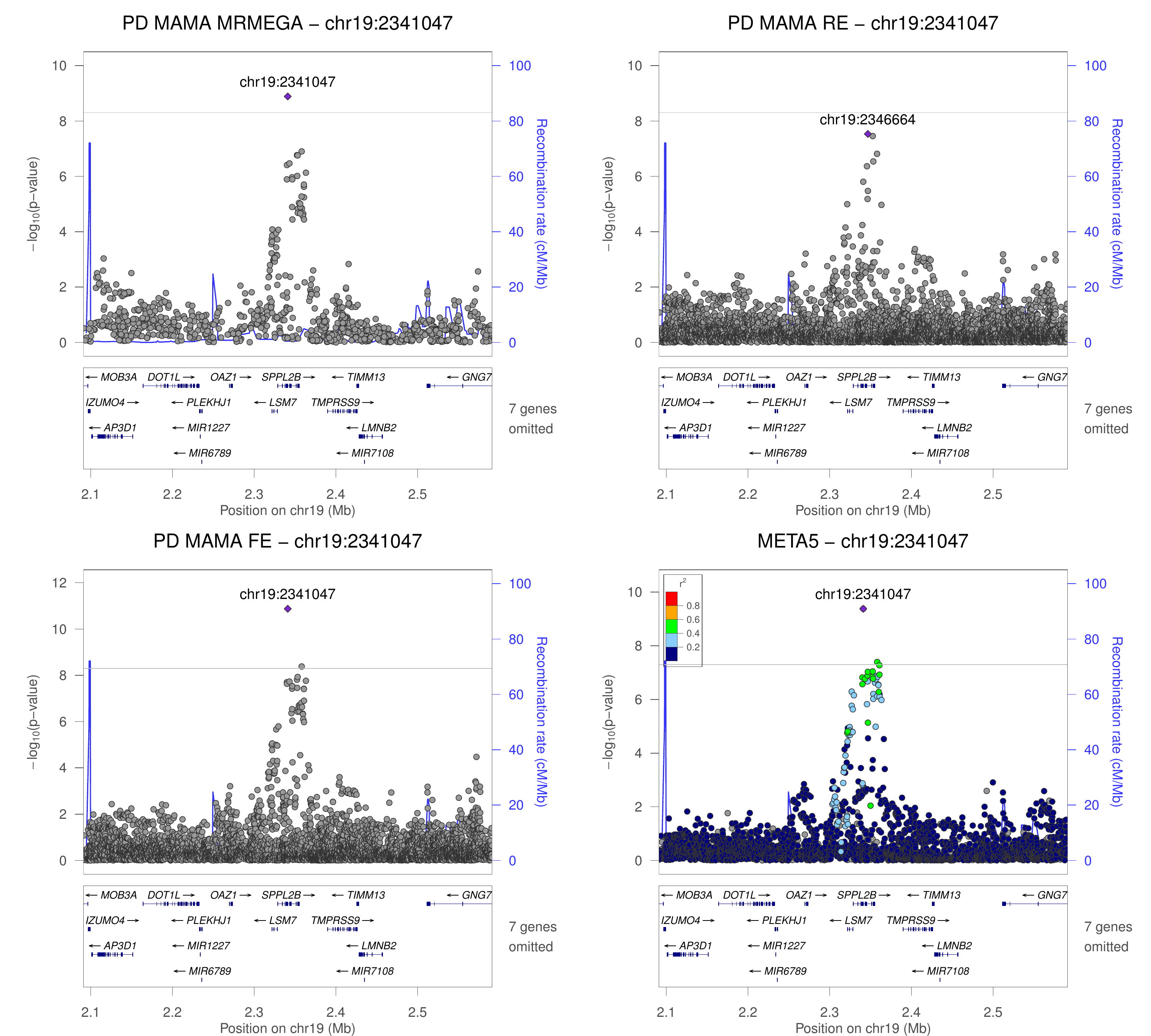

Supplement: Supplementary file 5 — This includes LocusZoom plots of all known European loci as well as novel loci. Each file contains four LocusZoom plots: PD MAMA MR-MEGA/RE/FE/ (MR-MEGA/random-effect/fixed-effect) and META5 (European-only meta-analysis from Nalls et al. 1). [file 41588_2023_1584_MOESM5_ESM.zip › LocusZoom plots of known EUR risk variants/chr19_2091047-2591047.png]

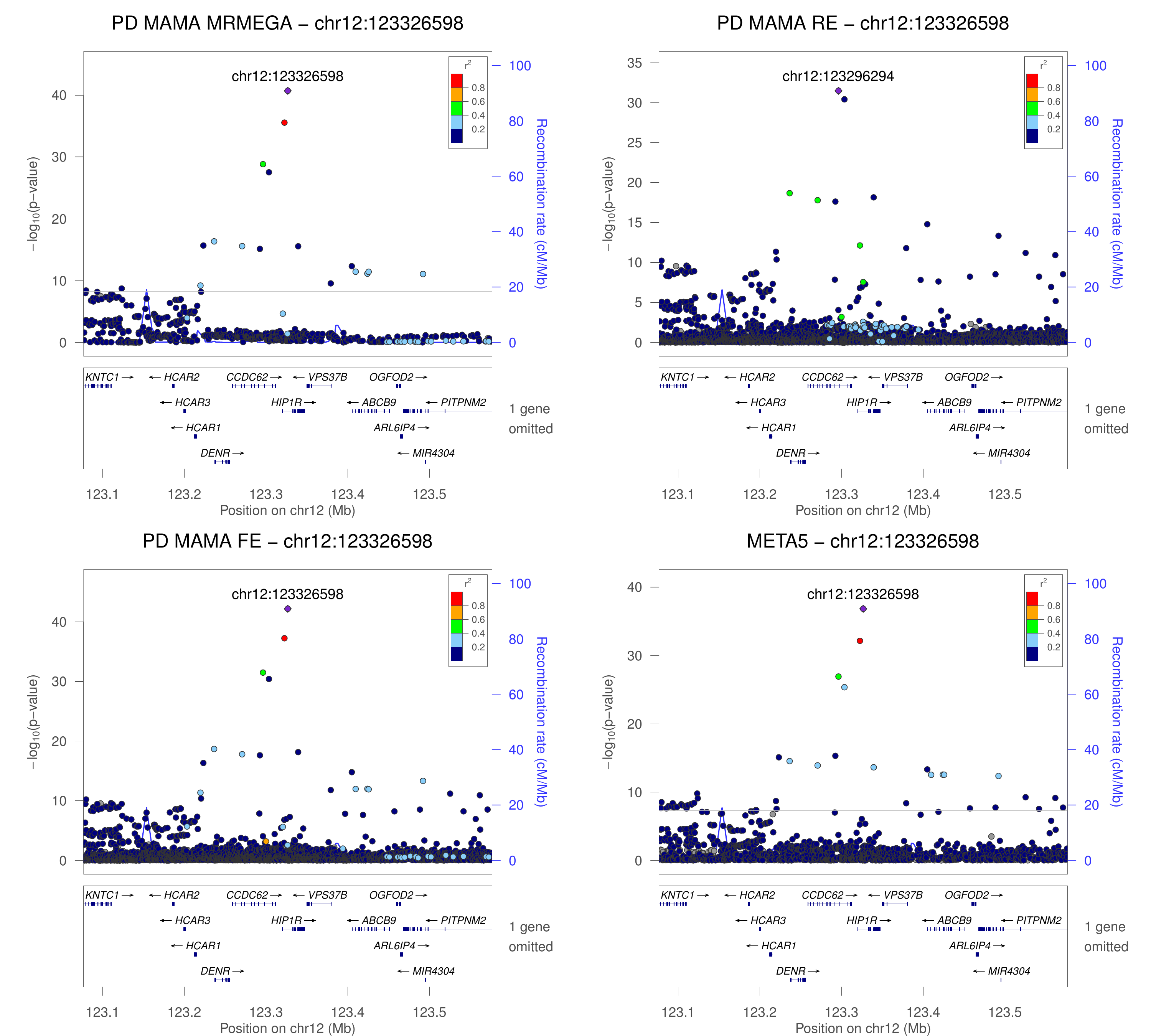

Supplement: Supplementary file 5 — This includes LocusZoom plots of all known European loci as well as novel loci. Each file contains four LocusZoom plots: PD MAMA MR-MEGA/RE/FE/ (MR-MEGA/random-effect/fixed-effect) and META5 (European-only meta-analysis from Nalls et al. 1). [file 41588_2023_1584_MOESM5_ESM.zip › LocusZoom plots of known EUR risk variants/chr12_123076598-123576598.png]

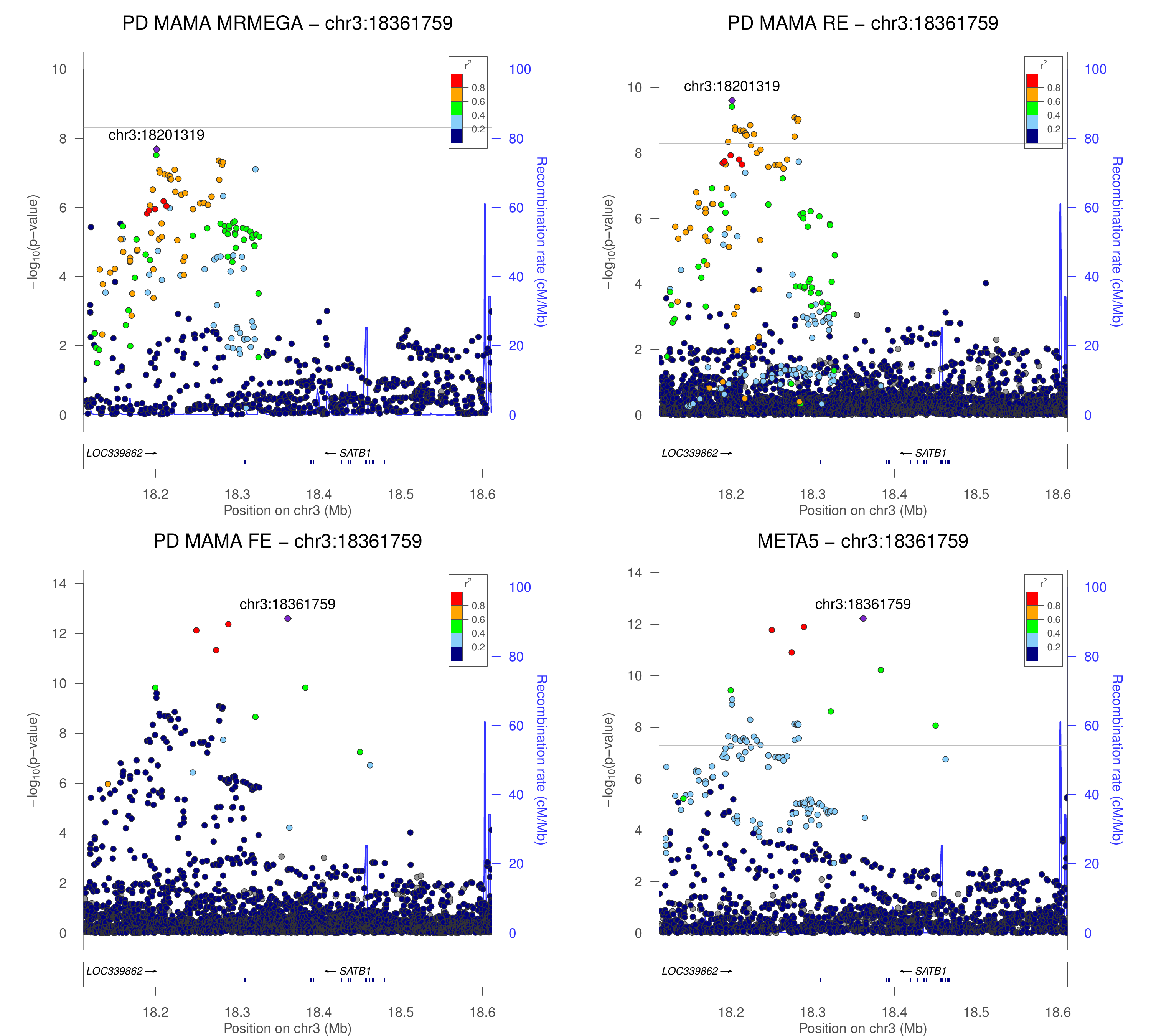

Supplement: Supplementary file 5 — This includes LocusZoom plots of all known European loci as well as novel loci. Each file contains four LocusZoom plots: PD MAMA MR-MEGA/RE/FE/ (MR-MEGA/random-effect/fixed-effect) and META5 (European-only meta-analysis from Nalls et al. 1). [file 41588_2023_1584_MOESM5_ESM.zip › LocusZoom plots of known EUR risk variants/chr3_18111759-18611759.png]

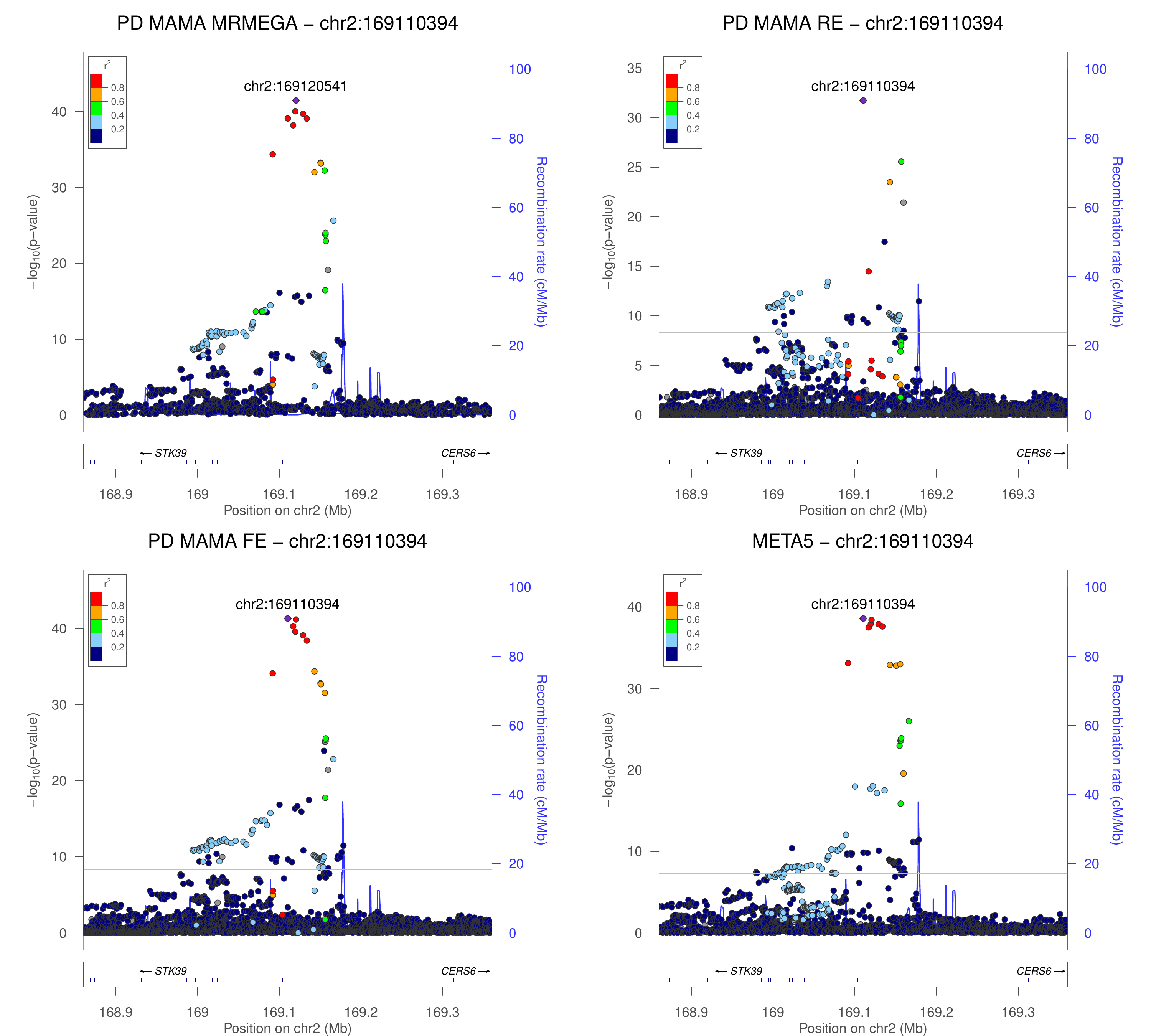

Supplement: Supplementary file 5 — This includes LocusZoom plots of all known European loci as well as novel loci. Each file contains four LocusZoom plots: PD MAMA MR-MEGA/RE/FE/ (MR-MEGA/random-effect/fixed-effect) and META5 (European-only meta-analysis from Nalls et al. 1). [file 41588_2023_1584_MOESM5_ESM.zip › LocusZoom plots of known EUR risk variants/chr2_168860394-169360394.png]

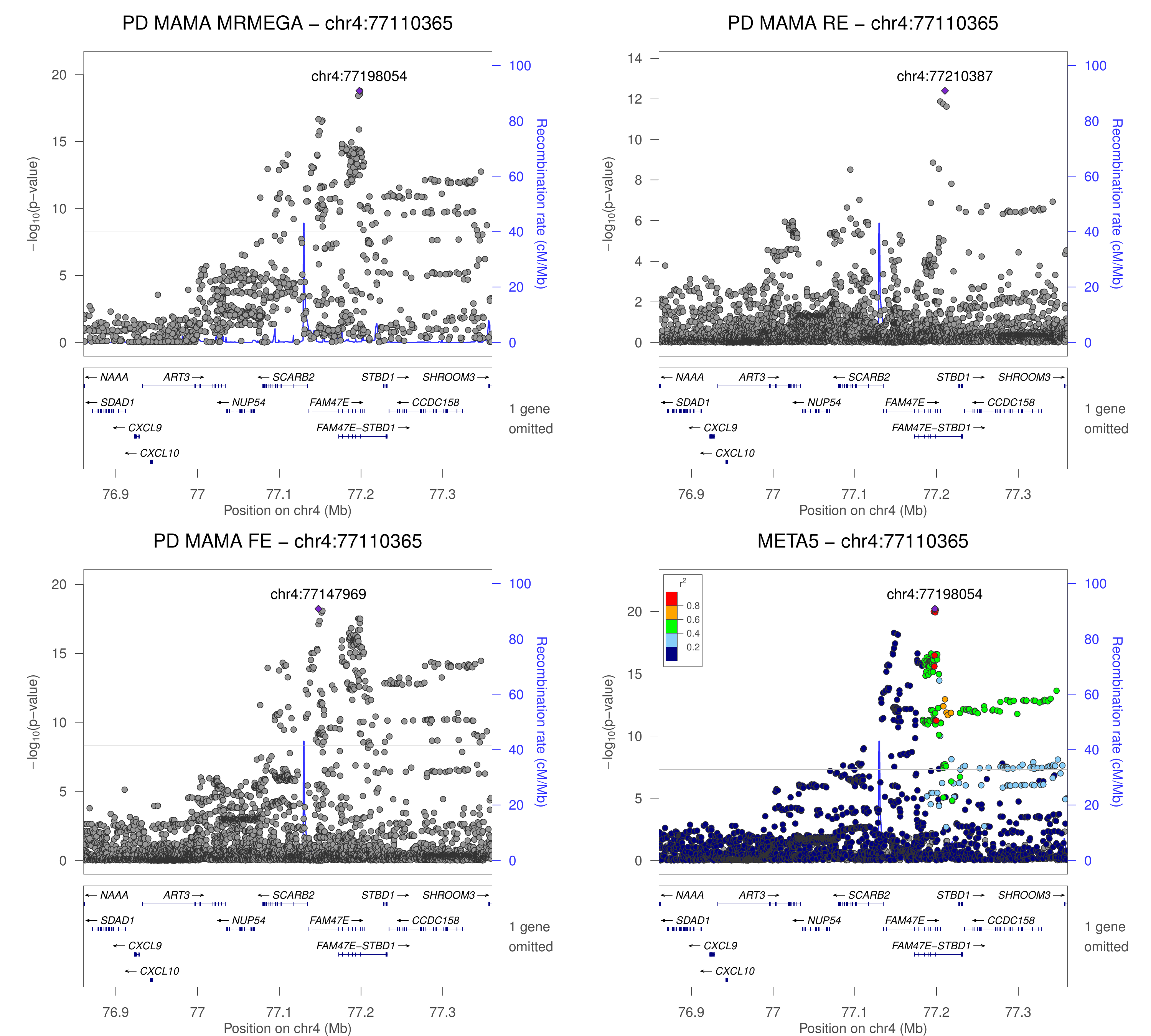

Supplement: Supplementary file 5 — This includes LocusZoom plots of all known European loci as well as novel loci. Each file contains four LocusZoom plots: PD MAMA MR-MEGA/RE/FE/ (MR-MEGA/random-effect/fixed-effect) and META5 (European-only meta-analysis from Nalls et al. 1). [file 41588_2023_1584_MOESM5_ESM.zip › LocusZoom plots of known EUR risk variants/chr4_76860365-77360365.png]

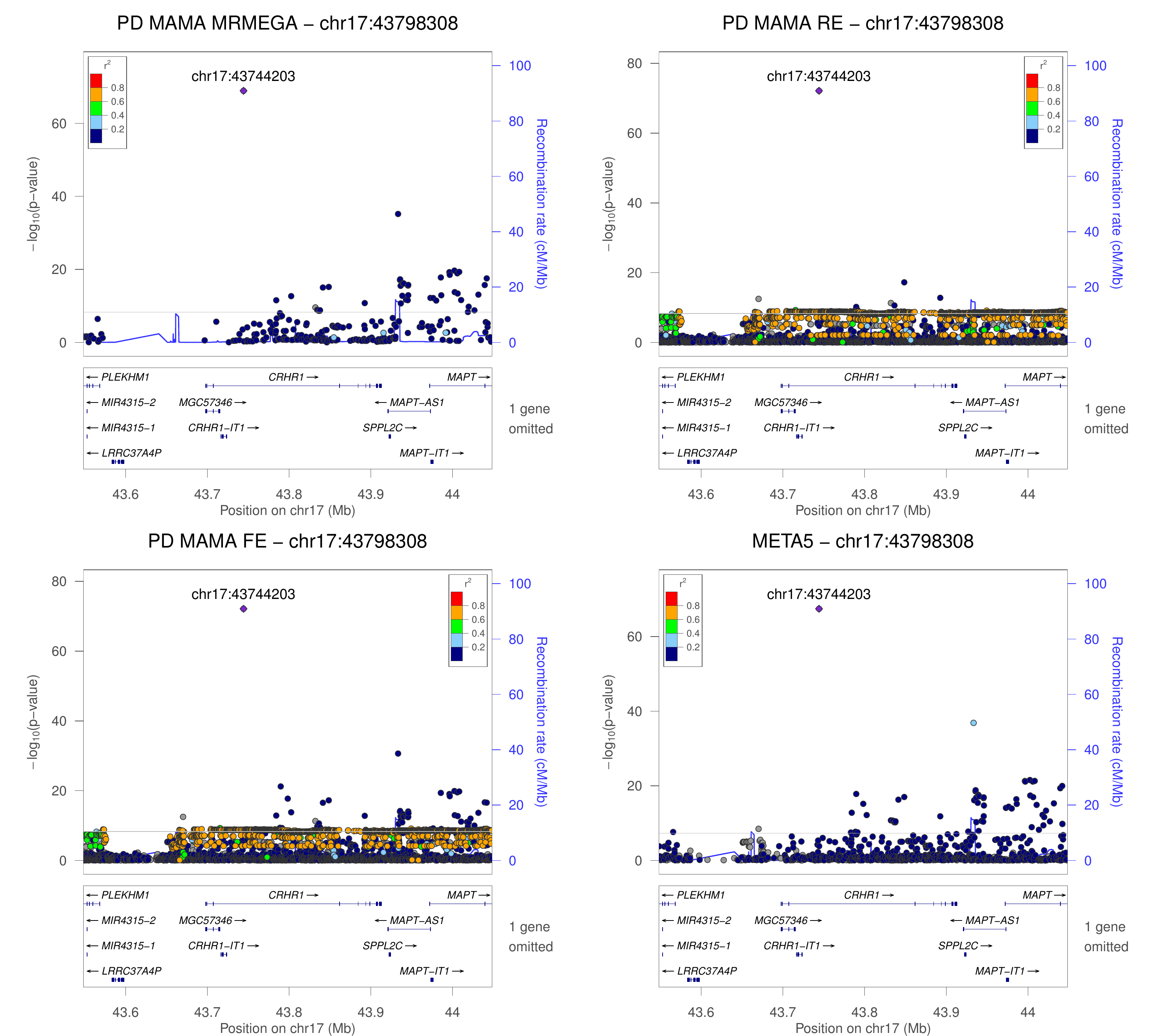

Supplement: Supplementary file 5 — This includes LocusZoom plots of all known European loci as well as novel loci. Each file contains four LocusZoom plots: PD MAMA MR-MEGA/RE/FE/ (MR-MEGA/random-effect/fixed-effect) and META5 (European-only meta-analysis from Nalls et al. 1). [file 41588_2023_1584_MOESM5_ESM.zip › LocusZoom plots of known EUR risk variants/chr17_43548308-44048308.png]

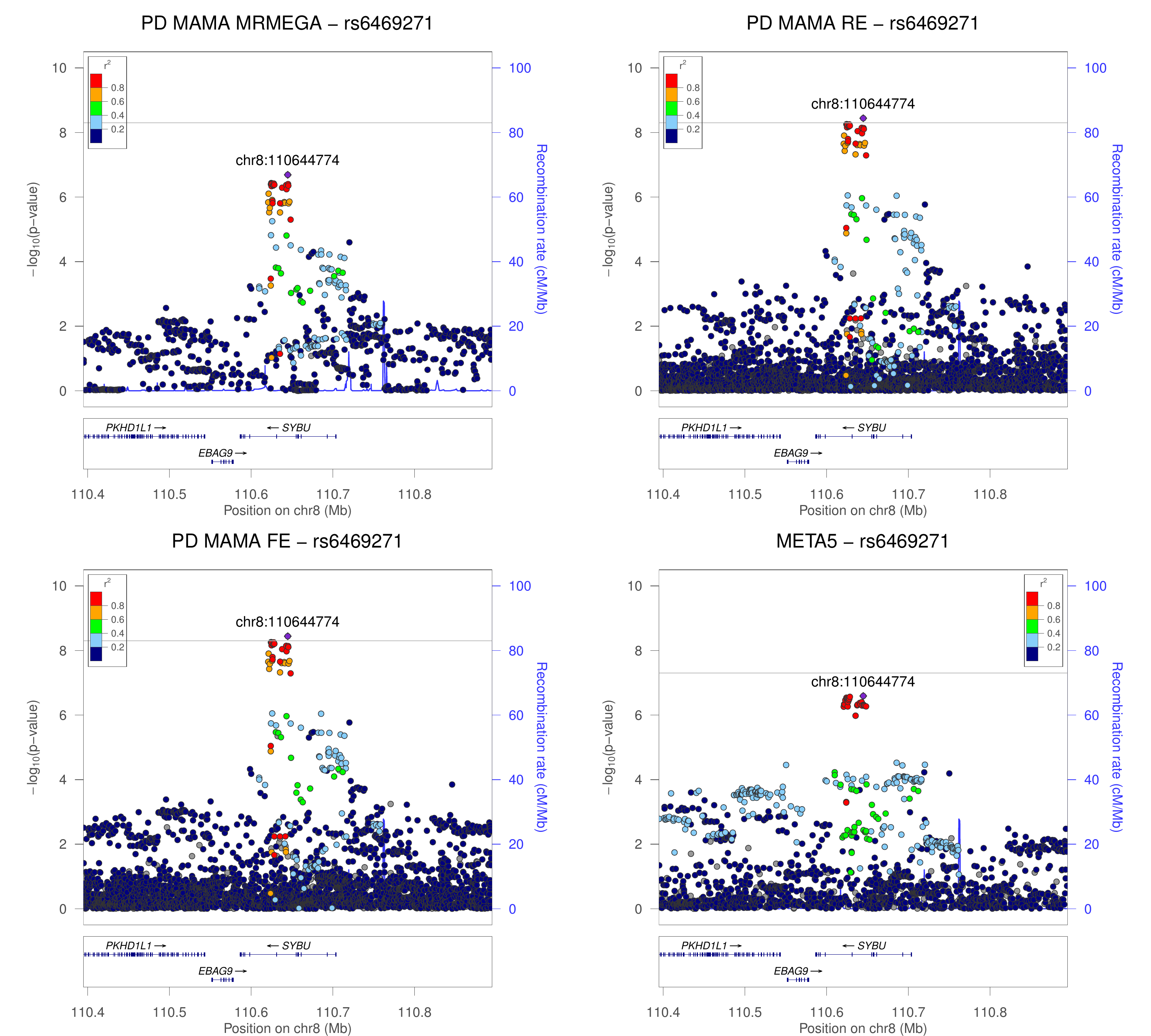

Supplement: Supplementary file 5 — This includes LocusZoom plots of all known European loci as well as novel loci. Each file contains four LocusZoom plots: PD MAMA MR-MEGA/RE/FE/ (MR-MEGA/random-effect/fixed-effect) and META5 (European-only meta-analysis from Nalls et al. 1). [file 41588_2023_1584_MOESM5_ESM.zip › LocusZoom plots of nominated novel loci/chr8_110394774-110894774.png]

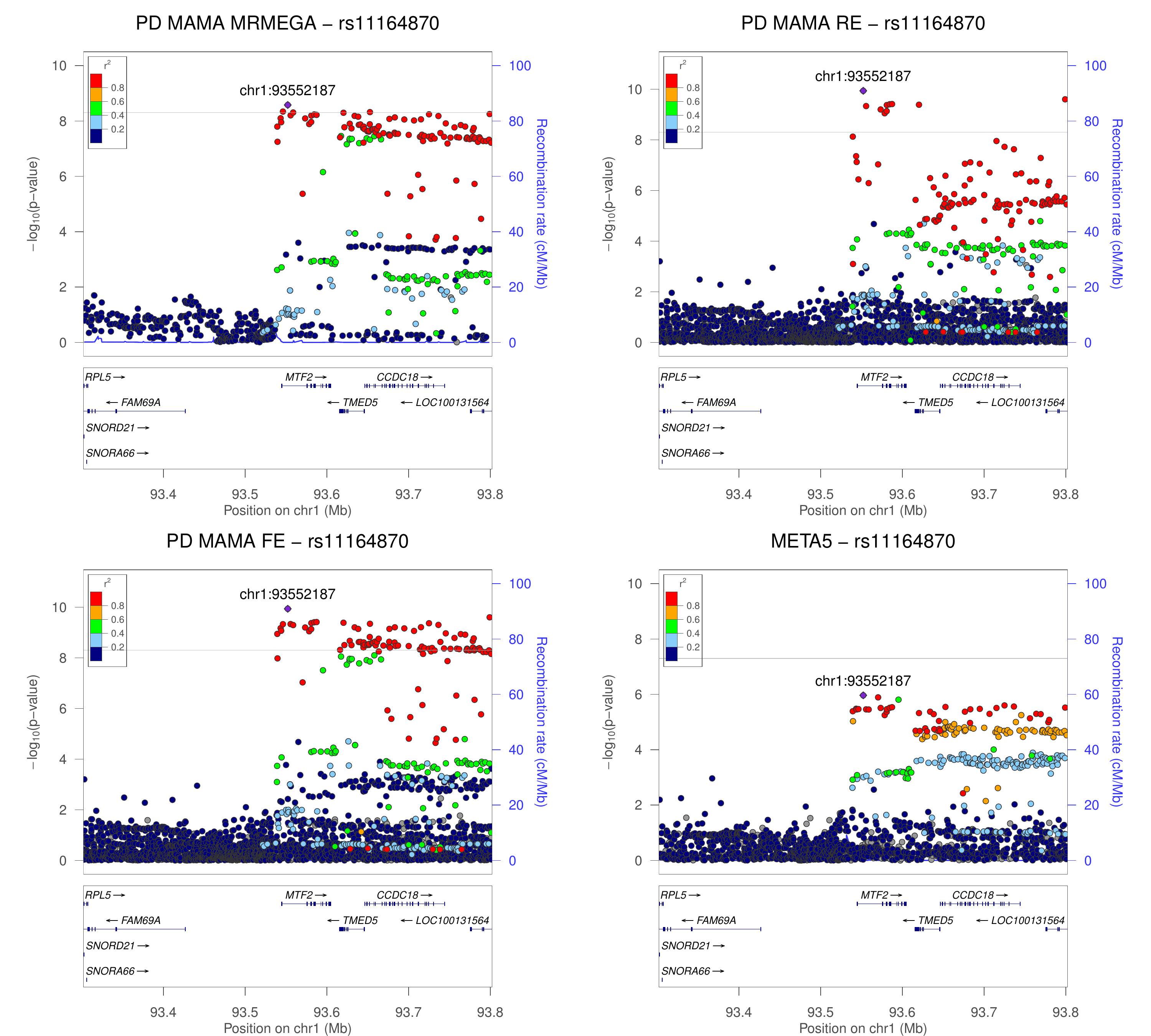

Supplement: Supplementary file 5 — This includes LocusZoom plots of all known European loci as well as novel loci. Each file contains four LocusZoom plots: PD MAMA MR-MEGA/RE/FE/ (MR-MEGA/random-effect/fixed-effect) and META5 (European-only meta-analysis from Nalls et al. 1). [file 41588_2023_1584_MOESM5_ESM.zip › LocusZoom plots of nominated novel loci/chr1_93302187-93802187.png]

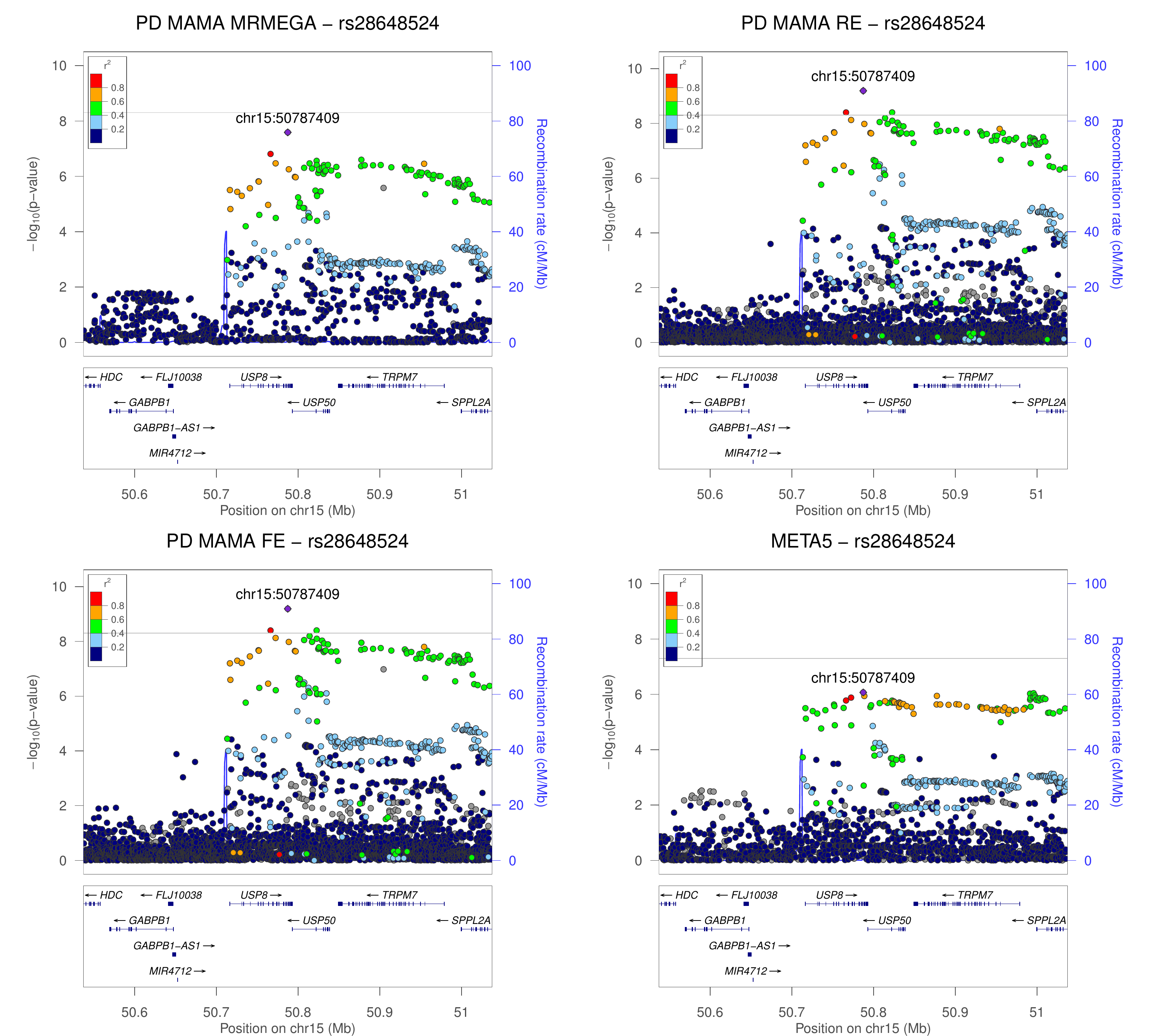

Supplement: Supplementary file 5 — This includes LocusZoom plots of all known European loci as well as novel loci. Each file contains four LocusZoom plots: PD MAMA MR-MEGA/RE/FE/ (MR-MEGA/random-effect/fixed-effect) and META5 (European-only meta-analysis from Nalls et al. 1). [file 41588_2023_1584_MOESM5_ESM.zip › LocusZoom plots of nominated novel loci/chr15_50537409-51037409.png]

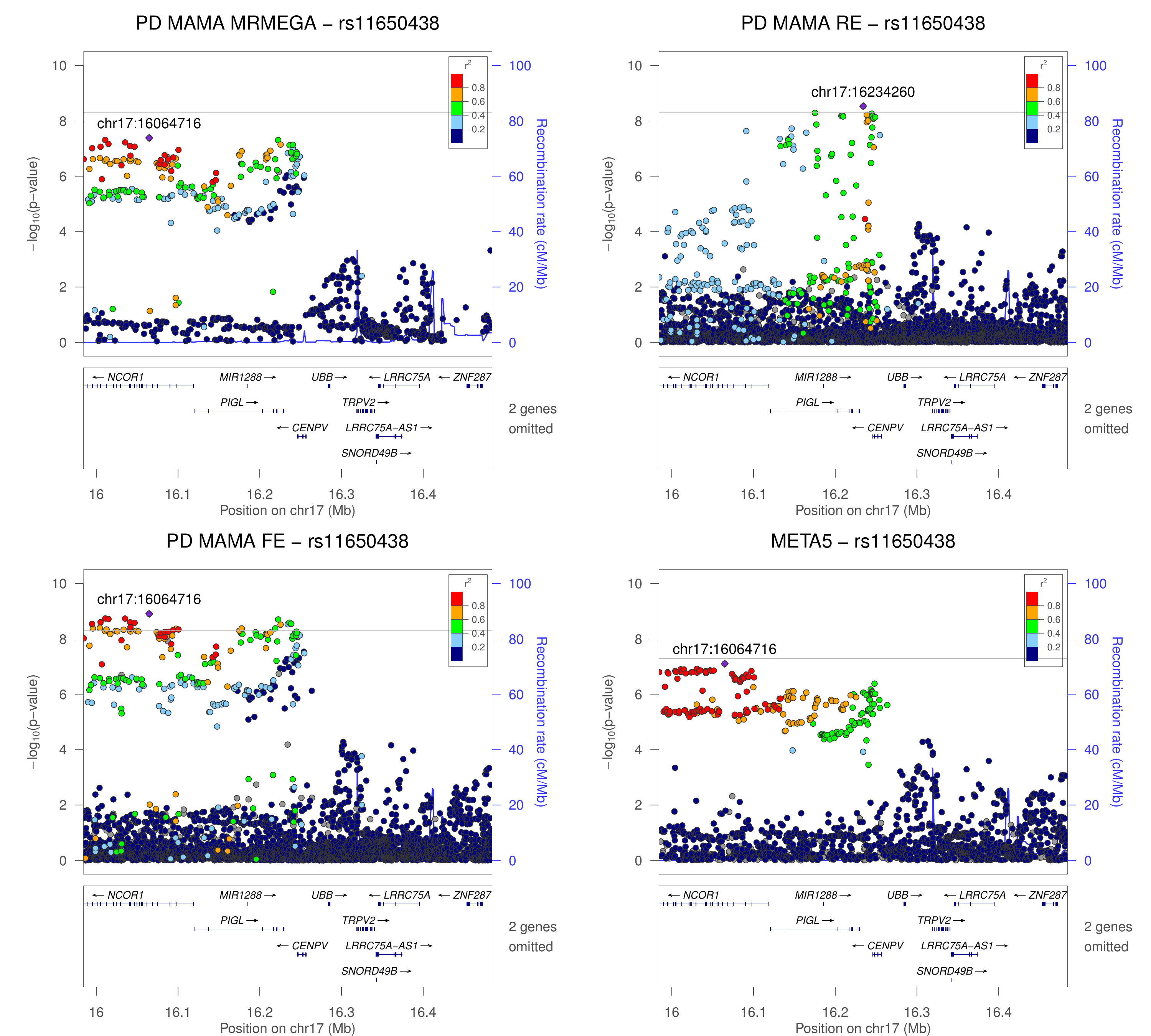

Supplement: Supplementary file 5 — This includes LocusZoom plots of all known European loci as well as novel loci. Each file contains four LocusZoom plots: PD MAMA MR-MEGA/RE/FE/ (MR-MEGA/random-effect/fixed-effect) and META5 (European-only meta-analysis from Nalls et al. 1). [file 41588_2023_1584_MOESM5_ESM.zip › LocusZoom plots of nominated novel loci/chr17_15984260-16484260.png]

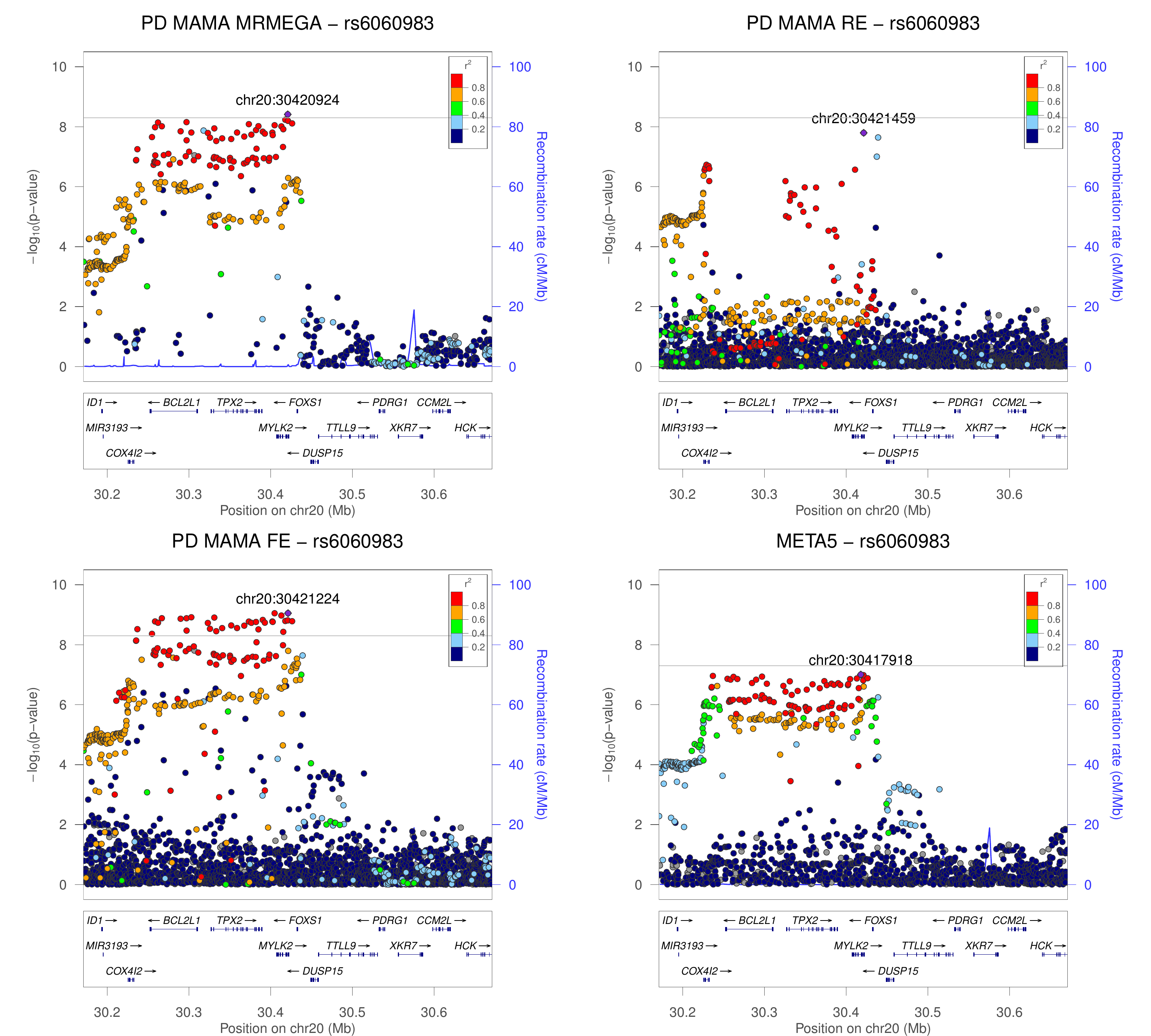

Supplement: Supplementary file 5 — This includes LocusZoom plots of all known European loci as well as novel loci. Each file contains four LocusZoom plots: PD MAMA MR-MEGA/RE/FE/ (MR-MEGA/random-effect/fixed-effect) and META5 (European-only meta-analysis from Nalls et al. 1). [file 41588_2023_1584_MOESM5_ESM.zip › LocusZoom plots of nominated novel loci/chr20_30170924-30670924.png]

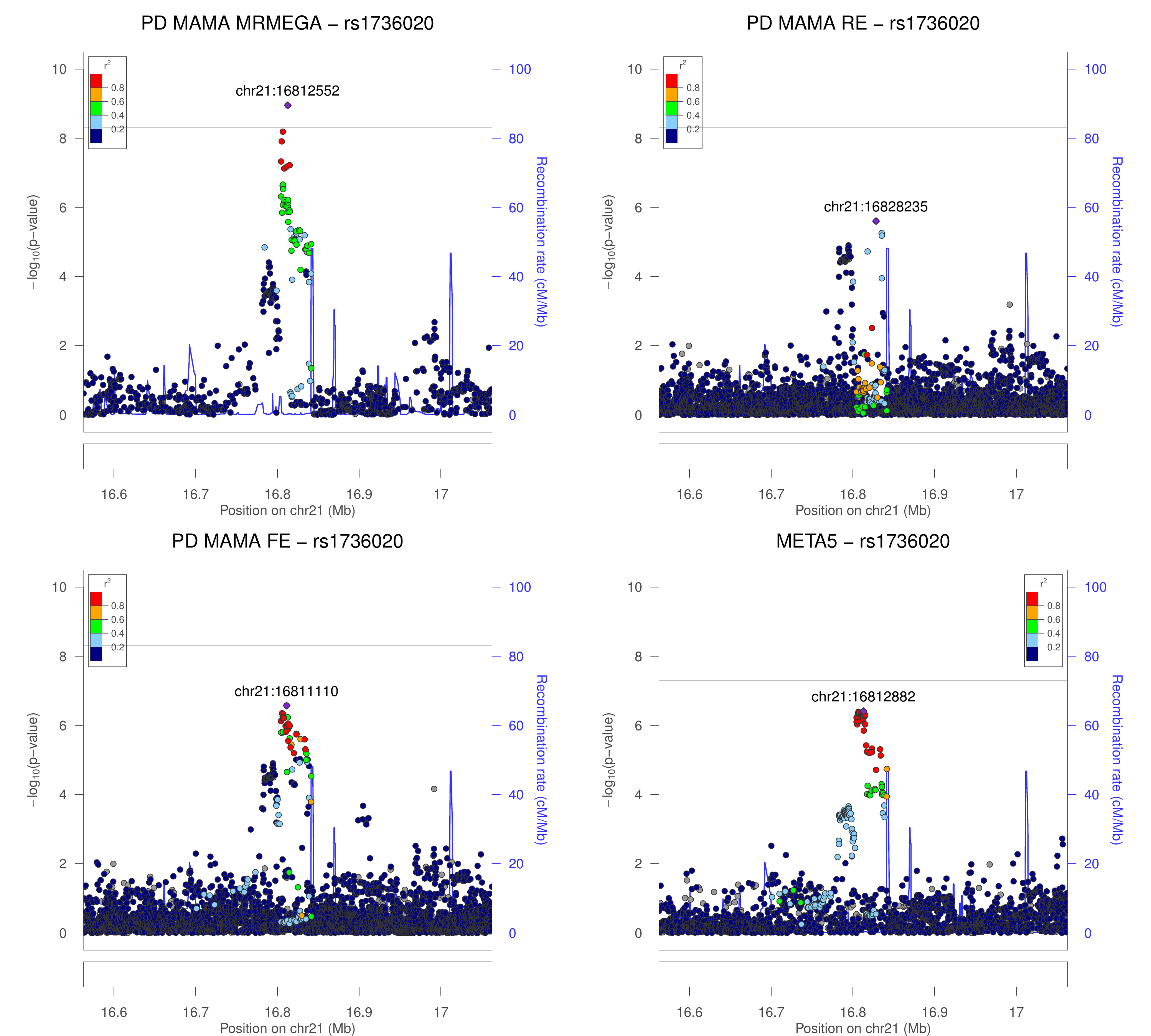

Supplement: Supplementary file 5 — This includes LocusZoom plots of all known European loci as well as novel loci. Each file contains four LocusZoom plots: PD MAMA MR-MEGA/RE/FE/ (MR-MEGA/random-effect/fixed-effect) and META5 (European-only meta-analysis from Nalls et al. 1). [file 41588_2023_1584_MOESM5_ESM.zip › LocusZoom plots of nominated novel loci/chr21_16562552-17062552.png]

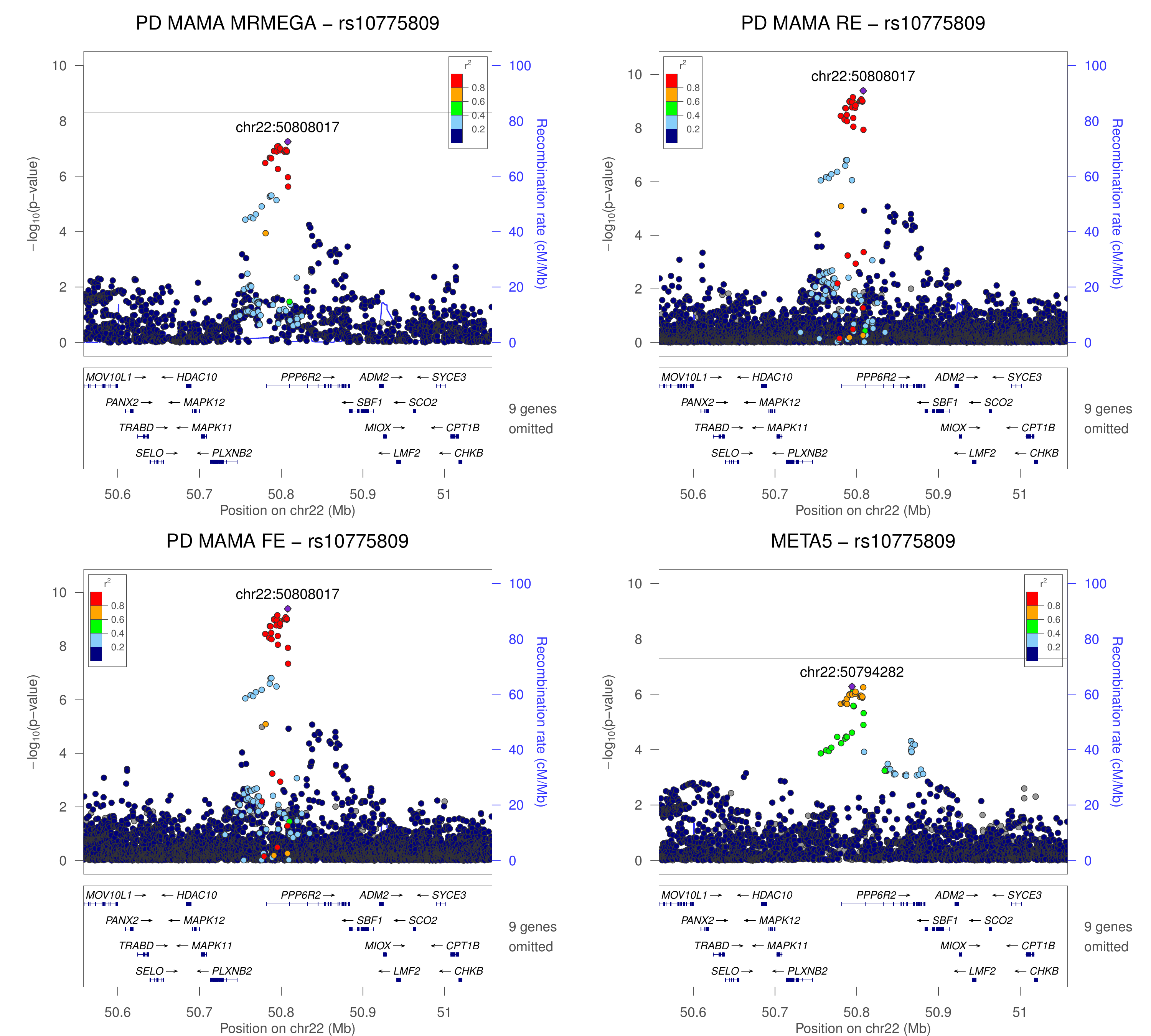

Supplement: Supplementary file 5 — This includes LocusZoom plots of all known European loci as well as novel loci. Each file contains four LocusZoom plots: PD MAMA MR-MEGA/RE/FE/ (MR-MEGA/random-effect/fixed-effect) and META5 (European-only meta-analysis from Nalls et al. 1). [file 41588_2023_1584_MOESM5_ESM.zip › LocusZoom plots of nominated novel loci/chr22_50558017-51058017.png]

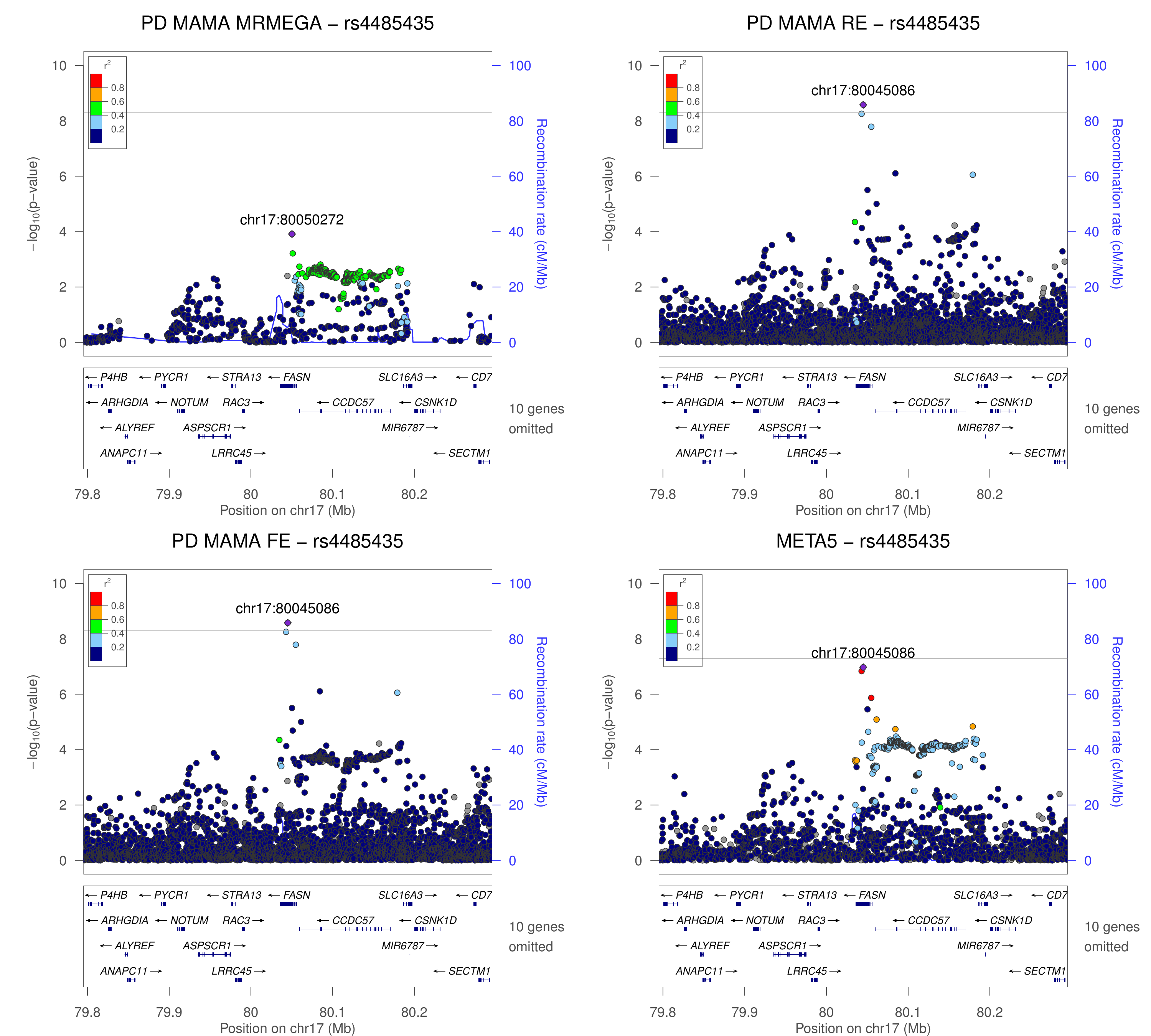

Supplement: Supplementary file 5 — This includes LocusZoom plots of all known European loci as well as novel loci. Each file contains four LocusZoom plots: PD MAMA MR-MEGA/RE/FE/ (MR-MEGA/random-effect/fixed-effect) and META5 (European-only meta-analysis from Nalls et al. 1). [file 41588_2023_1584_MOESM5_ESM.zip › LocusZoom plots of nominated novel loci/chr17_79795086-80295086.png]

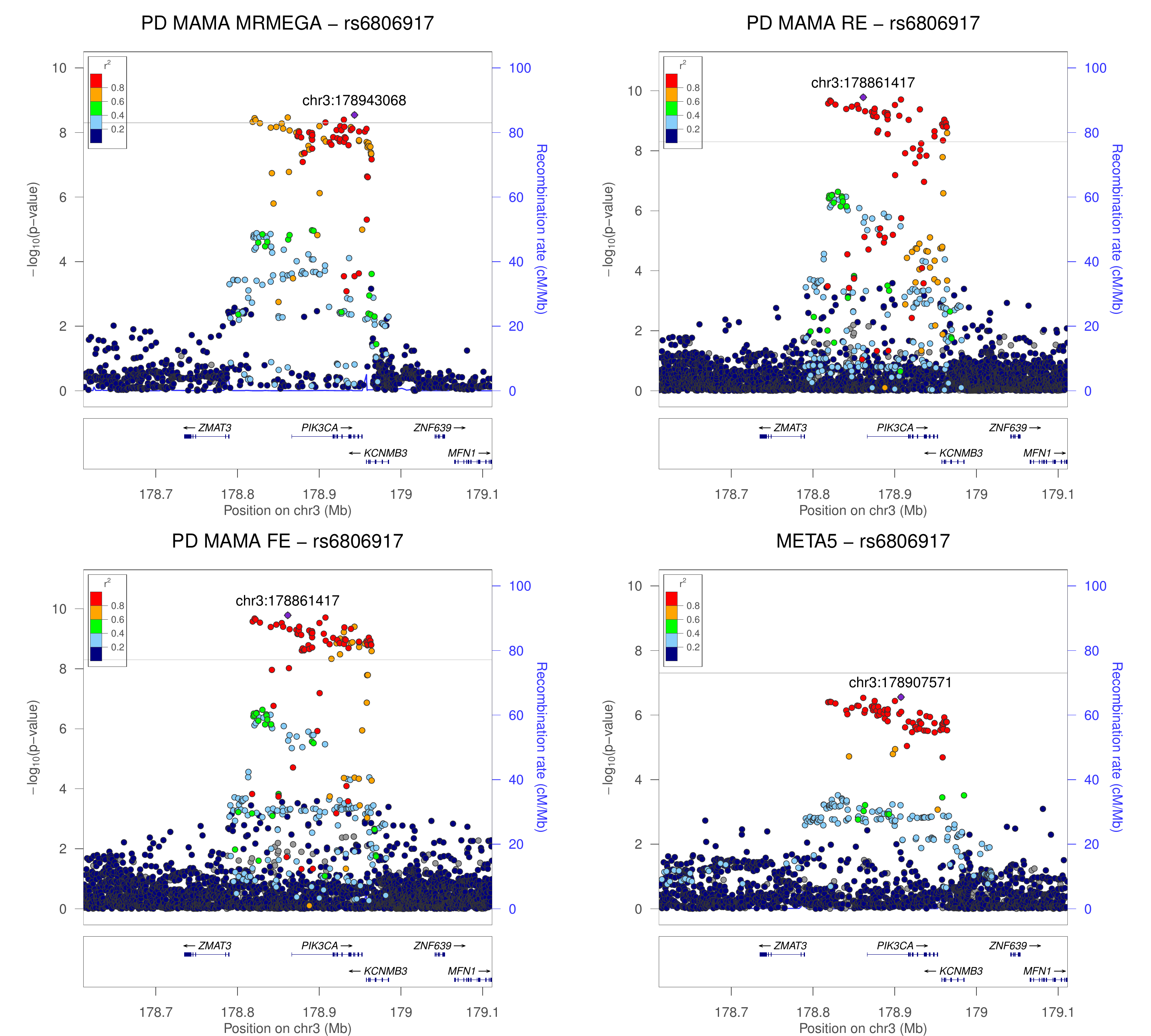

Supplement: Supplementary file 5 — This includes LocusZoom plots of all known European loci as well as novel loci. Each file contains four LocusZoom plots: PD MAMA MR-MEGA/RE/FE/ (MR-MEGA/random-effect/fixed-effect) and META5 (European-only meta-analysis from Nalls et al. 1). [file 41588_2023_1584_MOESM5_ESM.zip › LocusZoom plots of nominated novel loci/chr3_178611417-179111417.png]

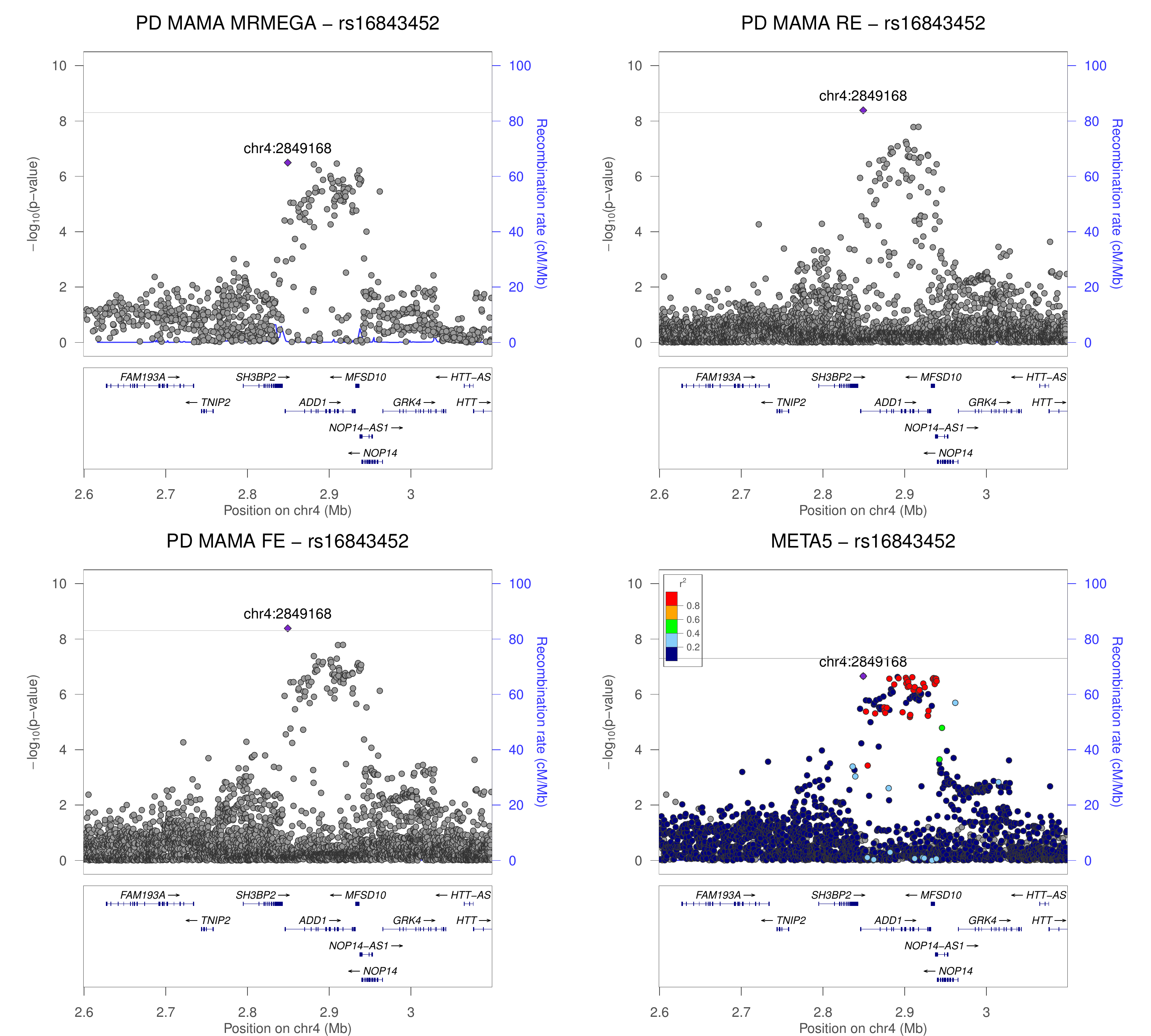

Supplement: Supplementary file 5 — This includes LocusZoom plots of all known European loci as well as novel loci. Each file contains four LocusZoom plots: PD MAMA MR-MEGA/RE/FE/ (MR-MEGA/random-effect/fixed-effect) and META5 (European-only meta-analysis from Nalls et al. 1). [file 41588_2023_1584_MOESM5_ESM.zip › LocusZoom plots of nominated novel loci/chr4_2599168-3099168.png]
